# Tuning the energetics of carbonyl-bridged triarylamines: from thermally activated delayed fluorescence to anti-Kasha dual-emission and room temperature phosphorescence materials†

**DOI:** 10.1039/d5sc02096d

**Published:** 2025-05-22

**Authors:** Liqiu Wan, Sisi Ling, Lei Yang, Bijin Li

**Affiliations:** a Engineering Research Center of the Ministry of Education for the Development of Targeted Anti-tumors and Anti-pathogens New Drugs & Chongqing Key Laboratory of Natural Product Synthesis and Drug Research, School of Pharmaceutical Sciences, Chongqing University Chongqing 401331 P. R. China bijinli@cqu.edu.cn

## Abstract

The first example of tuning the energetics of thermally activated delayed fluorescence (TADF) molecules to access anti-Kasha dual-emission and room-temperature phosphorescence materials through a strategy for structural modification is developed here. To rapidly construct a library of structurally diverse carbonyl-bridged triarylamine-based TADF materials, the copper-mediated cyclization of heterocycles and 2-bromobenzoic acids was further developed for the first time. Novel anti-Kasha dual-emission materials exhibit distinct white light emission with CIE coordinates of (0.32, 0.32) in the solid film and can be fabricated into low-cost and robust organic white LEDs. Ultralong room temperature organic phosphorescence (URTP) materials displayed a cyan afterglow for up to seven seconds with a lifetime of 508.8 ms and could be used in encryption.

## Abstract 2

This work reports the first example of the copper-mediated cyclization reaction to access thermally activated delayed fluorescence molecules, anti-Kasha dual-emission materials, and room-temperature phosphorescence materials.

## Graphical abstract

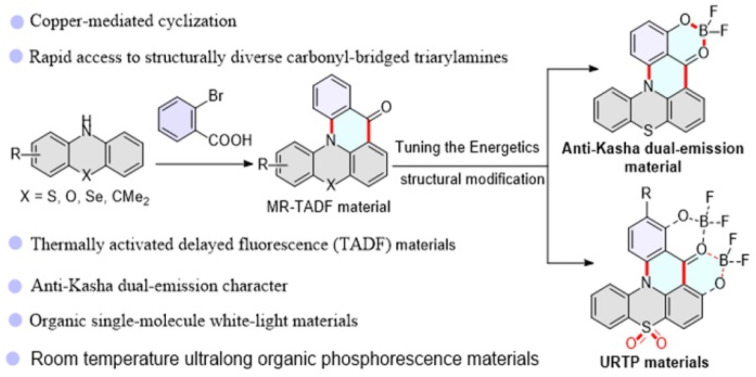

## Introduction

Novel luminescent materials, especially multiple resonance thermally activated delayed fluorescence (MR-TADF) materials, anti-Kasha dual-emission materials, and room-temperature ultralong organic phosphorescence materials, have been attracting considerable attention in recent years due to their potential to enable new technologies in lighting, optical sensing, and imaging.^1–12^ MR-TADF materials hold great potential as emitters owing to their narrowband emission and 100% exciton utilization in devices.^1–3,13–16^ Anti-Kasha dual-emission molecules have potential applications in highly accurate analysis of basic life science and single-molecule white organic light-emitting materials.^4–7,17–25^ Ultralong room temperature organic phosphorescence (URTP) materials show interesting applications in bio-imaging, anticounterfeiting, information storage, emergency signs, and optoelectronic devices due to their unique advantages such as high signal-to-noise ratio, large Stokes shift, long emission lifetimes, and rich excited states.^8–12,26–32^ Hence, developing MR-TADF, anti-Kasha dual-emission, and URTP materials has been attracting much research interest from both scientific and engineering arenas.^1–13,23–28^ However, it is still a great challenge to discover these novel luminescent materials due to the intrinsic limitation of the photophysical properties of organic molecules.^1–12^

**Scheme 1:**
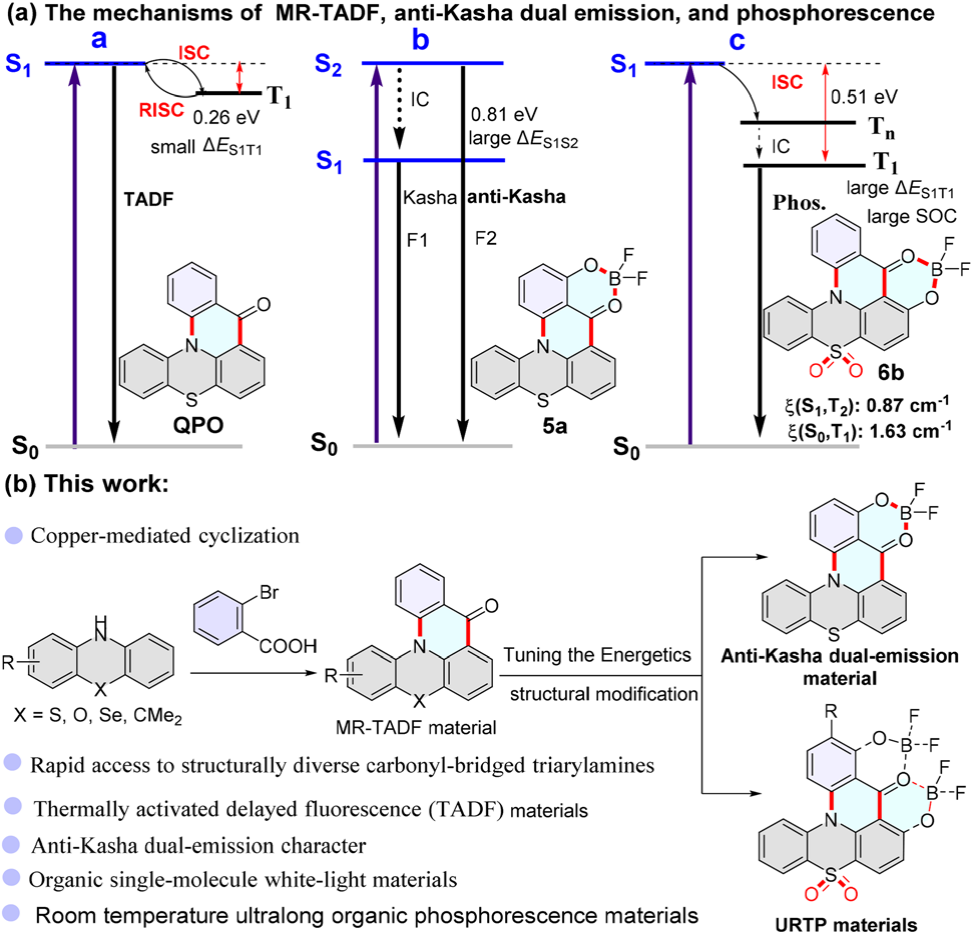
The structure and synthesis route of MR-TADF material-based carbonyl-bridged triphenylamine (N/C=O-based core) compounds.

MR-TADF materials need to satisfy a small singlet-triplet energy gap (Δ*E*_ST_) because a small energy barrier facilitates reverse intersystem crossing (RISC) of excitons from the lowest triplet-excited state (T_1_) to the lowest singlet-excited state (S_1_) (Scheme 1a).^1–3,33,34^ Anti-Kasha dual-emission fluorescence materials are dependent on a large energy gap between adjacent excited states, which leads to internal conversion (IC) from the S_*n*_ (*n* ≥ 2) to the S_*n*−1_ state being comparatively slow, and thus S_*n*_ fluorescence can compete favorably with IC (Scheme 1a).^4–7,35–39^ URTP materials often require a relatively large gap Δ*E*_S1T1_ (>0.4 eV) and a strong spin–orbit coupling (SOC), which facilitates ISC from singlet to triplet and reduces the nonradiative deactivation of triplet excitons.^8–12,40–46^ In short, the energy gap of excited states is closely related to these materials. Hence, by adjusting their energy levels, it is possible to obtain anti-Kasha dual emission and URTP materials from MR-TADF molecules.

Carbonyl-bridged triarylamines including 9*H*-quinolino[3,2,1-*kl*]phenothiazin-9-one (QPO) (Scheme 1), 9*H*-quinolino[3,2,1-*kl*]phenoxazin-9-one (QPXO), and 9,9-dimethylquinolino[3,2,1-*de*]acridin-5(9*H*)-one as typical MR-TADF materials exhibited excellent fluorescence performance.^47,48^ Theoretical calculations have become an effective means of assisting in developing luminous materials.^49–54^ Fluoroboron compounds are considered an excellent class of luminescent materials. Some sporadic literature has explored their anti-Kasha emission or phosphorescence characteristics.^8,17^ Transforming molecules into fluoroboron structures is an efficient method of tuning the molecular energy levels. We designed anti-Kasha dual emission fluoroboron compound 5a and URTP molecule 6b based on QPO as the core skeleton using density functional theory (DFT) calculations (Scheme 1a, Section XI, ESI†). The theoretical calculations reveal that they have relatively large energy gap Δ*E* (S_2_ → S_1,_5a: 0.81 eV; S_1_ → T_1,_6b: 0.51 eV) values. The large energy gap Δ*E*_S1T1_ (0.51 eV) indicates no RISC occurred. The relatively large SOC matrix elements ((S_1_, T_2_) = 0.87 cm^−1^, (S_0_, T_1_) = 1.63 cm^−1^) imply that effective ISC channels exist in 6b and triplet excitons can decay directly as phosphorescence (Scheme 1a, Section XI, ESI†).

Subsequently, we analyzed the synthetic routes of compound 5a and URTP molecule 6b. We first need to synthesize intermediates 8-methoxy-9*H*-quinolino[3,2,1-*kl*]phenothiazin-9-one (3a) and 10-methoxy-9*H*-quinolino[3,2,1-*kl*]phenothiazin-9-one (4a), which are QPO derivatives substituted with methoxy groups. Current methods of synthesizing QPO mainly involve the classical Ullmann coupling reaction, hydrolysis, and subsequent Friedel–Crafts acylation.^47,48,55^ However, this protocol usually has several disadvantages, such as cumbersome multi-step synthesis, being time-consuming, high temperature, and low yield, which makes it difficult to obtain the target compounds. Our group has recently developed copper-mediated C–H cyclization of 2-bromobenzaldehyde and 10*H*-phenothiazine to synthesize QPO and its derivatives, but unfortunately, this method is not compatible with 2-methoxy-10*H*-phenothiazine.^56^ Therefore, we must still develop an efficient method to synthesize 8-methoxy-9*H*-quinolino[3,2,1-*kl*]phenothiazin-9-one (3a) and 10-methoxy-9*H*-quinolino[3,2,1-*kl*]phenothiazin-9-one (4a).

## Results and discussion

### Condition optimization & substrate scope & luminescent testing

The cyclization reaction has become a promising strategy for constructing organic luminescent materials, which are simple, unique, and atomically efficient.^57–61^ Here, we deliberately designed a cyclization protocol based on a one-pot two-step using 2-methoxy-10*H*-phenothiazine and 2-bromobenzoic acid as substrates for the effective construction of 3a. After screening several parameters (see ESI, Table S1†), we got a yield of 85% under the standard reaction conditions (1a (0.1 mmol), 2a (2.0 equiv.), Cu_2_O (1.0 equiv.), NaO^*t*^Bu (2.0 equiv.), DMF (0.1 M), 120 °C, 24 h. Then the mixture was cooled to ambient temperature, followed by adding trifluoromethanesulfonic anhydride (TFAA, 0.05 M), and the reaction mixture was stirred at 80 °C for 6 h).

**Scheme 2:**
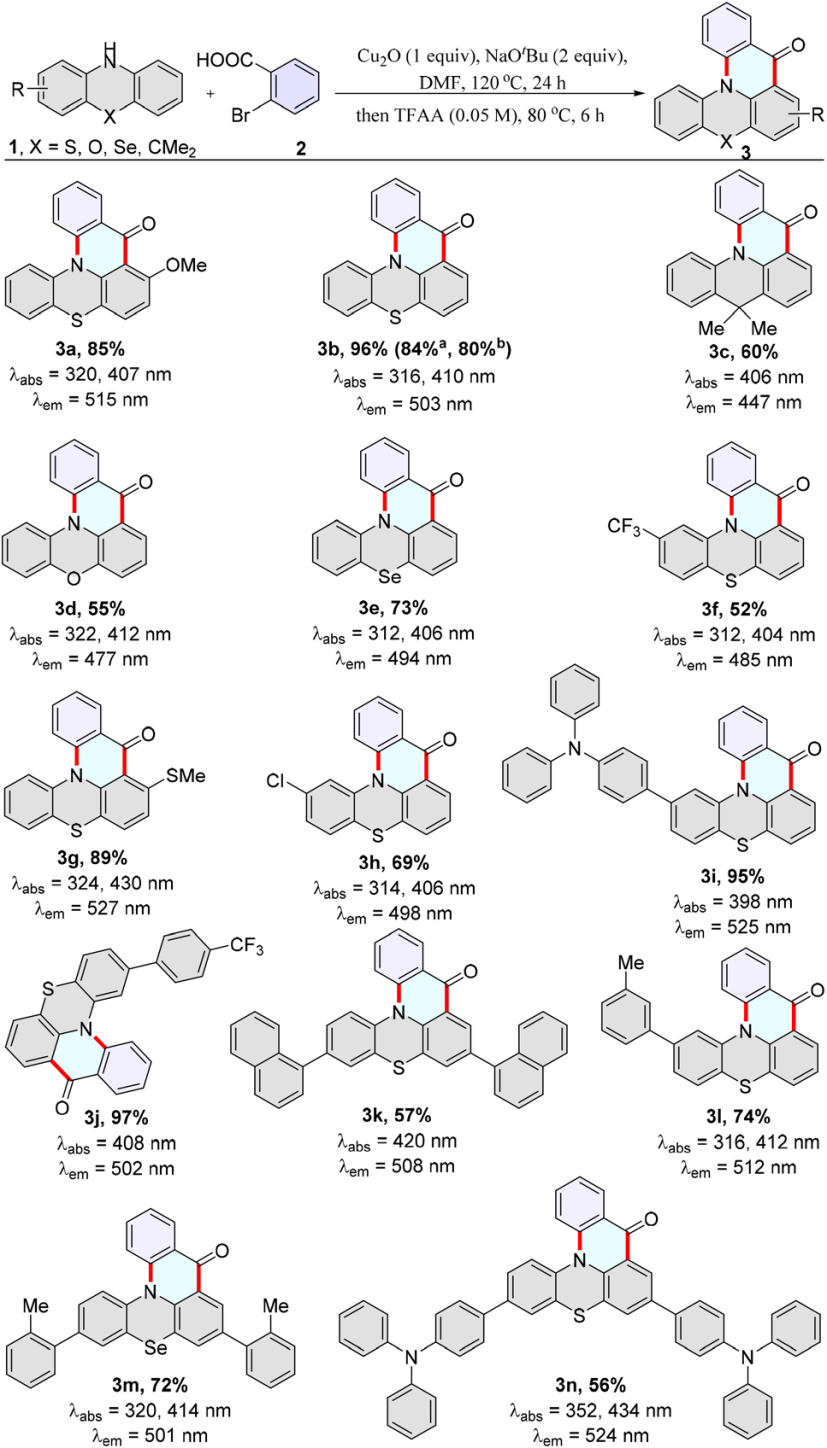
The scope of phenothiazines. Reaction conditions: 1 (0.1 mmol), 2 (2.0 equiv.), Cu_2_O (1.0 equiv.), NaO^*t*^Bu (2.0 equiv.), DMF (0.1 M), 120 °C, 24 h. Then the mixture was cooled to ambient temperature, followed by adding trifluoroacetic anhydride (TFAA) (0.05 M), 80 °C for 6 h. [a] 2-iodobenzoic acid, [b] and 2-chlorobenzoic acid as substrates. Absorption and emission maxima were measured in toluene (50 μM). Absolute quantum yield was determined with a calibrated integrating sphere system. *λ*_ex_ = 370 nm, PMT voltage = 700 V.

**Scheme 3:**
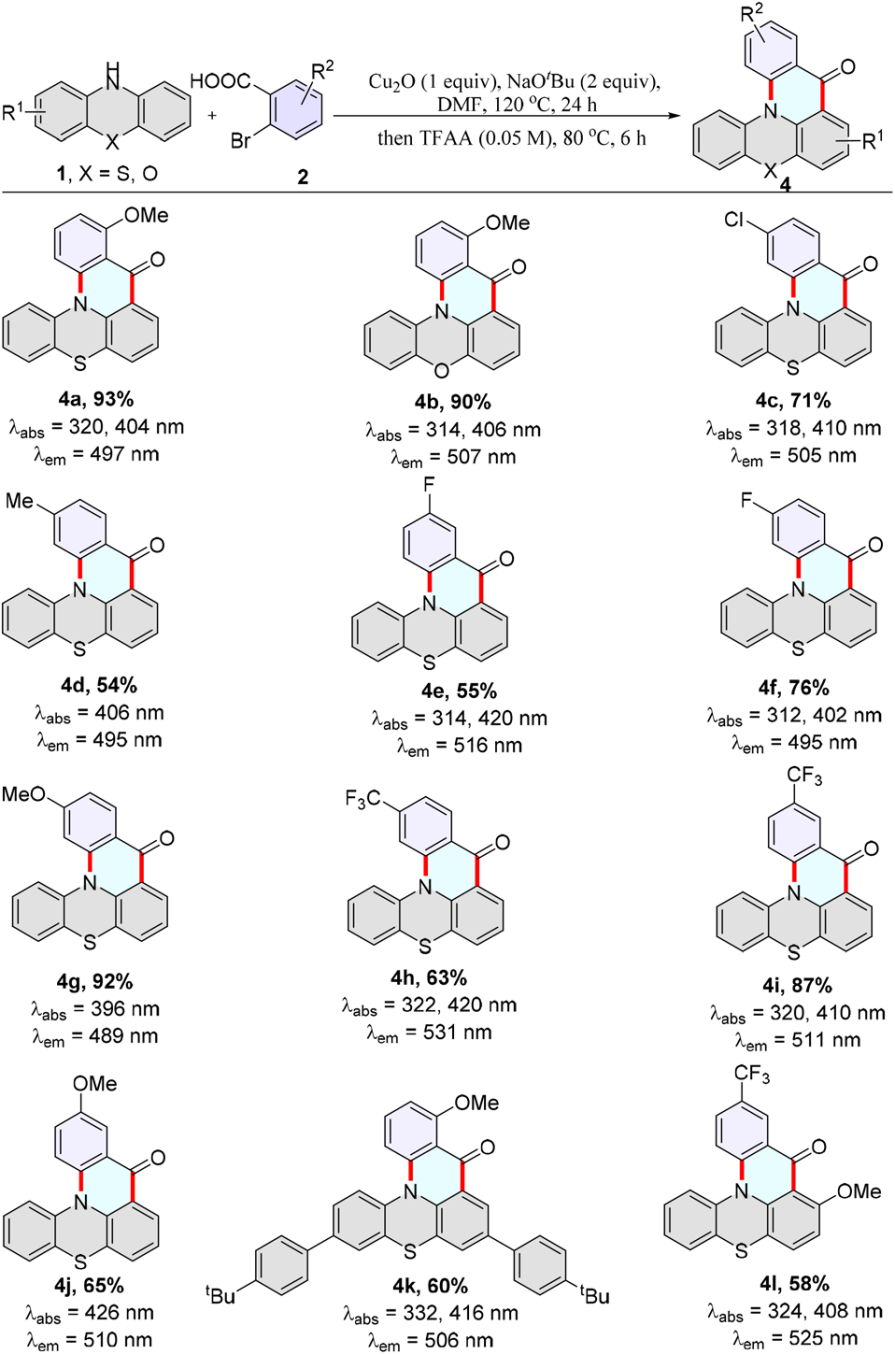
The scope of 2-bromo(hetero)aryl aldehydes. Reaction conditions: 1 (0.1 mmol), 2 (2.0 equiv.), Cu_2_O (1.0 equiv.), NaO^*t*^Bu (2.0 equiv.), DMF (0.1 M), 120 °C, 24 h. Then the mixture was cooled to ambient temperature, followed by adding TFAA (0.05 M), 80 °C for 6 h. Absorption and emission maxima were measured in toluene (50 μM). *λ*_ex_ = 370 nm, PMT voltage = 700 V.

Considering that this protocol is a novel approach for synthesizing MR-TADF materials QPO and its derivatives, it enables the synthesis of compounds that cannot be obtained by other methods. The substrate scope deserves further investigation. Subsequently, a variety of phenothiazines, phenoselenazine, 9,9-dimethyl-9,10-dihydroacridine, and phenoxazine were used to react with 2-bromobenzoic acids, providing the desired products 3a–3n in good to excellent yields under the optimized conditions (Scheme 2). 2-Bromobenzoic acids with electron-deficient and electron-rich groups on the aryl ring successfully cyclized with phenothiazine to yield the desired products (4a–4l) (Scheme 3). Moreover, 2-iodobenzoic acid and 2-chlorobenzoic acid could cyclize with phenothiazine to yield the desired product in good yields (3b). A variety of functional groups, such as fluorides, chlorides, trifluoromethyl, methoxy, methylthio, and triphenylamine, were well tolerated (3a, 3f, 3g, 3h, 3i, 3j, 3n, 4a–4c, 4e–4l), and thus offered an opportunity for further synthetic transformations.

In addition, these products (3a–4l) exhibit bright fluorescent emission, and the max emission wavelengths are located in the range of blue to yellow (447–531 nm) in degassed toluene (1 × 10^−5^ M). In particular, MR-TADF materials 3b, 3c, and 3d display remarkably high quantum yields of 57%, 85%, and 98%. Moreover 3b and 4a possess microsecond-scale delayed long-lifetime components (Table S2, Fig. S25 and S26†), indicating their TADF characteristics.

Furthermore, a tentative mechanism pathway is proposed (see ESI, Fig. S1†). First, intermediate I generates *via* the reaction of Cu^I^ with 1a under NaO^*t*^Bu conditions. Next, the oxidative addition of intermediate I with 2a gives the Cu^III^ species II, which undergoes reductive elimination to produce intermediate III and release the Cu^I^ species. Finally, intermediate III undergoes intramolecular cyclization under TFAA conditions to give the product 3a.

### Anti-Kasha dual-emission materials

**Scheme 4:**
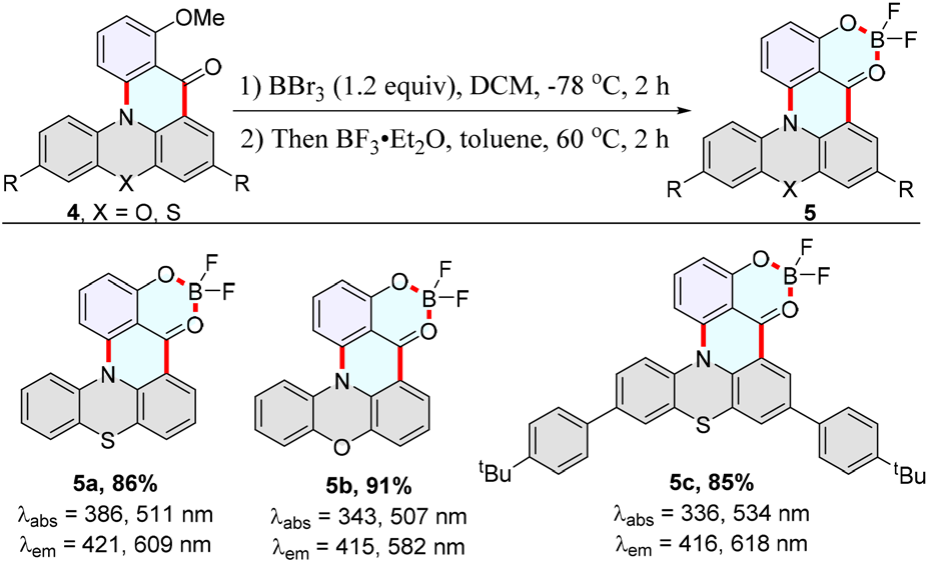
Synthesis of fluoroboron compounds 5a–5c. Step 1: 4 (0.1 mmol), BBr_3_ (1.2 equiv.), DCM (0.1 M), −78 °C, 2 h. Step 2: BF_3_·Et_2_O (3.5 equiv.), toluene (0.1 M), 60 °C, 2 h. Absorption and emission maxima were measured in CH_2_Cl_2_ (5a: 50 μM, 5b: 0.625 μM, 5c: 50 μM). *λ*_ex_ = 370 nm, PMT voltage = 700 V.

**Fig. 1:**
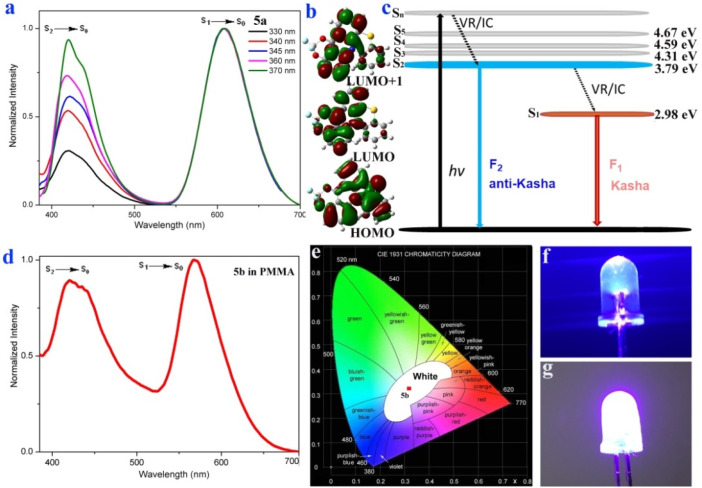
(a) Excitation-wavelength-dependent fluorescence spectra of 5a (50 μM in CH_2_Cl_2_). (b) Molecular orbitals of the S_0_, S_1_ or S_2_ states of 5a. (c) Jablonski diagram illustrating the anti-Kasha dual-emission mechanism of 5a. (d) The emission spectrum of 5b in the PMMA film (0.0125 wt%). (e) Emission color coordinates of 5b in the PMMA film (0.0125 wt%), CIE_1931_ (0.32, 0.32). (f) Luminescence image of a commercially available UV lamp (365 nm). (g) Luminescence image of UV LED light coated with the 5b PMMA film (0.0125 wt%) (turning on).

Subsequently, we successfully synthesized fluoroboron compounds 5a–5c based on cyclization products 4a, 4b, and 4k (Scheme 4). As expected, fluoroboron compounds 5a–5c exhibit apparent dual-emission behavior with a relatively short blue emission and a relatively long yellow emission wavelength (Scheme 4 and Fig. 1). Subsequently, the photoluminescence emissions of 5a in dichloromethane solution and 5b in the PMMA film were measured at 298 K and 77 K (Fig. S8 and S12†). The intensity of the longer yellow emission wavelength is unchanged, and the intensity of the shorter blue emission slightly decreases as the temperature is reduced from 298 K to 77 K (Fig. S8 and S12†). There is no significant change in the photoluminescence spectra on the whole. Furthermore, the photoluminescence-decay experiments of 5a in dichloromethane solution displayed a nanosecond-level lifetime in the dual-emission (Table S2 and Fig. S27†). The time-resolved photoluminescence measurements of 5b in the PMMA film at 298 K and 77 K demonstrate that both the short blue emission and the relatively long yellow emission have excited-state lifetimes on the order of nanoseconds (Table S2, Fig. S28–S29†), which ruled out the delayed fluorescence or phosphorescence emission. Furthermore, the anti-Kasha dual-emission characters of 5a were confirmed by the excitation-wavelength-dependent fluorescence experiments, emission-wavelength-dependent excitation experiments, and DFT calculations (Fig. 1, S10, Section XI, ESI†).

The DFT calculations reveal a longer wavelength (609 nm) from low-lying singlet state emission (S_1_ → S_0_), and a shorter wavelength (421 nm) from high-lying singlet state emission (S_2_ → S_0_, Fig. 1c, S34, Section XI, ESI†). The relatively large energy gap value (0.81 eV) could lead to a comparatively slow internal conversion (IC), and thus fluorescence emission could compete with IC (Fig. 1c). In the emission-wavelength-dependent excitation experiments, the intensity of the longer excitation wavelength (512 nm) is unchanged, and the intensity of the shorter excitation wavelength peak (385 nm) gradually decreases as the emission wavelength increases (Fig. S10†). In the excitation-wavelength-dependent emission experiments, lower energy excitations enhance intensity at shorter wavelengths (Fig. 1). These results implied that the dual emission was from different excited states and 5a has anti-Kasha properties.

Fluoroboron compound 5b exhibits white light emission with a fluorescence quantum yield of 25.8% and Commission Internationale de l'Eclairage (CIE) coordinates (0.35, 0.29) in solution. Furthermore, it displayed a distinct anti-Kasha dual-emission character with a relatively short blue emission (428 nm) and a relatively long orange emission (569 nm) in the PMMA film (0.0125 wt%) (Fig. 1d). The film exhibits white light emission and CIE coordinates of (0.32, 0.32), which are very close to those of pure white light (CIE: 0.33, 0.33) (Fig. 1e). The film was further coated on a commercially available ultraviolet (UV) light-emitting diode (LED) to obtain a low-cost and organic white LED (Fig. 1f and g).

### Room-temperature ultralong organic phosphorescence materials

**Scheme 5:**
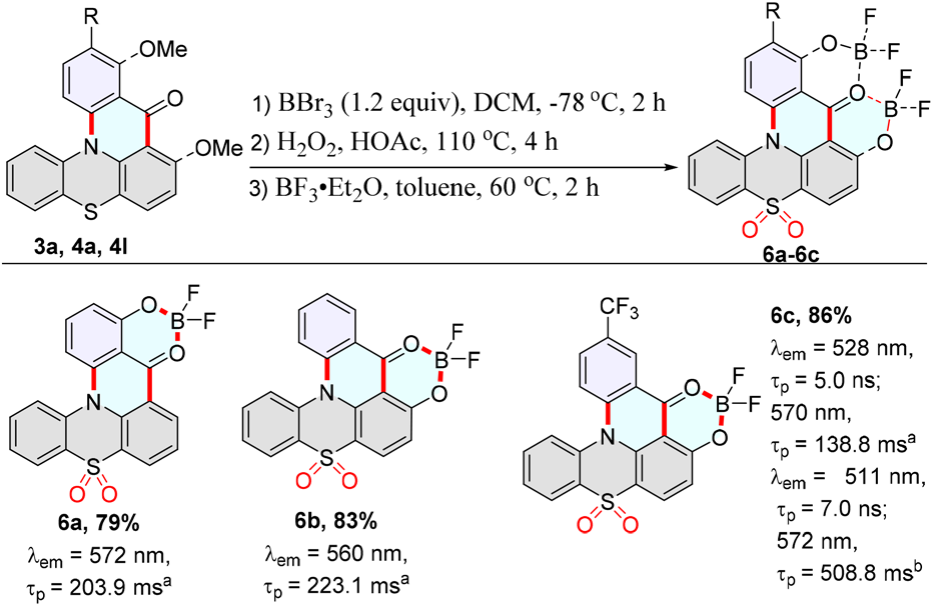
Synthesis of the room-temperature phosphorescence molecules 6a–6c. Step 1: 3a, 4a, 4l (0.1 mmol), BBr_3_ (1.2 equiv.), DCM (0.1 M), −78 °C, 2 h. Step 2: H_2_O_2_ 30% (2.5 equiv.), HOAc (0.1 M), 110 °C, 4 h. Step 3: BF_3_·Et_2_O (3.5 equiv.), toluene (0.1 M), 60 °C, 2 h. [a] Emission maxima and lifetime were measured in the TPA (triphenylamine) film (1.0 wt%). [b] Emission maxima and lifetime of 6c@TPA in the PMMA film (1.0 wt%). Emission maxima were measured in the TPA film (1.0 wt%). *λ*_ex_ = 370 nm, PMT voltage = 700 V.

Room-temperature phosphorescence molecules 6a–6c were obtained in good yields using cyclization products 3a, 4a, and 4l as the raw substrates (Scheme 5). As expected, ultralong organic room temperature phosphorescence (UORTP) was observed when 6a–6c were doped into TPA (triphenylamine) (1.0 wt%), exhibiting approximate lifetimes of 203.9, 223.1, and 138.9 ms, respectively (Scheme 5, Fig. S30–S32 and Table S2, ESI†).

**Fig. 2:**
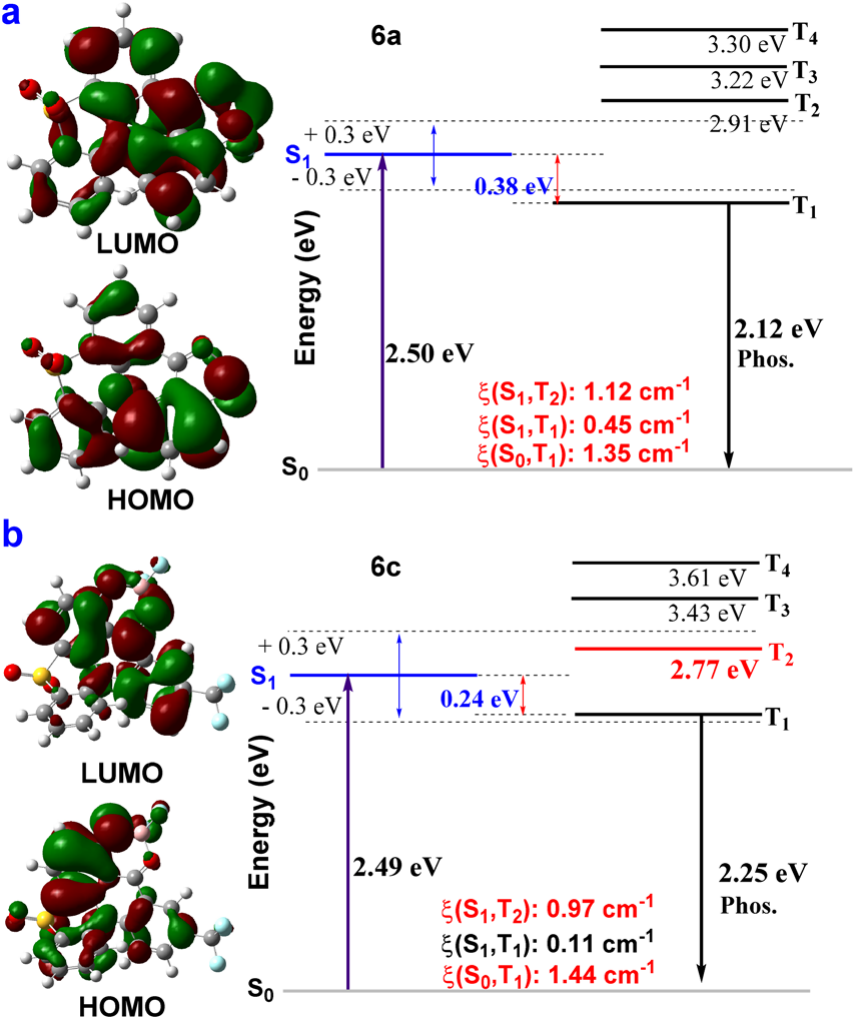
Molecular orbitals, energy levels, and SOC values between the singlet and triplet states of 6a (a) and 6c (b).

Subsequently, the energy levels and spin–orbit coupling (SOC) values between the singlet and triplet states of 6a–6c were calculated to further evaluate possible phosphorescence emission (Fig. 2, S35–S37, Section XI, ESI†). The theoretical S_1_ of 6a in dichloromethane solution was 2.50 eV (495 nm), which is very close to the experimental value of 2.44 eV (508 nm) in dichloromethane, and in accordance with the experimental value 2.50 eV (495 nm) in the PMMA film (Fig. 2, S15 and S35†). The theoretical T_1_ of 6a was 2.12 eV (579 nm), close to our experimental value of 2.19 eV (565 nm). This result could match the corresponding phosphorescence spectrum of 6a@TPA with a delay of 1 ms (Fig. 2, S15, S17 and S35†). The theoretical S_1_ of 6c in dichloromethane solution was 2.47 eV (502 nm), which is close to the experimental value of 2.43 eV (511 nm) in dichloromethane and 2.37 eV (523 nm) in the PMMA film (Fig. 2, Fig. S16 and S37†). The relatively large SOC matrix elements (6a: *ξ*(S_1_, T_1_) = 0.45 cm^−1^, *ξ*(S_0_, T_1_) = 1.35 cm^−1^; 6c: *ξ*(S_1_, T_2_) = 0.97 cm^−1^, *ξ*(S_0_, T_1_) = 1.44 cm^−1^) imply that effective ISC channels exist in 6a and 6c, and the triplet excitons could decay directly as phosphorescence (Fig. 2, S35 and S37, ESI†). Furthermore, a possible luminescent mechanism for the RTP behavior of the host–guest doped material based on 6a@TPA is shown in Fig. S38.†

Cyclic voltammetry (CV) was used to evaluate the electrochemical properties and energy levels of 4a, 5a and 6a. Fluoroboron compound 5a displays reversible oxidation waves, but 4a and 6a exhibit an irreversible reduction or oxidation wave (Fig. S41–S43, ESI†). They exhibit low-lying highest occupied molecular orbital (HOMO) levels. The HOMO and lowest unoccupied molecular orbital (LUMO) levels were further estimated from optical bandgaps and CV, which were −6.16/−3.38, −5.53/−3.47, and −5.60/−3.39 eV for 4a, 5a and 6a in CH_2_Cl_2_, respectively (Table S4, Fig. S41–S43, ESI†).

**Fig. 3:**
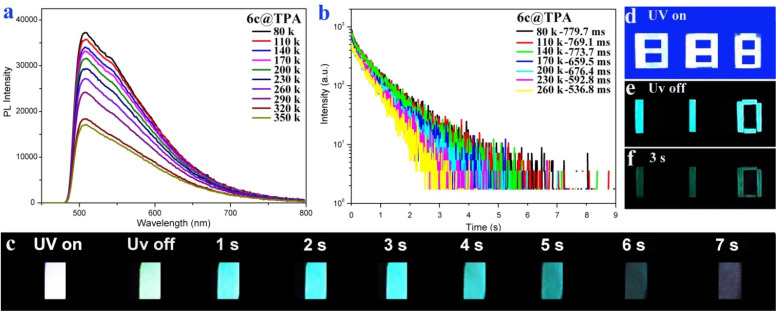
(a) Temperature-variable PL spectra of 6c@TPA in the PMMA film (1.0 wt%). (b) Temperature-dependent time-resolved phosphorescence decay curves of 6c@TPA in the PMMA film (1.0 wt%) (*λ*_em_ = 572 nm). (c) Photographs of the long-lived luminescence from 6c@TPA in the PMMA film (1.0 wt%) taken before and after turning off UV-365 nm excitation. (d) The digit “888” was prepared using both 6c@TPA and 3k in PMMA films (1.0 wt%). (e and f) Cyan afterglow image of “110” (UV off, and after turning off UV for 3 s) (*λ*_ex_ = 365 nm).

Subsequently, the doped system of 6c@TPA (1.0 wt%) was further made into a solid film in poly(methyl methacrylate) (PMMA) (1.0 wt%). Marvelously, the corresponding film showed a fluorescence emission at 511 nm with a lifetime of 7.0 ns, photo-activated UORTP at 572 nm with a lifetime of 508.8 ms and a photoluminescence quantum yield of 9.1% (Fig. S33, ESI†). Furthermore, the theoretical T_1_ of 6c was 2.25 eV (551 nm), which could match the corresponding phosphorescence spectrum of 6c@TPA in PMMA with a delay of 1 ms (Fig. 2, S20 and S37†). This result suggests that the ultralong phosphorescence bands in the doped system of 6c@TPA in the PMMA film (1.0 wt%) could be from 6c. Moreover, a possible luminescent mechanism for the RTP behavior of the host–guest doped system based on 6c@TPA in the PMMA film is shown in Fig. S39.† In addition, in the temperature-variable PL spectra, the emission intensity at 572 nm gradually enhanced when the temperature decreased from 350 to 80 K (Fig. 3a). The intensity of lifetimes increased from 536.8 ms to 779.7 ms when the temperature was decreased from 260 K to 80 K (Fig. 3b). Impressively, the corresponding film possessed a cyan afterglow for up to seven seconds (Fig. 3c) after the UV light was turned off. These results indicate the nature of the ultralong phosphorescence of this film. We further explored its application in encryption based on the excellent phosphorescence properties of the doped film. The digit “888” was prepared using both the doped system of 6c@TPA (1.0 wt%) and 3k; upon exposure to a 365 nm UV lamp, the pattern displayed “888”; when the UV lamp was turned off, a cyan afterglow image of “110” (alarm signal) was observed (Fig. 3d–f).

## Conclusions

In conclusion, we successfully obtained anti-Kasha dual-emission and room-temperature phosphorescence materials by energetic tuning and structural modification of MR-TADF molecule QPO. The copper-mediated cyclization of phenothiazines and 2-bromobenzoic acids was further developed to build a library of structurally diverse carbonyl-bridged triarylamines for the first time. This approach based on a one-pot two-step shows a broad substrate scope and provides straightforward access to QPO and its derivatives, which opens up a new route for the rapid screening of high-performance MR-TADF materials. Moreover, the novel anti-Kasha dual-emission fluoroboron compound displayed obvious white light emission with CIE coordinates of (0.32, 0.32) in the solid film, which are close to those of pure white light (CIE: 0.33, 0.33), and was further fabricated into low-cost and robust organic white LEDs. Furthermore, the novel room-temperature phosphorescence materials based on phenothiazines show an ultralong lifetime and afterglow, which could be used in encryption. This work represents the first example of tuning the energetics of carbonyl-bridged triarylamines to rapidly design and synthesize anti-Kasha dual-emission and room-temperature phosphorescence materials.

## Data availability

All experimental data associated with this work are provided in the ESI.†

## Author contributions

L. W., S. L., L. Y. performed the experiments and analyzed the data. B. L. designed and directed the project and wrote the manuscript. All authors contributed to discussions.

## Conflicts of interest

There are no conflicts to declare.

## Supplementary Material

SC-OLF-D5SC02096D-s001
