# Supplementary material for: Tuning the energetics of carbonyl-bridged triarylamines: from thermally activated delayed fluorescence to anti-Kasha dual-emission and room temperature phosphorescence materials
Source: Chem Sci. 2025 May 22;16(26):12051–7. doi: 10.1039/d5sc02096d (PMC12132550; doi:10.1039/d5sc02096d)
Supplement: SC-016-D5SC02096D-s001 [file SC-016-D5SC02096D-s001.pdf]

Supplementary Information (SI) for Chemical Science.
This journal is © The Royal Society of Chemistry 2025

Supporting Information

Tuning the energetics of carbonyl-bridged triarylamines: from thermally activated delayed fluorescence to anti-Kasha dual-emission and room temperature phosphorescence materials

Liqiu Wan, Sisi Ling, Lei Yang, and Bijin Li*

Engineering Research Center of the Ministry of Education for the Development of Targeted Anti-tumors and Anti-pathogens New Drugs & Chongqing Key Laboratory of Natural Product Synthesis and Drug Research, School of Pharmaceutical Sciences, Chongqing University, Chongqing 401331, P. R. China.

Table of Contents

I. General remarks S3

II. Synthesis of substrates **2**...S4

III. Optimization of the copper-mediated C−H cyclization of 2-methoxy-10*H*-phenothiazine S4

IV. General procedure for synthesizing the carbonyl-bridged triphenylamine compounds S6

V. General procedure for synthesizing the anti-Kasha compounds.............................S6

VI. General procedure for synthesizing the phosphorescent compounds S7

VII. Plausible catalytic cycle...S9

VIII. The photophysical properties of **3a-6c**...S10

IX. The excited state lifetimes of **3b**, **4a**, **5a**, **5b**, **6a-6c** in solution and films S21

X. Photoluminescence quantum yields S29

XI. Calculation...S30

XII. Electrochemistry properties of **4a**, **5a** and **6a**. S44

XIII. Experimental data for the desired products...S47

XIV. References. S69

XV. Copies of ^{1}H and ^{13}C NMR spectra. S70

I. General remarks

All NMR spectra were obtained on an Agilent 400 MR DD 2 (400 MHz) spectrometer. ^{1}H NMR (400 MHz) and ^{13}C NMR (101 MHz) chemical shifts were tested relative to $SiMe_4$, $CDCl_3$ using the chemical shift of residual solvent peaks as reference ($SiMe_4$: δ (ppm) 0 ppm for ^{1}H NMR and ^{13}C NMR; $CDCl_3$: 7.26 ppm for ^{1}H NMR and 77.16 ppm for ^{13}C NMR). High-resolution mass spectra (HR-MS) were measured using Agilent 6546 Q-TOF LC/MS system with Electrospray Ionization (ESI). Fluorescence spectra were recorded on F-7000 or Edinburgh Instruments FLS 1000 fluorescence spectrometer. The CIE_{1931} chromaticity coordinates were calculated using a Color Coordinate.exe program. UV−vis spectra were collected on the Agilent Cary 60 spectrometer. The determination of quantum yields for these compounds is the absolute quantum yields. The absolute quantum yields were taken using Edinburgh Instruments FLS 1000 fluorescence spectrometer with a calibrated integrating sphere system. The excited-state lifetimes were recorded using Edinburgh Instruments FLS 1000.

Unless otherwise noted, all reagents were obtained from commercial sources and used directly. Thin-layer chromatography is performed on a plate bonded with sodium carboxymethyl cellulose (silica gel GF 254) and visualized by fluorescence quenching under UV light. Column chromatography was performed through silica gel (200-300 mesh) using a proper solvent system. All syntheses and manipulations were carried out under an air atmosphere using standard Schlenk.

II. Synthesis of substrates 2

General procedure for the synthesis of substrates 2:

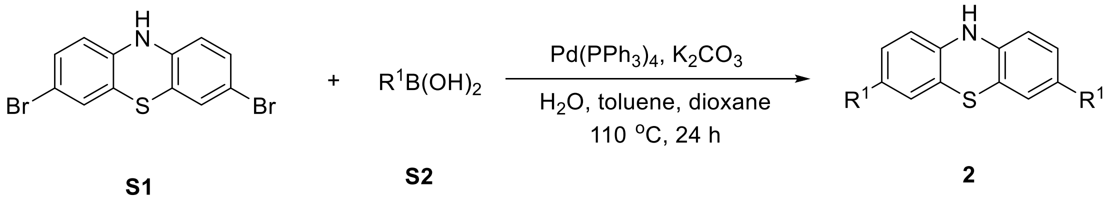

Scheme S1. Synthesis of substrates **2**.

A Schlenk test tube with a magnetic stir bar was charged with **S1** (0.8 mmol, 1.0 equiv.), **S2** (2.4 mmol, 3.0 equiv.), $Pd(PPh_3)_4$ (2 mol%), K_2CO_3 (1.4 mmol, 1.75 equiv.), H_2O (0.2 mL, 11 mmol), toluene (4 mL), and dioxane (4 mL) under an N_2 atmosphere and the reaction mixture was allowed to stir at 110 ℃ for 24 h. The reaction mixture was cooled to ambient temperature, diluted with 10 mL of DCM, and evaporated under reduced pressure. Purification via column chromatography on silica gel (petroleum ether/ethyl acetate = 10:1, v/v) afforded the corresponding substrates **2**.

III. Optimization of the copper-mediated C-H cyclization of 2-methoxy-10*H*-phenothiazine

A Schlenk tube with a magnetic stir bar was charged with copper salt (0.1 mmol), base (0.2 mmol), 2-methoxy-10*H*-phenothiazine (**1a**, 0.1 mmol), 2-bromobenzoic acid (**2a**, 0.2 mmol), and solvent (1 mL), and then heated at the indicated temperature for the indicated time. Then, the reaction mixture was cooled to ambient temperature, 0.05 M trifluoroacetic anhydride (TFAA) was added, and the reaction mixture was

allowed to stir at 80 ℃ for 6 hours. After that, the reaction mixture was extracted by ethyl acetate (EA) for 3 times, and the combined organic layers were washed with water (15 mL × 3). The organic layers were combined, dried over Na_2SO_4, filtered, and concentrated under reduced pressure. The crude product was analyzed by ^1H NMR in $CDCl_3$. Yields are based on **1a**, determined by crude ^1H NMR using dibromomethane as the internal standard, and the residue was purified by flash column chromatography on silica gel to provide the desired product.

Table S1. Optimization of reaction conditions[a]

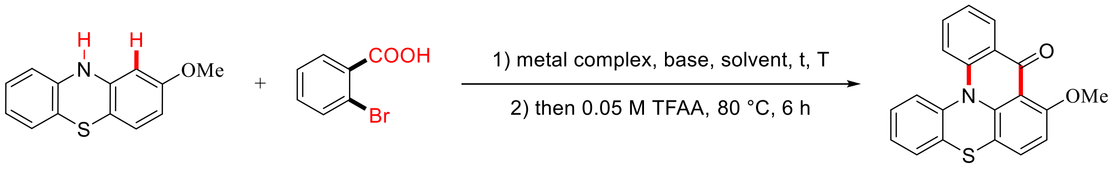

1a 2a 3a

Entry	Copper salt	Base	Solvent	T (°C)	Time (h)	Yield (%)[b]
1	Cu_2O	K_2CO_3	DMF	120	24	72%
2	CuI	K_2CO_3	DMF	120	24	32%
3	CuCl	K_2CO_3	DMF	120	24	43%
4	CuBr	K_2CO_3	DMF	120	24	37%
5	Cu_2O	NaOAc	DMF	120	24	52%
6	Cu_2O	Bu^tONa	DMF	120	24	85%[c]
7	Cu_2O	K_3PO_4	DMF	120	24	40%
8	Cu_2O	Bu^tONa	Dioxane	120	24	70%
9	Cu_2O	Bu^tONa	Toluene	120	24	47%
10	Cu_2O	Bu^tONa	MeCN	120	24	27%
11	Cu_2O	Bu^tONa	DMF	100	24	53%
12	Cu_2O	Bu^tONa	DMF	80	24	Trace
13	Cu_2O	Bu^tONa	DMF	120	18	64%
14	Cu_2O	Bu^tONa	DMF	120	12	41%
15	none	Bu^tONa	DMF	120	24	N.D.
16	Cu_2O	none	DMF	120	24	33%
17[d]	Cu_2O/TEMPO	Bu^tONa	DMF	120	24	82%

[a] **1a** (0.1 mmol, 1.0 equiv.), **2a** (0.2 mmol, 2.0 equiv.), copper salt (0.1 mmol, 1.0 equiv.), base (0.2 mmol, 2.0 equiv.), solvent (1 mL), 80-120 °C, 12-24 h. [b] Yield is

based on **1a**, determined by ^{1}H-NMR using dibromomethane as the internal standard. [c] Isolated yield. [d] TEMPO (1.0 equiv.). N.D.: No detected.

IV. General procedure for synthesizing the carbonyl-bridged triphenylamine compounds

A Schlenk tube with a magnetic stir bar was charged with **1** (0.1 mmol, 1.0 equiv.), **2** (0.2 mmol, 2.0 equiv.), Cu_2O (0.1 mmol, 1.0 equiv.), Bu^{t}ONa (0.2 mmol, 2.0 equiv.), and DMF (0.1 M) and the reaction mixture was allowed to stir at 120 ℃ for 24 h. Then, the reaction mixture was cooled to ambient temperature, 0.05 M trifluoroacetic anhydride was added, and the reaction mixture was allowed to stir at 80 ℃ for 6 hours. After that, the reaction mixture was extracted by ethyl acetate 3 times, and the combined organic layers were washed with water (15 mL × 3). The organic layers were combined, dried over Na_2SO_4, filtered, and concentrated under reduced pressure. The resulting residue was purified by flash column chromatography on silica gel (petroleum ether/ethyl acetate = 10:1, v/v) to provide the desired products **3-4**.

V. General procedure for synthesizing the anti-Kasha compounds

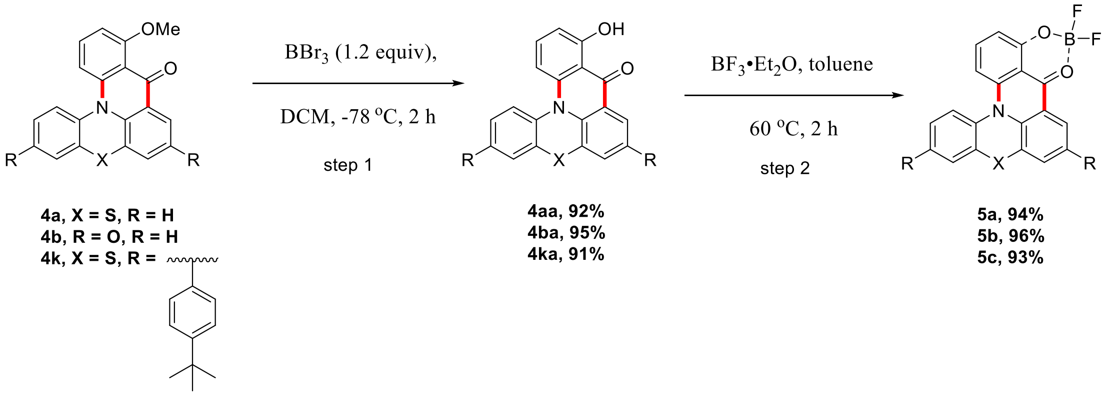

Scheme S2. Synthesis of fluoroboron compounds **5a-5c**.

Step 1: BBr_3 in CH_2Cl_2 (1 M, 0.2 mL, 0.2 mmol) was added to a solution of **4a**, **4b**, or **4k** (0.1 mmol) in dry CH_2Cl_2 (3 mL) at -78 ℃. The reaction mixture was stirred for 2 h at -78 ℃, and then raised to room temperature and stirred for 4 h. The mixture was then diluted with ice water (100 mL) and neutralized with sat. aq. $NaHCO_3$ (pH = 7). After that, the reaction mixture was extracted by CH_2Cl_2 3 times, the combined organic extracts were dried with Na_2SO_4, and the solvents evaporated at reduced pressure. The resulting residue was purified by flash column chromatography on silica gel (petroleum ether/ethyl acetate = 10:1, v/v) to provide the desired products **4aa**, **4ba**, **4ka**. Step 2: Toluene (1 mL) and $BF_3 \cdot Et_2O$ (3.5 equiv., 0.35 mmol) were added to **4aa**, **4ba**, or **4ka**, and then the reaction mixture was stirred at 60 ℃ for 2 hours. After cooling down to room temperature, the precipitate was collected by filtration and washed with toluene to obtain the desired products **5a**-**5c**.

VI. General procedure for synthesizing the phosphorescent compounds

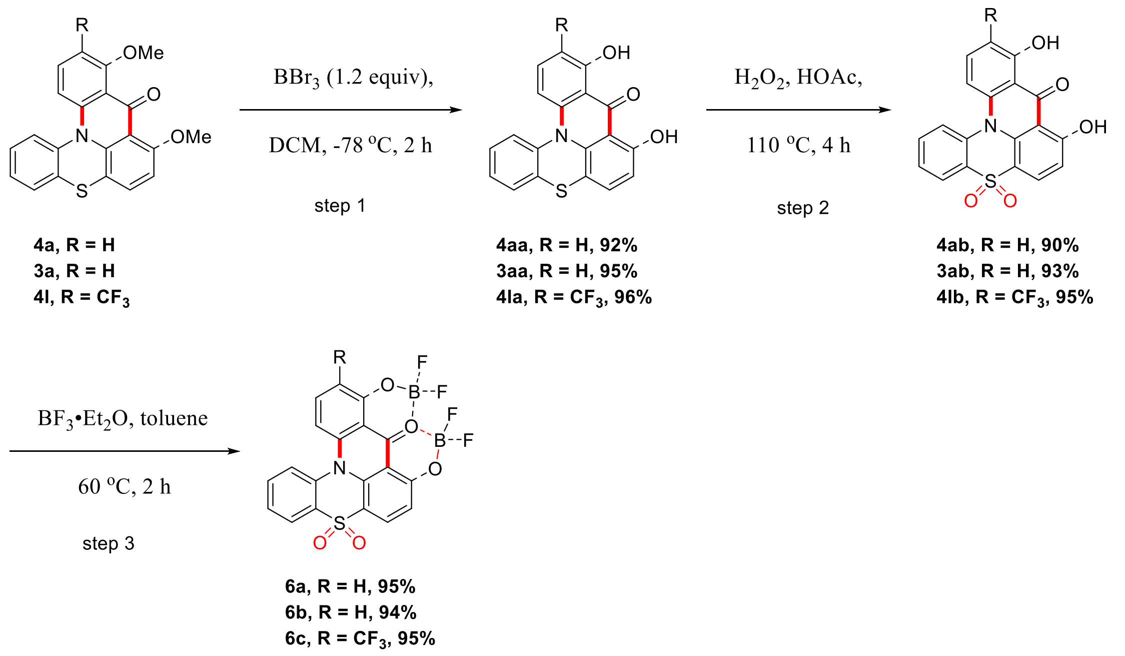

Scheme S3. Synthesis of the room-temperature phosphorescence molecules **6a-6c**.

Step 1: BBr_3 in CH_2Cl_2 (1 M, 0.2 mL, 0.2 mmol) was added to a solution of **4a**, **3a**, or **4l** (0.1 mmol) in dry CH_2Cl_2 (3 mL) at -78 ℃. The reaction mixture was stirred for 2 h at -78 ℃, and then raised to room temperature and stirred for 4 h. Then dilute the mixture with ice water (100 mL) and neutralize with sat. aq. $NaHCO_3$ (pH = 7). After that, the reaction mixture was extracted by CH_2Cl_2 3 times, the combined organic extracts were dried with Na_2SO_4, and the solvents evaporated at reduced pressure. The resulting residue was purified by flash column chromatography on silica gel (petroleum ether/ethyl acetate = 10:1, v/v) to provide the desired products **4aa**, **3aa**, **4la**. Step 2: A Schlenk tube with a magnetic stir bar was charged with **4aa**, **3aa**, or **4la** (0.1 mmol), H_2O_2 30% (0.25 mmol), HOAc (1 mL), and the reaction mixture was allowed to stir at 110 ℃ for 4 h. The reaction mixture was cooled to ambient temperature, and then the resulting mixture was extracted with CH_2Cl_2 (3 × 20 mL). The organic layers were combined, dried over Na_2SO_4, filtered, and concentrated

under reduced pressure. The resulting residue was purified by flash column chromatography on silica gel (petroleum ether/ethyl acetate = 5:1, v/v) to provide the desired products **4ab**, **3ab**, and **4lb**. Step 3: Toluene (1 mL) and $BF_3 \cdot Et_2O$ (3.5 equiv., 0.35 mmol) were added and the reaction mixture was allowed to stir at 60 ℃ for 2 hours. After cooling down to room temperature, the precipitate was collected by filtration and washed with toluene to obtain the desired products **6a-6c**.

VII. Plausible catalytic cycle

First, the intermediate **I** generates via Cu^I reacts with **1a** under the ^tBuONa condition. Next, the oxidative addition of intermediate **I** with **2a** gives the Cu^{III} species **II**, which undergoes reductive elimination to produce the intermediate **III** and release the Cu^I species. Finally, intermediate **III** undergoes intramolecular cyclization under TFAA conditions to give the product **3a**.

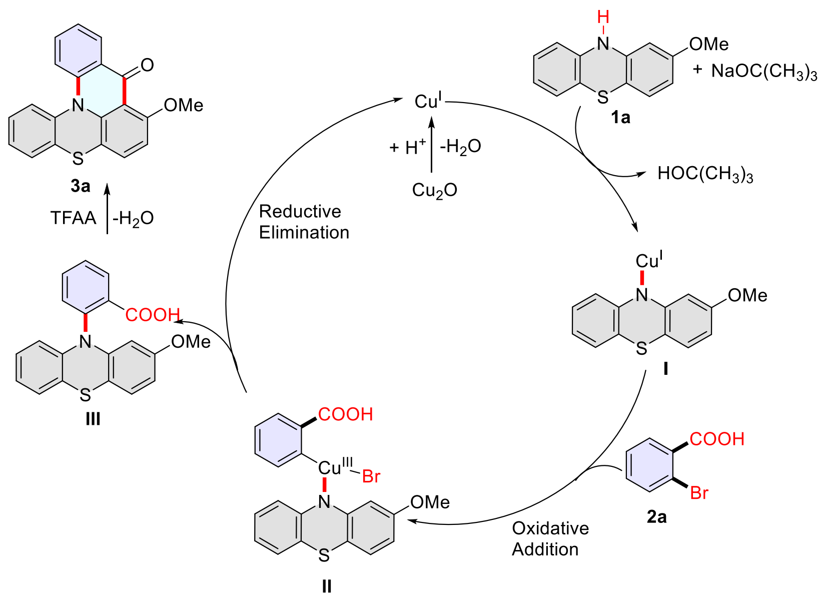

Figure S1. The tentative mechanism pathway.

VIII. The photophysical properties of 3a-6c

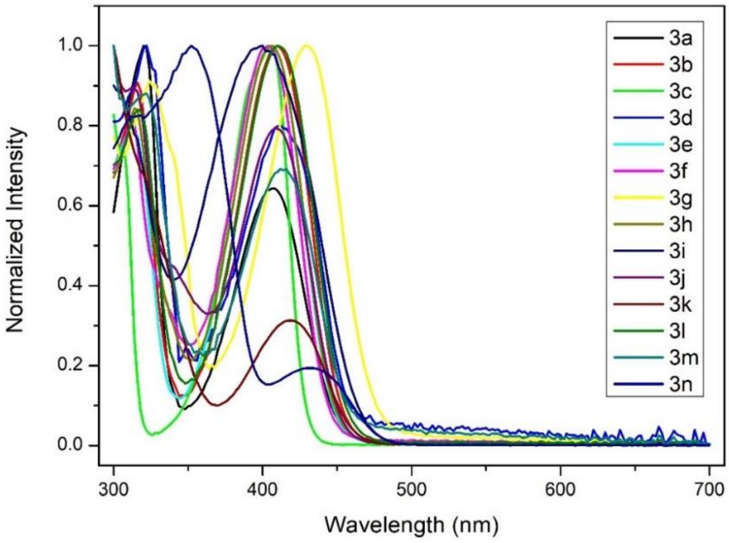

Figure S2. Normalized UV-Vis absorption spectra of **3a-3n** in toluene (5×10^{-5} M).

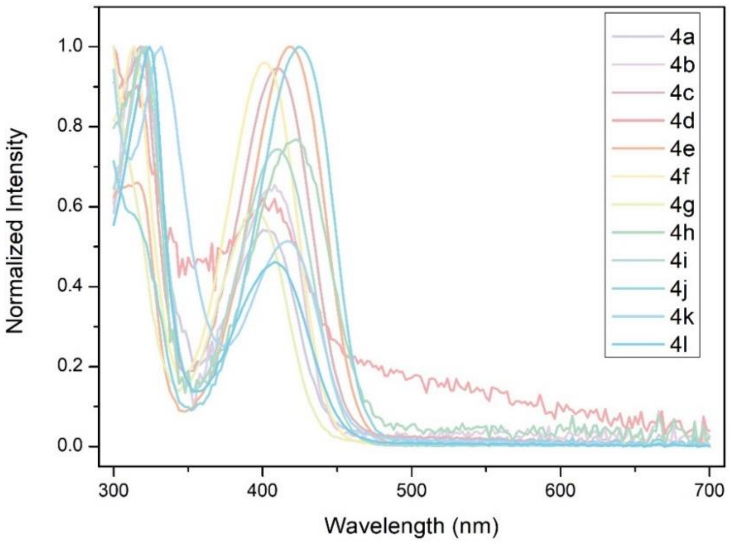

Figure S3. Normalized UV-Vis absorption spectra of **4a-4l** in toluene (5×10^{-5} M).

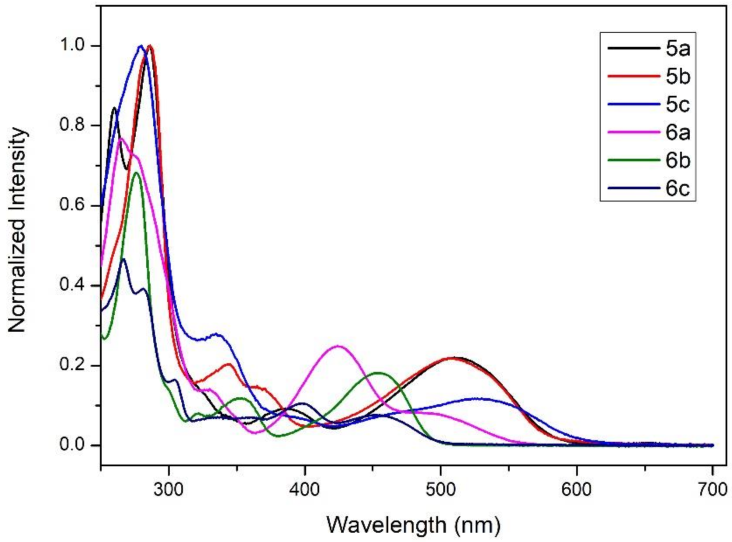

Figure S4. Normalized UV-Vis absorption spectra of **5a-6c** in CH_2Cl_2 (5×10^{-5} M).

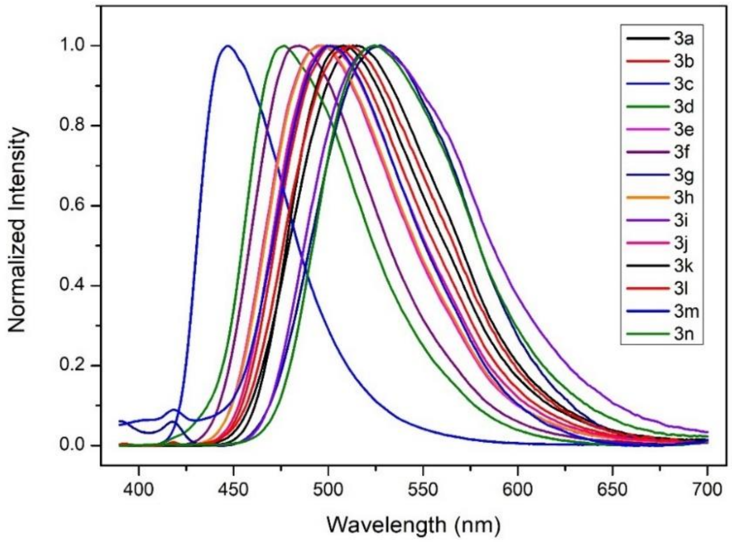

Figure S5. Normalized fluorescence emission spectra of **3a-3n** in toluene (5×10^{-5} M).

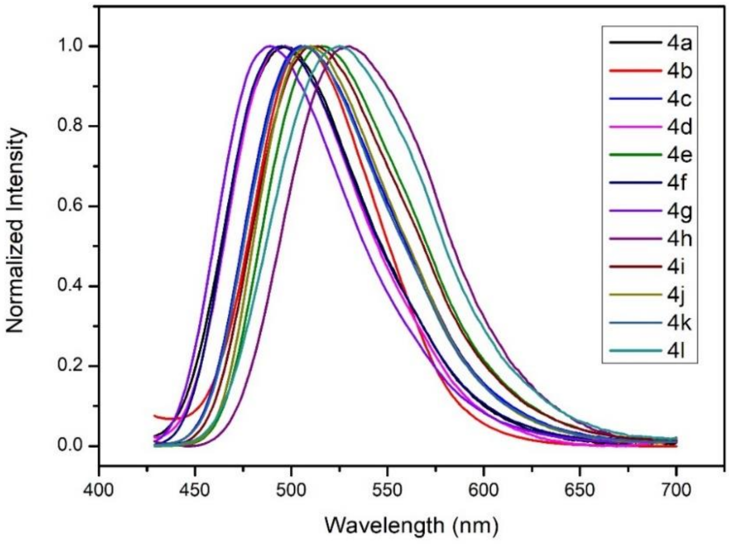

Figure S6. Normalized fluorescence emission spectra of **4a-4l** in toluene (5×10^{-5} M).

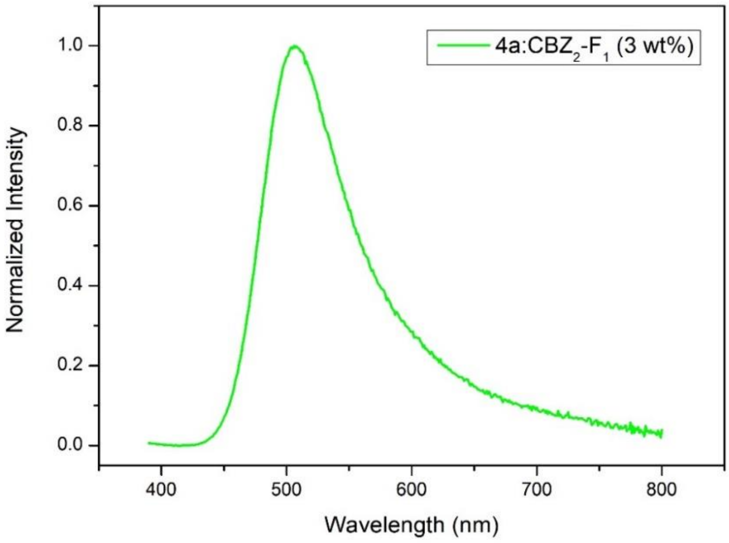

Figure S7. Normalized fluorescence emission spectra of **4a** in CBZ_2-F_1 film (3 wt%) (λ_{ex} = 370 nm).

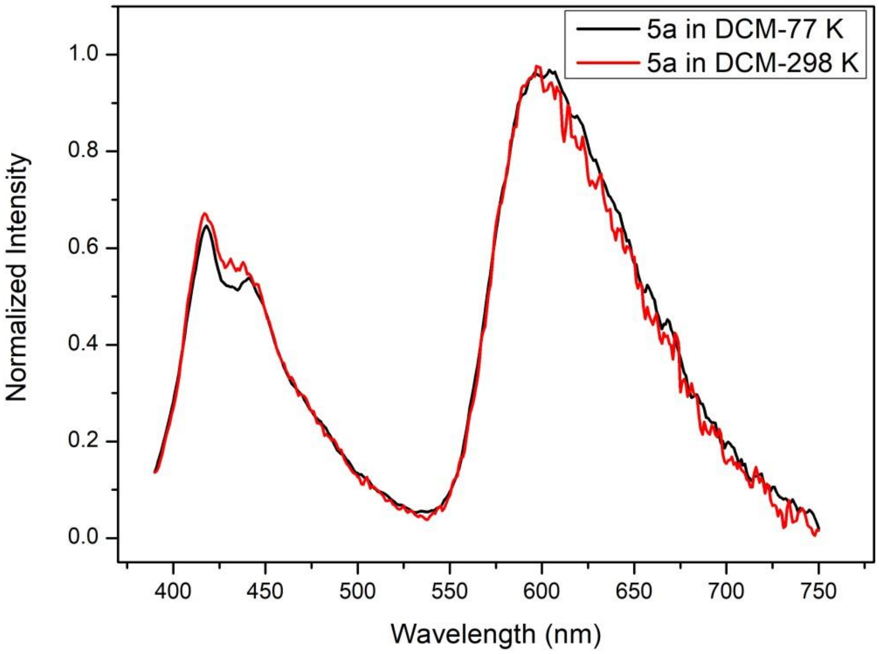

Figure S8. Normalized fluorescence emission spectra of **5a** in DCM at 77 K and 298 K (5 $\times 10^{-5}$ M).

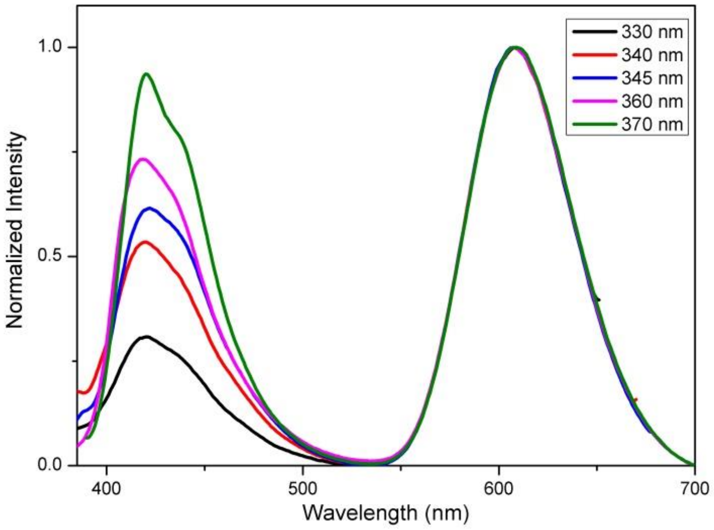

Figure S9. Excitation-wavelength-dependent fluorescence spectra of **5a** in CH_2Cl_2 (5 $\times 10^{-5}$ M).

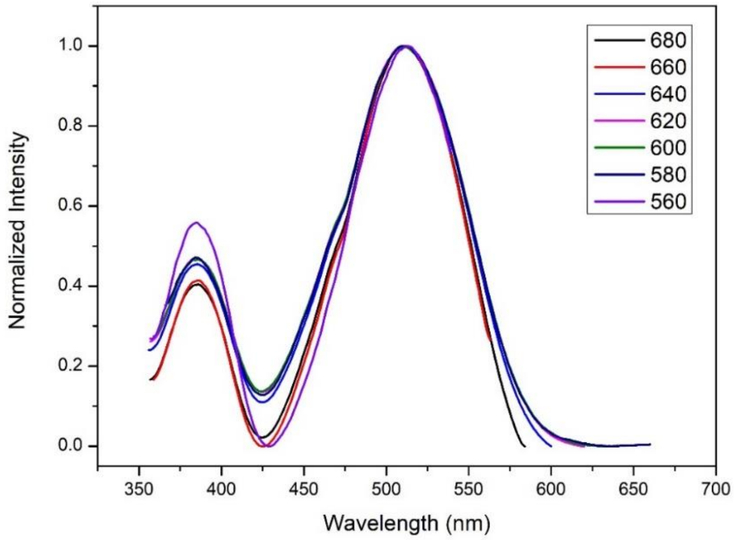

Figure S10. Emission wavelength-dependent excitation spectra of **5a** in CH_2Cl_2 (5 × 10^{-5} M).

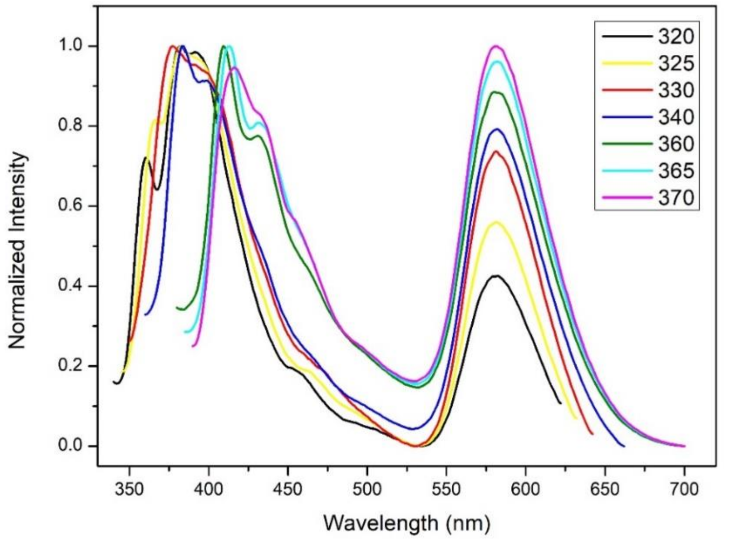

Figure S11. Excitation-wavelength-dependent fluorescence spectra of **5b** in CH_2Cl_2 (6.25 × 10^{-7} M).

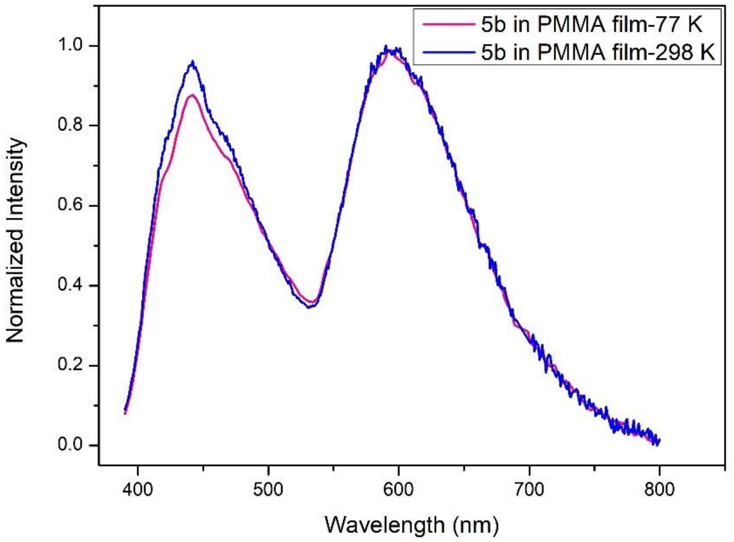

Figure S12. Normalized fluorescence emission spectra of **5b** in PMMA film at 77 K and 298 K (0.0125 wt%).

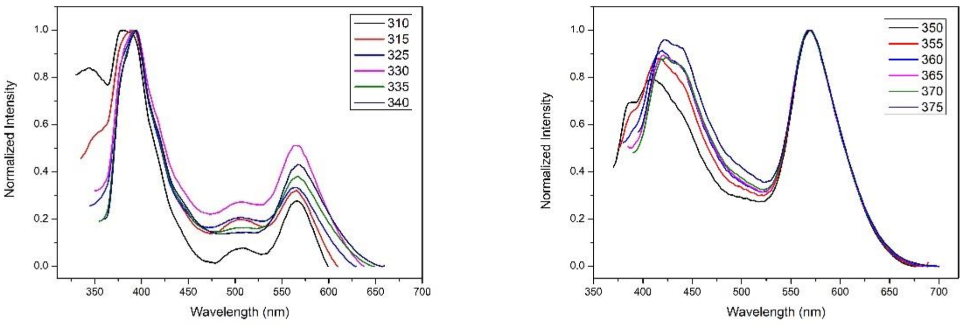

Figure S13. Excitation-wavelength-dependent fluorescence spectra of **5b** in PMMA film (0.0125 wt%).

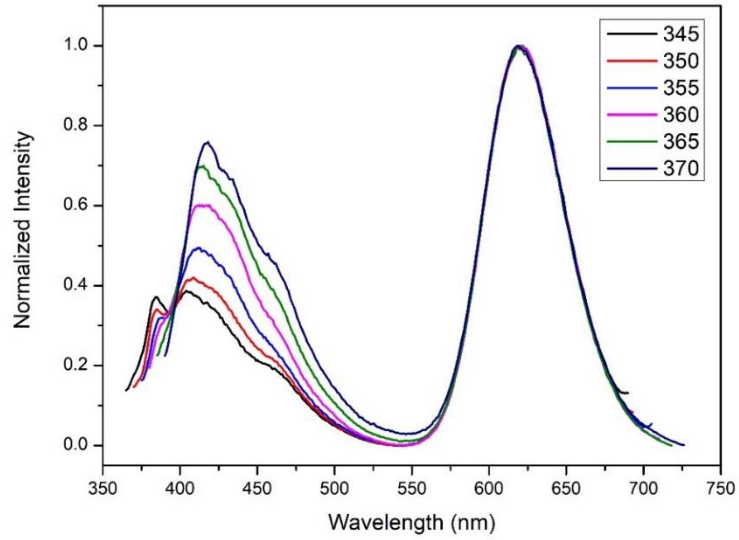

Figure S14. Excitation-wavelength-dependent fluorescence spectra of **5c** in CH_2Cl_2 (5×10^{-5} M).

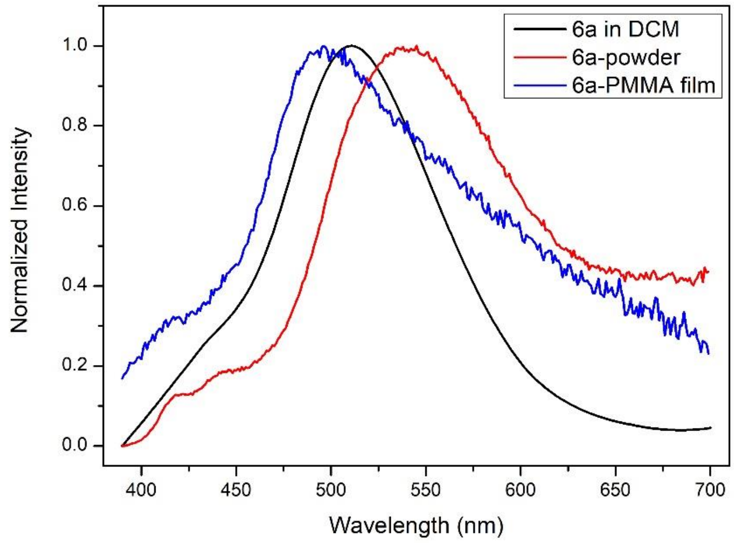

Figure S15. Normalized fluorescence emission spectra of compound **6a** in different states (solution: 5×10^{-5} M; PMMA film: 1 wt%).

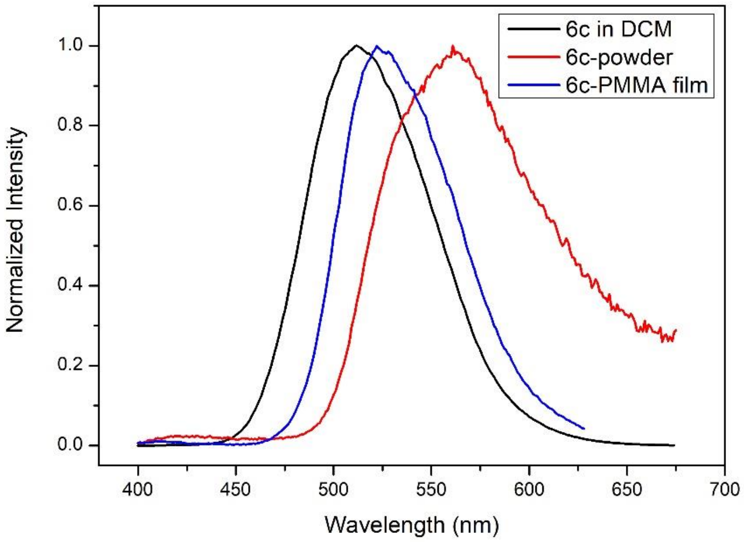

Figure S16. Normalized fluorescence emission spectra of compound **6c** in different states (solution: 5×10^{-5} M; PMMA film: 1 wt%).

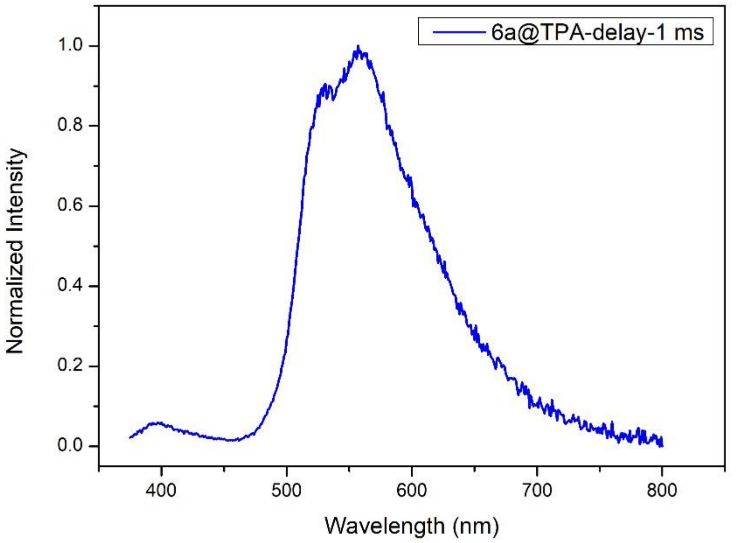

Figure S17. Normalized phosphorescence emission spectrum of **6a**@TPA (1 wt%) film with a delay of 1 ms (λ_{ex} = 370 nm).

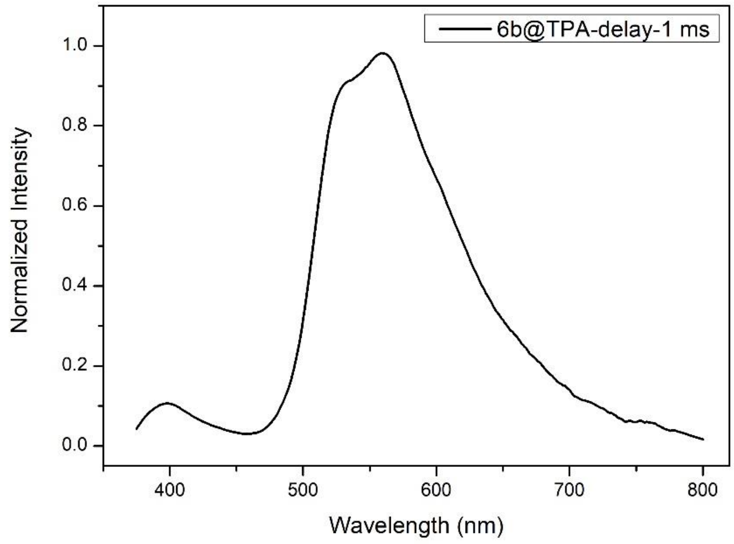

Figure S18. Normalized phosphorescence emission spectrum of **6b**@TPA (1 wt%) film with a delay of 1 ms (λ_{ex} = 370 nm).

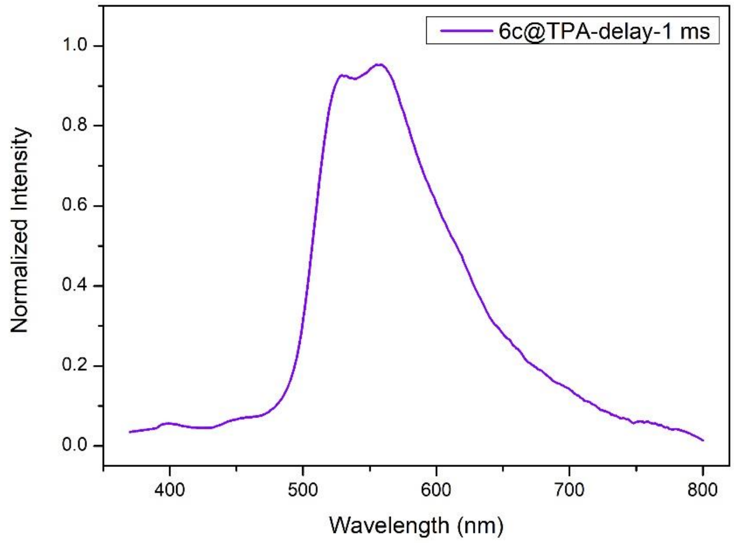

Figure S19. Normalized phosphorescence emission spectrum of **6c**@TPA (1 wt%) film with a delay of 1 ms (λ_{ex} = 370 nm).

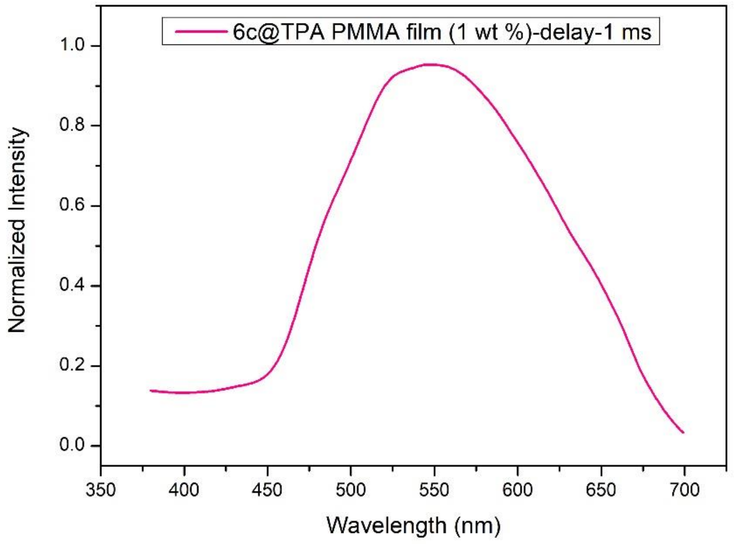

Figure S20. Normalized phosphorescence emission spectrum of **6c**@TPA in PMMA film (1.0 wt%) with a delay of 1 ms (λ_{ex} = 370 nm).

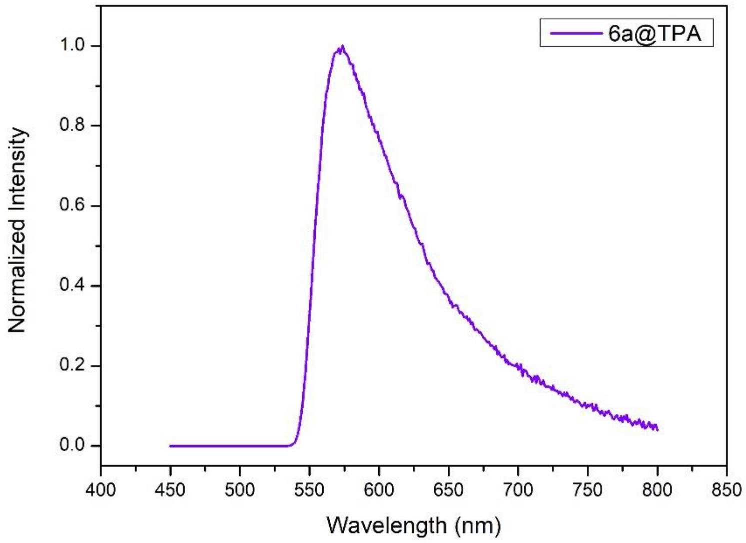

Figure S21. Normalized photoluminescence spectrum (298K) of **6a**@TPA (1 wt%) film (λ_{ex} = 370 nm).

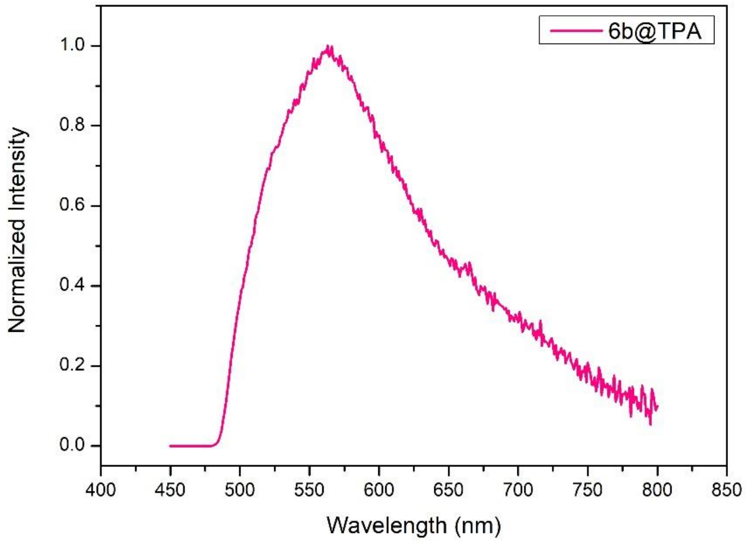

Figure S22. Normalized photoluminescence spectrum (298K) of **6b**@TPA (1 wt%) film (λ_{ex} = 370 nm).

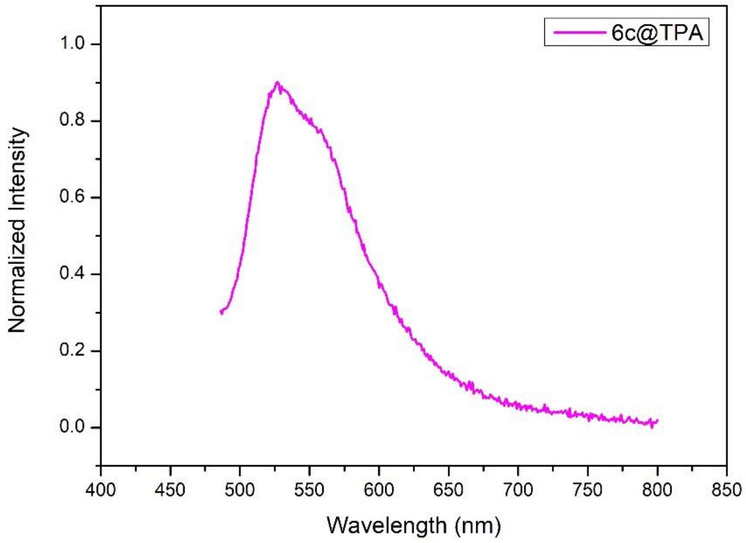

Figure S23. Normalized photoluminescence spectrum (298K) of **6c**@TPA (1 wt%) film (λ_{ex} = 370 nm).

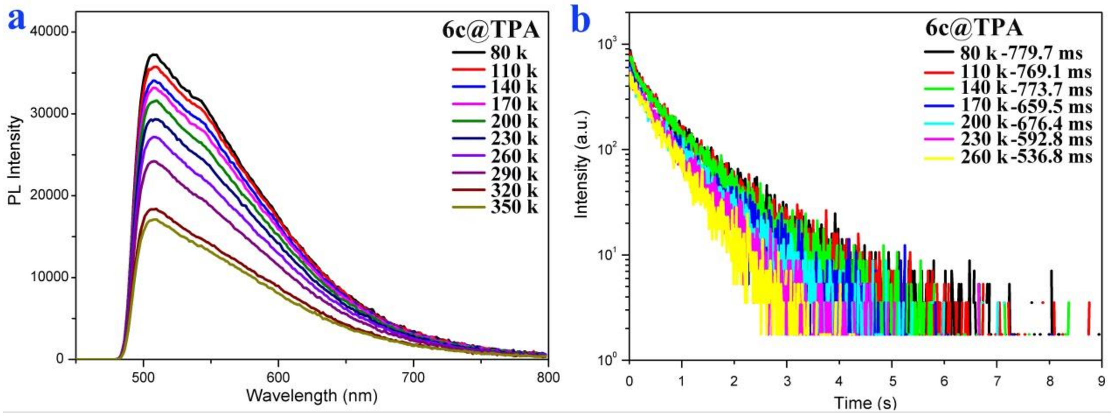

Figure S24. (a) Temperature-variable PL spectra of **6c**@TPA in PMMA film (1.0 wt%). (b) Temperature-dependent time-resolved phosphorescence decay curves of **6c**@TPA in PMMA film (1.0 wt%) (λ_{em} = 572 nm).

IX. The excited state lifetimes of 3b, 4a, 5a, 5b, 6a-6c in solution and films

Table S2. The excited state lifetimes of **3b**, **4a, 5a, 5b, 6a-6c** [a].

<table>
<tr><th>Compound</th><th>λ_{em} [nm]</th><th>Lifetime</th></tr>
<tr><td>3b in CBZ_2-F_1 film</td><td>540</td><td>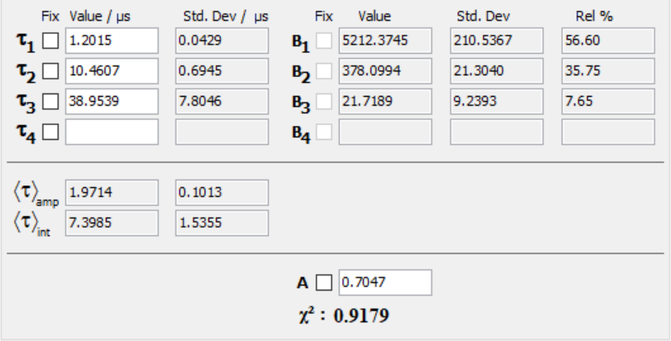
<table>
<tr><th></th><th>Fix</th><th>Value / μs</th><th>Std. Dev / μs</th><th></th><th>Fix</th><th>Value</th><th>Std. Dev</th><th>Rel %</th></tr>
<tr><td>τ_1</td><td>☐</td><td>1.2015</td><td>0.0429</td><td>B_1</td><td>☐</td><td>5212.3745</td><td>210.5367</td><td>56.60</td></tr>
<tr><td>τ_2</td><td>☐</td><td>10.4607</td><td>0.6945</td><td>B_2</td><td>☐</td><td>378.0994</td><td>21.3040</td><td>35.75</td></tr>
<tr><td>τ_3</td><td>☐</td><td>38.9539</td><td>7.8046</td><td>B_3</td><td>☐</td><td>21.7189</td><td>9.2393</td><td>7.65</td></tr>
<tr><td>τ_4</td><td>☐</td><td></td><td></td><td>B_4</td><td>☐</td><td></td><td></td><td></td></tr>
<tr><td>$\langle\tau\rangle_{amp}$</td><td></td><td>1.9714</td><td>0.1013</td><td></td><td></td><td></td><td></td><td></td></tr>
<tr><td>$\langle\tau\rangle_{int}$</td><td></td><td>7.3985</td><td>1.5355</td><td></td><td></td><td></td><td></td><td></td></tr>
</table>
A ☐ 0.7047
χ^2 : 0.9179</td></tr>
</table>

<table>
<tr><td>4a in CBZ_2-F_1 film</td><td>505</td><td>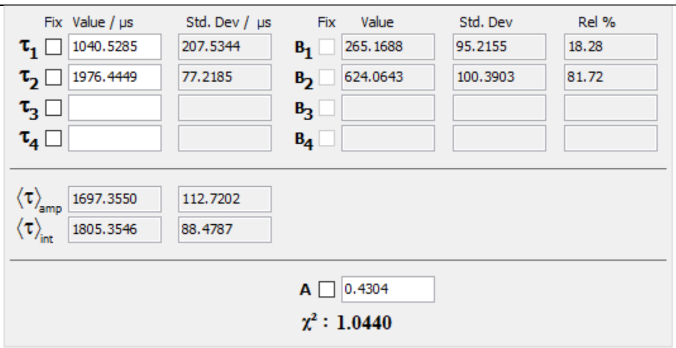
</td></tr>
<tr><td rowspan="2">5a in DCM</td><td>421</td><td>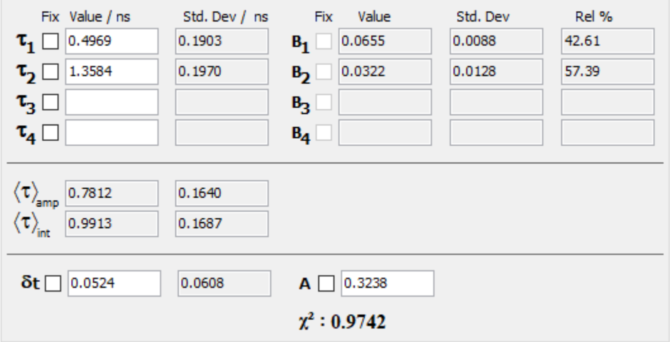
</td></tr>
<tr><td>609</td><td>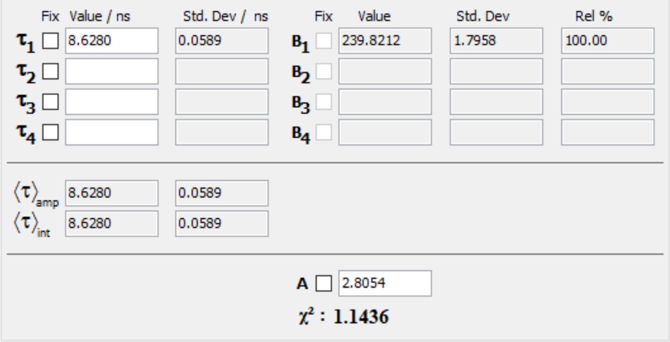
</td></tr>
<tr><td rowspan="2">5b in PMMA film (RT)</td><td>430</td><td>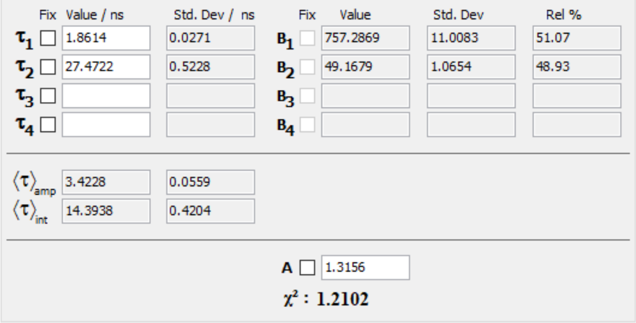
</td></tr>
<tr><td>598</td><td>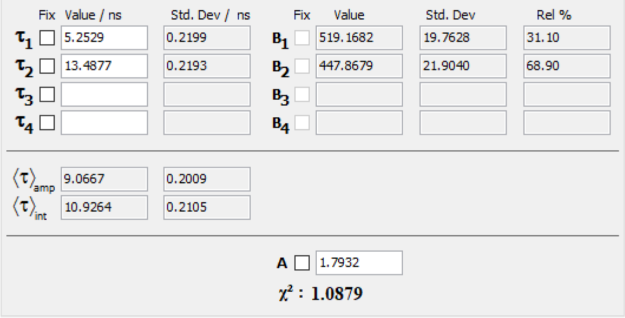
</td></tr>
</table>

<table>
<tr><td rowspan="2">5b in PMMA film (77 K)</td><td>430</td><td>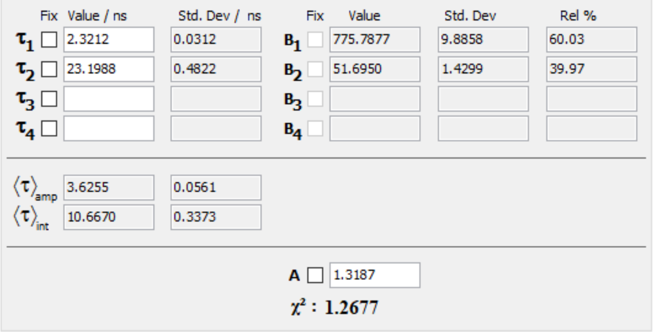
</td></tr>
<tr><td>598</td><td>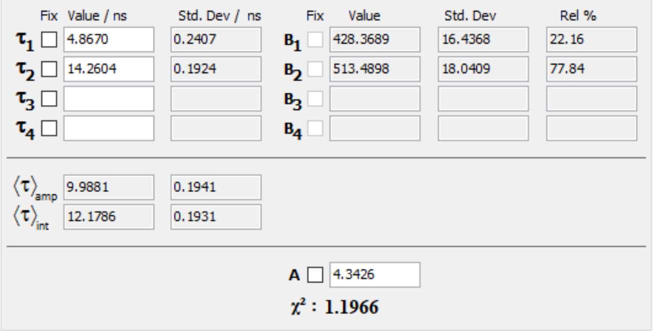
</td></tr>
<tr><td>6a@TPA (1 wt%) film</td><td>572</td><td>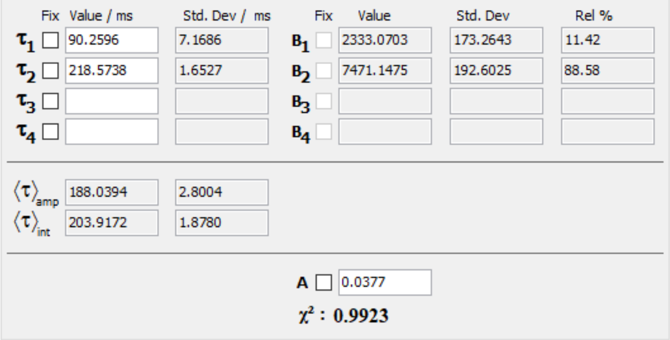
</td></tr>
<tr><td>6b@TPA (1 wt%) film</td><td>565</td><td>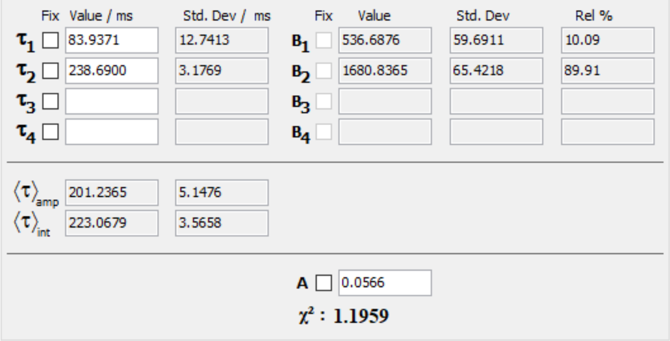
</td></tr>
</table>

**6c**@TPA (1 wt%) film	528	Fix Value / ns, Std. Dev / ns, Fix Value, Std. Dev, Rel %<br>τ_1 2.8735, 0.1428; B_1 413.9392, 16.3382, 63.54<br>τ_2 8.7738, 0.9172; B_2 77.7822, 18.2336, 36.46<br>τ_3; B_3<br>τ_4; B_4<br>$\langle\tau\rangle_{amp}$ 3.8068, 0.2653<br>$\langle\tau\rangle_{int}$ 5.0246, 0.5778<br>A 22.7556<br>χ^2 : 1.0264
	570	Fix Value / ms, Std. Dev / ms, Fix Value, Std. Dev, Rel %<br>τ_1 8.5681, 0.3585; B_1 178.5712, 6.4489, 17.67<br>τ_2 166.8290, 3.0975; B_2 42.7386, 0.7882, 82.33<br>τ_3; B_3<br>τ_4; B_4<br>$\langle\tau\rangle_{amp}$ 39.1309, 1.2006<br>$\langle\tau\rangle_{int}$ 138.8682, 3.2477<br>A 0.5462<br>χ^2 : 1.1784
**6c**@TPA PMMA (1 wt%) film	511	Fix Value / ns, Std. Dev / ns, Fix Value, Std. Dev, Rel %<br>τ_1 2.8834, 0.0618; B_1 806.3418, 10.8492, 49.86<br>τ_2 11.1030, 0.1928; B_2 210.6209, 8.3511, 50.14<br>τ_3; B_3<br>τ_4; B_4<br>$\langle\tau\rangle_{amp}$ 4.5858, 0.0848<br>$\langle\tau\rangle_{int}$ 7.0051, 0.1584<br>A 1.4197<br>χ^2 : 1.0079
	572	Fix Value / ms, Std. Dev / ms, Fix Value, Std. Dev, Rel %<br>τ_1 105.4754, 102.0492; B_1 23.5439, 12.7316, 1.59<br>τ_2 515.3651, 9.4622; B_2 297.5534, 10.1929, 98.41<br>τ_3; B_3<br>τ_4; B_4<br>$\langle\tau\rangle_{amp}$ 485.3106, 18.9896<br>$\langle\tau\rangle_{int}$ 508.8332, 11.0516<br>A 0.0532<br>χ^2 : 1.296

[a]The excited state lifetimes were determined on an Edinburgh FLS1000 steady transient fluorescence spectrometer.

(a) 3b in CBZ_2-F_1 film- 540 nm

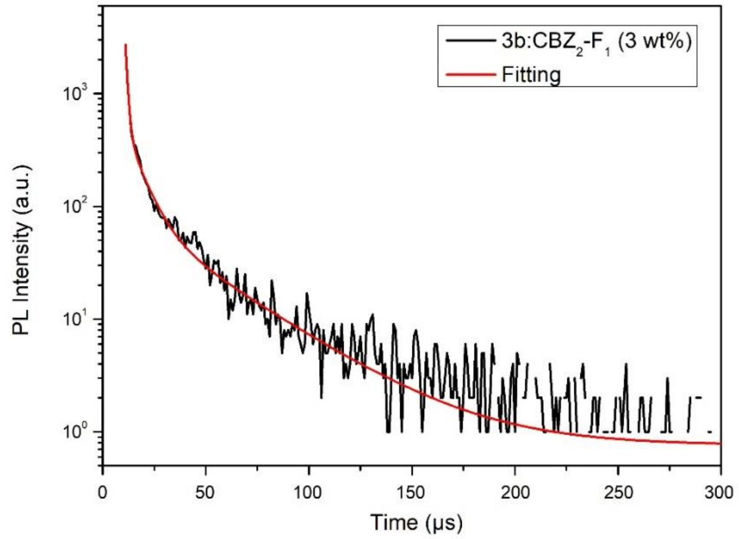

Figure S25. Fluorescence lifetime decay curves of **3b** in CBZ_2-F_1 film (3 wt%).

(b) 4a in CBZ_2-F_1 film- 505 nm

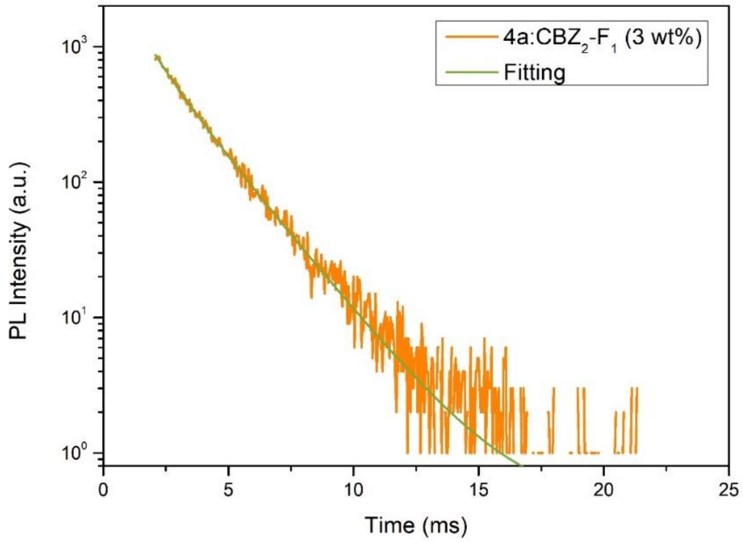

Figure S26. Fluorescence lifetime decay curves of **4a** in CBZ_2-F_1 film (3 wt%).

(c) 5a in DCM-421 nm and 609 nm

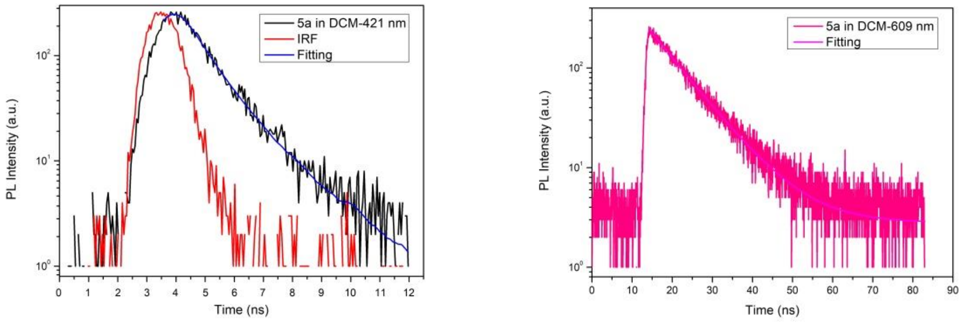

Figure S27. Fluorescence lifetime decay curves of **5a** in CH_2Cl_2 (Concentration: 5.0 $\times 10^{-5}$ M).

(d) 5b in PMMA film-430 nm and 598 nm (298 K)

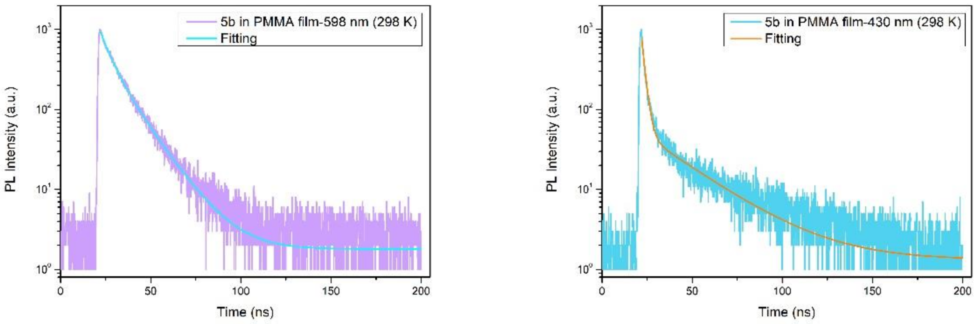

Figure S28. Fluorescence lifetime decay curves (298 K) of **5b** in PMMA film (0.0125 wt%).

(e) 5b in PMMA film-430 nm and 598 nm (77 K)

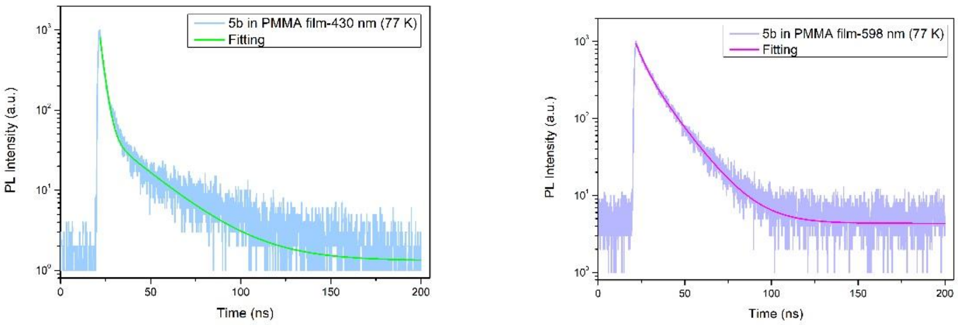

Figure S29. Fluorescence lifetime decay curves (77 K) of **5b** in PMMA film (0.0125 wt%).

(f) 6a@TPA (1 wt%) film-572 nm

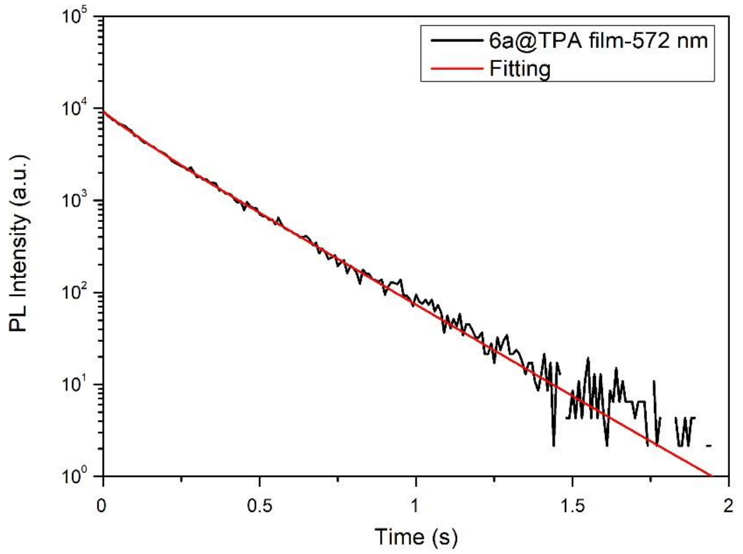

Figure S30. Phosphorescence lifetime decay curves of **6a**@TPA (1 wt%) film.

(g) 6b@TPA (1 wt%) film-565 nm

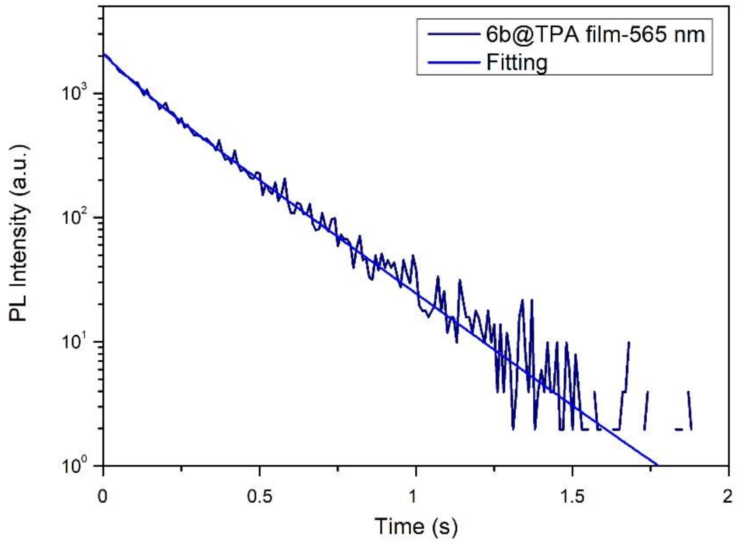

Figure S31. Phosphorescence lifetime decay curves of **6b**@TPA (1 wt%) film.

(h) 6c@TPA (1 wt%) film-528 nm and 570 nm

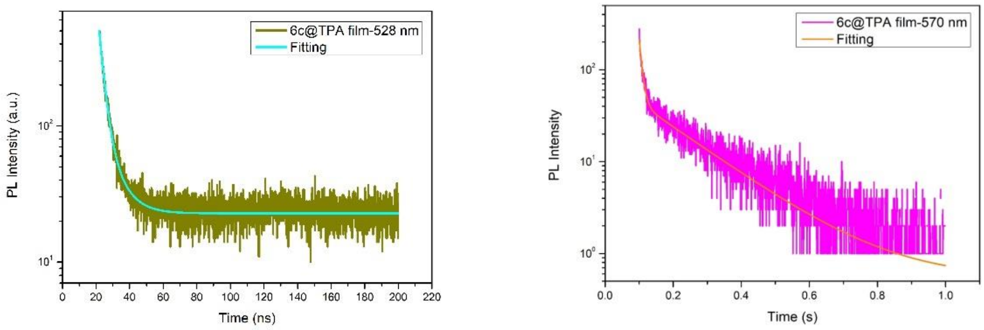

Figure S32. Phosphorescence lifetime decay curves of **6c**@TPA (1 wt%) film.

(i) 6c@TPA PMMA film (1 wt%)-511 nm and 572 nm

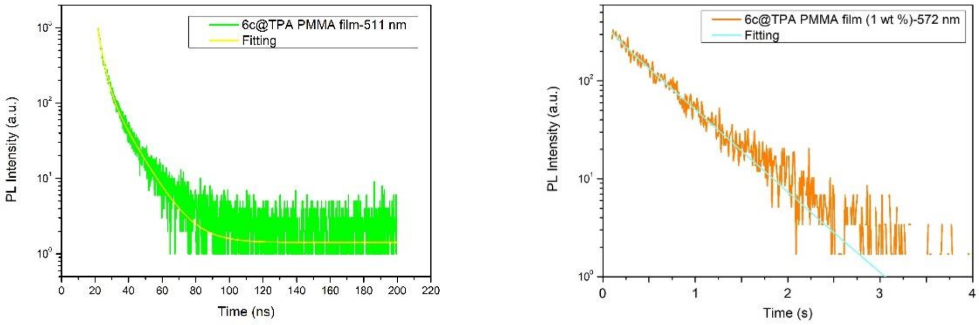

Figure S33. Phosphorescence lifetime decay curves of **6c**@TPA PMMA film (1 wt%).

X. Photoluminescence quantum yields

Table S3. The PLQY of **3b**, **3k**, **4m**, **5b**, **5c**, **6b**, **6c** in toluene (concentration: 50 μM**)** and films.[a]

Compound	PLQY

**3b**	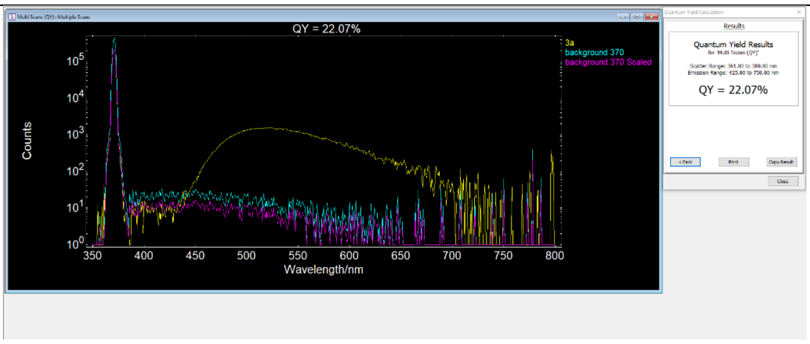 QY = 22.07%<br>Counts<br>Wavelength/nm<br>3a<br>background 370<br>background 370 Scaled<br>Results<br>Quantum Yield Results<br>QY = 22.07%
**3k**	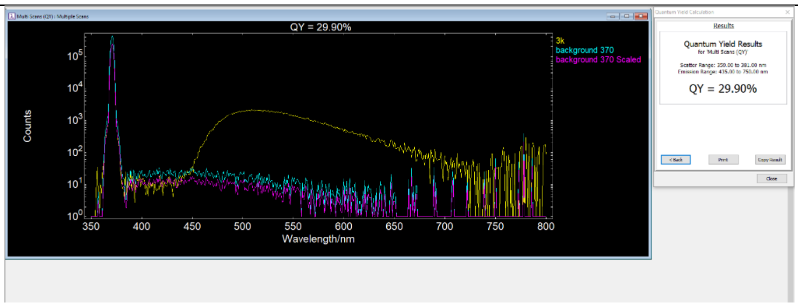 QY = 29.90%<br>Counts<br>Wavelength/nm<br>3k<br>background 370<br>background 370 Scaled<br>Results<br>Quantum Yield Results<br>QY = 29.90%
**4m**	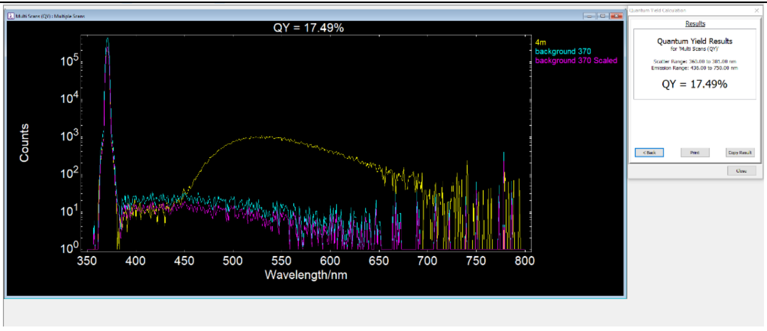 QY = 17.49%<br>Counts<br>Wavelength/nm<br>4m<br>background 370<br>background 370 Scaled<br>Results<br>Quantum Yield Results<br>QY = 17.49%
**5b**	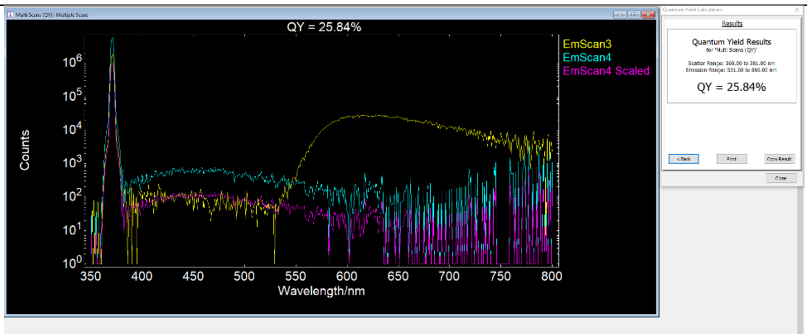 QY = 25.84%<br>Counts<br>Wavelength/nm<br>EmScan3<br>EmScan4<br>EmScan4 Scaled<br>Results<br>Quantum Yield Results<br>QY = 25.84%
**5c**	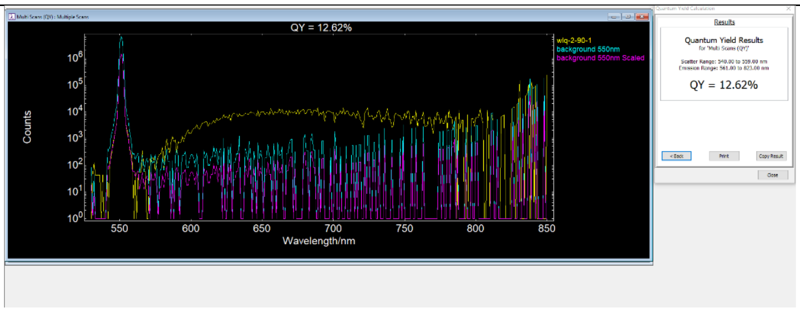 QY = 12.62%<br>Counts<br>Wavelength/nm<br>background 550nm<br>background 550nm Scaled<br>Results<br>Quantum Yield Results<br>QY = 12.62%

**6c**@TPA PMMA film **(1 wt%)**	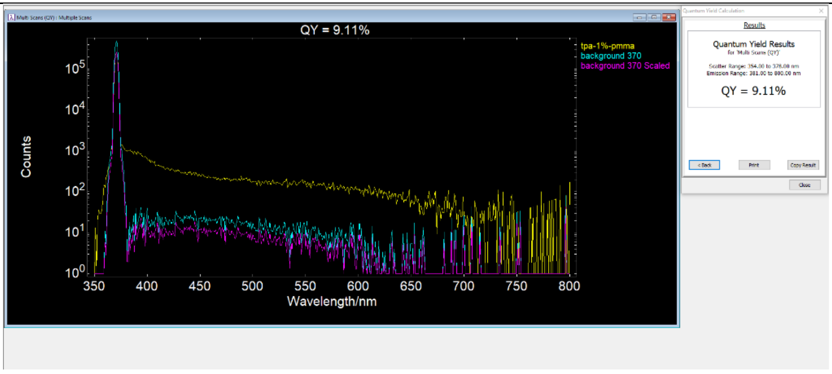

[a] The determination of quantum yield data for these compounds is the absolute quantum yields. The absolute quantum yields were taken using Edinburgh Instruments FLS 1000 fluorescence spectrometer with a calibrated integrating sphere system.

XI. Calculation

DFT calculations were performed using the Gaussian 16, Revision A.03.[1,2] Geometries were optimized in dichloromethane with the CPCM solvation model by the B3-LYP functional and a basis set of 6-31G(d,p). Time dependent DFT (TDDFT) with the CAM-B3LYP functional and 6-31+G(d,p) basis set. Vibrational frequencies were calculated at the same level and check the optimized structure. The SOC calculations at B3LYP functional with def2-TZVP basis set level were performed based on optimized structure in ORCA software on the grounds of previous literatures.[1,2] The time-dependent density functional theory (TDDFT) calculations of the excitation energies were calculated at the optimized geometries of the ground states. Calculations were performed at the cam-b3lyp/6-31g scrf = (cpcm,solvent = dichloromethane) (**5a**).

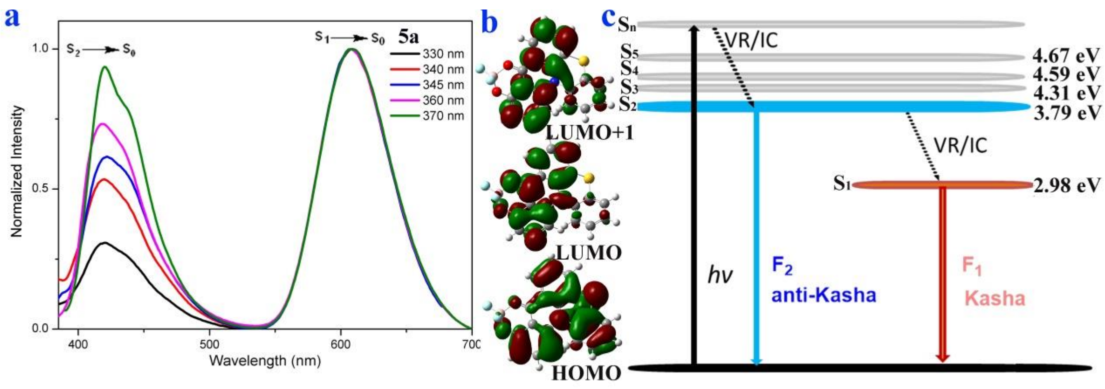

Figure S34. (a) Excitation-wavelength-dependent fluorescence spectra of **5a** (50 μM in CH_2Cl_2). (b) Molecular orbitals of the S_0, S_1 or S_2 states of **5a**. (c) Jablonski diagram illustrating the anti-Kasha dual-emission mechanism of **5a**.

Cartesian coordinates of the structures

5a

C	3.4908	-2.46227	1.45176
C	4.68545	-1.80606	1.13338
C	4.6439	-0.62197	0.39422
C	3.42077	-0.12464	-0.06525
C	2.22351	-0.80165	0.20558
C	2.2683	-1.95547	1.00878
S	3.3782	1.45884	-0.99225
C	1.76251	2.04144	-0.33872
C	0.71511	1.11462	-0.1584
N	0.95121	-0.27247	-0.24035
C	1.53566	3.40289	-0.17399
C	0.24765	3.88482	0.1296
C	-0.81038	2.99744	0.239
C	-0.59248	1.61016	0.08828
C	-1.69012	0.67868	0.10763
C	-0.13034	-1.14102	-0.50337
C	-1.45627	-0.66654	-0.26753
C	-2.58011	-1.52298	-0.49468
C	-2.37382	-2.81278	-0.99094
C	-1.07018	-3.24396	-1.242
C	0.05016	-2.4377	-0.99923

O	-2.89101	1.11783	0.39018
O	-3.82717	-1.0753	-0.27252
B	-4.14582	0.17625	0.48783
F	-5.20961	0.84863	-0.10808
F	-4.32795	-0.09979	1.85339
H	3.5048	-3.3604	2.05919
H	5.63661	-2.19633	1.47785
H	5.55377	-0.07247	0.18052
H	1.34802	-2.45596	1.28084
H	2.3584	4.09624	-0.30918
H	0.08682	4.94965	0.24996
H	-1.82228	3.33486	0.42533
H	-3.23322	-3.44373	-1.17473
H	-0.91343	-4.23854	-1.64647
H	1.04263	-2.80663	-1.21771

Gaussian optimization:

opt b3lyp/6-31+g(d,p) scrf=(cpcm,solvent=dichloromethane) geom=conne ctivity

6a

C	-2.8391	-1.181	3.0352
C	-2.0231	-2.1148	2.5301
C	-1.2293	-1.8468	1.4772
C	-1.2564	-0.6551	0.85
C	-2.0297	0.2899	1.4403
S	-0.1436	-3.1755	0.9664
C	0.9843	-2.0691	0.0987
C	0.5794	-0.8986	-0.4418
N	-0.51	-0.3082	-0.1307
C	2.2107	-2.561	-0.1514
C	3.0676	-1.8949	-0.9317
C	2.6634	-0.7608	-1.5149
C	1.4237	-0.2756	-1.3026
C	1.0136	0.8208	-2.014
C	-1.1013	0.292	-1.0983
C	-0.3408	0.9497	-1.9989
C	-0.8583	1.7154	-2.9695
C	-2.1903	1.8003	-3.1005
C	-2.9798	1.104	-2.2744
C	-2.438	0.3657	-1.2942
O	1.8103	1.4292	-2.7467

O	-0.0022	2.3476	-3.8081
B	1.3634	2.8312	-2.9503
F	0.454	3.9832	-2.4551
F	2.0263	3.1258	-1.5814
O	0.685	-3.7302	2.0336
O	-0.6104	-3.9832	-0.1578
H	-3.4461	0.8431	2.9248
H	-3.4782	-1.4008	3.9062
H	-2.0088	-3.1064	3.0143
H	-2.0273	1.3349	1.079
H	2.5535	-3.5123	0.2894
H	4.0751	-2.2995	-1.1267
H	3.3849	-0.2881	-2.2019
H	-2.6285	2.4116	-3.9062
H	-4.0751	1.1458	-2.4
H	-3.16	-0.1777	-0.6616

6b

C	-3.3447	-1.1354	-1.6681
C	-3.4277	-2.4582	-1.4788
C	-2.5321	-3.0586	-0.684
C	-1.5734	-2.3427	-0.071
C	-1.4894	-0.998	-0.2155
C	-2.3876	-0.4262	-1.0504
S	-0.3605	-3.2132	0.9365
C	0.7938	-2.1426	0.06
C	0.5475	-0.8187	-0.0485
N	-0.5531	-0.372	0.4107
C	1.9212	-2.6604	-0.4474
C	2.7902	-1.8544	-1.0675
C	2.5716	-0.532	-1.1633
C	1.4525	-0.0041	-0.6303
C	1.2401	1.3465	-0.684
C	-0.7509	0.8815	0.4978
C	0.098	1.7723	-0.0552
C	-0.2007	3.0859	0.0404
C	-1.2881	3.5162	0.6978
C	-2.0974	2.632	1.2932
C	-1.8211	1.325	1.1922
O	1.9692	2.2114	-1.1928
B	3.3767	1.7703	-1.1812
F	3.9411	1.8144	0.2566

F	4.21	2.723	-2.067
O	3.4856	0.2491	-1.7871
O	-0.4528	-2.8285	2.3397
O	-0.2168	-4.595	0.4955
H	-4.062	-0.6337	-2.3397
H	-4.21	-3.0466	-1.9862
H	-2.5977	-4.1516	-0.554
H	-2.3743	0.6534	-1.278
H	2.1425	-3.738	-0.3767
H	3.7111	-2.2833	-1.4976
H	0.4326	3.8754	-0.3972
H	-1.5069	4.595	0.7698
H	-2.9825	2.9758	1.8537
H	-2.5057	0.6146	1.6891

6c

C	-3.286	-2.1461	-1.7709
C	-3.2952	-3.4388	-1.4226
C	-2.4034	-3.8763	-0.5238
C	-1.5226	-3.0278	0.0343
C	-1.5164	-1.7078	-0.272
C	-2.4065	-1.3037	-1.2076
S	-0.3072	-3.6848	1.1898
C	0.8175	-2.6528	0.2322
C	0.4941	-1.3713	-0.048
N	-0.6527	-0.9482	0.309
C	1.9973	-3.1503	-0.1645
C	2.841	-2.3678	-0.8468
C	2.5444	-1.0845	-1.1127
C	1.3719	-0.5728	-0.6906
C	1.0786	0.7443	-0.917
C	-0.9308	0.2905	0.2333
C	-0.1158	1.1652	-0.3889
C	-0.5042	2.4567	-0.4655
C	-1.635	2.9182	0.0987
C	-2.4173	2.0346	0.7375
C	-2.0599	0.7459	0.8147
O	1.7743	1.5913	-1.4979
O	3.4336	-0.3241	-1.7949
B	3.2047	1.2483	-1.3854
F	3.7047	1.5032	0.0543

F	4.0134	2.1405	-2.3532
O	-0.4908	-3.137	2.5284
O	-0.0567	-5.0972	0.9317
F	-3.3514	4.5498	0.1055
C	-2.0505	4.3651	-0.0555
F	-1.7201	4.8565	-1.24
F	-1.4311	5.0972	0.858
H	-3.9996	-1.7795	-2.5284
H	-4.0134	-4.1363	-1.8846
H	-2.4064	-4.9476	-0.2623
H	-2.4492	-0.2614	-1.5668
H	2.2821	-4.1939	0.0466
H	3.8052	-2.7822	-1.1867
H	0.1076	3.2132	-0.9822
H	-3.3614	2.3566	1.2062
H	-2.7304	0.0565	1.3585

The energy levels calculation：

tda = (50-50, nstates = 6) cam-b3lyp/6-31+g(d,p) scrf = (cpcm, solvent = dichloromethane) geom = connectivity

6a

C	-2.84592	-2.93412	-1.58443
C	-4.10551	-2.35774	-1.38619
C	-4.20393	-1.15376	-0.69574
C	-3.04778	-0.56476	-0.17785
C	-1.77936	-1.14312	-0.34571
C	-1.69288	-2.33207	-1.08523
S	-3.15715	0.9847	0.67962
C	-1.65405	1.72763	0.08042
C	-0.50489	0.91214	0.01161
N	-0.59282	-0.47655	0.11399
C	-1.58929	3.09921	-0.12753
C	-0.35476	3.71319	-0.38454
C	0.7997	2.94766	-0.38481
C	0.74553	1.55329	-0.17961
C	1.93874	0.75016	-0.13488
C	0.55866	-1.21093	0.45534
C	1.833	-0.60377	0.25862
C	3.03053	-1.31822	0.57103
C	2.94202	-2.59985	1.11242

C	1.68071	-3.1583	1.32585
C	0.4937	-2.49293	1.00248
O	3.08559	1.29944	-0.37458
O	4.21313	-0.73344	0.39764
B	4.35423	0.46198	-0.44123
F	4.5406	0.10818	-1.78076
F	5.39348	1.24495	0.03116
O	-4.32507	1.75073	0.20591
O	-3.01264	0.75536	2.13338
H	-2.75239	-3.85217	-2.15518
H	-4.99642	-2.83023	-1.78512
H	-5.15943	-0.65918	-0.55992
H	-0.72662	-2.7753	-1.28925
H	-2.49889	3.68752	-0.06552
H	-0.30594	4.78317	-0.54997
H	1.77003	3.40556	-0.53859
H	3.84937	-3.13418	1.36919
H	1.61273	-4.14727	1.76822
H	-0.45974	-2.96429	1.20053

6b

C	-3.34470000	-1.13540000	-1.66810000
C	-3.42770000	-2.45820000	-1.47880000
C	-2.53210000	-3.05860000	-0.68400000
C	-1.57340000	-2.34270000	-0.07100000
C	-1.48940000	-0.99800000	-0.21550000
C	-2.38760000	-0.42620000	-1.05040000
S	-0.36050000	-3.21320000	0.93650000
C	0.79380000	-2.14260000	0.06000000
C	0.54750000	-0.81870000	-0.04850000
N	-0.55310000	-0.37200000	0.41070000
C	1.92120000	-2.66040000	-0.44740000
C	2.79020000	-1.85440000	-1.06750000
C	2.57160000	-0.53200000	-1.16330000
C	1.45250000	-0.00410000	-0.63030000
C	1.24010000	1.34650000	-0.68400000
C	-0.75090000	0.88150000	0.49780000
C	0.09800000	1.77230000	-0.05520000
C	-0.20070000	3.08590000	0.04040000
C	-1.28810000	3.51620000	0.69780000
C	-2.09740000	2.63200000	1.29320000
C	-1.82110000	1.32500000	1.19220000
O	1.96920000	2.21140000	-1.19280000

B	3.37670000	1.77030000	-1.18120000
F	3.94110000	1.81440000	0.25660000
F	4.21000000	2.72300000	-2.06700000
O	3.48560000	0.24910000	-1.78710000
O	-0.45280000	-2.82850000	2.33970000
O	-0.21680000	-4.59500000	0.49550000
H	-4.06200000	-0.63370000	-2.33970000
H	-4.21000000	-3.04660000	-1.98620000
H	-2.59770000	-4.15160000	-0.55400000
H	-2.37430000	0.65340000	-1.27800000
H	2.14250000	-3.73800000	-0.37670000
H	3.71110000	-2.28330000	-1.49760000
H	0.43260000	3.87540000	-0.39720000
H	-1.50690000	4.59500000	0.76980000
H	-2.98250000	2.97580000	1.85370000
H	-2.50570000	0.61460000	1.68910000

6c

C	-1.57762	-2.9226	2.2458
C	-2.88692	-3.09517	2.02574
C	-3.47088	-2.42794	1.02158
C	-2.75094	-1.60987	0.23439
C	-1.41746	-1.44626	0.41139
C	-0.86402	-2.10725	1.45432
S	-3.60182	-0.69172	-1.06062
C	-2.60625	0.66997	-0.42689
C	-1.28305	0.50316	-0.21087
N	-0.78598	-0.65596	-0.38686
C	-3.17716	1.86345	-0.21132
C	-2.42479	2.87862	0.22804
C	-1.10333	2.73543	0.42494
C	-0.52092	1.54527	0.18186
C	0.83079	1.40024	0.33639
C	0.47501	-0.82094	-0.37537
C	1.31695	0.1615	0.00201
C	2.64045	-0.10728	0.02994
C	3.15839	-1.28769	-0.35527
C	2.30338	-2.24198	-0.75412
C	0.98542	-2.00344	-0.77716
O	1.65291	2.25594	0.69799
O	-0.37705	3.79345	0.85781

B	1.17382	3.60834	0.35475
F	1.27172	3.84003	-1.16986
F	2.06006	4.65233	1.06982
O	-3.14328	-1.08763	-2.38676
O	-5.00732	-0.5026	-0.72442
F	4.93387	-2.84654	-0.19446
C	4.64458	-1.55646	-0.26071
F	5.19282	-0.96883	0.7918
F	5.24559	-1.07116	-1.33653
H	-1.09103	-3.44791	3.08526
H	-3.47903	-3.76132	2.67491
H	-4.55429	-2.56122	0.86492
H	0.2022	-2.00209	1.7177
H	-4.25536	2.02355	-0.37448
H	-2.89793	3.85644	0.42029
H	3.37544	0.64721	0.35259
H	2.67313	-3.23178	-1.06761
H	0.32033	-2.81557	-1.12163

The SOC calculations at B_3LYP functional with def2-TZVP basis set level

6a

C	-2.84591533	-2.93412162	-1.58442573
C	-4.10551161	-2.35774390	-1.38619004
C	-4.20392880	-1.15375510	-0.69574383
C	-3.04777725	-0.56475899	-0.17785211
C	-1.77935580	-1.14311819	-0.34571130
C	-1.69287934	-2.33206581	-1.08522683
S	-3.15715185	0.98469704	0.67962181
C	-1.65405436	1.72762975	0.08042404
C	-0.50489394	0.91213982	0.01160826
N	-0.59282377	-0.47655167	0.11398526
C	-1.58928527	3.09921278	-0.12753476
C	-0.35476217	3.71318529	-0.38453654
C	0.79970448	2.94766337	-0.38480571
C	0.74552866	1.55328772	-0.17960523
C	1.93874214	0.75015531	-0.13487630
C	0.55865992	-1.21093387	0.45534145
C	1.83300220	-0.60376733	0.25861608
C	3.03053289	-1.31821818	0.57102775
C	2.94201523	-2.59985393	1.11242451
C	1.68071320	-3.15830186	1.32585014
C	0.49369998	-2.49292713	1.00248369

O	3.08559284	1.29944419	-0.37458405
O	4.21313390	-0.73343783	0.39763623
B	4.35422518	0.46198489	-0.44123469
F	4.54060443	0.10818257	-1.78076056
F	5.39348094	1.24494695	0.03115855
O	-4.32506504	1.75072632	0.20590847
O	-3.01263724	0.75536015	2.13338220
H	-2.75239226	-3.85217473	-2.15518286
H	-4.99641585	-2.83023264	-1.78511509
H	-5.15942749	-0.65918285	-0.55991523
H	-0.72662283	-2.77530245	-1.28924837
H	-2.49888664	3.68752330	-0.06552100
H	-0.30593565	4.78316952	-0.54997080
H	1.77002886	3.40555889	-0.53859090
H	3.84936551	-3.13417771	1.36918703
H	1.61272680	-4.14726609	1.76822274
H	-0.45974313	-2.96428773	1.20053224

6b

C	3.68203554	1.86543814	1.83589740
C	4.61358959	0.85075025	1.59146151
C	4.25234199	-0.23030949	0.79271581
C	2.98163667	-0.26172151	0.21358706
C	2.04592512	0.76421030	0.42513823
C	2.40728405	1.82009041	1.27504159
S	2.50321660	-1.65560458	-0.78083335
C	0.81116724	-1.77167144	-0.27386179
C	0.09057541	-0.57735596	-0.13314900
N	0.71280519	0.66796546	-0.10273913
C	0.16106510	-3.01140115	-0.21834367
C	-1.21319539	-3.10252445	-0.03773703
C	-1.96986023	-1.92759721	0.06356762
C	-1.31907986	-0.65786875	0.00253229
C	-2.10441625	0.51981034	-0.01163346
C	-0.04756148	1.81936641	-0.38277540
C	-1.46598564	1.77111431	-0.30493283
C	-2.22741075	2.93452375	-0.56248494
C	-1.60376516	4.11325037	-0.92103819
C	-0.20157564	4.14182449	-1.04796185
C	0.56898501	3.01959923	-0.78243556
O	-3.38846103	0.47028917	0.15146783
B	-4.09055390	-0.80867482	0.56429514
F	-5.30650240	-0.85099956	-0.09405918

F	-4.25094250	-0.76705302	1.94959158
O	-3.28500807	-1.98582267	0.18259775
O	2.55561708	-1.27184934	-2.20926169
O	3.24485575	-2.86099018	-0.36155680
H	3.93711338	2.69184015	2.49107057
H	5.60086630	0.89024716	2.03853793
H	4.93562394	-1.05458670	0.62009535
H	1.68849734	2.59158325	1.52010942
H	0.75218115	-3.91435871	-0.33289696
H	-1.71224896	-4.06308623	0.00693725
H	-3.30688432	2.86944081	-0.49127033
H	-2.18835432	5.00313119	-1.12676877
H	0.29231410	5.05181848	-1.37319337
H	1.64300132	3.06354913	-0.90888426

6c

C	-1.57762345	-2.92260255	2.24579836
C	-2.88692348	-3.09517365	2.02574444
C	-3.47088301	-2.42794192	1.02157898
C	-2.75093982	-1.60986630	0.23438723
C	-1.41746268	-1.44625981	0.41139009
C	-0.86401565	-2.10725419	1.45432117
S	-3.60181998	-0.69172090	-1.06061813
C	-2.60624748	0.66996666	-0.42688735
C	-1.28304865	0.50316135	-0.21087122
N	-0.78598495	-0.65596323	-0.38685693
C	-3.17715639	1.86344807	-0.21131700
C	-2.42479046	2.87862247	0.22804155
C	-1.10332925	2.73542827	0.42494049
C	-0.52092056	1.54527117	0.18186025
C	0.83079025	1.40024331	0.33638963
C	0.47500683	-0.82093517	-0.37536735
C	1.31694612	0.16150103	0.00201246
C	2.64045330	-0.10728073	0.02993639
C	3.15839251	-1.28769054	-0.35527063
C	2.30337979	-2.24198429	-0.75412480
C	0.98542307	-2.00344244	-0.77715586
O	1.65291270	2.25593622	0.69799281
O	-0.37704809	3.79345364	0.85781449
B	1.17382022	3.60834423	0.35475427
F	1.27171766	3.84002813	-1.16986015
F	2.06006204	4.65232924	1.06982380
O	-3.14328358	-1.08762615	-2.38675820

O	-5.00732470	-0.50260432	-0.72441681
F	4.93387244	-2.84653830	-0.19446257
C	4.64458197	-1.55646163	-0.26071482
F	5.19282495	-0.96882718	0.79180312
F	5.24559070	-1.07116022	-1.33653094
H	-1.09102568	-3.44791357	3.08526122
H	-3.47903079	-3.76131878	2.67491411
H	-4.55428994	-2.56121578	0.86491815
H	0.20219738	-2.00209405	1.71770020
H	-4.25535874	2.02354577	-0.37448497
H	-2.89793251	3.85643500	0.42029254
H	3.37544022	0.64721315	0.35258754
H	2.67312650	-3.23178489	-1.06760657
H	0.32032824	-2.81556572	-1.12163496

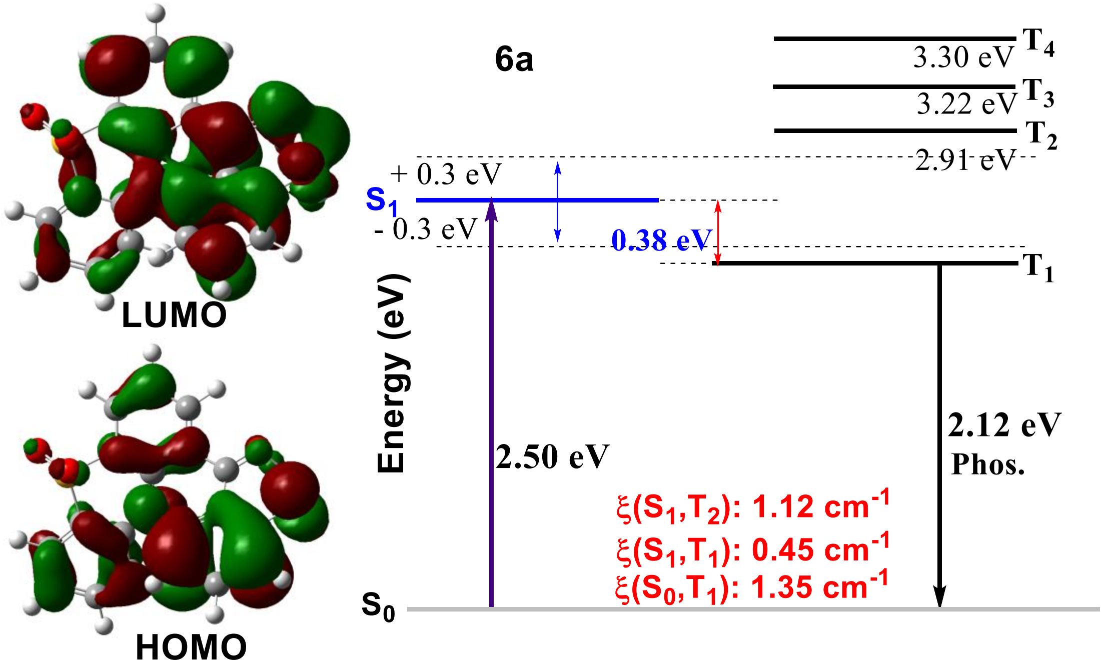

Figure S35. Molecular orbitals, energy levels, and SOC values between singlet and triplet states of **6a**.

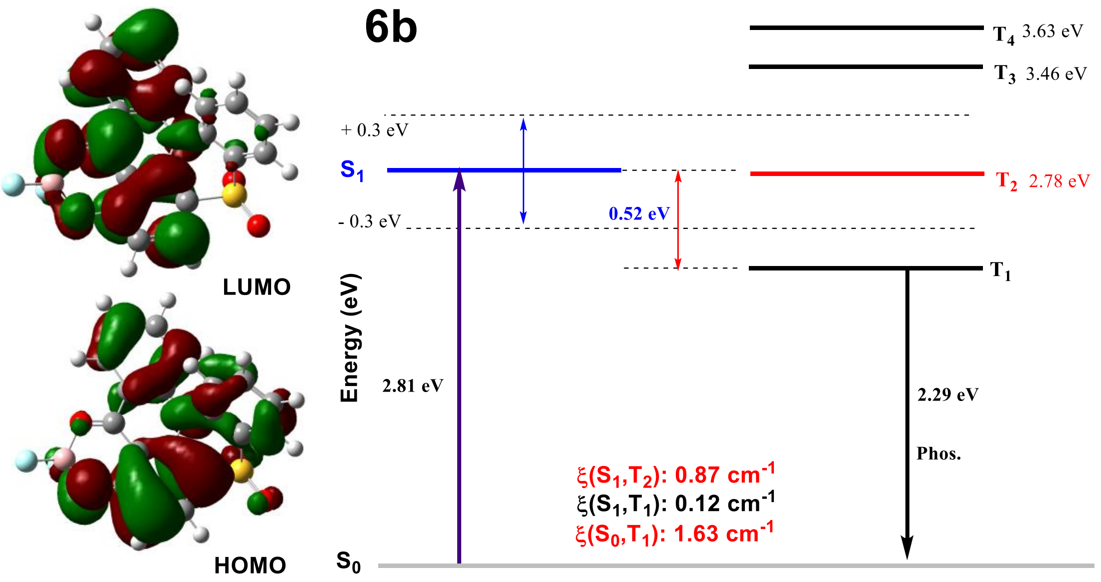

Figure S36. Molecular orbitals, energy levels, and SOC values between singlet and triplet states of **6b**.

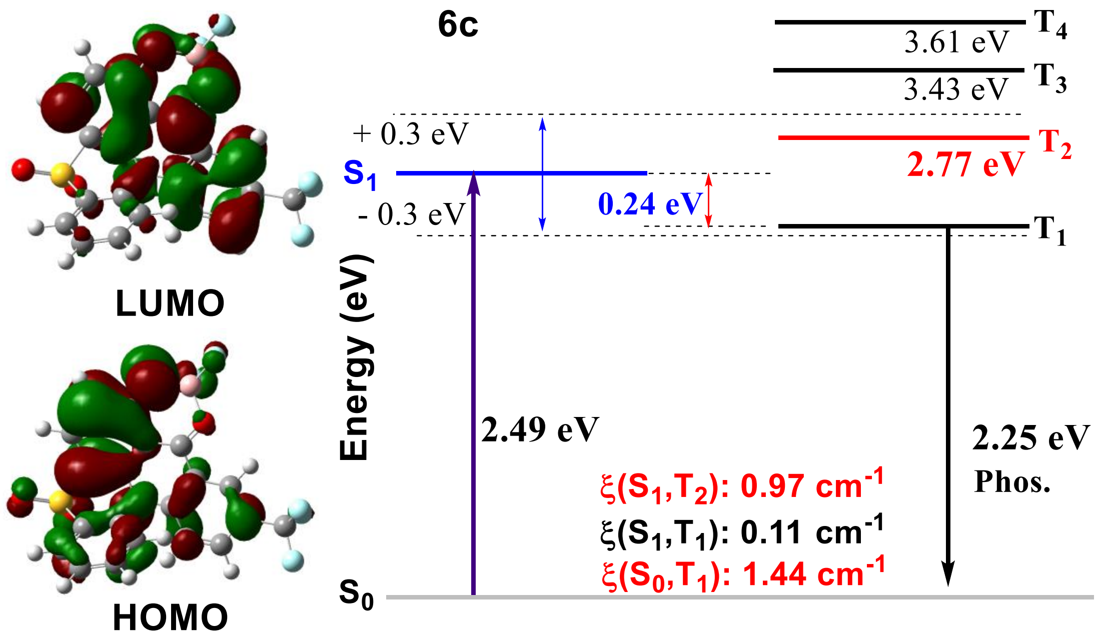

Figure S37. Molecular orbitals, energy levels, and SOC values between singlet and triplet states of **6c**.

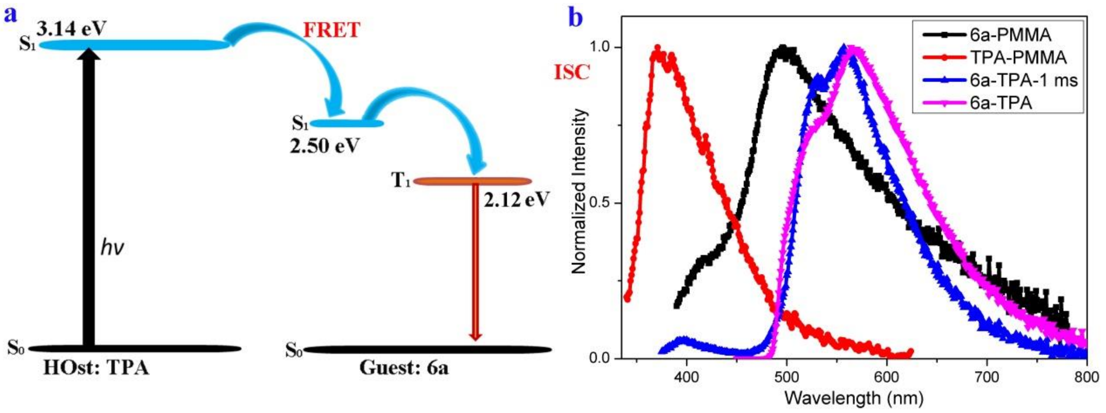

Figure S38. (a) Possible luminescent mechanism of RTP behaviour of host–guest doped system based on **6a**@TPA. (b) Normalized emission spectrum of **6a** in PMMA film (1.0 wt%); Normalized emission spectrum of **TPA** in PMMA film (1.0 wt%); Normalized delayed emission spectrum of **6a** in TPA film (1.0 wt%) (1 ms delay); Normalized emission spectrum of **6a** in TPA film (1.0 wt%).

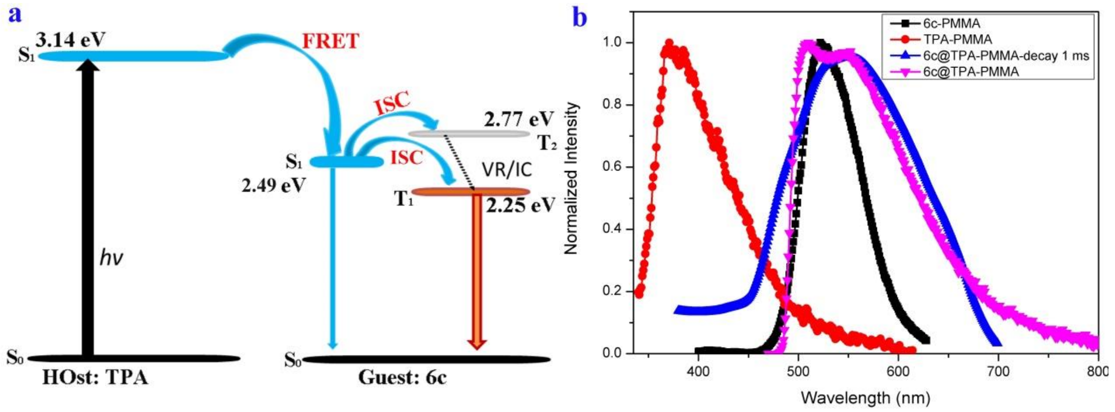

Figure S39. (a) Possible luminescent mechanism of RTP behaviour of host–guest doped system based on **6c**@TPA in PMMA film. (b) Normalized emission spectrum of **6c** in PMMA film (1.0 wt%); Normalized emission spectrum of **TPA** in PMMA film (1.0 wt%); Normalized delayed emission spectrum of **6c**@TPA in PMMA film (1.0 wt%) (1 ms delay); Normalized emission spectrum of **6c**@TPA in PMMA film (1.0 wt%).

XII Electrochemistry properties of 4a, 5a and 6a.

Prepare an electrochemical workstation, a three-electrode system (a glassy carbon electrode as the working electrode, a platinum wire electrode as the counter electrode, and an Ag/AgCl electrode as the reference electrode), a glass electrolytic cell, a magnetic stirrer, and a platinum wire stir bar. Before the experiment, polish the working electrode, ultrasonically clean it, and carry out the cyclic voltammetry activation process. Accurately weigh tetrabutylammonium chloride as the electrolyte and compounds **4a**, **5a**, and **6a** as the analytes. Then, prepare dichloromethane solutions containing 0.1 M tetrabutylammonium chloride and 1.0 $\times 10^{-4}$ M of each analyte for cyclic voltammetry measurement. After assembling the apparatus, set the scanning parameters of the electrochemical workstation, limited to a potential range of -1.3 V to +1.0 V and a scanning rate of 50 mV/s. Under a nitrogen atmosphere, perform cyclic voltammetry tests on the prepared samples and record the data.

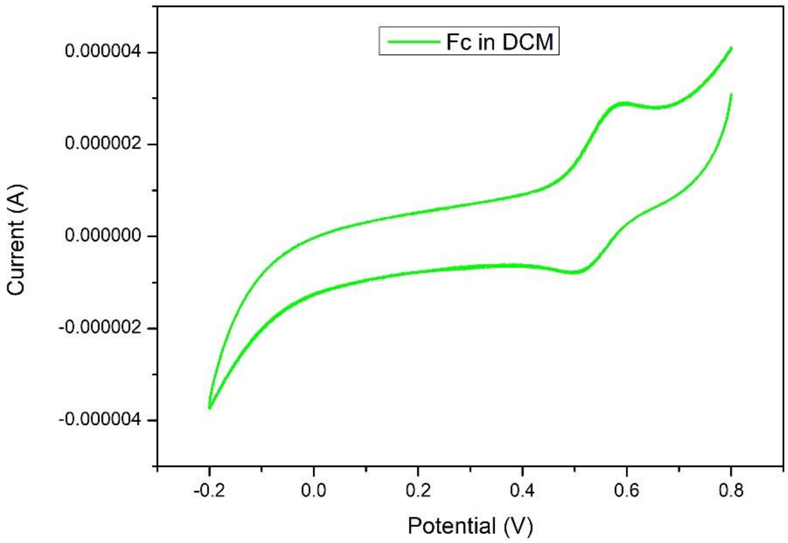

Figure S40. Cyclic voltammogram of **Fc** in CH_2Cl_2.

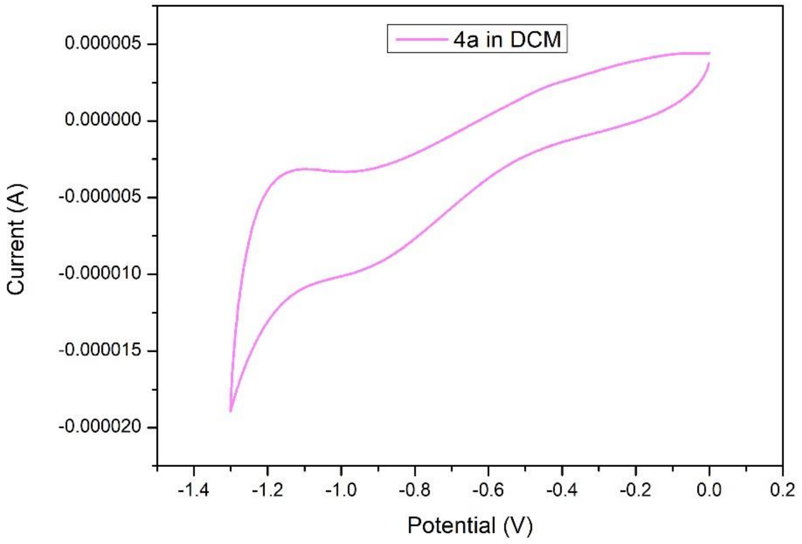

Figure S41. Cyclic voltammogram of **4a** in CH_2Cl_2.

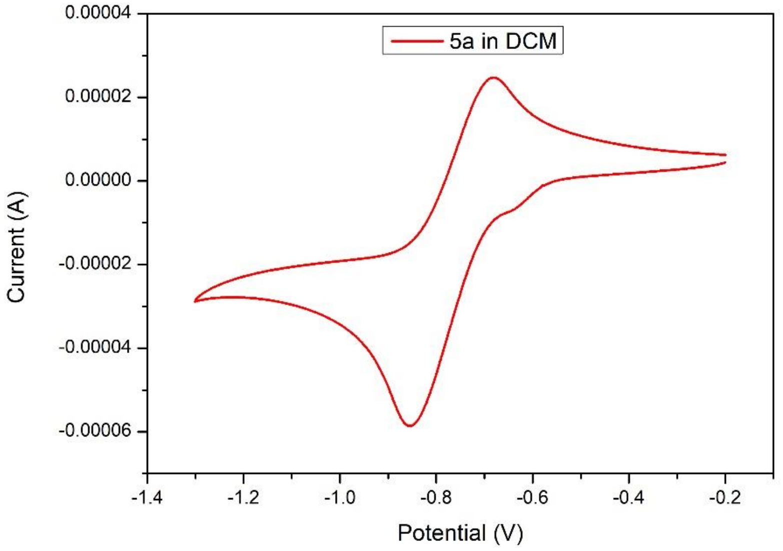

Figure S42. Cyclic voltammogram of **5a** in CH_2Cl_2.

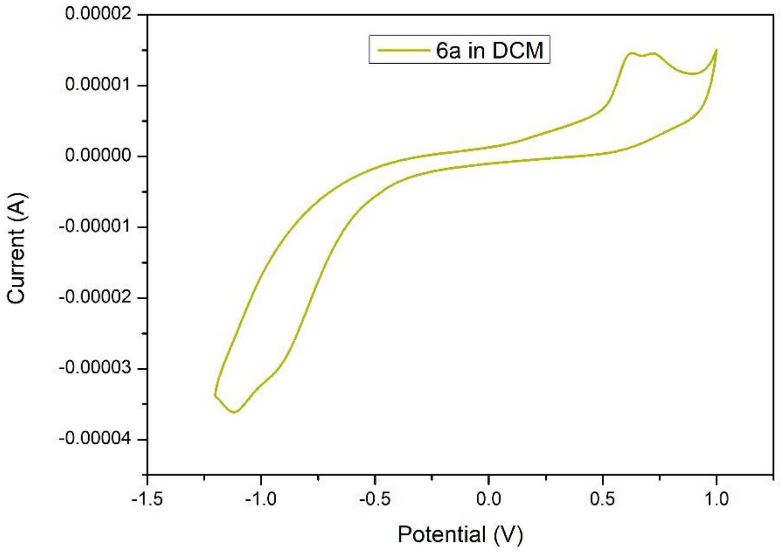

Figure S43. Cyclic voltammogram of **6a** in CH_2Cl_2.

Table S4. Energy levels of **4a**, **5a and 6a**

Compound	E_g^{opt}(eV)[a]	E^{red}(eV)[b]	HOMO (eV)[c]	LUMO (eV)[d]
**4a**	2.78	-0.93	-6.16	-3.38
**5a**	2.06	-0.84	-5.53	-3.47
**6a**	2.21	-0.92	-5.60	-3.39

[a] E_g^{opt} = 1240/λ_{onset} (eV). [b]Estimated from the onset voltages of the cyclic voltammetry in 1.0 × 10^{-4} M DCM solution. [c]HOMO = LUMO− E_g^{opt} (eV). [d]LUMO = -[E^{red}-E(Fc/Fc^+)+4.8] (eV).

XIII Experimental data for the desired products

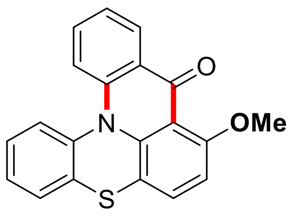

8-methoxy-9*H*-quinolino[3,2,1-*kl*]phenothiazin-9-one (3a)

Yellow solid 28.1 mg, yield: 85%. ^{1}H NMR (400 MHz, Chloroform-*d*) δ (ppm) 3.99 (s, 3H), 6.77 (d, J = 8.4 Hz, 1H), 7.11-7.20 (m, 3H), 7.32 -7.40 (m, 3H), 7.54 (t, J = 8.4 Hz, 1H), 7.67 (d, J = 8.4 Hz, 1H), 8.33 (d, J = 8.0 Hz, 1H); ^{13}C NMR (101 MHz, Chloroform-*d*) δ (ppm) 56.63, 106.49, 114.45, 115.60, 119.96, 121.89, 124.05, 126.29, 127.20, 127.39, 127.98, 128.23, 128.73, 131.37, 132.06, 140.18, 140.74, 145.32, 159.37, 178.91. HRMS (ESI^{+}): calcd for $C_{20}H_{14}NO_2S^{+}$ [M+H]$^{+}$ 332.0740, found 332.0740.

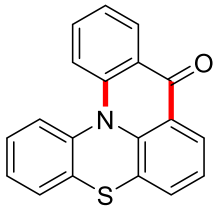

9*H*-quinolino[3,2,1-*kl*]phenothiazin-9-one (3b)

Yellow solid 28.9 mg, yield: 96%. ^{1}H NMR (400 MHz, Chloroform-*d*) δ (ppm) 7.15-7.19 (m, 2H), 7.28-7.32 (m, 2H), 7.36-7.40 (m, 2H), 7.51 (d, J = 8.4 Hz, 1H), 7.61 (t, J = 8.4 Hz, 1H), 7.82 (d, J = 8.8 Hz, 1H), 8.19 (d, J = 8.8 Hz, 1H), 8.42 (d, J = 8.8 Hz, 1H); ^{13}C NMR (101 MHz, Chloroform-d) δ (ppm) 120.63, 121.76, 124.07, 124.15, 124.38, 124.47, 124.92, 125.85, 126.31, 127.21, 127.34, 127.60, 129.15, 130.76, 132.67, 139.56, 141.53, 142.96, 179.03. HRMS (ESI^{+}): calcd for $C_{19}H_{12}NOS^{+}$ [M+H]$^{+}$ 302.0634, found 302.0631.

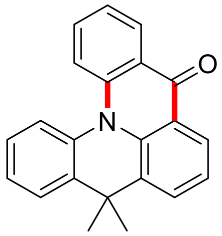

9,9-dimethylquinolino[3,2,1-*de*]acridin-5(9*H*)-one (3c)

Yellow solid 18.7 mg, yield: 60%. ^{1}H NMR (400 MHz, Chloroform-*d*) δ (ppm) 1.35 (s, 3H), 2.00 (s, 3H), 7.21-7.28 (m, 2H), 7.37-7.41 (m, 2H), 7.58-7.68 (m, 3H), 7.75 (d, J = 7.6 Hz, 1H), 8.04 (d, J = 8.4 Hz, 1H), 8.31 (d, J = 8.0 Hz, 1H), 8.51 (d, J = 8.0 Hz, 1H); ^{13}C NMR (101 MHz, Chloroform-*d*) δ (ppm) 23.17, 31.20, 37.15, 119.58, 119.97, 123.23, 123.42, 123.54, 124.56, 125.46, 125.51, 126.43, 127.58, 127.75, 132.47, 135.39, 137.35, 137.53, 139.27, 140.28, 179.47. HRMS (ESI$^+$): calcd for $C_{22}H_{18}NO^+$ [M+H]$^+$ 312.1383, found 312.1383.

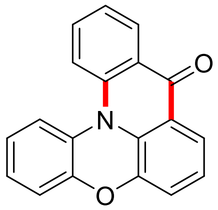

9*H*-quinolino[3,2,1-*kl*]phenoxazin-9-one (3d)

Yellow solid 15.7 mg, yield: 55%. ^{1}H NMR (400 MHz, Chloroform-*d*) δ (ppm) 7.04-7.14 (m, 3H), 7.21-7.25 (m, 2H), 7.37 (t, J = 7.4 Hz, 1H), 7.58 (d, J = 8.0 Hz, 1H), 7.66 (t, J = 7.8 Hz, 1H), 7.97 (d, J = 6.8 Hz, 1H), 8.04 (d, J = 8.4 Hz, 1H), 8.49 (d, J = 8.0 Hz, 1H); ^{13}C NMR (101 MHz, Chloroform-d) δ (ppm) 117.80, 118.24, 118.36, 118.43, 120.82, 123.45, 123.61, 123.91, 123.94, 125.02, 126.26, 128.08, 128.58,

132.77, 133.37, 138.56, 146.48, 148.46, 177.77. HRMS (ESI$^+$): calcd for $C_{19}H_{12}NO_2^+$ $[M+H]^+$ 286.0863, found 286.0867.

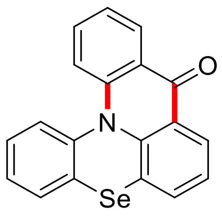

9*H*-quinolino[3,2,1-*kl*]phenoselenazin-9-one (3e)

Yellow solid 25.5 mg, yield: 73%. ^{1}H NMR (400 MHz, Chloroform-*d*) δ (ppm) 7.16-7.20 (m, 3H), 7.30 (t, J = 7.6 Hz, 1H), 7.37 (t, J = 7.6 Hz, 1H), 7.52-7.60 (m, 2H), 7.67 (d, J = 7.2 Hz, 1H), 7.71 (d, J = 8.4 Hz, 1H), 8.24 (d, J = 7.6 Hz, 1H), 8.40 (d, J = 8.0 Hz, 1H); ^{13}C NMR (101 MHz, Chloroform-d) δ (ppm) 119.96, 121.24, 123.24, 123.45, 124.01, 124.78, 125.00, 125.37, 125.97, 126.64, 127.11, 127.95, 131.56, 132.67, 133.46, 140.20, 142.73, 143.24, 179.44. HRMS (ESI$^+$): calcd for $C_{19}H_{12}NOSe^+$ $[M+H]^+$ 350.0079, found 350.0077.

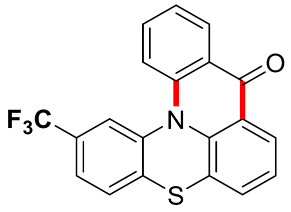

2-(trifluoromethyl)-9*H*-quinolino[3,2,1-*kl*]phenothiazin-9-one (3f)

Yellow solid 19.2 mg, yield: 52%. ^{1}H NMR (400 MHz, Chloroform-*d*) δ (ppm) 7.34 (t, J = 7.8 Hz, 1H), 7.41-7.46 (m, 2H), 7.49-7.53 (m, 2H), 7.56 (s, 1H), 7.65-7.69 (m, 1H), 7.78 (d, J = 8.8 Hz, 1H), 8.20-8.22 (m, 1H), 8.43-8.46 (m, 1H); ^{13}C NMR (101

MHz, Chloroform-*d*) *δ* (ppm) 118.37, 118.41, 119.99, 122.75, 122.78, 123.08, 124.58, 124.76, 124.99, 125.45, 126.09, 127.70, 129.56, 129.95, 130.28, 131.02, 132.18, 133.21, 140.14, 141.13, 142.58, 178.75. HRMS (ESI$^+$): calcd for $C_{20}H_{11}F_3NOS^+$ $[M+H]^+$ 370.0508, found 370.0503.

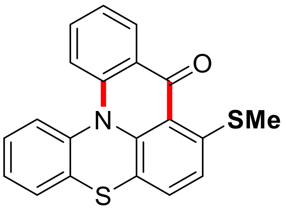

8-(methylthio)-9*H*-quinolino[3,2,1-*kl*]phenothiazin-9-one (3g)

Yellow solid 30.9 mg, yield: 89%. ^{1}H NMR (400 MHz, Chloroform-*d*) *δ* (ppm) 2.47 (s, 3H), 7.06 (d, *J* = 8.4 Hz, 1H), 7.14-7.24 (m, 3H), 7.34-7.42 (m, 3H), 7.57 (t, *J* = 7.8 Hz, 1H), 7.71 (d, *J* = 8.4 Hz, 1H), 8.37 (d, *J* = 7.6 Hz, 1H); ^{13}C NMR (101 MHz, Chloroform-*d*) *δ* (ppm) 16.15, 118.33, 119.18, 120.17, 121.58, 122.12, 124.14, 126.48, 126.66, 127.40, 127.53, 127.96, 128.94, 130.33, 132.48, 140.07, 141.01, 143.28, 145.15, 179.60. HRMS (ESI$^+$): calcd for $C_{20}H_{14}NOS_2^+$ $[M+H]^+$ 348.0511, found 348.0510.

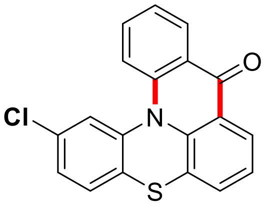

2-chloro-9*H*-quinolino[3,2,1-*kl*]phenothiazin-9-one (3h)

Yellow solid 23.1 mg, yield: 69%. ^{1}H NMR (400 MHz, Chloroform-*d*) *δ* (ppm) 7.14-7.17 (m, 1H), 7.29-7.34 (m, 3H), 7.42 (t, *J* = 7.6 Hz, 1H), 7.51 (d, *J* = 7.6 Hz, 1H),

7.67 (t, J = 7.8 Hz, 1H), 7.82 (d, J = 8.8 Hz, 1H), 8.19 (d, J = 7.6 Hz, 1H), 8.43 (d, J = 8.0 Hz, 1H); ^{13}C NMR (101 MHz, Chloroform-*d*) δ (ppm) 120.41, 121.68, 123.86, 124.55, 124.80, 125.20, 125.70, 125.98, 126.33, 127.55, 129.84, 130.93, 133.11, 133.53, 140.83, 141.16, 142.65, 178.86. HRMS (ESI$^+$): calcd for $C_{19}H_{11}ClNOS^+$ $[M+H]^+$ 336.0244, found 336.0243.

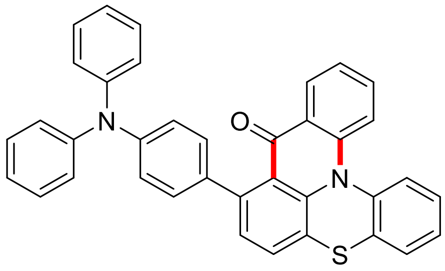

8-(4-(diphenylamino)phenyl)-9*H*-quinolino[3,2,1-*kl*]phenothiazin-9-one (3i)

Yellow solid 51.7 mg, yield: 95%. ^{1}H NMR (400 MHz, Chloroform-*d*) δ (ppm) 6.99 (t, J = 6.8 Hz, 1H), 7.07-7.44 (m, 19H), 7.50-7.54 (m, 1H), 7.68 (t, J = 8.6 Hz, 1H), 7.90 (d, J = 8.8 Hz, 1H), 8.22 (d, J = 8.0 Hz, 1H); ^{13}C NMR (101 MHz, Chloroform-*d*) δ (ppm) 118.42, 120.16, 121.10, 121.81, 122.05, 122.33, 122.85, 123.72, 124.03, 124.65, 125.36, 125.94, 126.19, 126.32, 127.01, 127.14, 127.52, 127.84, 128.75, 129.26, 129.48, 129.85, 129.97, 130.05, 132.09, 132.33, 139.15, 139.84, 140.79, 140.96, 143.88, 143.97, 145.38, 147.72, 154.03, 179.14. HRMS (ESI$^+$): calcd for $C_{37}H_{25}N_2OS^+$ $[M+H]^+$ 545.1682, found 545.1671.

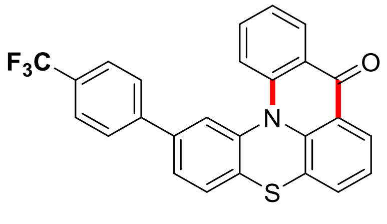

2-(4-(trifluoromethyl)phenyl)-9*H*-quinolino[3,2,1-*kl*]phenothiazin-9-one (3j)

Yellow solid 43.2 mg, yield: 97%. ^{1}H NMR (400 MHz, Chloroform-*d*) δ (ppm) 7.31-7.37 (m, 2H), 7.40-7.44 (m, 3H), 7.46 (d, J = 8.0 Hz, 2H), 7.52 (d, J = 8.8 Hz, 1H), 7.60-7.66 (m, 3H), 7.86 (d, J = 8.4 Hz, 1H), 8.21 (d, J = 9.2 Hz, 1H), 8.45 (d, J = 9.2 Hz, 1H); ^{13}C NMR (101 MHz, Chloroform-*d*) δ (ppm) 120.34, 120.43, 123.84, 124.33, 124.51, 124.69, 125.01, 125.10, 125.96, 125.99, 126.02, 127.20, 127.46, 127.54, 129.62, 130.84, 132.83, 139.41, 140.10, 141.44, 142.80, 143.07, 178.93. HRMS (ESI^{+}): calcd for $C_{26}H_{15}F_{3}NOS^{+}$ [M+H]$^{+}$ 446.0821, found 446.0816.

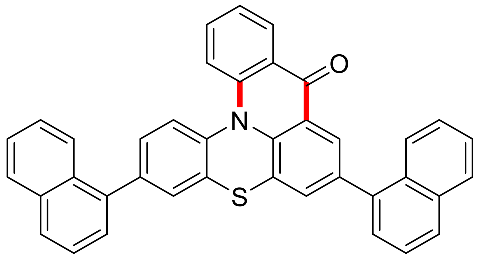

3,7-di(naphthalen-1-yl)-9*H*-quinolino[3,2,1-*kl*]phenothiazin-9-one (3k)

Yellow solid 31.5 mg, yield: 57%. ^{1}H NMR (400 MHz, Chloroform-*d*) δ (ppm) 7.29-7.32 (m, 1H), 7.38-7.54 (m, 11H), 7.63-7.66 (m, 2H), 7.84-7.99 (m, 7H), 8.37 (d, J = 2.0 Hz, 1H), 8.48 (d, J = 7.6 Hz, 1H); ^{13}C NMR (101 MHz, Chloroform-*d*) δ (ppm) 120.66, 121.47, 124.19, 124.22, 124.26, 125.48, 125.59, 125.96, 126.06, 126.11, 126.49, 126.56, 127.03, 127.37, 127.47, 128.35, 128.50, 128.55, 129.39, 130.46, 131.35, 131.39, 132.37, 132.81, 133.85, 137.36, 138.06, 138.15, 138.61, 139.13, 141.44, 142,02, 178.94. HRMS (ESI^{+}): calcd for $C_{39}H_{24}NOS^{+}$ [M+H]$^{+}$ 554.1573, found 554.1562.

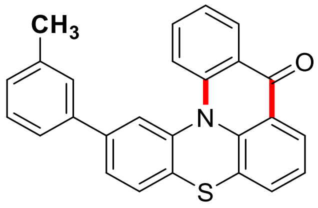

2-(m-tolyl)-9*H*-quinolino[3,2,1-*kl*]phenothiazin-9-one (3l)

Yellow solid 28.9 mg, yield: 74%. ^{1}H NMR (400 MHz, Chloroform-*d*) δ (ppm) 2.41 (s, 3H), 7.09 (d, J = 8.0 Hz, 3H), 7.17-7.23 (m, 4H), 7.28-7.33 (m, 2H), 7.38-7.42 (m, 1H), 7.49 (d, J = 7.6 Hz, 1H), 7.57 (t, J = 8.4 Hz, 1H), 7.74 (d, J = 8.4 Hz, 1H), 8.22 (d, J = 7.2 Hz, 1H); ^{13}C NMR (101 MHz, Chloroform-*d*) δ (ppm) 21.74, 120.25, 121.86, 122.46, 123.35, 124.07, 125.72, 126.31, 127.25, 127.45, 127.61, 127.86, 127.90, 127.98, 128.89, 129.08, 129.85, 132.26, 137.30, 140.27, 141.16, 141.80, 142.15, 143.90, 179.33. HRMS (ESI^{+}): calcd for $C_{26}H_{18}NOS^{+}$ [M+H]$^{+}$ 392.1104, found 392.1104.

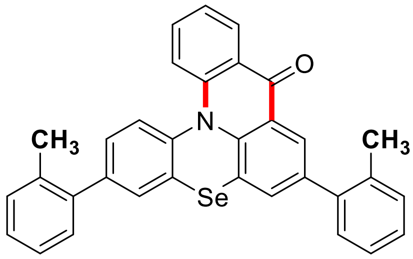

3,7-di-o-tolyl-9*H*-quinolino[3,2,1-*kl*]phenoselenazin-9-one (3m)

Yellow solid 38.1 mg, yield: 72%. ^{1}H NMR (400 MHz, Chloroform-*d*) δ (ppm) 2.32 (s, 3H), 2.33 (s, 3H), 7.18 (d, J = 10.4 Hz, 1H), 7.22-7.34 (m, 9H), 7.41 (t, J = 7.4 Hz, 1H), 7.53 (d, J = 1.6 Hz, 1H), 7.65 (t, J = 8.4 Hz, 1H), 7.70 (d, J = 1.6 Hz, 1H), 7.87 (d, J = 8.8 Hz, 1H), 8.24 (d, J = 2.0 Hz, 1H), 8.44 (d, J = 8.0 Hz, 1H); ^{13}C NMR (101 MHz, Chloroform-*d*) δ (ppm) 20.69, 119.84, 121.33, 122.75, 123.04, 124.14, 124.66,

125.76, 126.13, 127.30, 127.97, 129.02, 129.76, 129.97, 130.64, 130.66, 132.10, 132.80, 134.31, 135.43, 135.53, 138.87, 138.97, 139.91, 140.00, 140.68, 142.75, 179.49. HRMS (ESI$^+$): calcd for $C_{33}H_{24}NOSe^+$ [M+H]$^+$ 530.1018, found 530.1014.

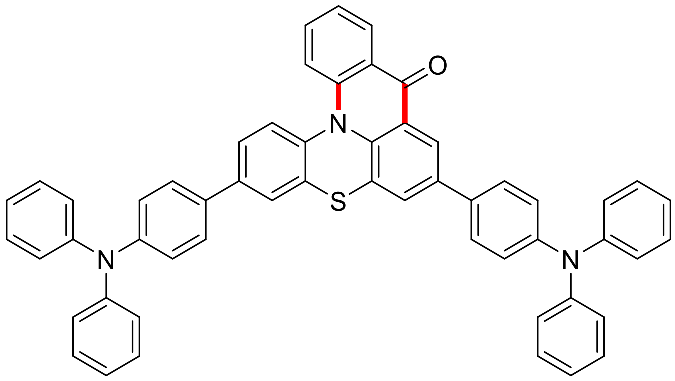

3,7-bis(4-(diphenylamino)phenyl)-9*H*-quinolino[3,2,1-*kl*]phenothiazin-9-one (3n)

Yellow solid 44.1 mg, yield: 56%. ^{1}H NMR (400 MHz, Chloroform-*d*) δ (ppm) 7.05 (t, J = 7.2 Hz, 4H), 7.13-7.15 (m, 11H), 7.26-7.30 (m, 9H), 7.38 (s, 2H), 7.38-7.46 (m, 3H), 7.54 (d, J = 8.4 Hz, 2H), 7.60 (s, 1H), 7.65 (t, J = 7.2 Hz, 1H), 7.77 (d, J = 1.8 Hz, 1H), 7.91 (d, J = 8.8 Hz, 1H), 8.40 (d, J = 1.8 Hz, 1H), 8.46 (d, J = 8.0 Hz, 1H); ^{13}C NMR (101 MHz, Chloroform-*d*) δ (ppm) 120.62, 121.95, 122.19, 123.30, 123.37, 123.67, 123.78, 124.04, 124.50, 124.66, 124.75, 124.78, 125.72, 125.84, 126.82, 127.46, 127.55, 127.71, 128.85, 129.48, 132.64, 132.72, 137.22, 138.00, 139.01, 141.40, 141.58, 147.61, 147.65, 147.84, 147.89, 178.95. HRMS (ESI$^+$): calcd for $C_{55}H_{38}N_3OS^+$ [M+H]$^+$ 788.2730, found 788.2693.

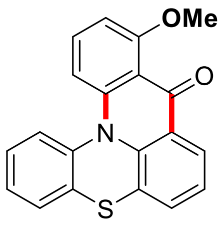

10-methoxy-9*H*-quinolino[3,2,1-*kl*]phenothiazin-9-one (4a)

Yellow solid 30.8 mg, yield: 93%. ^{1}H NMR (400 MHz, Chloroform-*d*) δ (ppm) 4.04 (s, 3H), 6.80 (d, J = 8.0 Hz, 1H), 7.12-7.18 (m, 2H), 7.23-7.30 (m, 2H), 7.34-7.36 (m, 2H), 7.43-7.48 (m, 2H), 8.09 (d, J = 8.0 Hz, 1H); ^{13}C NMR (101 MHz, Chloroform-*d*) δ (ppm) 56.51, 105.54, 112.77, 116.95, 121.99, 123.62, 124.40, 124.97, 126.10, 126.69, 127.15, 127.58, 129.06, 129.90, 132.97, 140.22, 141.50, 144.12, 160.60, 178.94. HRMS (ESI^{+}): calcd for $C_{20}H_{14}NO_{2}S^{+}$ [M+H]$^{+}$ 332.0740, found 332.0742.

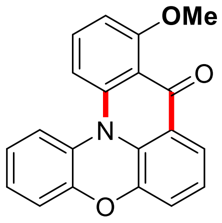

10-methoxy-9*H*-quinolino[3,2,1-*kl*]phenoxazin-9-one (4b)

Yellow solid 28.4 mg, yield: 90%. ^{1}H NMR (400 MHz, Chloroform-*d*) δ (ppm) 4.02 (s, 3H), 6.77 (d, J = 8.0 Hz, 1H), 7.00-7.04 (m, 1H), 7.08-7.18 (m, 4H), 7.47-7.56 (m, 3H), 7.86-7.88 (m, 1H); ^{13}C NMR (101 MHz, Chloroform-*d*) δ (ppm) 56.48, 104.99, 109.64, 115.78, 117.54, 118.13, 118.79, 120.73, 123.77, 125.63, 126.04, 128.79, 131.88, 133.10, 141.09, 145.93, 148.44, 161.19, 177.62. HRMS (ESI^{+}): calcd for $C_{20}H_{14}NO_{3}^{+}$ [M+H]$^{+}$ 316.0968, found 316.0974.

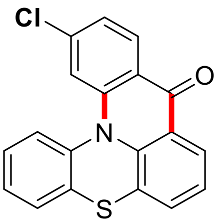

12-chloro-9*H*-quinolino[3,2,1-*kl*]phenothiazin-9-one (4c)

Yellow solid 23.8 mg, yield: 71%. ^{1}H NMR (400 MHz, Chloroform-*d*) δ (ppm) 7.20-7.25 (m, 2H), 7.33 (t, J = 8.0 Hz, 3H), 7.40-7.42 (m, 1H), 7.54 (d, J = 7.6 Hz, 1H), 7.83 (s, 1H), 8.18 (d, J = 8.0 Hz, 1H), 8.36 (d, J = 8.4 Hz, 1H); ^{13}C NMR (101 MHz, Chloroform-*d*) δ (ppm) 120.19, 121.64, 124.24, 124.30, 124.51, 124.78, 124.86, 124.93, 126.70, 127.48, 127.94, 129.08, 129.34, 131.02, 139.05, 139.22, 142.24, 142.89, 178.28. HRMS (ESI^{+}): calcd for $C_{19}H_{11}ClNOS^{+}$ [M+H]$^{+}$ 336.0244, found 336.0243.

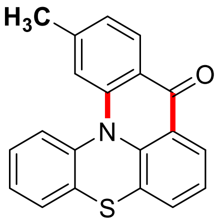

12-methyl-9*H*-quinolino[3,2,1-*kl*]phenothiazin-9-one (4d)

Yellow solid 17.0 mg, yield: 54%. ^{1}H NMR (400 MHz, Chloroform-*d*) δ (ppm) 2.45 (s, 3H), 7.17-7.21 (m, 3H), 7.27-7.34 (m, 2H), 7.38-7.40 (m, 1H), 7.50 (d, J = 7.2 Hz, 1H), 7.62 (s, 1H), 8.19 (d, J = 7.6 Hz, 1H), 8.31 (d, J = 8.4 Hz, 1H); ^{13}C NMR (101 MHz, Chloroform-*d*) δ (ppm) 22.28, 120.36, 121.86, 123.98, 124.21, 124.40, 124.56, 124.99, 125.77, 126.22, 127.38, 127.43, 127.62, 129.20, 130.63, 139.88, 141.80,

143.12, 143.77, 178.82. HRMS (ESI$^+$): calcd for $C_{20}H_{14}NOS^+$ [M+H]$^+$ 316.0791, found 316.0789.

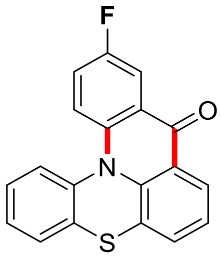

11-fluoro-9*H*-quinolino[3,2,1-*kl*]phenothiazin-9-one (4e)

Yellow solid 17.5 mg, yield: 55%. ^{1}H NMR (400 MHz, Chloroform-*d*) δ (ppm) 7.17-7.25 (m, 3H), 7.29-7.40 (m, 3H), 7.52 (d, J = 7.6 Hz, 1H), 7.81-7.85 (m, 1H), 8.03-8.06 (m, 1H), 8.17 (d, J = 8.0 Hz, 1H); ^{13}C NMR (101 MHz, Chloroform-*d*) δ (ppm) 111.83, 112.06, 121.06, 121.30, 121.51, 122.97, 123.05, 123.71, 124.26, 124.66, 124.92, 126.43, 127.12, 127.72, 129.24, 131.02, 137.92, 139.49, 142.96, 157.80, 160.25, 178.18. HRMS (ESI$^+$): calcd for $C_{19}H_{11}FNOS^+$ [M+H]$^+$ 320.0540, found 320.0537.

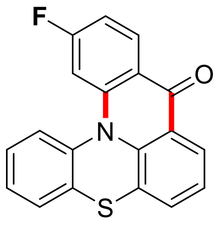

12-fluoro-9*H*-quinolino[3,2,1-*kl*]phenothiazin-9-one (4f)

Yellow solid 24.2 mg, yield: 76%. ^{1}H NMR (400 MHz, Chloroform-*d*) δ (ppm) 7.08-7.13 (m, 1H), 7.19-7.24 (m, 2H), 7.30-7.35 (m, 2H), 7.39-7.41 (m, 1H), 7.46-7.53 (m, 2H), 8.18 (d, J = 8.0 Hz, 1H), 8.42-8.46 (m, 1H); ^{13}C NMR (101 MHz, Chloroform-*d*)

δ (ppm) 106.55, 106.81, 112.80, 113.03, 121.59, 122.65, 124.13, 124.41, 124.80, 124.94, 126.68, 127.53, 127.82, 129.31, 130.43, 130.54, 130.84, 139.02, 143.10, 164.00, 166.52, 178.05. HRMS (ESI$^+$): calcd for $C_{19}H_{11}FNOS^+$ $[M+H]^+$ 320.0540, found 320.0544.

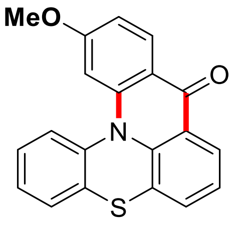

12-methoxy-9*H*-quinolino[3,2,1-*kl*]phenothiazin-9-one (4g)

Yellow solid 30.5 mg, yield: 92%. ^{1}H NMR (400 MHz, Chloroform-*d*) δ (ppm) 3.84 (s, 3H), 6.94-6.97 (m, 1H), 7.15-7.18 (m, 3H), 7.28 (t, J = 6.4 Hz, 1H), 7.37-7.39 (m, 2H), 7.47 (d, J = 7.6 Hz, 1H), 8.17 (d, J = 8.0 Hz, 1H), 8.35 (d, J = 8.8 Hz, 1H); ^{13}C NMR (101 MHz, Chloroform-*d*) δ (ppm) 55.82, 103.20, 113.14, 120.16, 121.73, 123.95, 124.40, 124.47, 124.86, 126.29, 127.51, 129.23, 129.34, 130.41, 139.65, 143.15, 143.47, 163.16, 178.09. HRMS (ESI$^+$): calcd for $C_{20}H_{14}NO_2S^+$ $[M+H]^+$ 332.0740, found 332.0739.

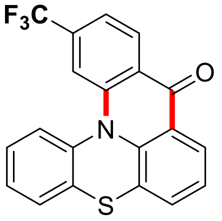

12-(trifluoromethyl)-9*H*-quinolino[3,2,1-*kl*]phenothiazin-9-one (4h)

Yellow solid 23.2 mg, yield: 63%. ^{1}H NMR (400 MHz, Chloroform-*d*) δ (ppm) 7.22-7.24 (m, 3H), 7.35 (t, J = 7.6 Hz, 1H), 7.42-7.45 (m, 1H), 7.58 (t, J = 8.2 Hz, 2H), 8.12 (s, 1H), 8.19 (d, J = 8.8 Hz, 1H), 8.54 (d, J = 8.4 Hz, 1H); ^{13}C NMR (101 MHz, Chloroform-*d*) δ (ppm) 118.23, 118.27, 118.31, 118.35, 120.06, 120.10, 121.41, 124.55, 124.71, 125.05, 125.08, 126.94, 127.60, 127.70, 128.03, 128.78, 129.50, 131.30, 134.08, 134.41, 139.08, 141.42, 143.05, 178.25. HRMS (ESI$^+$): calcd for $C_{20}H_{11}F_3NOS^+$ [M+H]$^+$ 370.0508, found 370.0505.

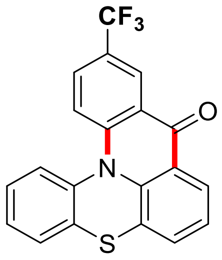

11-(trifluoromethyl)-9*H*-quinolino[3,2,1-*kl*]phenothiazin-9-one (4i)

Yellow solid 32.1 mg, yield: 87%. ^{1}H NMR (400 MHz, Chloroform-*d*) δ (ppm) 7.19-7.28 (m, 3H), 7.34 (t, J = 7.6 Hz, 1H), 7.40-7.43 (m, 1H), 7.55 (d, J = 7.6 Hz, 1H), 7.80 (d, J = 8.8 Hz, 1H), 7.93 (d, J = 8.8 Hz, 1H), 8.18 (d, J = 8.0 Hz, 1H), 8.71 (s, 1H); ^{13}C NMR (101 MHz, Chloroform-*d*) δ (ppm) 121.43, 121.79, 124.39, 124.53, 124.97, 125.12, 125.24, 125.37, 125.41, 125.45, 125.49, 125.82, 126.16, 126.93, 127.62, 127.82, 128.80, 128.83, 129.38, 131.24, 138.76, 142.74, 143.53, 178.13. HRMS (ESI$^+$): calcd for $C_{20}H_{11}F_3NOS^+$ [M+H]$^+$ 370.0508, found 370.0507.

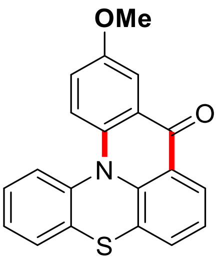

11-methoxy-9*H*-quinolino[3,2,1-*kl*]phenothiazin-9-one (4j)

Yellow solid 21.5 mg, yield: 65%. ^{1}H NMR (400 MHz, Chloroform-*d*) *δ* (ppm) 3.96 (s, 3H), 7.14-7.19 (m, 2H), 7.23-7.26 (m, 2H), 7.30 (t, *J* = 7.8 Hz, 1H), 7.37-7.39 (m, 1H), 7.51 (d, *J* = 7.2 Hz, 1H), 7.77 (d, *J* = 9.2 Hz, 1H), 7.81 (d, *J* = 2.8 Hz, 1H), 8.20 (d, *J* = 8.0 Hz, 1H); ^{13}C NMR (101 MHz, Chloroform-*d*) *δ* (ppm) 55.97, 106.29, 121.62, 122.51, 123.12, 123.72, 124.11, 124.32, 124.96, 126.12, 126.73, 126.87, 127.61, 129.11, 130.59, 135.86, 139.73, 142.90, 156.30, 178.68. HRMS (ESI^{+}): calcd for $C_{20}H_{14}NO_2S^{+}$ $[M+H]^{+}$ 332.0740, found 332.0742.

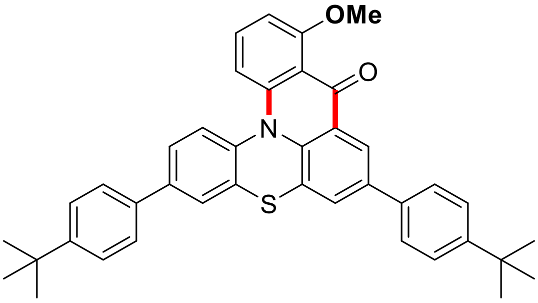

3,7-bis(4-(tert-butyl)phenyl)-10-methoxy-9*H*-quinolino[3,2,1-*kl*]phenothiazin-9-one (4k)

Yellow solid 35.7 mg, yield: 60%. ^{1}H NMR (400 MHz, Chloroform-*d*) *δ* (ppm) 1.36 (s, 18H), 4.04 (s, 3H), 6.80 (d, *J* = 8.0 Hz, 1H), 7.34-7.42 (m, 3H), 7.46-7.52 (m, 7H), 7.58 (d, *J* = 7.6 Hz, 3H), 7.71 (s, 1H), 8.34 (s, 1H); ^{13}C NMR (101 MHz, Chloroform-*d*) *δ* (ppm) 31.45, 34.71, 56.51, 105.53, 112.75, 116.87, 122.14, 122.78, 124.05,

126.03, 126.15, 126.56, 126.60, 126.72, 127.16, 127.27, 128.34, 132.99, 136.34, 136.38, 137.43, 138.88, 139.17, 140.34, 143.95, 150.90, 151.04, 160.68, 178.91. HRMS (ESI$^+$): calcd for $C_{40}H_{38}NO_2S^+$ [M+H]$^+$ 596.2618, found 596.2614.

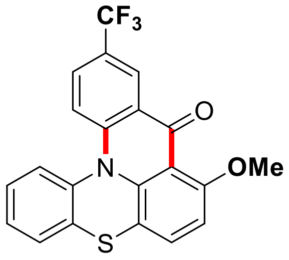

8-methoxy-11-(trifluoromethyl)-9*H*-quinolino[3,2,1-*kl*]phenothiazin-9-one (4l)

Yellow solid 23.1 mg, yield: 58%. ^{1}H NMR (400 MHz, Chloroform-*d*) δ (ppm) 3.97 (s, 3H), 6.76 (d, J = 8.8 Hz, 1H), 7.07-7.15 (m, 3H), 7.28-7.32 (m, 2H), 7.71 (s, 2H), 8.59 (s, 1H); ^{13}C NMR (101 MHz, Chloroform-*d*) δ (ppm) 56.43, 106.88, 114.18, 115.39, 120.56, 121.60, 124.97, 125.12, 125.42, 125.76, 126.65, 127.30, 128.07, 128.65, 131.68, 138.93, 142.61, 144.72, 159.08, 177.40. HRMS (ESI$^+$): calcd for $C_{21}H_{13}F_3NO_2S^+$ [M+H]$^+$ 400.0614, found 400.0613.

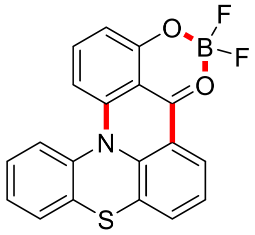

2,2-difluoro-2*H*-2λ^4,3λ^3-[1,3,2]dioxaborinino[6',5',4':4,5]quinolino[3,2,1-*kl*]phenothiazine (5a)

Red solid 31.4 mg, yield: 86%. ^{1}H NMR (400 MHz, Chloroform-*d*) δ (ppm) 7.03 (d, J = 8.0 Hz, 1H), 7.29-7.36 (m, 2H), 7.47-7.53 (m, 4H), 7.74 (d, J = 7.2 Hz, 1H), 7.83 (t,

J = 8.4 Hz, 1H), 8.40 (d, J = 8.0 Hz, 1H); ^{13}C NMR (101 MHz, Chloroform-*d*) δ (ppm) 109.19, 112.12, 118.09, 123.57, 124.07, 124.28, 126.02, 127.30, 128.33, 128.52, 129.66, 131.36, 133.33, 136.85, 140.13, 141.68, 143.96. HRMS (ESI$^+$): calcd for $C_{19}H_{10}BF_2NNaO_2S^+$ [M+Na]$^+$ 388.0386, found 386.0388.

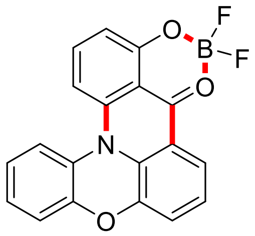

2,2-difluoro-2*H*-2λ^4,3λ^3-[1,3,2]dioxaborinino[6',5',4':4,5]quinolino[3,2,1-*kl*]phenoxazine (5b)

Red solid 31.8 mg, yield: 91%. ^{1}H NMR (400 MHz, Chloroform-*d*) δ (ppm) 7.01 (d, J = 8.0 Hz, 1H), 7.18-7.25 (m, 2H), 7.30-7.34 (m, 1H), 7.41-7.48 (m, 2H), 7.67 (d, J = 8.8 Hz, 1H), 7.79 (d, J = 8.0 Hz, 1H), 7.88 (t, J = 8.4 Hz, 1H), 8.14 (d, J = 7.6 Hz, 1H); ^{13}C NMR (101 MHz, Chloroform-*d*) δ (ppm) 106.85, 111.79, 117.38, 118.86, 119.80, 119.87, 120.47, 124.66, 125.93, 126.22, 128.98, 134.09, 138.46, 140.33, 145.59, 148.14, 159.97, 173.01. HRMS (ESI$^+$): calcd for $C_{19}H_{10}BF_2NNaO_3^+$ [M+Na]$^+$ 372.0614, found 372.0615.

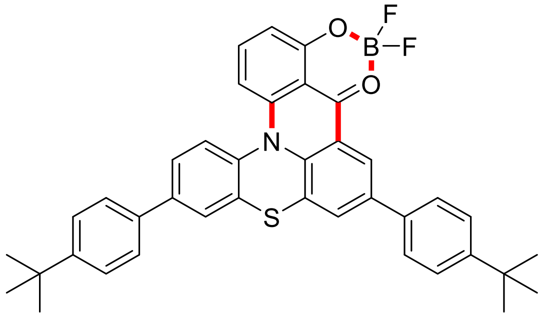

5,9-bis(4-(tert-butyl)phenyl)-2,2-difluoro-2*H*-2λ^4,3λ^3-[1,3,2]dioxaborinino[6',5',4':4,5]quinolino[3,2,1-*kl*]phenothiazine (5c)

Red solid 53.5 mg, yield: 85%. ^{1}H NMR (400 MHz, Chloroform-*d*) δ (ppm) 1.38 (s, 18H), 7.04 (d, J = 8.4 Hz, 1H), 7.50-7.57 (m, 9H), 7.61-7.66 (m, 3H), 7.84 (t, J = 8.4 Hz, 1H), 7.99 (d, J = 1.6 Hz, 1H), 8.56 (d, J = 2.0 Hz, 1H); ^{13}C NMR (101 MHz, Chloroform-*d*) δ (ppm) 31.44, 34.85, 109.24, 112.05, 118.35, 120.92, 123.77, 124.72, 126.27, 126.41, 126.72, 126.75, 126.88, 127.51, 127.58, 132.08, 134.79, 135.46, 135.56, 139.32, 139.95, 141.43, 141.63, 142.63, 151.93, 152.21, 159.74, 174.41. HRMS (ESI$^+$): calcd for $C_{39}H_{34}BF_2NNaO_2S^+$ $[M+Na]^+$ 652.2264, found 652.2265.

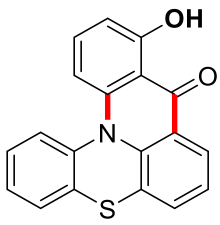

10-hydroxy-9*H*-quinolino[3,2,1-*kl*]phenothiazin-9-one (4aa)

Yellow solid 29.2 mg, yield: 92%. ^{1}H NMR (400 MHz, Chloroform-*d*) δ (ppm) 6.72 (d, J = 8.0 Hz, 1H), 7.10-7.18 (m, 3H), 7.23 (t, J = 7.6 Hz, 1H), 7.29-7.34 (m, 2H), 7.39-7.46 (m, 2H), 8.08 (d, J = 8.0 Hz, 1H), 13.23 (s, 1H); ^{13}C NMR (101 MHz, Chloroform-*d*) δ (ppm) 110.10, 110.34, 112.76, 122.33, 123.10, 124.24, 124.35, 124.60, 126.66, 127.28, 127.69, 129.25, 131.34, 135.15, 139.48, 142.37, 143.22, 162.58, 183.40. HRMS (ESI$^+$): calcd for $C_{19}H_{12}NO_2S^+$ $[M+H]^+$ 318.0583, found 318.0590.

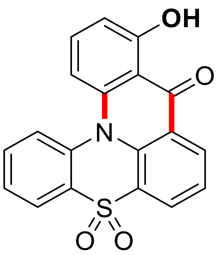

10-hydroxy-9*H*-quinolino[3,2,1-*kl*]phenothiazin-9-one 5,5-dioxide (4ab)

Yellow solid 31.4 mg, yield: 90%. ^{1}H NMR (400 MHz, Chloroform-*d*) δ (ppm) 6.95 (d, J = 8.0 Hz, 1H), 7.36 (d, J = 8.4 Hz, 1H), 7.52-7.68 (m, 4H), 7.93 (d, J = 8.0 Hz, 1H), 8.23 (d, J = 7.2 Hz, 1H), 8.42-8.47 (m, 1H), 8.65 (d, J = 7.2 Hz, 1H), 12.98 (s, 1H); ^{13}C NMR (101 MHz, Chloroform-*d*) δ (ppm) 111.08, 112.69, 122.34, 123.37, 124.12, 124.74, 126.64, 126.78, 129.32, 129.96, 131.60, 132.84, 136.10, 138.87, 140.00, 140.82, 162.87, 182.46. HRMS (ESI^{+}): calcd for $C_{19}H_{12}NO_{4}S^{+}$ [M+H]$^{+}$ 350.0482, found 350.0486.

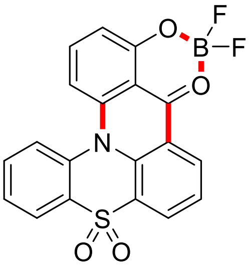

2,2-difluoro-2*H*-2λ^{4},3λ^{3}-[1,3,2]dioxaborinino[6',5',4':4,5]quinolino[3,2,1-*kl*]phenothiazine 7,7-dioxide (6a)

Yellow solid 37.7 mg, yield: 95%. ^{1}H NMR (400 MHz, Chloroform-*d*) δ (ppm) 7.17 (d, J = 8.0 Hz, 1H), 7.62 (d, J = 8.4 Hz, 1H), 7.67-7.75 (m, 2H), 7.88 (t, J = 7.8 Hz, 1H), 7.95-8.03 (m, 2H), 8.32 (d, J = 8.8 Hz, 1H), 8.68 (d, J = 7.6 Hz, 1H), 8.93 (d, J = 7.2 Hz, 1H); ^{13}C NMR (101 MHz, Chloroform-*d*) δ (ppm) 110.31, 114.43, 118.05,

123.20, 125.39, 125.50, 127.35, 128.57, 130.71, 132.22, 132.84, 133.58, 138.54, 139.00, 140.52, 141.77, 143.99, 161.08, 175.52. HRMS (ESI$^+$): calcd for $C_{19}H_{10}BF_2NNaO_4S^+$ [M+Na]$^+$ 420.0284, found 420.0287.

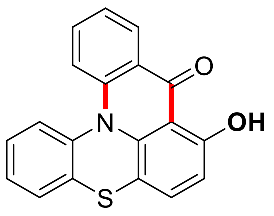

8-hydroxy-9*H*-quinolino[3,2,1-*kl*]phenothiazin-9-one (3aa)

Yellow solid 30.1 mg, yield: 95%. ^{1}H NMR (400 MHz, Chloroform-*d*) δ (ppm) 6.78 (d, J = 8.4 Hz, 1H), 7.14-7.21 (m, 2H), 7.25 (d, J = 8.4 Hz, 1H), 7.36-7.41 (m, 3H), 7.64 (t, J = 7.8 Hz, 1H), 7.81 (d, J = 8.4 Hz, 1H), 8.39 (d, J = 8.0 Hz, 1H), 13.12 (s, 1H); ^{13}C NMR (101 MHz, Chloroform-*d*) δ (ppm) 110.72, 111.36, 111.67, 120.65, 122.01, 124.18, 124.56, 126.74, 126.83, 127.36, 128.47, 129.27, 133.40, 133.54, 139.40, 141.84, 143.98, 161.00, 183.24. HRMS (ESI$^+$): calcd for $C_{19}H_{12}NO_2S^+$ [M+H]$^+$ 318.0583, found 318.0587.

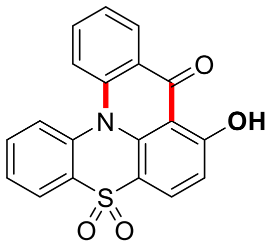

8-hydroxy-9*H*-quinolino[3,2,1-*kl]*phenothiazin-9-one 5,5-dioxide (3ab)

Yellow solid 32.5 mg, yield: 93%. ^{1}H NMR (400 MHz, Chloroform-*d*) δ (ppm) 7.04 (d, J = 8.8 Hz, 1H), 7.53-7.60 (m, 3H), 7.75-7.82 (m, 2H), 7.98 (d, J = 8.4 Hz, 1H),

8.21-8.27 (m, 2H), 8.49 (d, *J* = 7.6 Hz, 1H), 13.97 (s, 1H); ^{13}C NMR (101 MHz, Chloroform-*d*) *δ* (ppm) 109.65, 112.37, 115.75, 121.38, 122.26, 124.33, 124.76, 125.86, 126.82, 127.23, 130.74, 131.60, 132.25, 134.26, 139.60, 140.07, 140.31, 167.07, 182.81. HRMS (ESI$^+$): calcd for $C_{19}H_{12}NO_4S^+$ [M+H]$^+$ 350.0482, found 350.0480.

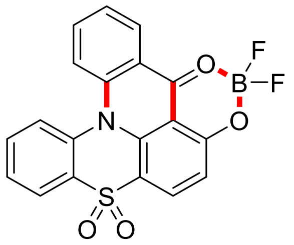

6,6-difluoro-6*H*-5λ^3,6λ^4-benzo[5,6][1,4]thiazino[2,3,4-*de*][1,3,2]dioxaborinino[6,5,4-*mn*]acridine 10,10-dioxide (6b)

Yellow solid 37.3 mg, yield: 94%. ^{1}H NMR (400 MHz, Chloroform-*d*) *δ* (ppm) 7.23 (d, *J* = 8.8 Hz, 1H), 7.68-7.74 (m, 2H), 7.79 (t, *J* = 7.6 Hz, 1H), 7.86-7.89 (m, 1H), 8.07 (t, *J* = 7.8 Hz, 1H), 8.30-8.34 (m, 2H), 8.56 (d, *J* = 8.8 Hz, 1H), 8.78 (d, *J* = 8.4 Hz, 1H); ^{13}C NMR (101 MHz, Chloroform-*d*) *δ* (ppm) 114.07, 115.70, 119.43, 121.21, 123.37, 125.04, 127.16, 127.65, 128.76, 131.65, 132.79, 136.72, 137.53, 138.07, 138.69, 141.52; HRMS (ESI$^+$): calcd for $C_{19}H_{10}BF_2NNaO_4S^+$ [M+Na]$^+$ 420.0284, found 420.0286.

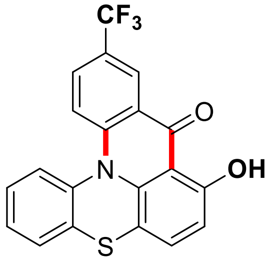

8-methoxy-11-(trifluoromethyl)-9*H*-quinolino[3,2,1-*kl*]phenothiazin-9-one 5,5-dioxide (4ma)

Yellow solid 37.0 mg, yield: 96%. ^{1}H NMR (400 MHz, Chloroform-*d*) δ (ppm) 6.83 (d, J = 8.4 Hz, 1H), 7.22-7.25 (m, 3H), 7.41-7.43 (m, 2H), 7.83 (d, J = 8.4 Hz, 1H), 7.92 (d, J = 8.8 Hz, 1H), 8.68 (s, 1H), 12.81 (s, 1H); ^{13}C NMR (101 MHz, Chloroform-*d*) δ (ppm) 111.13, 111.92, 112.17, 121.47, 122.02, 124.03, 124.89, 127.42, 127.60, 128.89, 129.52, 134.12, 138.65, 143.65, 143.80, 160.90, 182.42. HRMS (ESI^{+}): calcd for $C_{20}H_{11}F_{3}NO_{2}S^{+}$ [M+H]$^{+}$ 386.0457, found 386.0433.

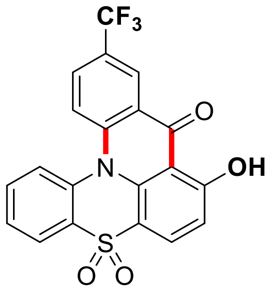

8-hydroxy-11-(trifluoromethyl)-9*H*-quinolino[3,2,1-*kl*]phenothiazin-9-one 5,5-dioxide (4mb)

Yellow solid 39.6 mg, yield: 95%. ^{1}H NMR (400 MHz, Chloroform-*d*) δ (ppm) 7.09 (d, J = 8.8 Hz, 1H), 7.59-7.64 (m, 2H), 7.52-7.77 (m, 1H), 7.97 (d, J = 8.8 Hz, 1H), 8.12 (d, J = 8.8 Hz, 1H), 8.24-8.30 (m, 2H), 8.78 (s, 1H), 13.64 (s, 1H); ^{13}C NMR (101 MHz, Chloroform-*d*) δ (ppm) 109.84, 113.04, 116.29, 122.14, 122.29, 124.48, 124.65, 125.18, 127.47, 130.45, 131.17, 132.13, 132.54, 139.15, 140.07, 142.33, 166.90, 181.93. HRMS (ESI^{+}): calcd for $C_{20}H_{11}F_{3}NO_{4}S^{+}$ [M+H]$^{+}$ 418.0355, found 418.0360.

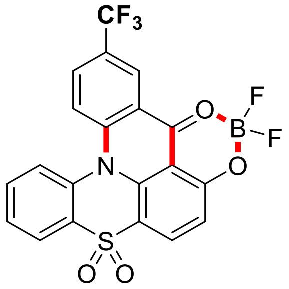

6,6-difluoro-3-(trifluoromethyl)-6*H*-5λ^{3},6λ^{4}-benzo[5,6][1,4]thiazino[2,3,4-*de*][1,3,2]dioxaborinino[6,5,4-*mn*]acridine 10,10-dioxide (6c)

Yellow solid 44.2 mg, yield: 95%. ^{1}H NMR (400 MHz, Chloroform-*d*) *δ* (ppm) 7.29 (d, *J* = 9.2 Hz, 1H), 7.72-7.79 (m, 2H), 7.83 (d, *J* = 8.4 Hz, 1H), 8.24 (d, *J* = 8.8 Hz, 1H), 8.36 (d, *J* = 8.8 Hz, 1H), 8.44 (d, *J* = 8.8 Hz, 1H), 8.61 (d, *J* = 8.8 Hz, 1H), 9.06 (s, 1H); ^{13}C NMR (101 MHz, Chloroform-*d*) *δ* (ppm) 113.04, 114.83, 122.15, 122.30, 122.36, 123.26, 124.48, 124.65, 125.13, 125.36, 127.47, 129.34, 130.42, 132.12, 132.55, 133.07, 137.53, 166.90; HRMS (ESI^{+}): calcd for $C_{20}H_{9}BF_{5}NNaO_{4}S^{+}$ [M+Na]$^{+}$ 488.0158, found 488.0160.

XIV References.

[1] M. J. Frisch, G. W. Trucks, H. B. Schlegel, G. E. Scuseria, M. A. Robb, J. R. Cheeseman, G. Scalmani, V. Barone, G. A. Petersson, H. Nakatsuji, X. Li, M. Caricato, A. V. Marenich, J. Bloino, B. G. Janesko, R. Gomperts, B. Mennucci, H. P. Hratchian, J. V. Ortiz, A. F. Izmaylov, J. L. Sonnenberg, D. Williams-Young, F. Ding, F. Lipparini, F. Egidi, J. Goings, B. Peng, A. Petrone, T. Henderson, D. Ranasinghe, V. G. Zakrzewski, J. Gao, N. Rega, G. Zheng, W. Liang, M. Hada, M. Ehara, K. Toyota, R. Fukuda, J. Hasegawa, M. Ishida, T. Nakajima, Y. Honda, O. Kitao, H. Nakai, T. Vreven, K. Throssell, J. A. Montgomery, Jr., J. E. Peralta, F. Ogliaro, M. J. Bearpark, J. J. Heyd, E. N. Brothers, K. N. Kudin, V. N. Staroverov, T. A. Keith, R. Kobayashi, J. Normand, K. Raghavachari, A. P. Rendell, J. C. Burant, S. S. Iyengar, J. Tomasi, M. Cossi, J. M. Millam, M. Klene, C. Adamo, R. Cammi, J. W. Ochterski, R. L. Martin, K. Morokuma, O. Farkas, J. B. Foresman, and D. J. Fox, Gaussian 16, Revision A.03, Gaussian, Inc., Wallingford CT, **2016**.

[2] I. M. Khan, F. Naaz, S. Shakya, M. Islam, A. Khan and M. Ahmad, *J. Mol. Liq.* **2024**, *399*, 14.

XV Copies of ^{1}H and ^{13}C NMR spectra.

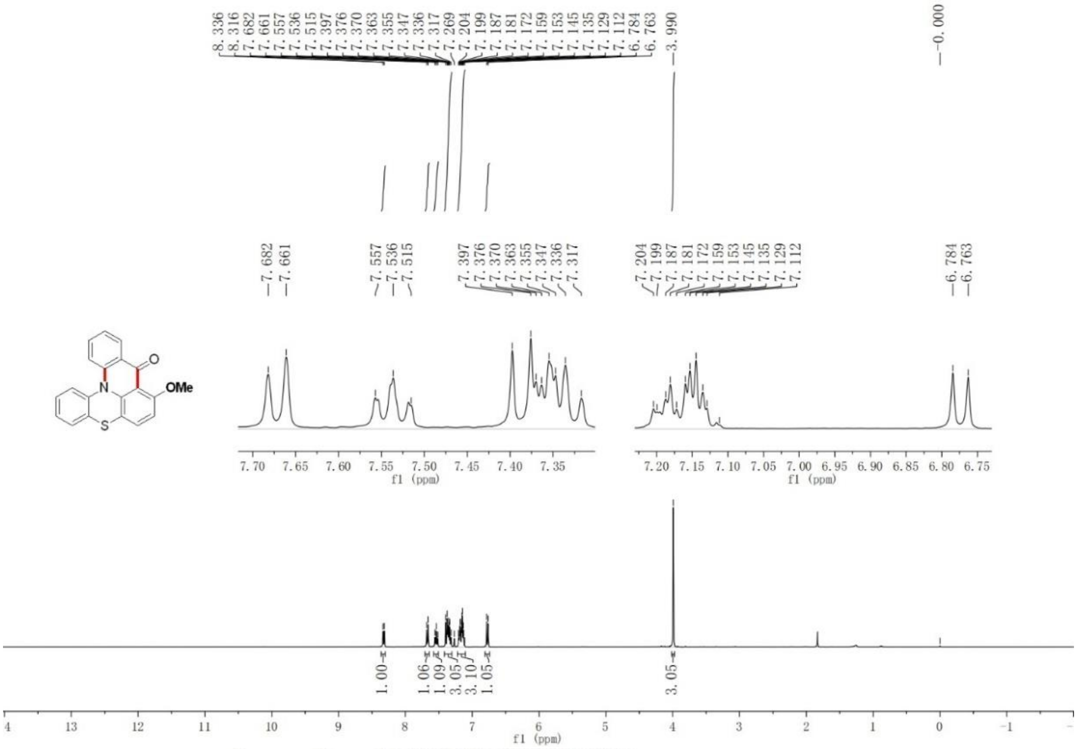

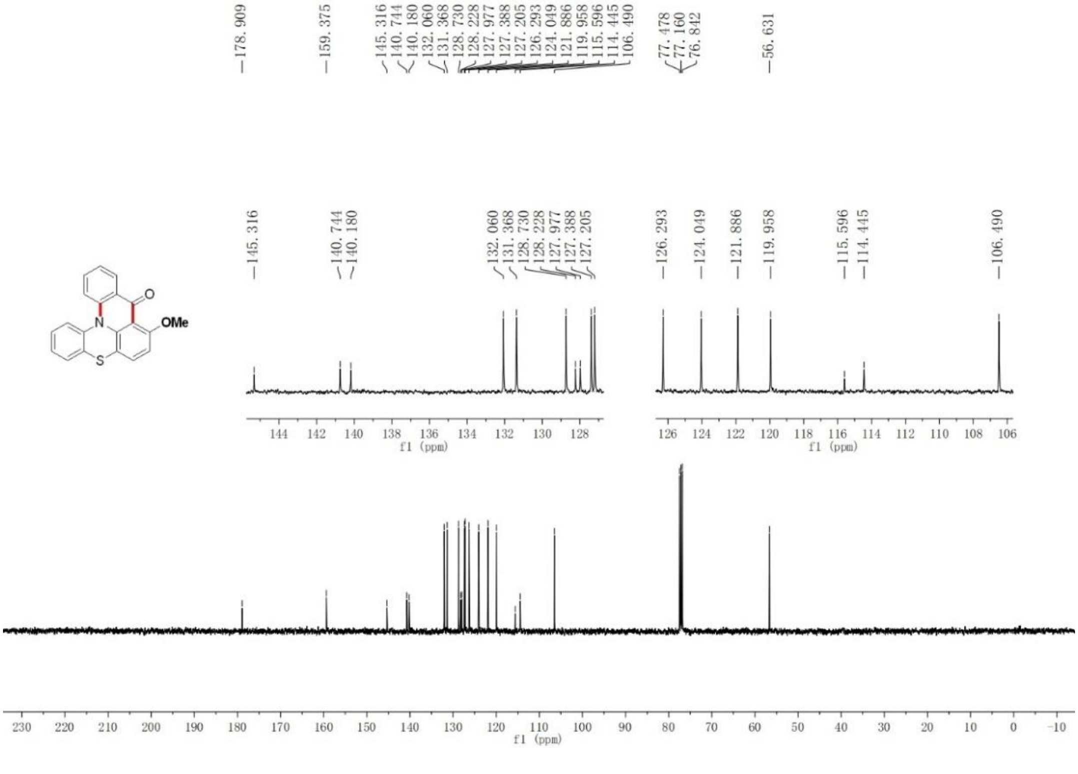

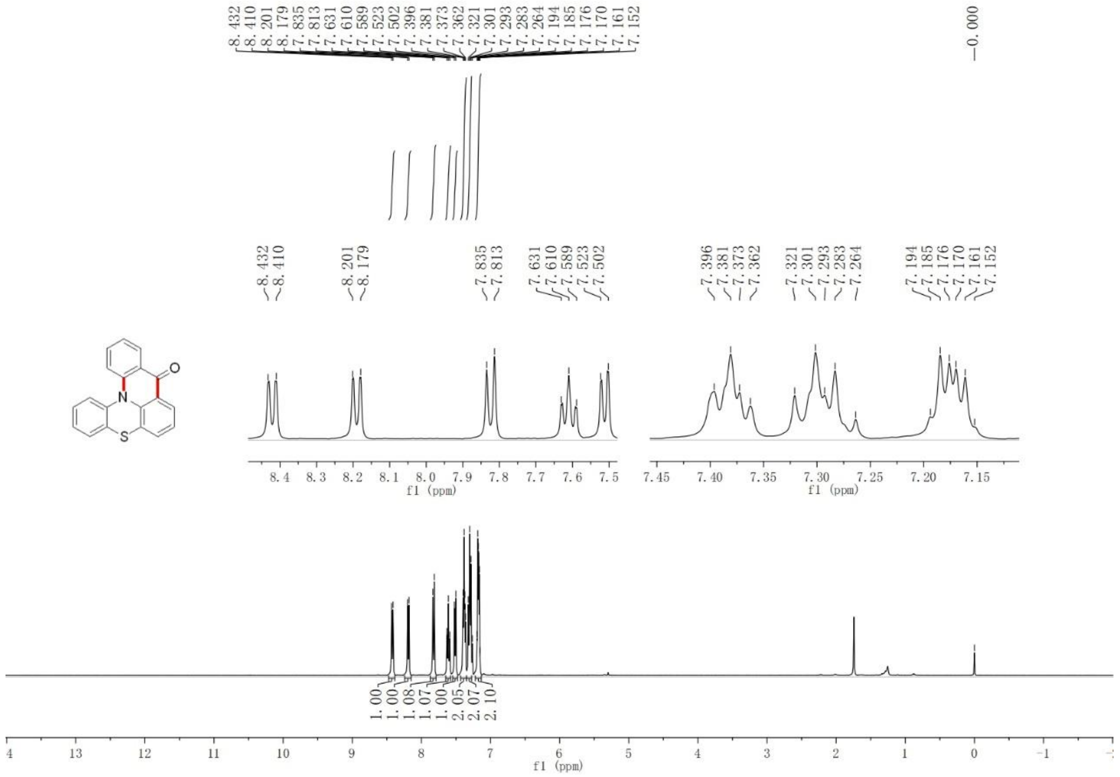
8.432
8.410
8.201
8.179
7.835
7.813
7.631
7.610
7.589
7.523
7.502
7.396
7.381
7.373
7.362
7.321
7.301
7.293
7.283
7.264
7.194
7.185
7.176
7.170
7.161
7.152
0.000
N
O
S
f1 (ppm)
1.00
1.00
1.08
1.07
1.00
2.05
2.07
2.10

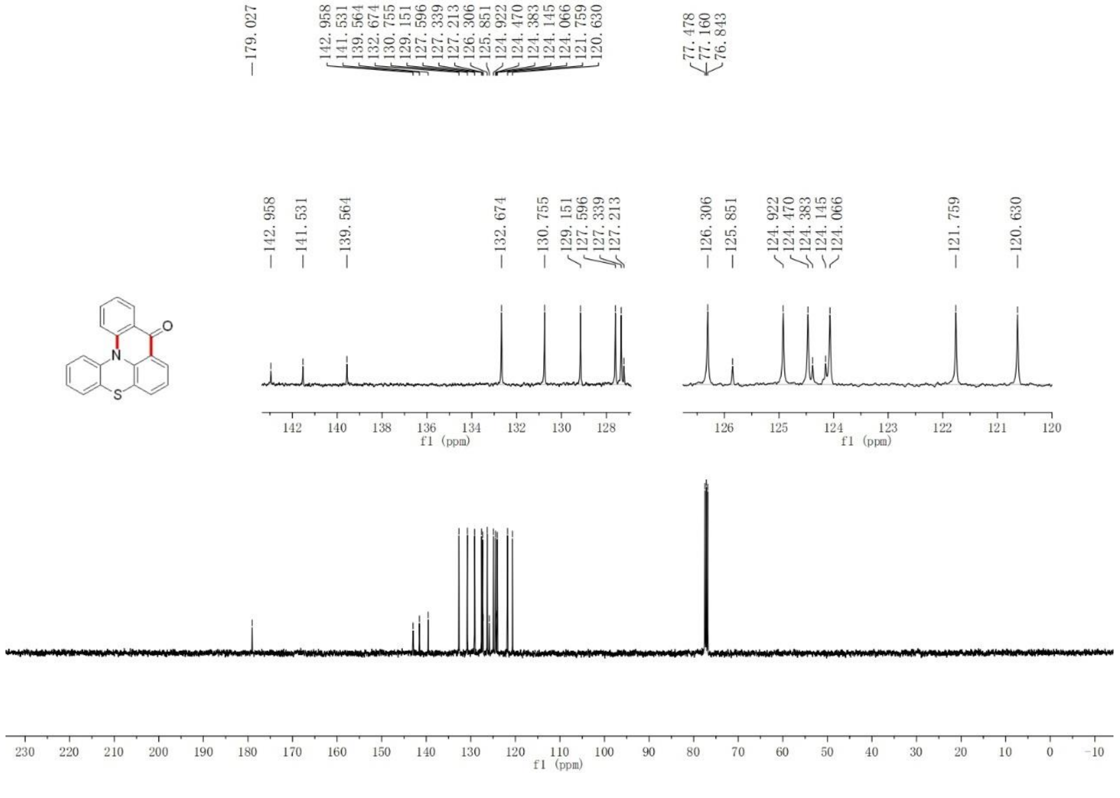
179.027
142.958
141.531
139.564
132.674
130.755
129.151
127.596
127.339
127.213
126.306
125.851
124.922
124.470
124.383
124.145
124.066
121.759
120.630
77.478
77.160
76.843
N
O
S
f1 (ppm)

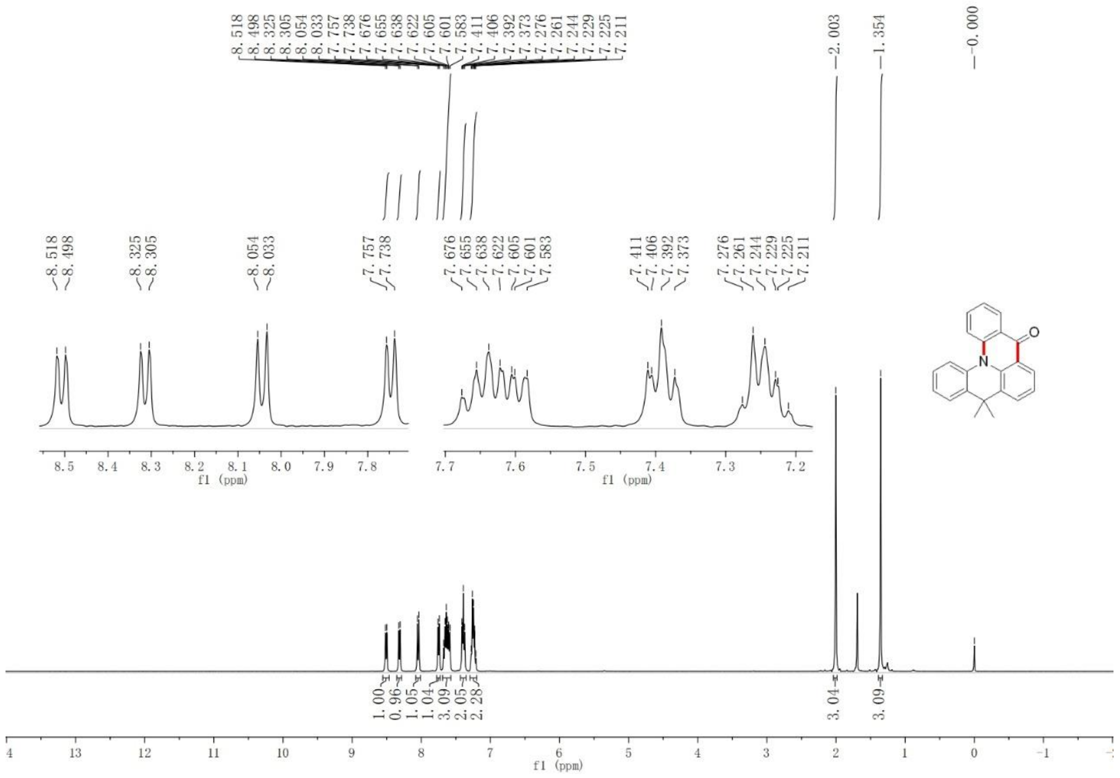
8.518 8.498 8.325 8.305 8.054 8.033 7.757 7.738 7.676 7.655 7.638 7.622 7.605 7.601 7.583 7.411 7.406 7.392 7.373 7.276 7.261 7.244 7.229 7.225 7.211
2.003
1.354
0.000
N
O
1.00 0.96 1.05 1.04 3.09 2.05 2.28
3.04 3.09
f1 (ppm)

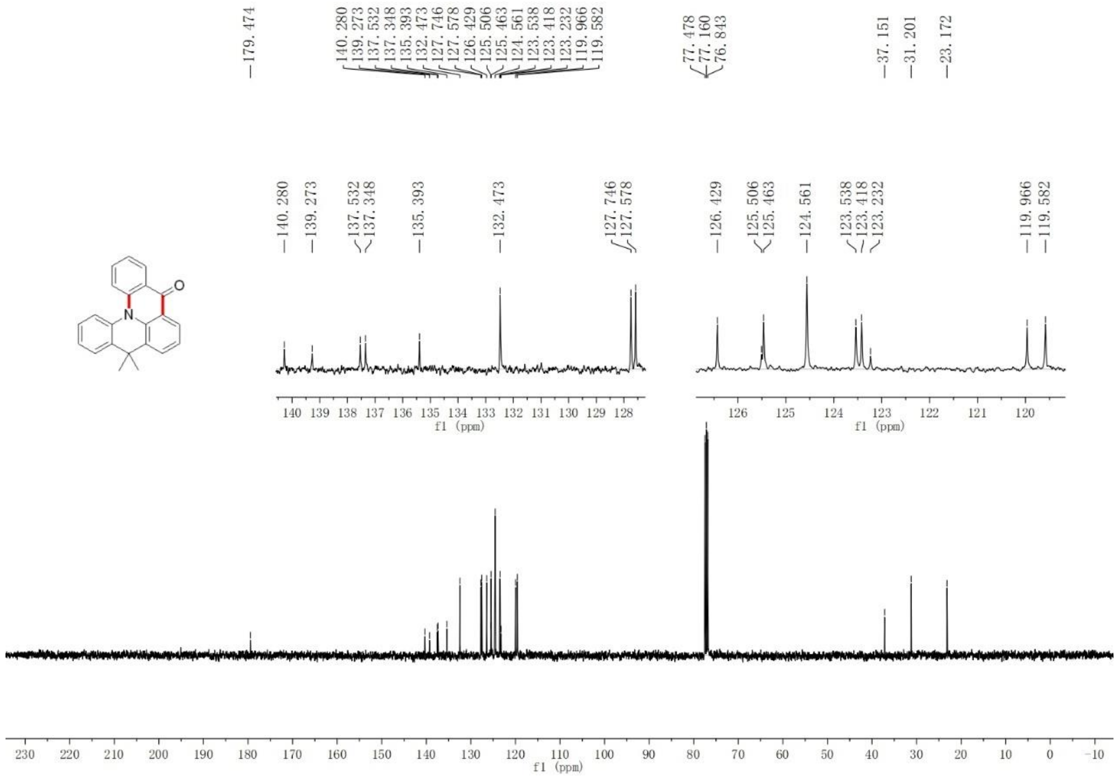
179.474
140.280 139.273 137.532 137.348 135.393 132.473 127.746 127.578 126.429 125.506 125.463 124.561 123.538 123.418 123.232 119.966 119.582
77.478 77.160 76.843
37.151
31.201
23.172
N
O
f1 (ppm)

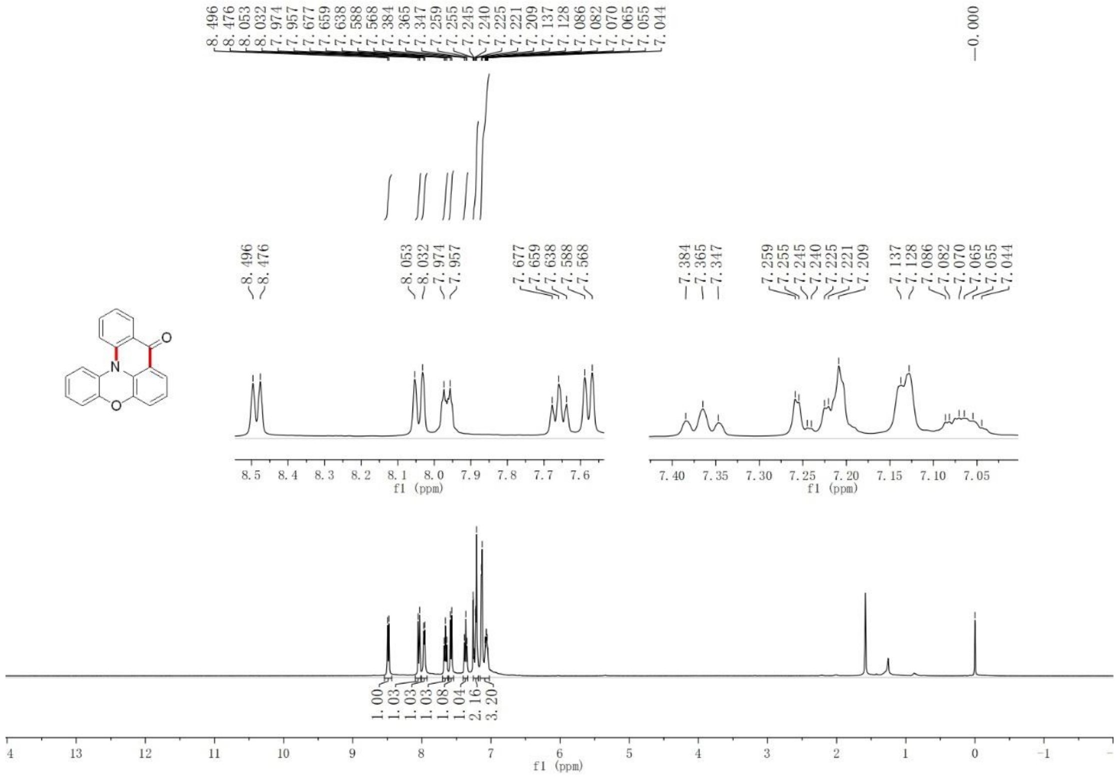
8.496 8.476 8.053 8.032 7.974 7.957 7.677 7.659 7.638 7.588 7.568 7.384 7.365 7.347 7.259 7.255 7.245 7.240 7.225 7.221 7.209 7.137 7.128 7.086 7.082 7.070 7.065 7.055 7.044
0.000
1.00 1.03 1.03 1.03 1.08 1.04 2.16 3.20
f1 (ppm)

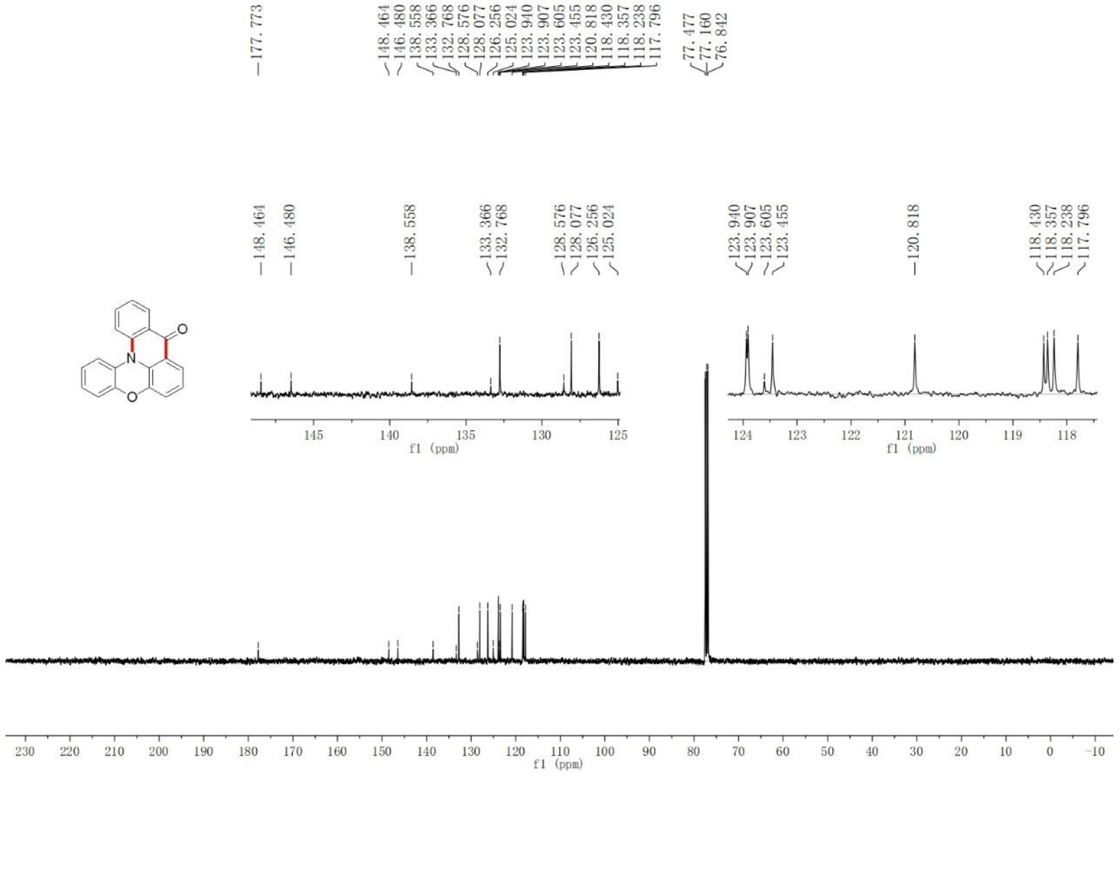
177.773
148.464 146.480 138.558 133.366 132.768 128.576 128.077 126.256 125.024 123.940 123.907 123.605 123.455 120.818 118.430 118.357 118.238 117.796
77.477 77.160 76.842
f1 (ppm)

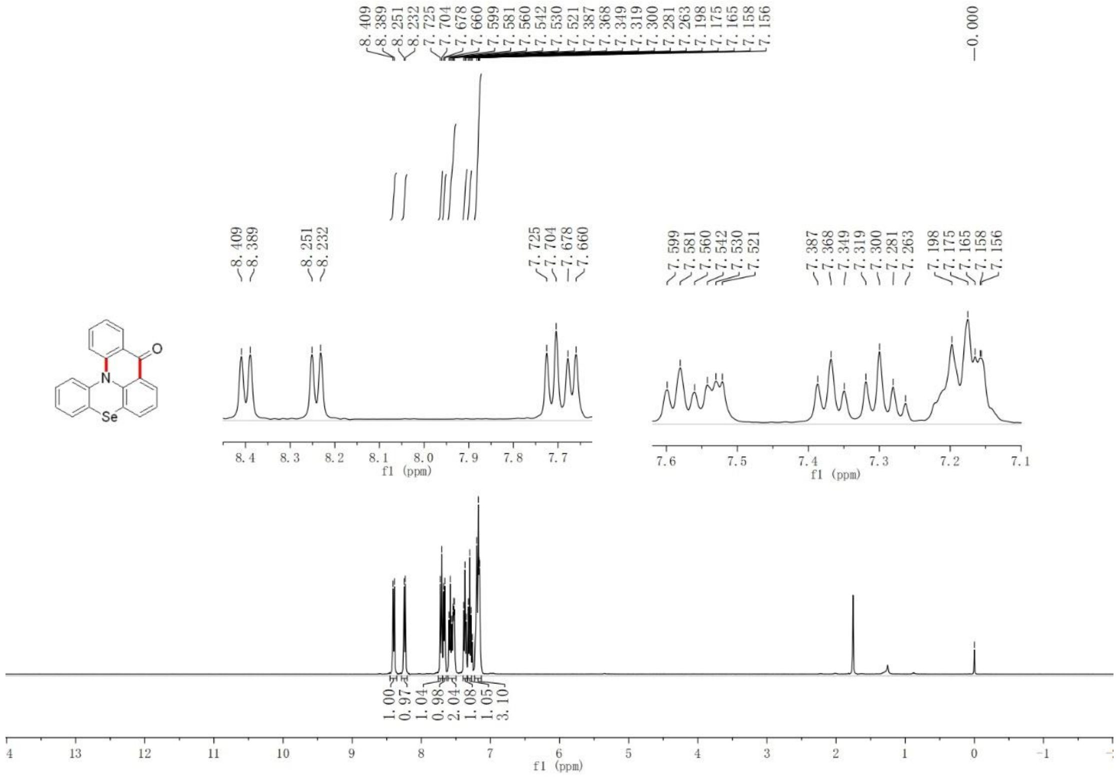
8.409 8.389 8.251 8.232 7.725 7.704 7.678 7.660 7.599 7.581 7.560 7.542 7.530 7.521 7.387 7.368 7.349 7.319 7.300 7.281 7.263 7.198 7.175 7.165 7.158 7.156
0.000
Se
N
O
1.00 0.97 1.04 0.98 2.04 1.08 1.05 3.10
f1 (ppm)

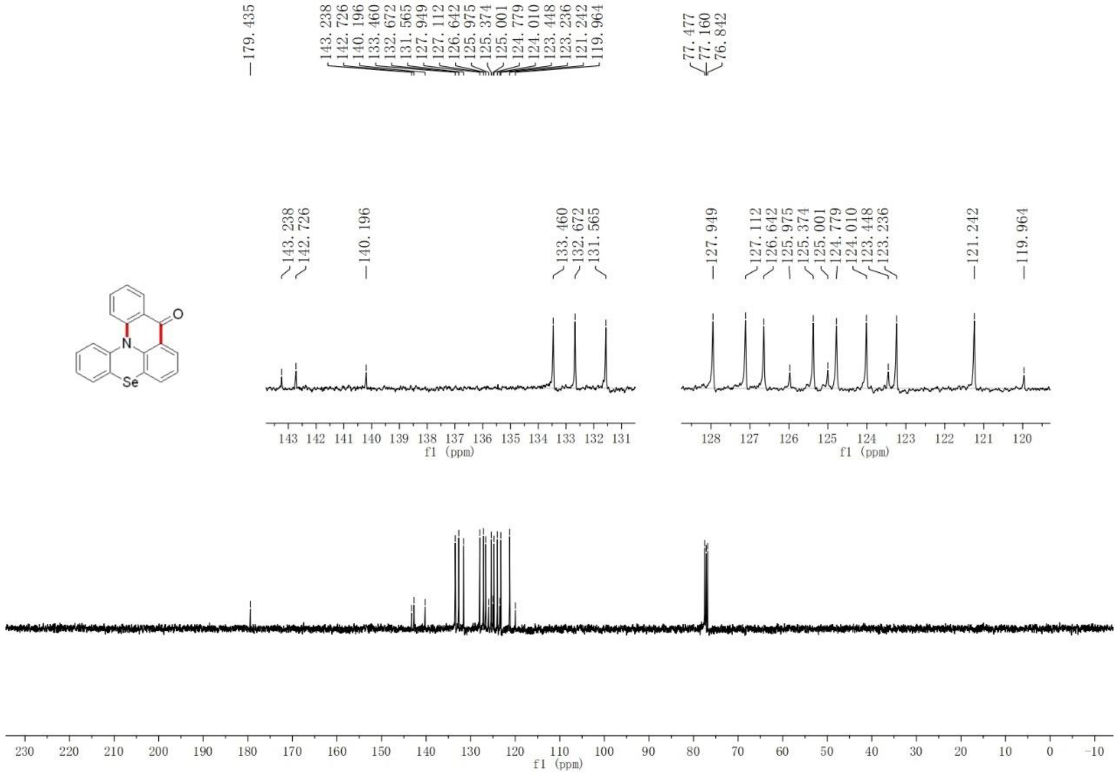
179.435
143.238 142.726 140.196 133.460 132.672 131.565 127.949 127.112 126.642 125.975 125.374 125.001 124.779 124.010 123.448 123.236 121.242 119.964
77.477 77.160 76.842
Se
N
O
f1 (ppm)

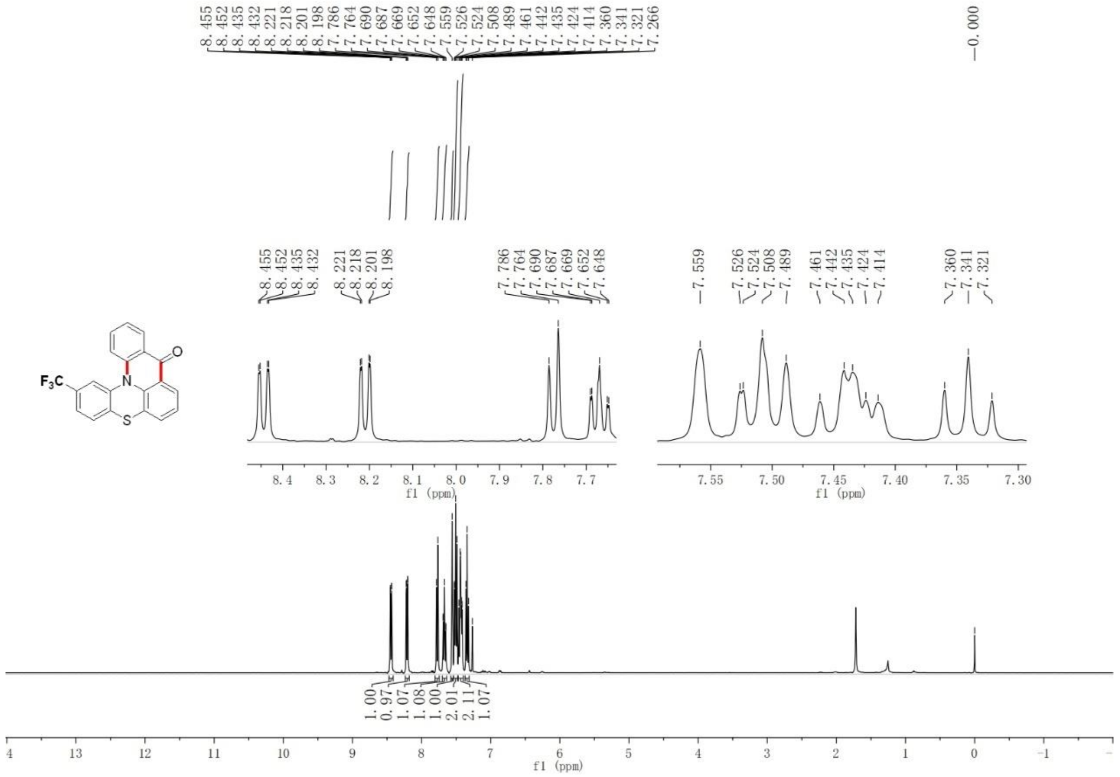
8.455 8.452 8.435 8.432 8.221 8.218 8.201 8.198 7.786 7.764 7.690 7.687 7.669 7.652 7.648 7.559 7.526 7.524 7.508 7.489 7.461 7.442 7.435 7.424 7.414 7.360 7.341 7.321 7.266
0.000
F3C
N
S
O
1.00 0.97 1.07 1.08 1.00 2.01 2.11 1.07
f1 (ppm)

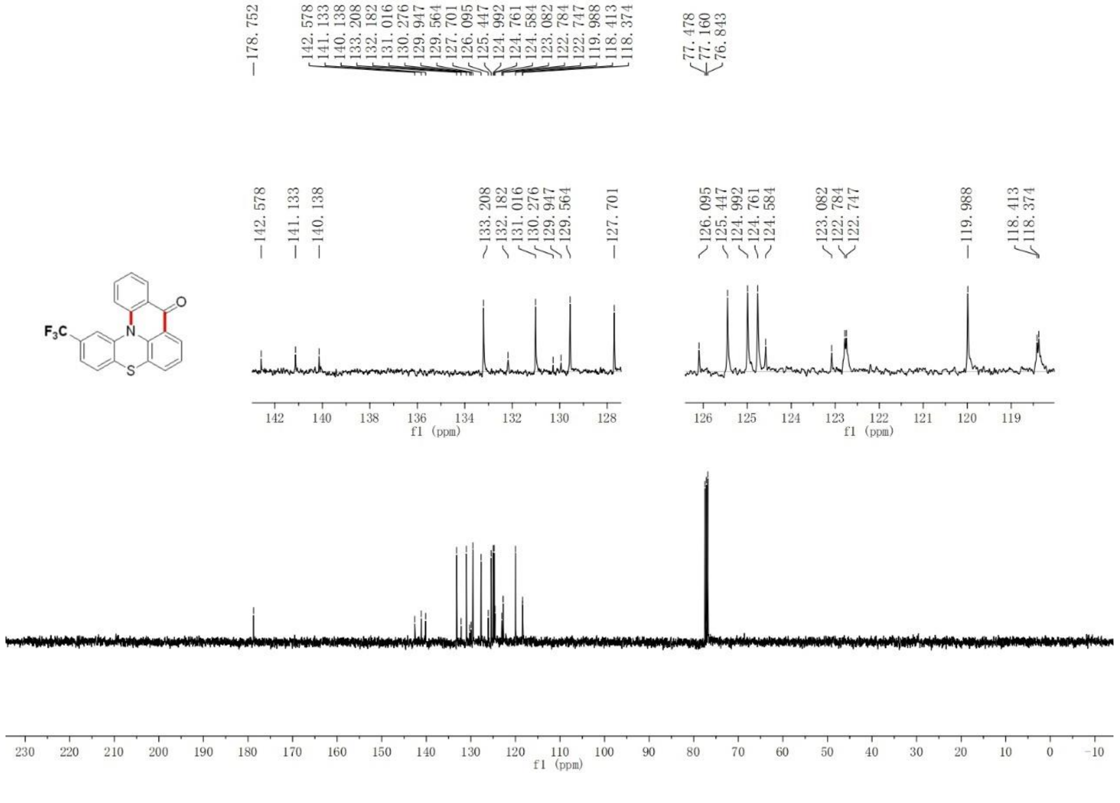
178.752
142.578 141.133 140.138 133.208 132.182 131.016 130.276 129.947 129.564 127.701 126.095 125.447 124.992 124.761 124.584 123.082 122.784 122.747 119.988 118.413 118.374
77.478 77.160 76.843
F3C
N
S
O
f1 (ppm)

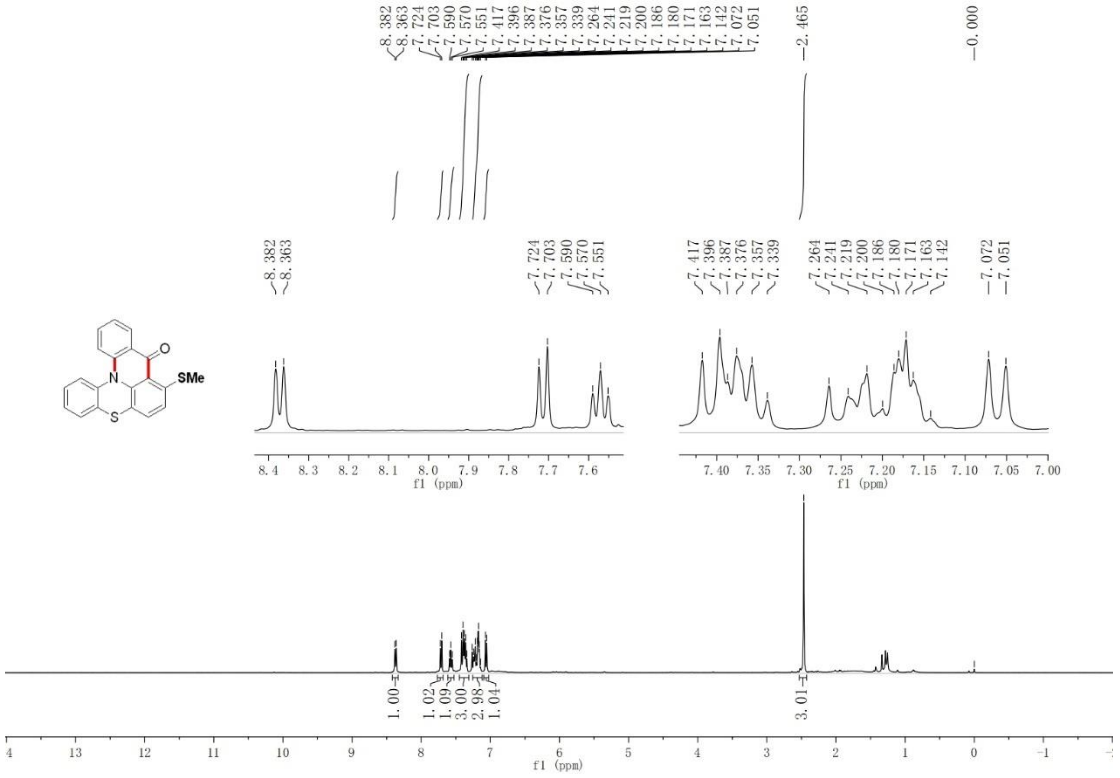
8.382
8.363
7.724
7.703
7.590
7.570
7.551
7.417
7.396
7.387
7.376
7.357
7.339
7.264
7.241
7.219
7.200
7.186
7.180
7.171
7.163
7.142
7.072
7.051
2.465
0.000
SMe
N
S
O
f1 (ppm)
1.00
1.02
1.09
3.00
2.98
1.04
3.01

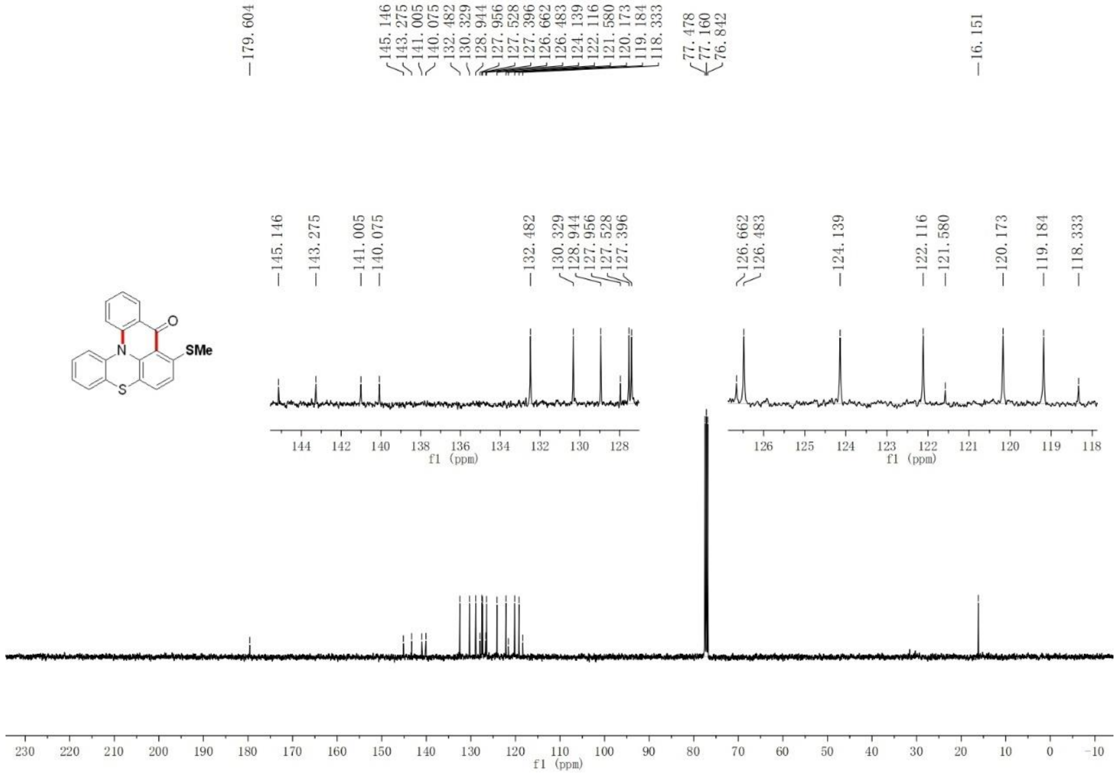
179.604
145.146
143.275
141.005
140.075
132.482
130.329
128.944
127.956
127.528
127.396
126.662
126.483
124.139
122.116
121.580
120.173
119.184
118.333
77.478
77.160
76.842
16.151
SMe
N
S
O
f1 (ppm)

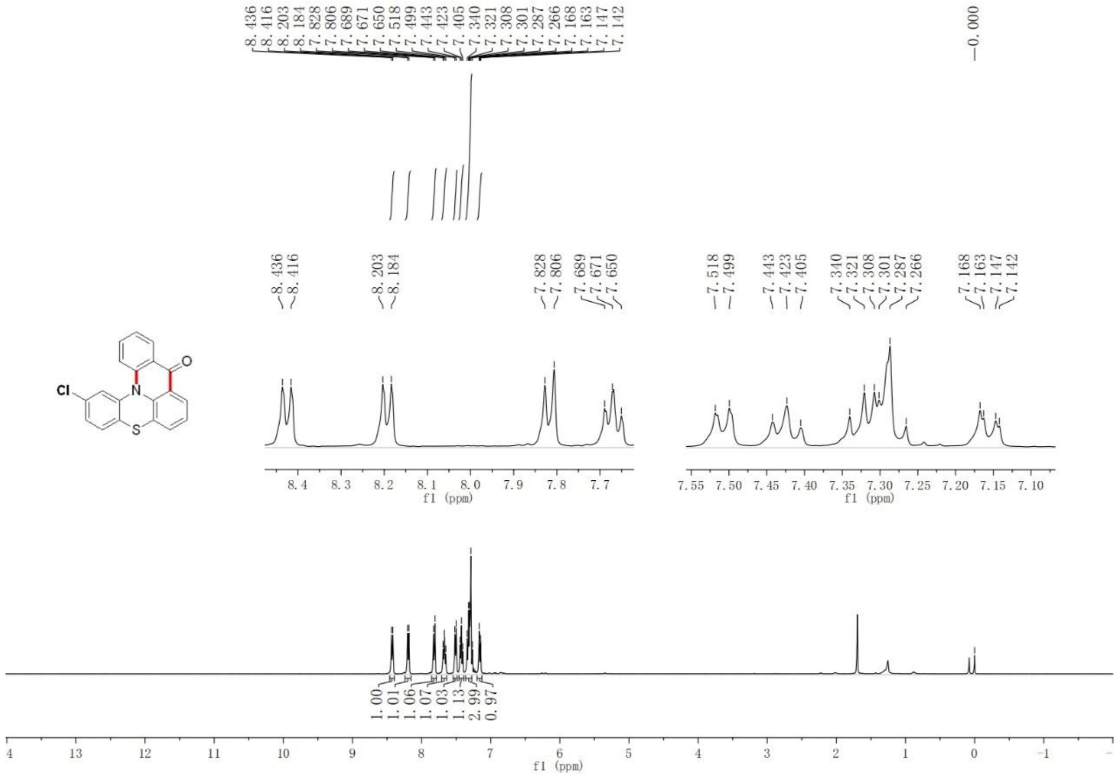
8.436 8.416 8.203 8.184 7.828 7.806 7.689 7.671 7.650 7.518 7.499 7.443 7.423 7.405 7.340 7.321 7.308 7.301 7.287 7.266 7.168 7.163 7.147 7.142
0.000
Cl
N
O
S
1.00 1.01 1.06 1.07 1.03 1.13 2.99 0.97
f1 (ppm)

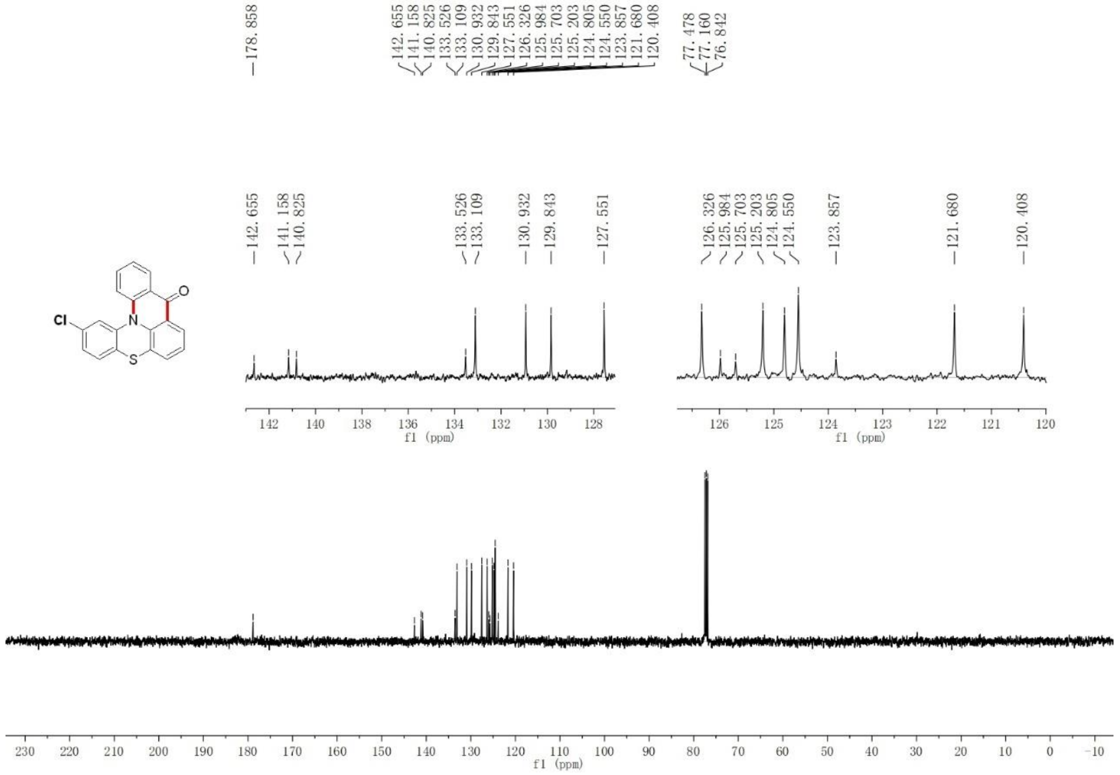
178.858
142.655 141.158 140.825 133.526 133.109 130.932 129.843 127.551 126.326 125.984 125.703 125.203 124.805 124.550 123.857 121.680 120.408
77.478 77.160 76.842
Cl
N
O
S
f1 (ppm)

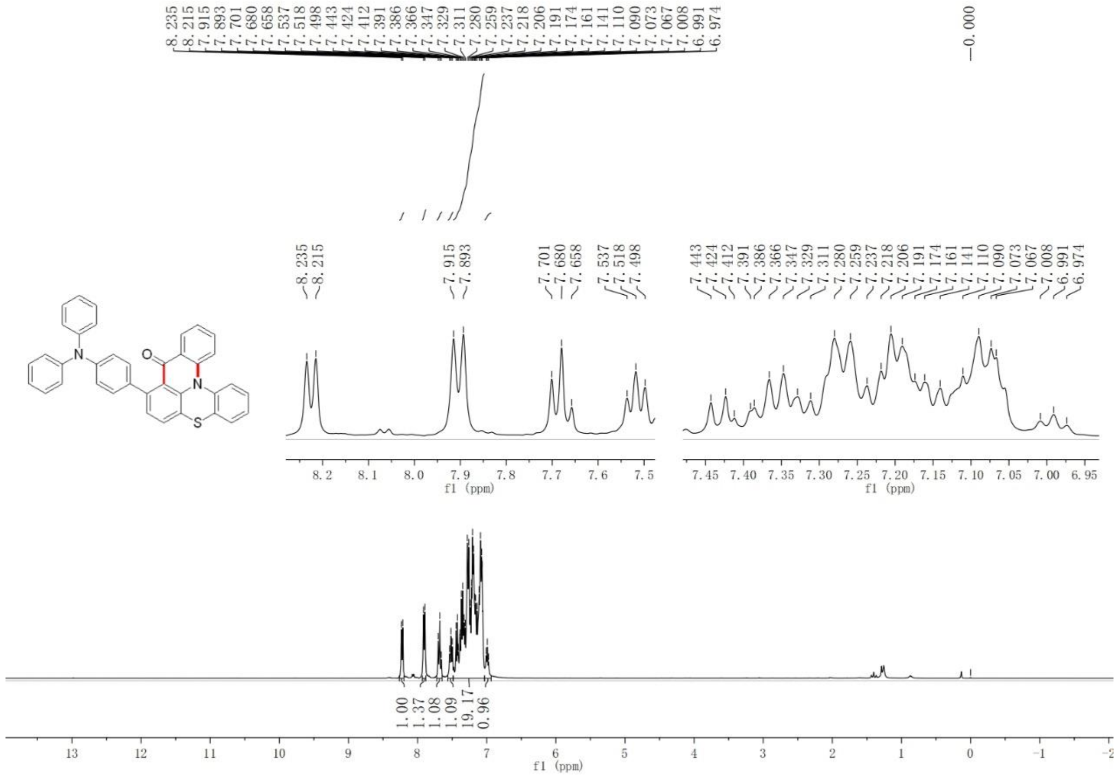
8.235 8.215 7.915 7.893 7.701 7.680 7.658 7.537 7.518 7.498 7.443 7.424 7.412 7.391 7.386 7.366 7.347 7.329 7.311 7.280 7.259 7.237 7.218 7.206 7.191 7.174 7.161 7.141 7.110 7.090 7.073 7.067 7.008 6.991 6.974
0.000
N
O
S
1.00 1.37 1.08 1.09 19.17 0.96
f1 (ppm)

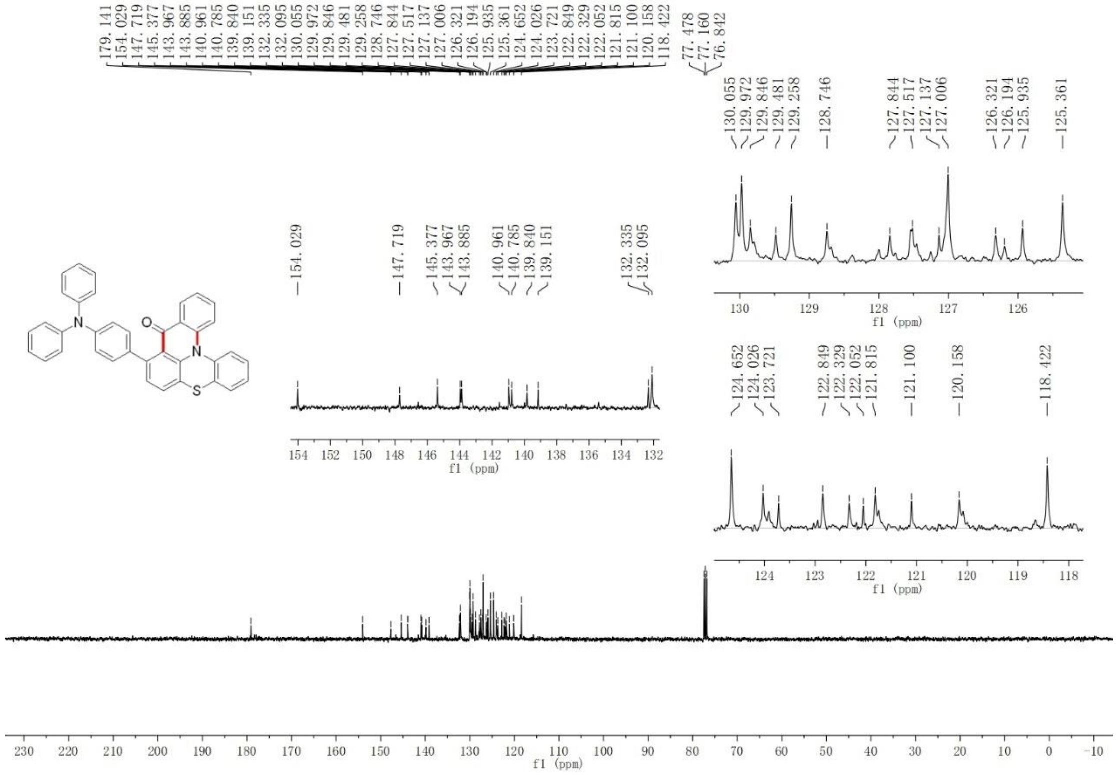
179.141 154.029 147.719 145.377 143.967 143.885 140.961 140.785 139.840 139.151 132.335 132.095 130.055 129.972 129.846 129.481 129.258 128.746 127.844 127.517 127.137 127.006 126.321 126.194 125.935 125.361 124.652 124.026 123.721 122.849 122.329 122.052 121.815 121.100 120.158 118.422
77.478 77.160 76.842
N
O
S
f1 (ppm)

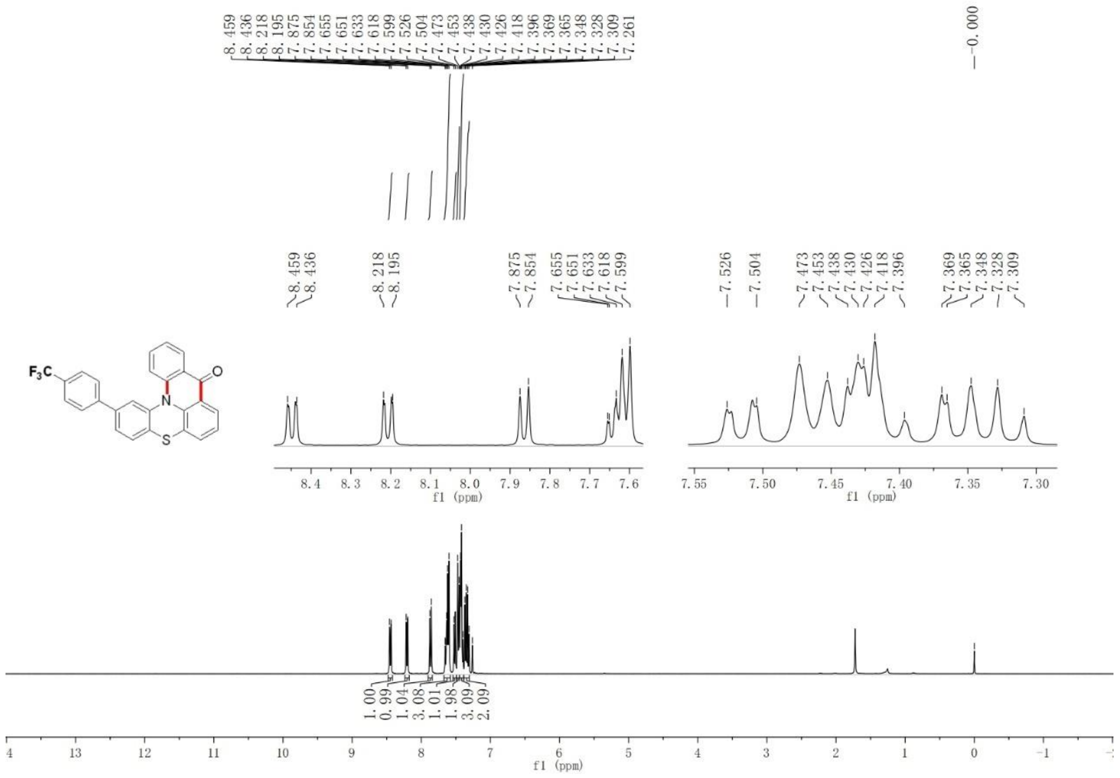
8.459 8.436 8.218 8.195 7.875 7.854 7.655 7.651 7.633 7.618 7.599 7.526 7.504 7.473 7.453 7.438 7.430 7.426 7.418 7.396 7.369 7.365 7.348 7.328 7.309 7.261
0.000
F3C
N
O
S
1.00 0.99 1.04 3.08 1.01 1.98 3.09 2.09
f1 (ppm)

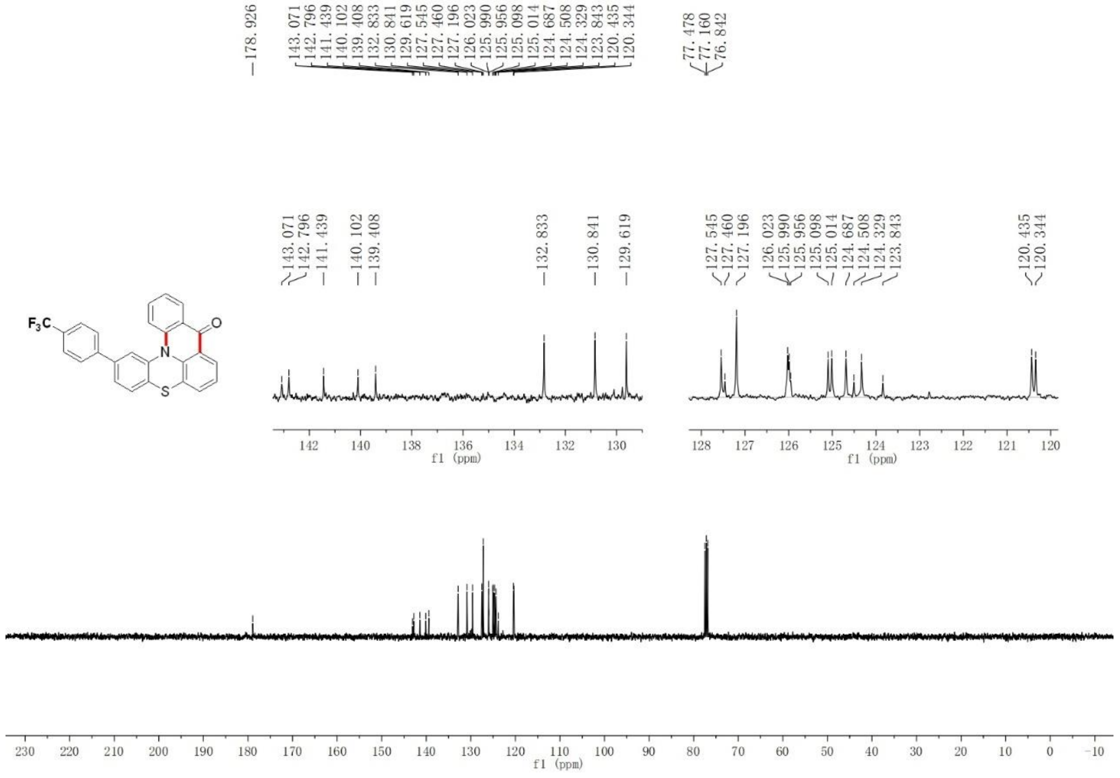
178.926
143.071 142.796 141.439 140.102 139.408 132.833 130.841 129.619 127.545 127.460 127.196 126.023 125.990 125.956 125.098 125.014 124.687 124.508 124.329 123.843 120.435 120.344
77.478 77.160 76.842
F3C
N
O
S
f1 (ppm)

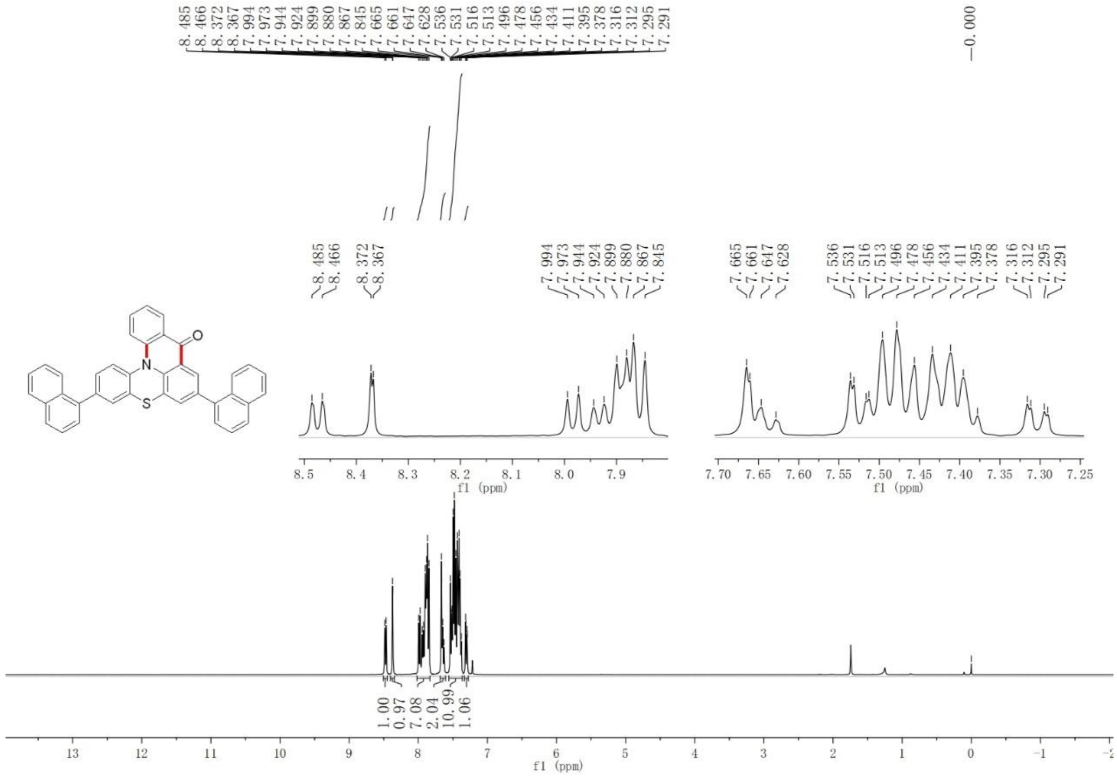
8.485 8.466 8.372 8.367 7.994 7.973 7.944 7.924 7.899 7.880 7.867 7.845 7.665 7.661 7.647 7.628 7.536 7.531 7.516 7.513 7.496 7.478 7.456 7.434 7.411 7.395 7.378 7.316 7.312 7.295 7.291
0.000
N
O
S
1.00 0.97 7.08 2.04 10.99 1.06
f1 (ppm)

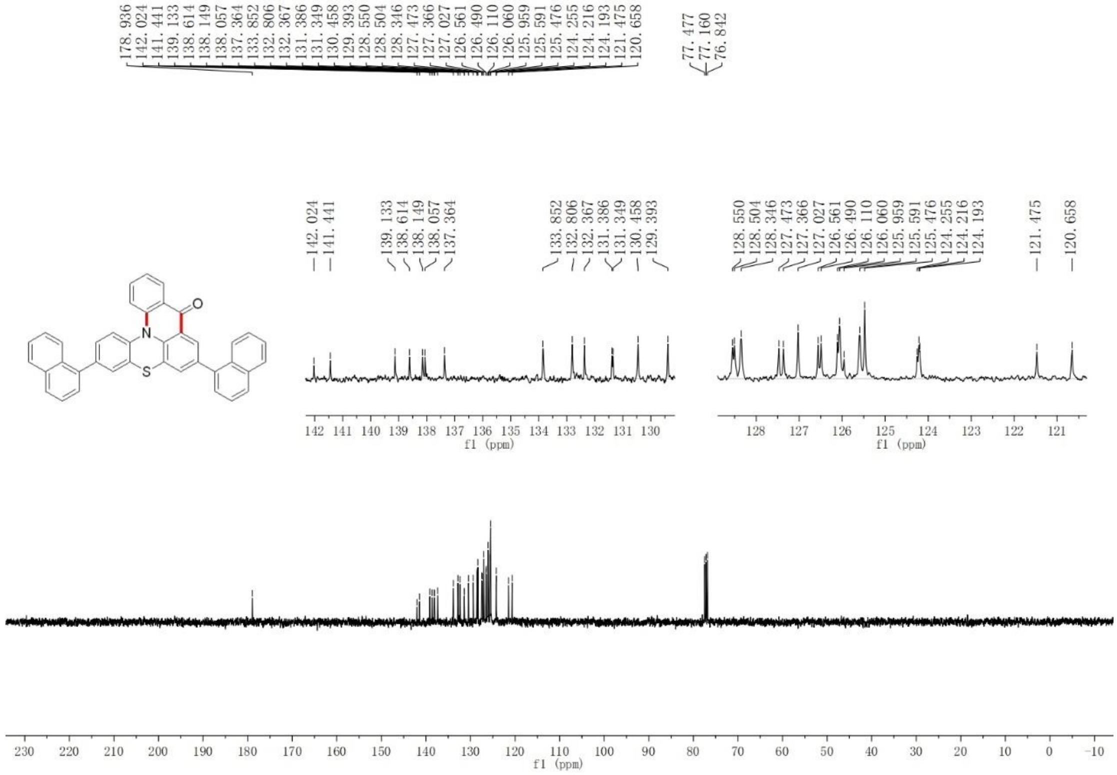
178.936 142.024 141.441 139.133 138.614 138.149 138.057 137.364 133.852 132.806 132.367 131.386 131.349 130.458 129.393 128.550 128.504 128.346 127.473 127.366 127.027 126.561 126.490 126.110 126.060 125.959 125.591 125.476 124.255 124.216 124.193 121.475 120.658
77.477 77.160 76.842
N
O
S
f1 (ppm)

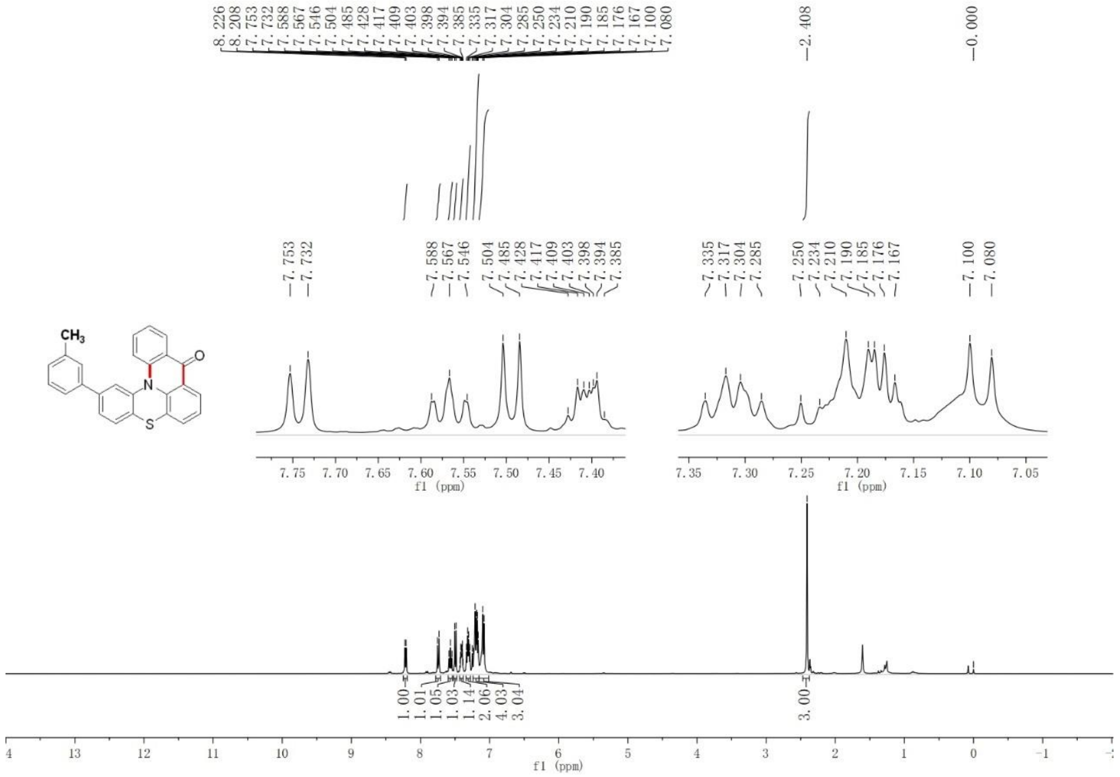
8.226 8.208 7.753 7.732 7.588 7.567 7.546 7.504 7.485 7.428 7.417 7.409 7.403 7.398 7.394 7.385 7.335 7.317 7.304 7.285 7.250 7.234 7.210 7.190 7.185 7.176 7.167 7.100 7.080
2.408
0.000
CH3
N
O
S
1.00 1.01 1.05 1.03 1.14 2.06 4.03 3.04
3.00
f1 (ppm)

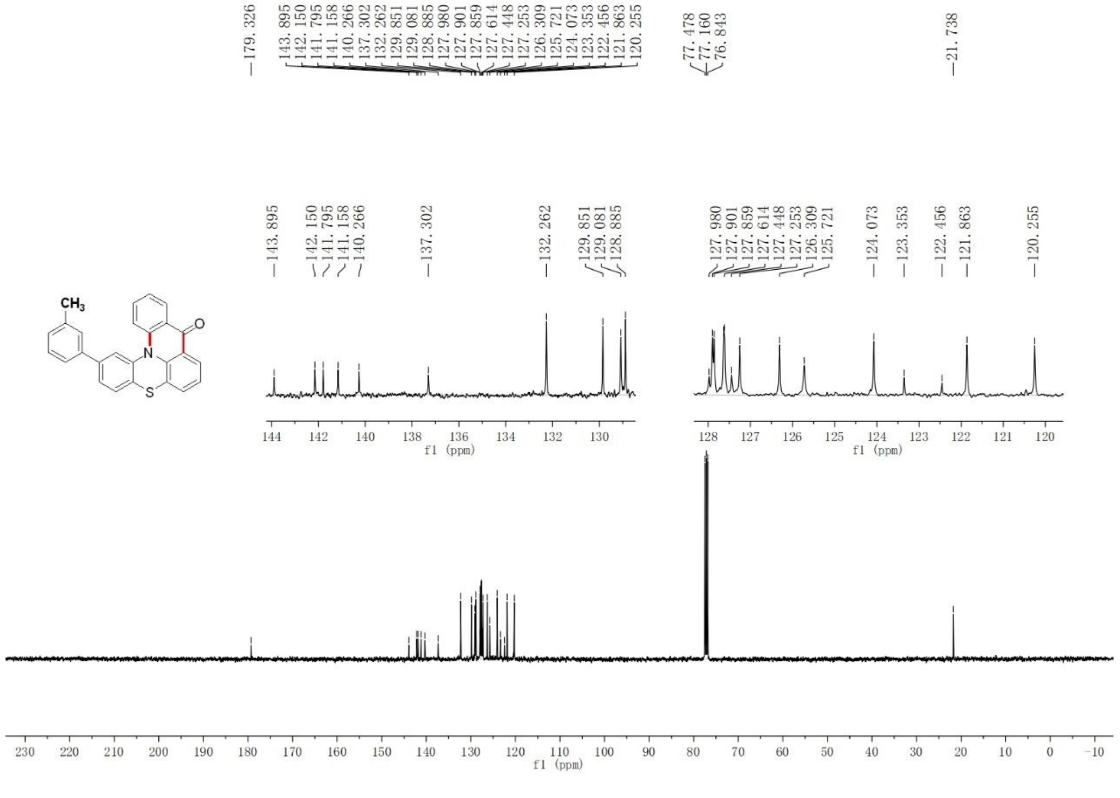
179.326
143.895 142.150 141.795 141.158 140.266 137.302 132.262 129.851 129.081 128.885 127.980 127.901 127.859 127.614 127.448 127.253 126.309 125.721 124.073 123.353 122.456 121.863 120.255
77.478 77.160 76.843
21.738
CH3
N
O
S
f1 (ppm)

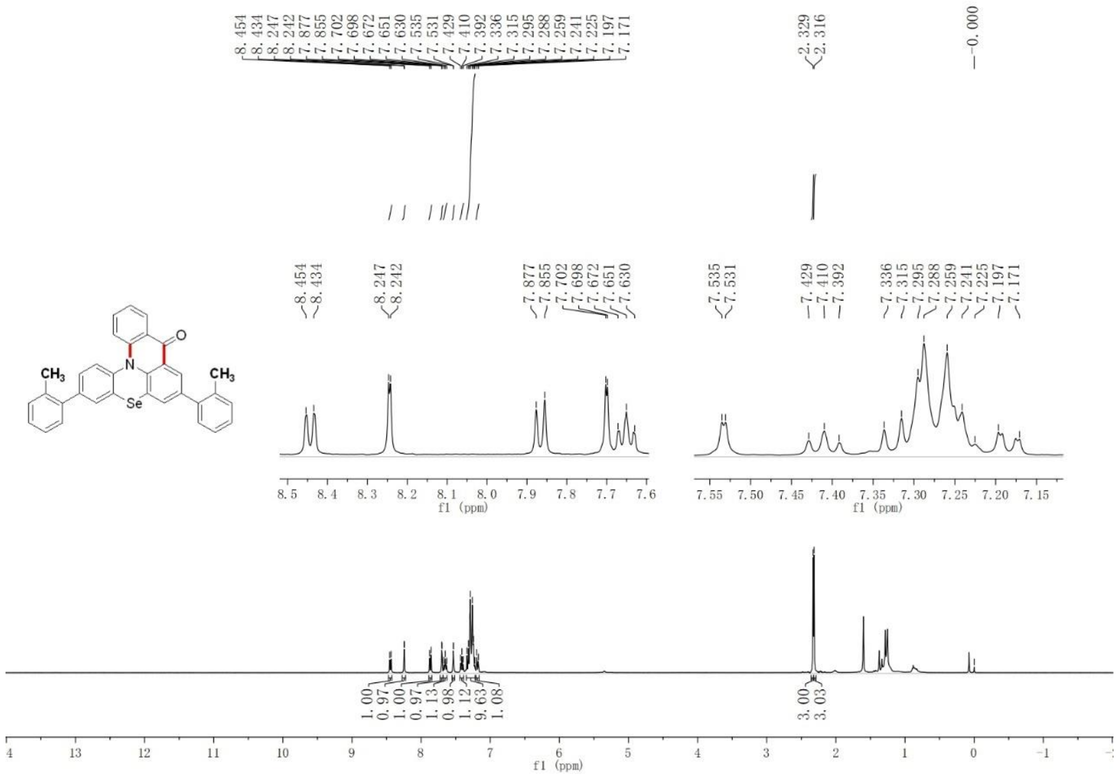
8.454
8.434
8.247
8.242
7.877
7.855
7.702
7.698
7.672
7.651
7.630
7.535
7.531
7.429
7.410
7.392
7.336
7.315
7.295
7.288
7.259
7.241
7.225
7.197
7.171
2.329
2.316
0.000
CH3
N
O
Se
1.00
0.97
1.00
0.97
1.13
0.98
1.12
9.63
1.08
3.00
3.03
f1 (ppm)

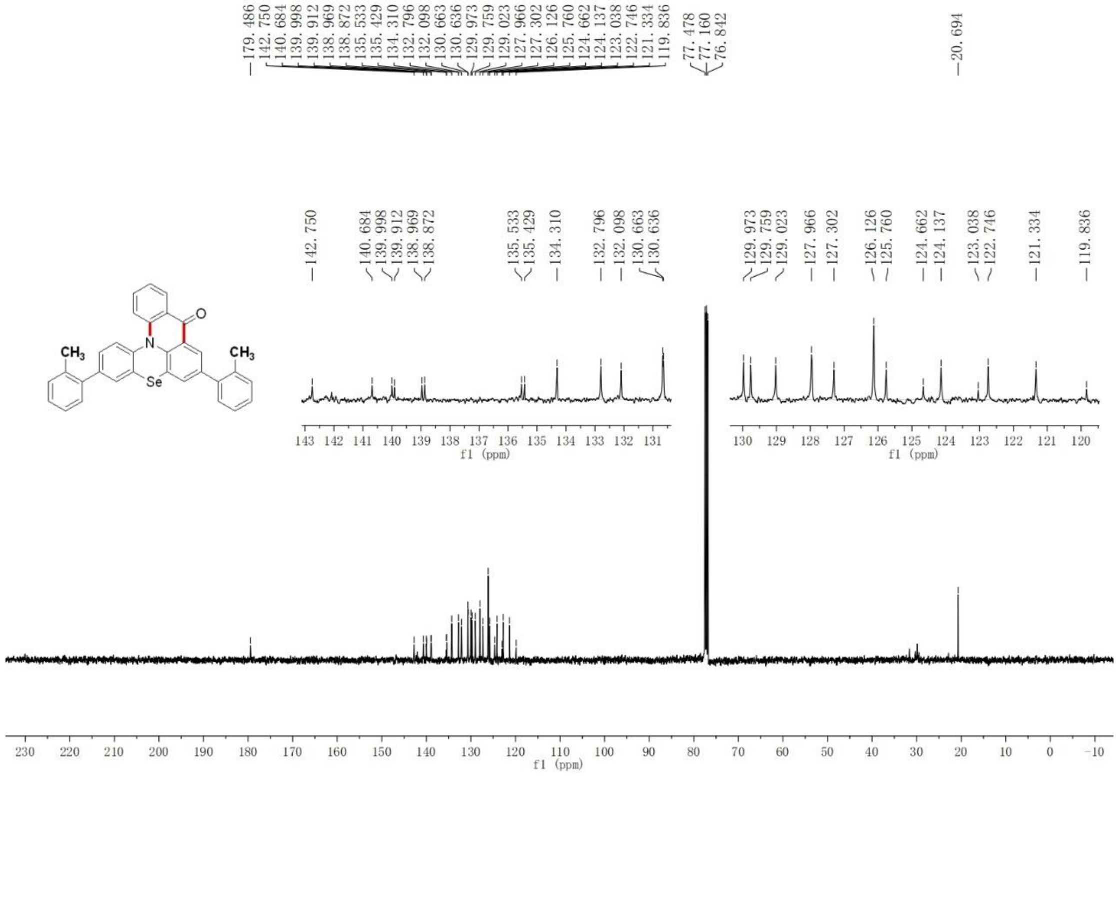
179.486
142.750
140.684
139.998
139.912
138.969
138.872
135.533
135.429
134.310
132.796
132.098
130.663
130.636
129.973
129.759
129.023
127.966
127.302
126.126
125.760
124.662
124.137
123.038
122.746
121.334
119.836
77.478
77.160
76.842
20.694
CH3
N
O
Se
f1 (ppm)

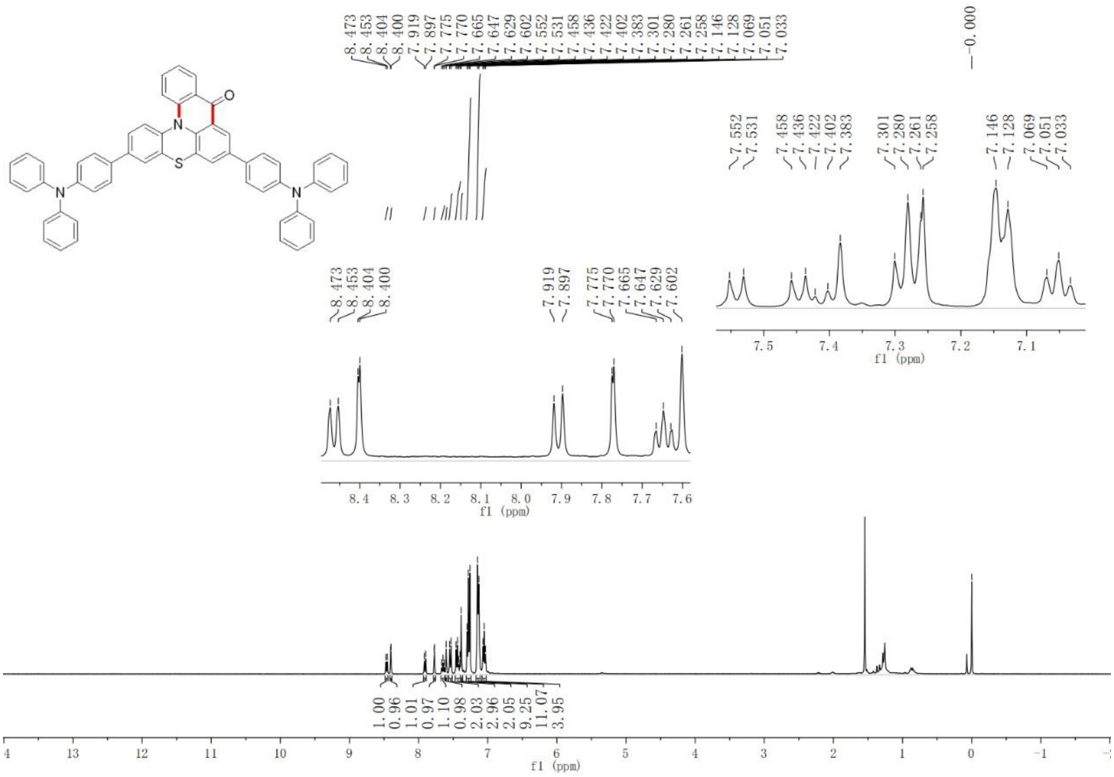
8.473
8.453
8.404
8.400
7.919
7.897
7.775
7.770
7.665
7.647
7.629
7.602
7.552
7.531
7.458
7.436
7.422
7.402
7.383
7.301
7.280
7.261
7.258
7.146
7.128
7.069
7.051
7.033
0.000
N
S
O
1.00
0.96
1.01
0.97
1.10
0.98
2.03
2.96
2.05
9.25
11.07
3.95
f1 (ppm)

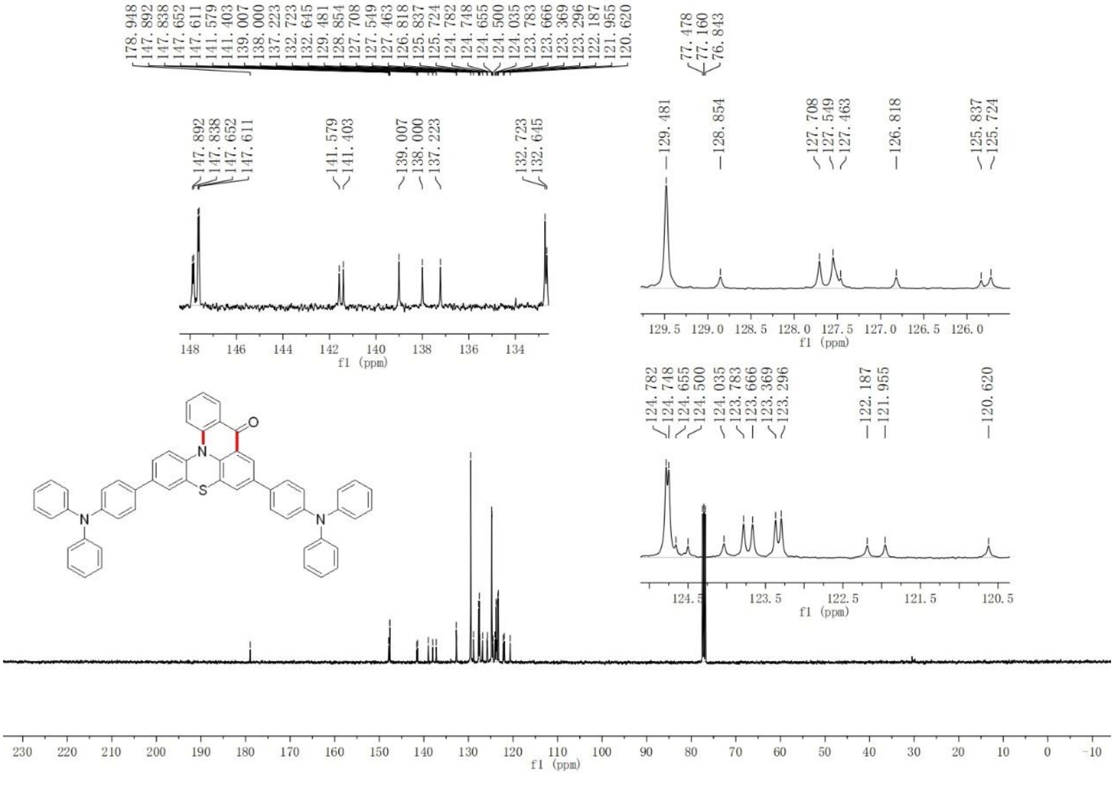
178.948
147.892
147.838
147.652
147.611
141.579
141.403
139.007
138.000
137.223
132.723
132.645
129.481
128.854
127.708
127.549
127.463
126.818
125.837
125.724
124.782
124.748
124.655
124.500
124.035
123.783
123.666
123.369
123.296
122.187
121.955
120.620
77.478
77.160
76.843
N
S
O
f1 (ppm)

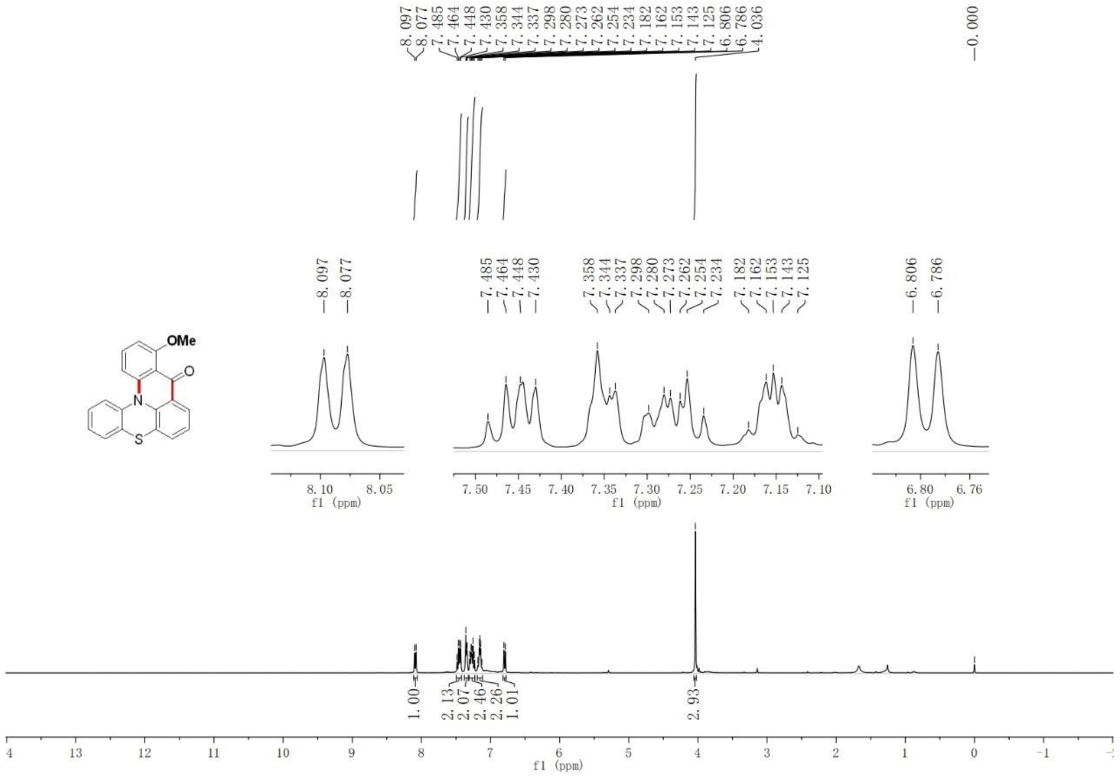
8.097
8.077
7.485
7.464
7.448
7.430
7.358
7.344
7.337
7.298
7.280
7.273
7.262
7.254
7.234
7.182
7.162
7.153
7.143
7.125
6.806
6.786
4.036
0.000
OMe
O
N
S
1.00
2.13
2.07
2.46
2.26
1.01
2.93
f1 (ppm)

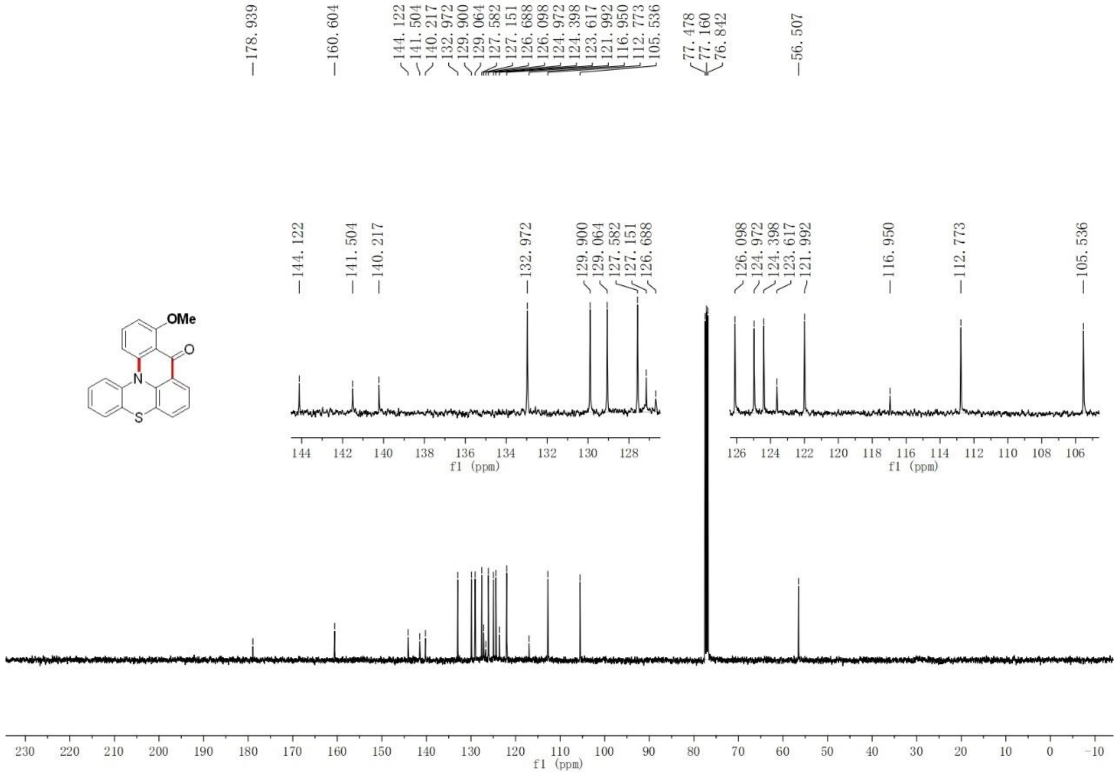
178.939
160.604
144.122
141.504
140.217
132.972
129.900
129.064
127.582
127.151
126.688
126.098
124.972
124.398
123.617
121.992
116.950
112.773
105.536
77.478
77.160
76.842
56.507
OMe
O
N
S
f1 (ppm)

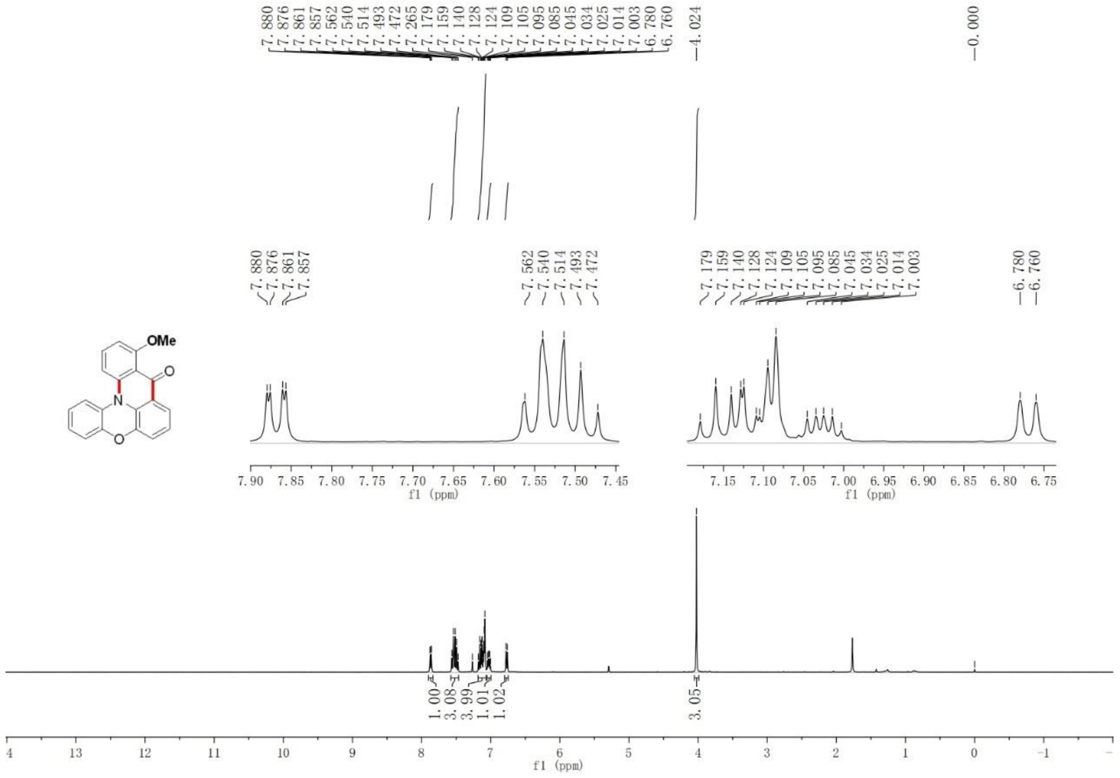
7.880 7.876 7.861 7.857 7.562 7.540 7.514 7.493 7.472 7.265 7.179 7.159 7.140 7.128 7.124 7.109 7.105 7.095 7.085 7.045 7.034 7.025 7.014 7.003 6.780 6.760
4.024
0.000
OMe
N
O
1.00 3.08 3.99 1.01 1.02
3.05
f1 (ppm)

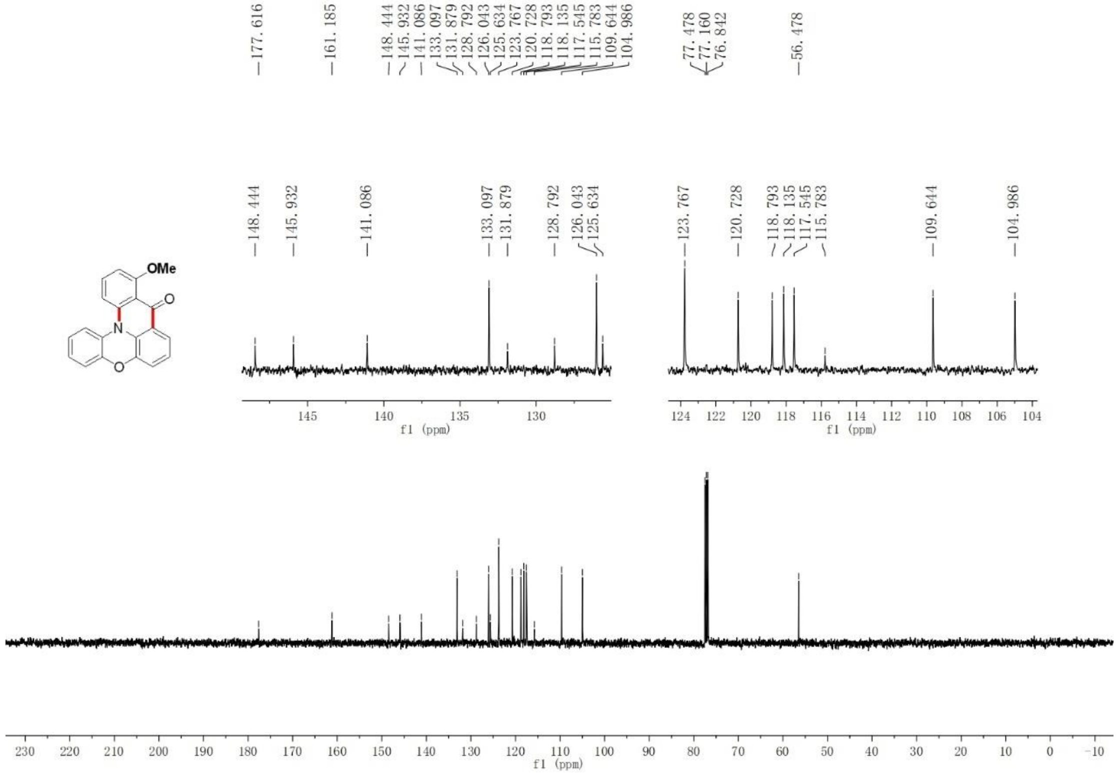
177.616
161.185
148.444 145.932 141.086 133.097 131.879 128.792 126.043 125.634 123.767 120.728 118.793 118.135 117.545 115.783 109.644 104.986
77.478 77.160 76.842
56.478
OMe
N
O
f1 (ppm)

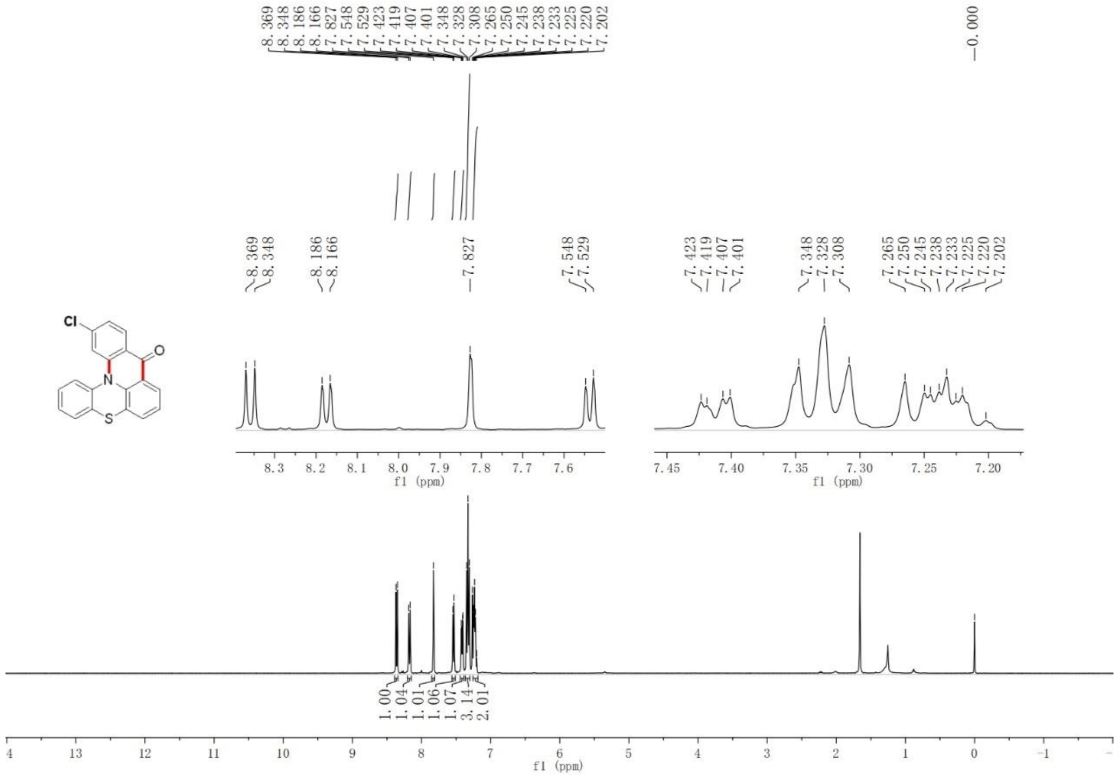
8.369 8.348 8.186 8.166 7.827 7.548 7.529 7.423 7.419 7.407 7.401 7.348 7.328 7.308 7.265 7.250 7.245 7.238 7.233 7.225 7.220 7.202
0.000
Cl
N
O
S
1.00 1.04 1.01 1.06 1.07 3.14 2.01
f1 (ppm)

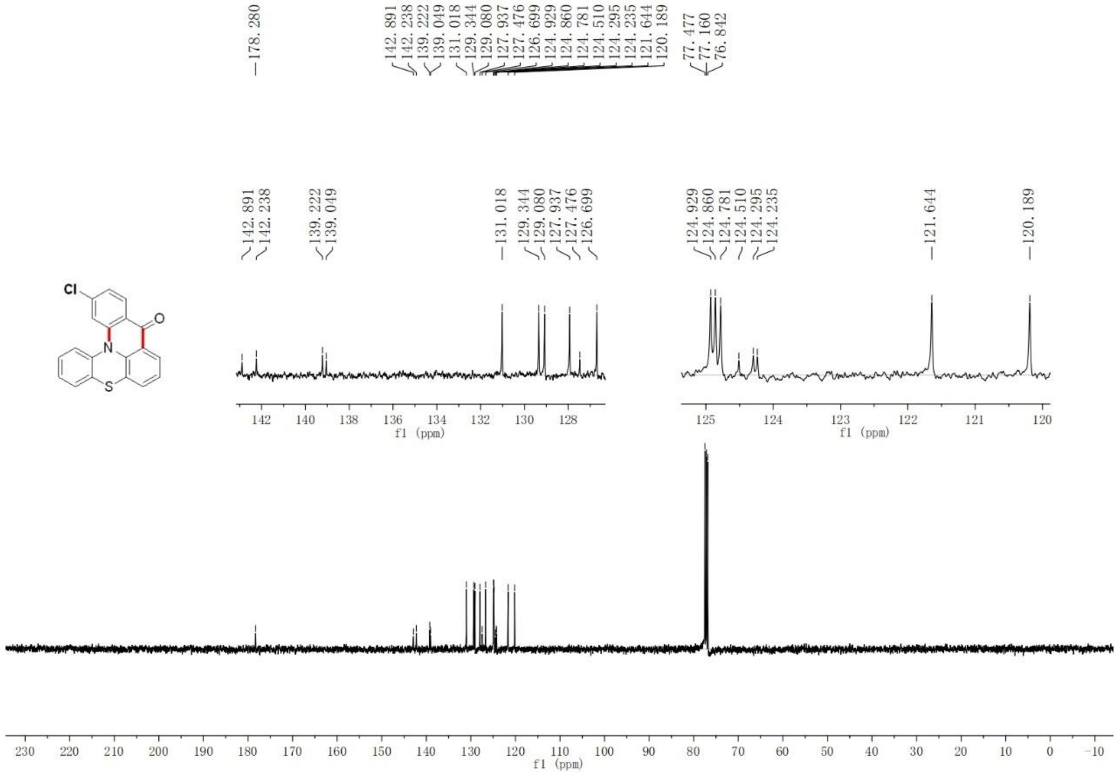
178.280
142.891 142.238 139.222 139.049 131.018 129.344 129.080 127.937 127.476 126.699 124.929 124.860 124.781 124.510 124.295 124.235 121.644 120.189
77.477 77.160 76.842
Cl
N
O
S
f1 (ppm)

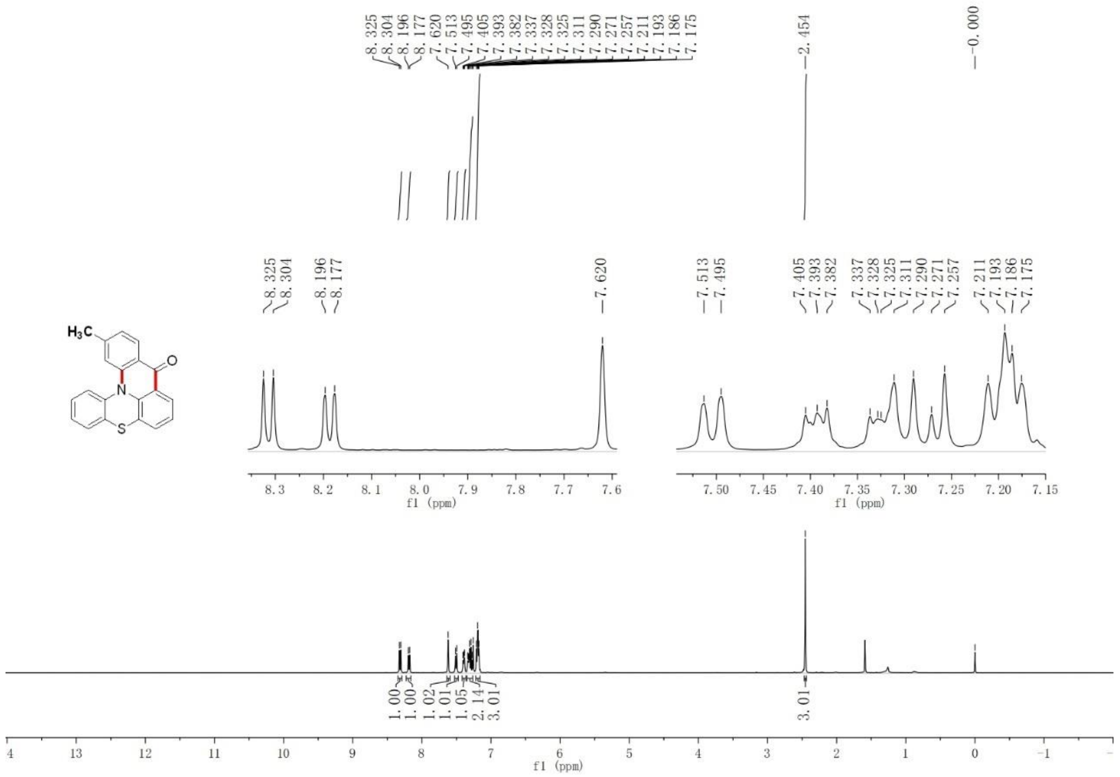
8.325
8.304
8.196
8.177
7.620
7.513
7.495
7.405
7.393
7.382
7.337
7.328
7.325
7.311
7.290
7.271
7.257
7.211
7.193
7.186
7.175
2.454
0.000
H3C
N
S
O
1.00
1.00
1.02
1.01
1.05
2.14
3.01
3.01
f1 (ppm)

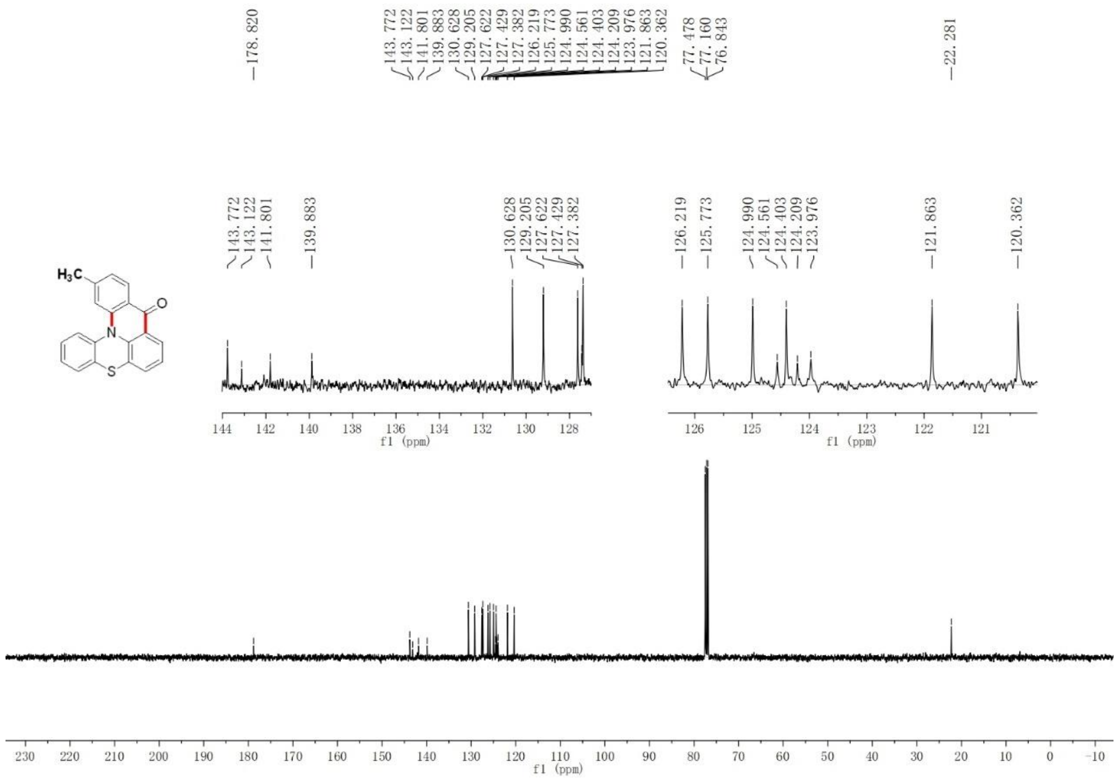
178.820
143.772
143.122
141.801
139.883
130.628
129.205
127.622
127.429
127.382
126.219
125.773
124.990
124.561
124.403
124.209
123.976
121.863
120.362
77.478
77.160
76.843
22.281
H3C
N
S
O
f1 (ppm)

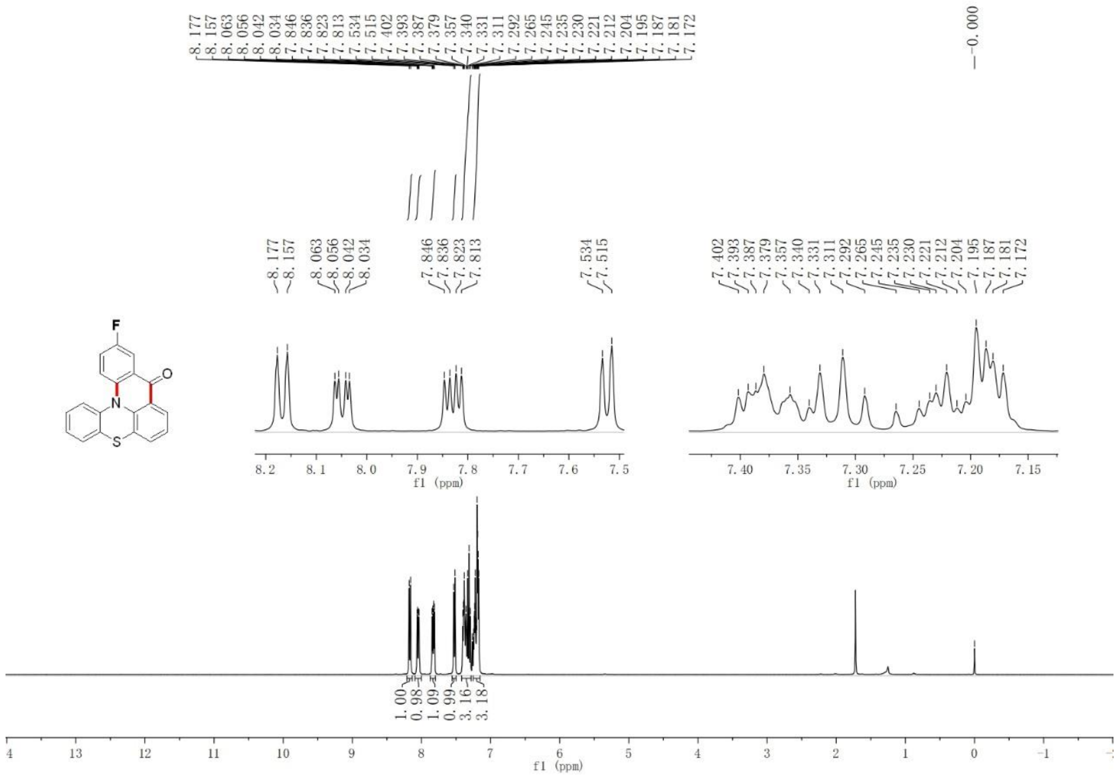
F
N
O
S
f1 (ppm)

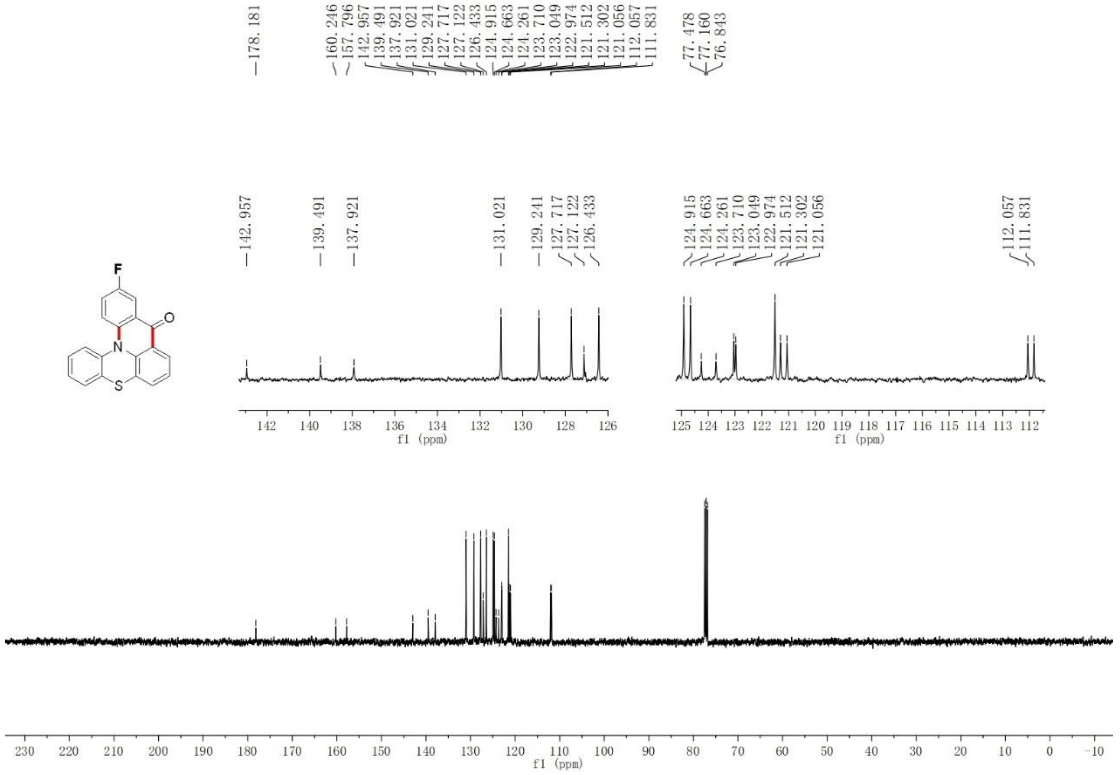
F
N
O
S
f1 (ppm)

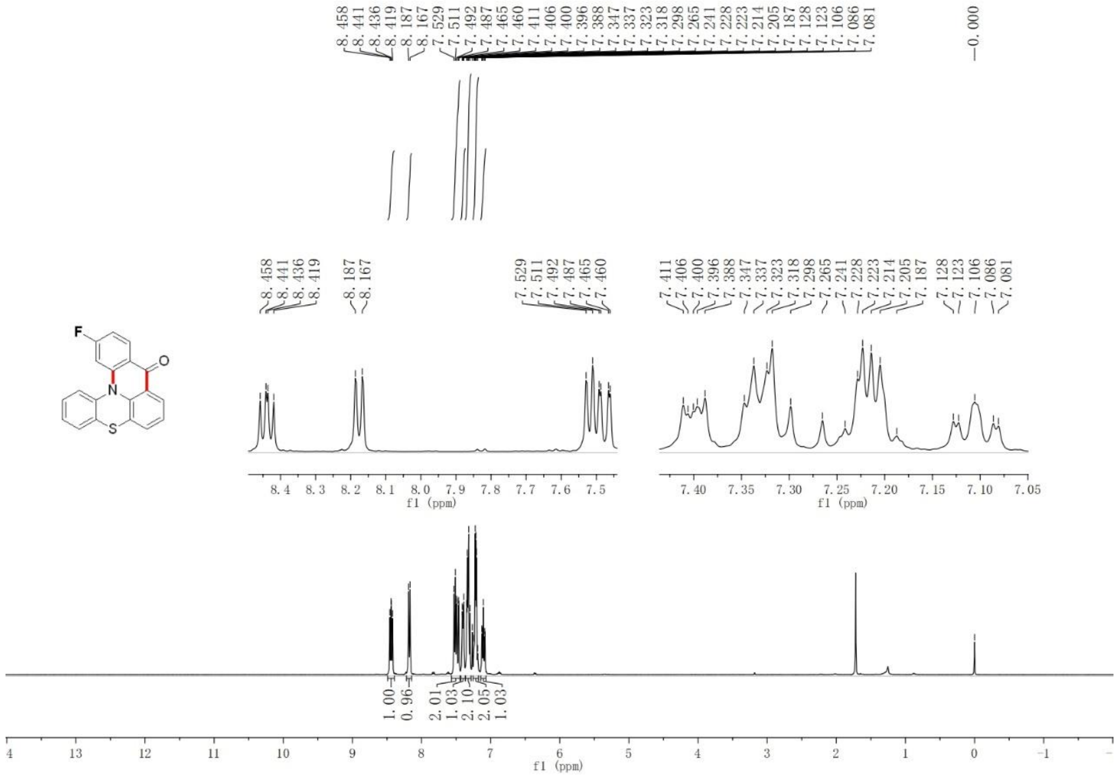
8.458
8.441
8.436
8.419
8.187
8.167
7.529
7.511
7.492
7.487
7.465
7.460
7.411
7.406
7.400
7.396
7.388
7.347
7.337
7.323
7.318
7.298
7.265
7.241
7.228
7.223
7.214
7.205
7.187
7.128
7.123
7.106
7.086
7.081
0.000
F
N
O
S
f1 (ppm)
1.00
0.96
2.01
1.03
2.10
2.05
1.03

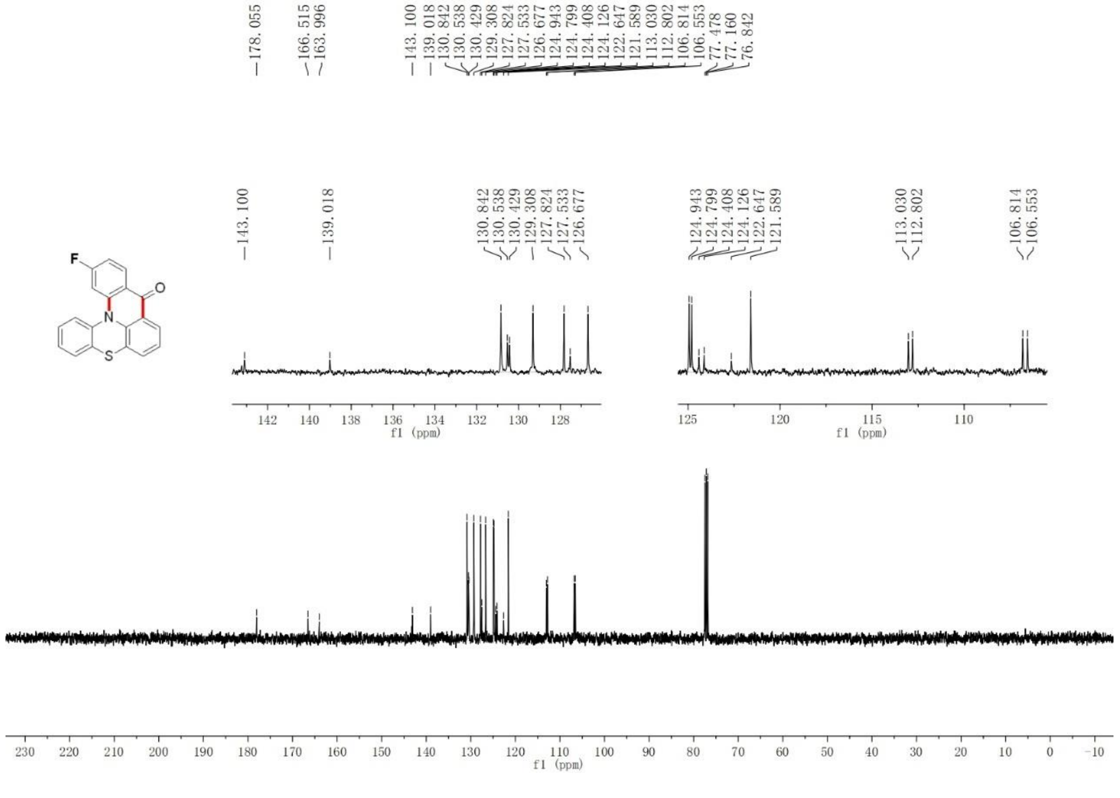
178.055
166.515
163.996
143.100
139.018
130.842
130.538
130.429
129.308
127.824
127.533
126.677
124.943
124.799
124.408
124.126
122.647
121.589
113.030
112.802
106.814
106.553
77.478
77.160
76.842
F
N
O
S
f1 (ppm)

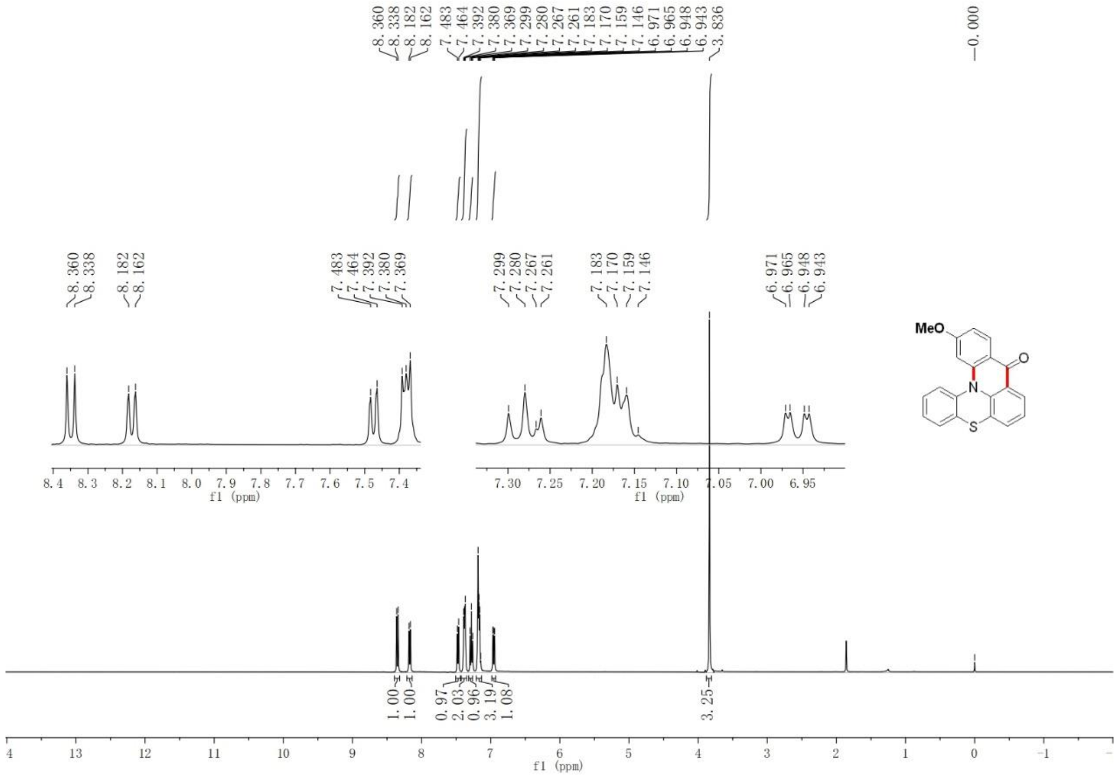
8.360 8.338 8.182 8.162 7.483 7.464 7.392 7.380 7.369 7.299 7.280 7.267 7.261 7.183 7.170 7.159 7.146 6.971 6.965 6.948 6.943 3.836
0.000
1.00 1.00 0.97 2.03 0.96 3.19 1.08 3.25
MeO
O
N
S
f1 (ppm)

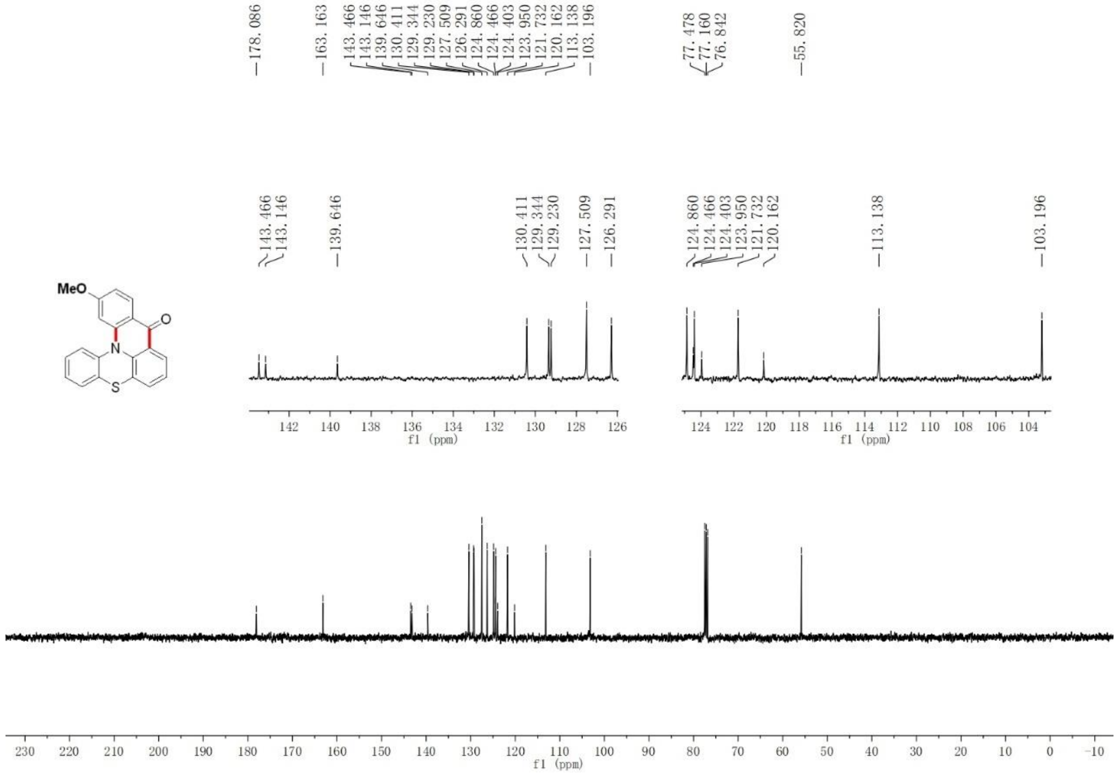
178.086
163.163
143.466 143.146 139.646 130.411 129.344 129.230 127.509 126.291 124.860 124.466 124.403 123.950 121.732 120.162 113.138 103.196
77.478 77.160 76.842
55.820
MeO
O
N
S
f1 (ppm)

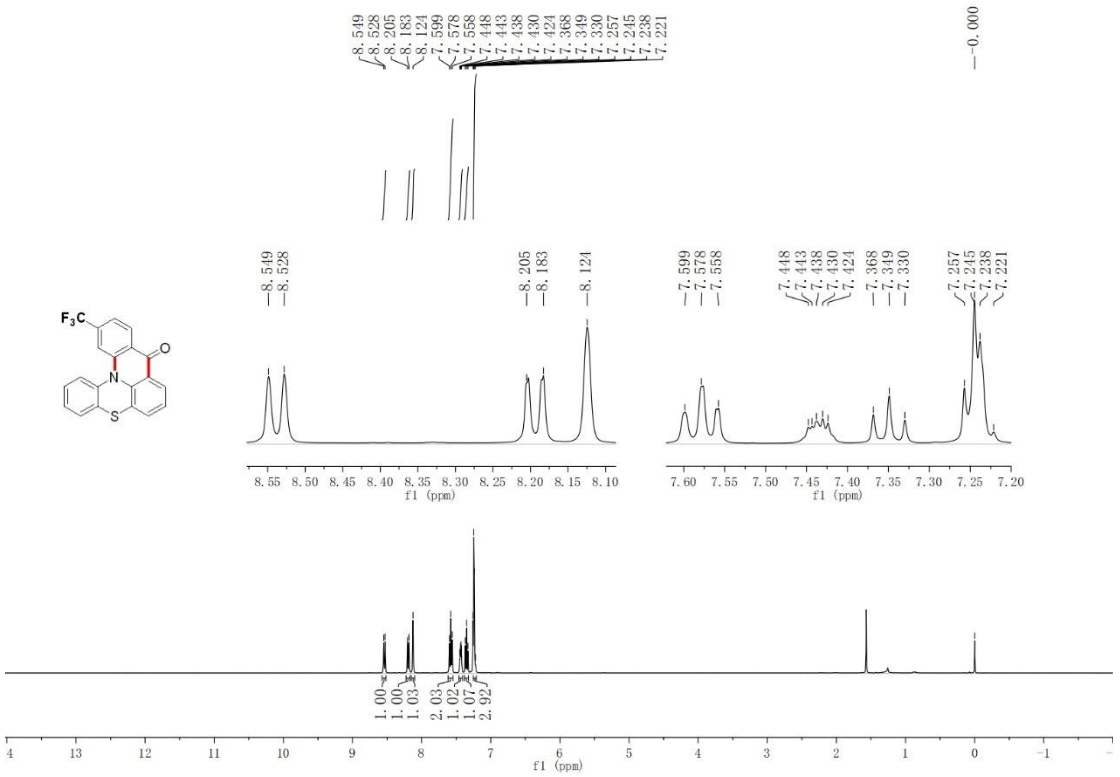
8.549
8.528
8.205
8.183
8.124
7.599
7.578
7.558
7.448
7.443
7.438
7.430
7.424
7.368
7.349
7.330
7.257
7.245
7.238
7.221
0.000
F_3C
N
O
S
1.00
1.00
1.03
2.03
1.02
1.07
2.92
f1 (ppm)

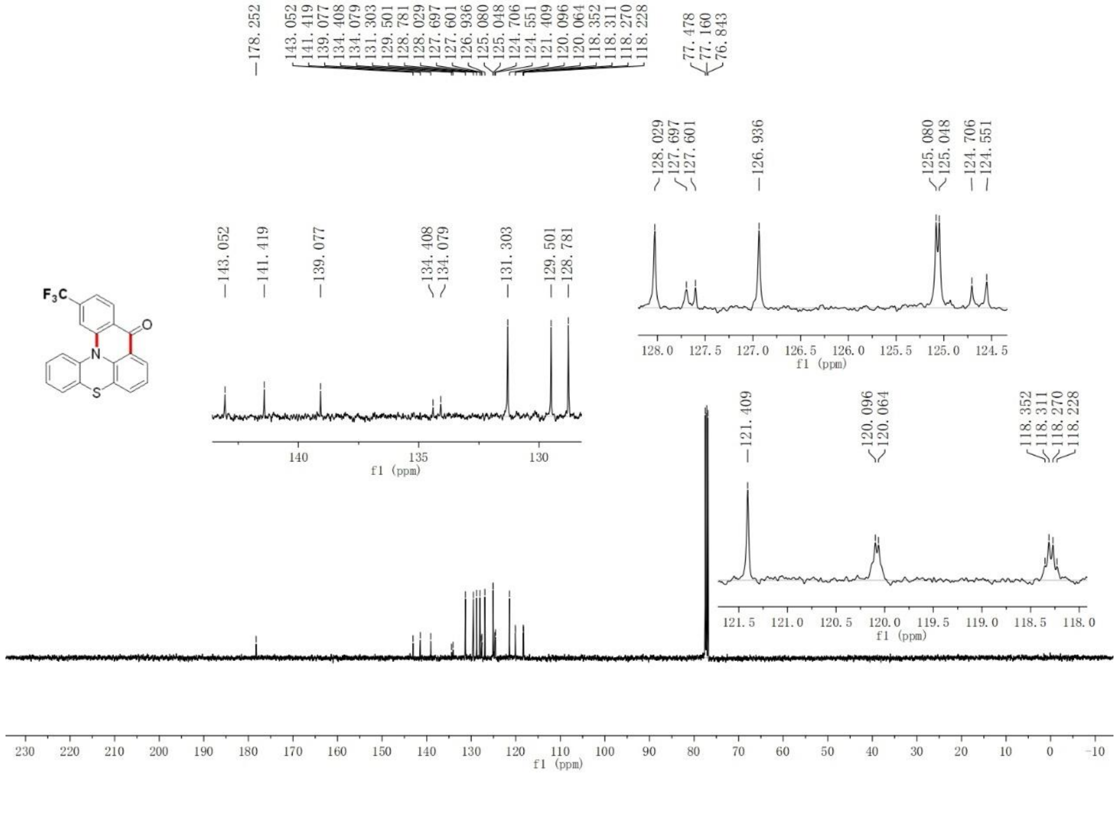
178.252
143.052
141.419
139.077
134.408
134.079
131.303
129.501
128.781
128.029
127.697
127.601
126.936
125.080
125.048
124.706
124.551
121.409
120.096
120.064
118.352
118.311
118.270
118.228
77.478
77.160
76.843
F_3C
N
O
S
f1 (ppm)

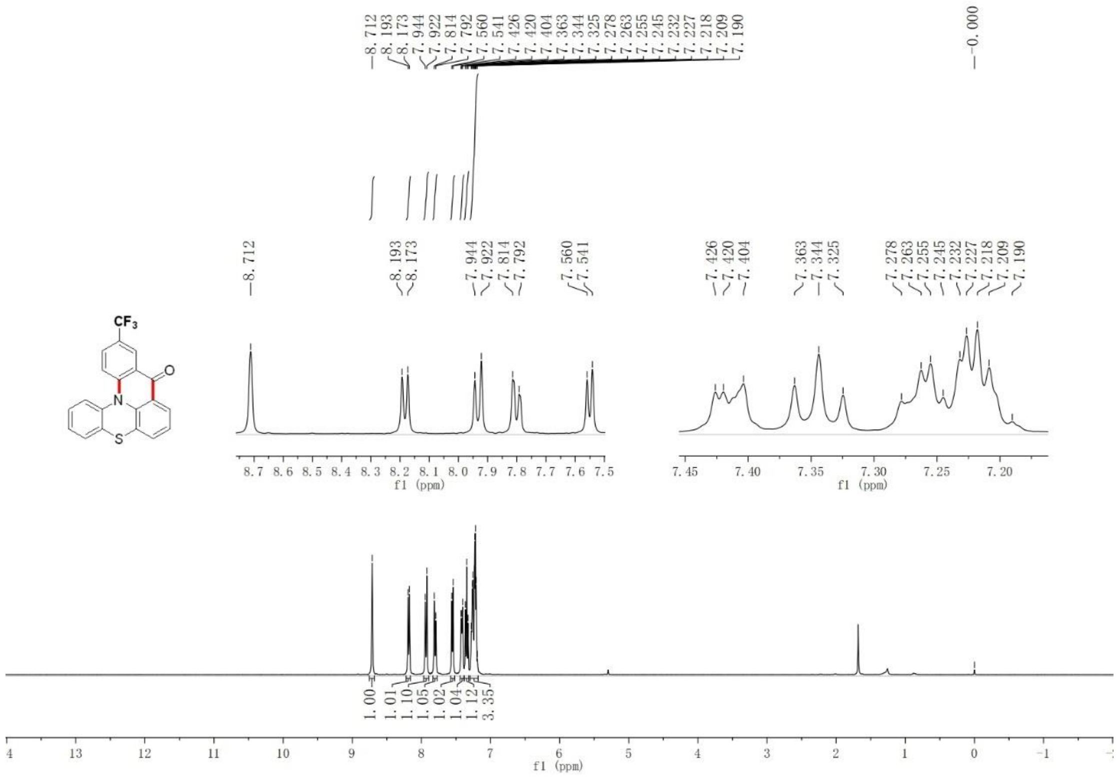
8.712 8.193 8.173 7.944 7.922 7.814 7.792 7.560 7.541 7.426 7.420 7.404 7.363 7.344 7.325 7.278 7.263 7.255 7.245 7.232 7.227 7.218 7.209 7.190
0.000
CF3
N
O
S
1.00 1.01 1.10 1.05 1.02 1.04 1.12 3.35
f1 (ppm)

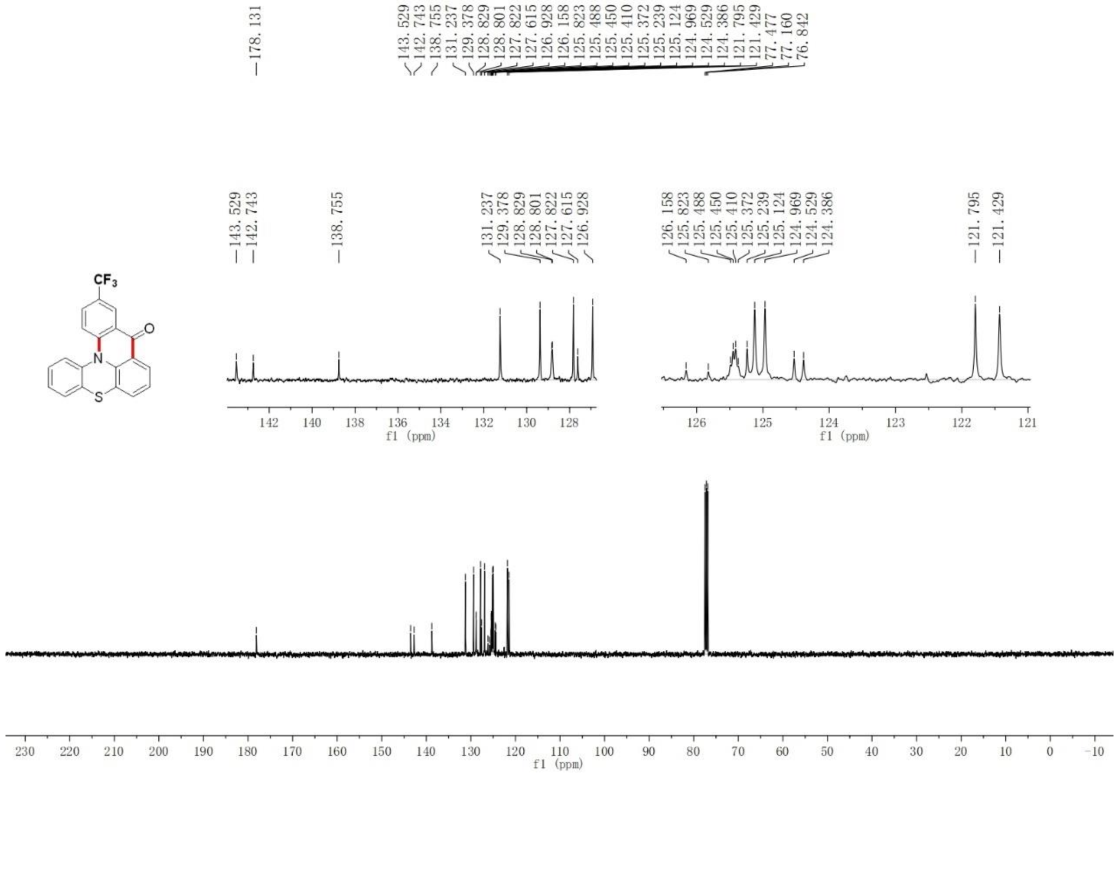
178.131
143.529 142.743 138.755 131.237 129.378 128.829 128.801 127.822 127.615 126.928 126.158 125.823 125.488 125.450 125.410 125.372 125.239 125.124 124.969 124.529 124.386 121.795 121.429 77.477 77.160 76.842
CF3
N
O
S
f1 (ppm)

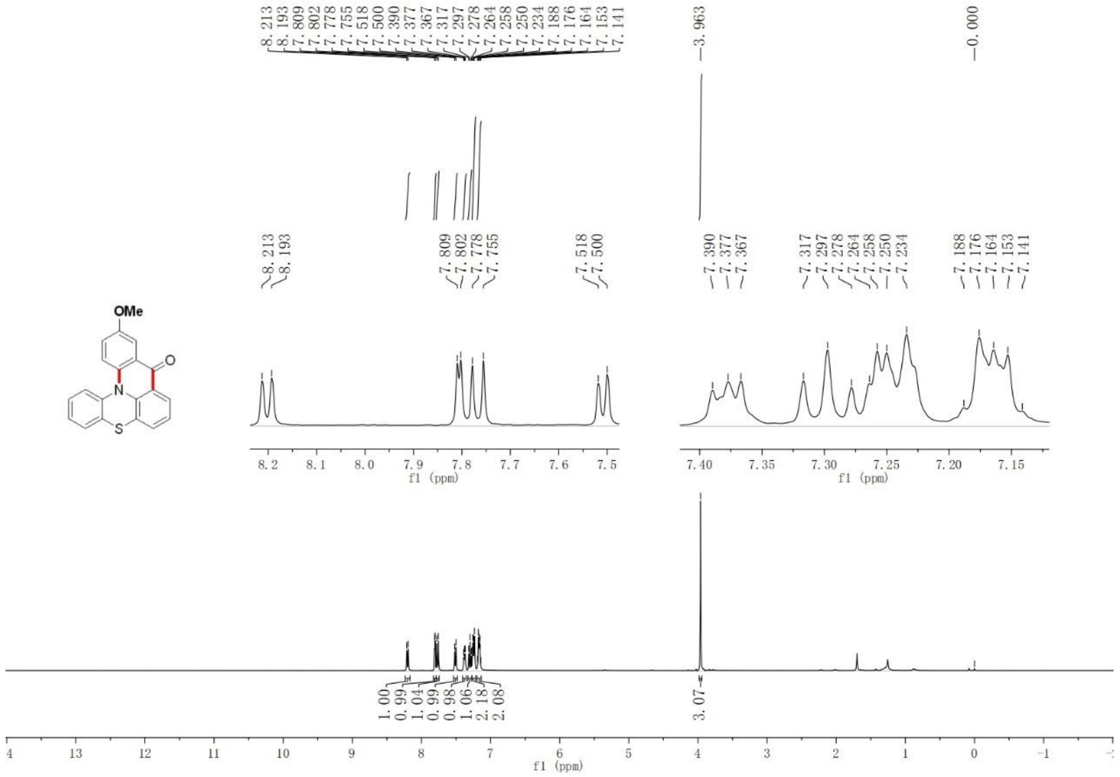
8.213
8.193
7.809
7.802
7.778
7.755
7.518
7.500
7.390
7.377
7.367
7.317
7.297
7.278
7.264
7.258
7.250
7.234
7.188
7.176
7.164
7.153
7.141
3.963
0.000
OMe
N
O
S
1.00
0.99
1.04
0.99
0.98
1.06
2.18
2.08
3.07
f1 (ppm)

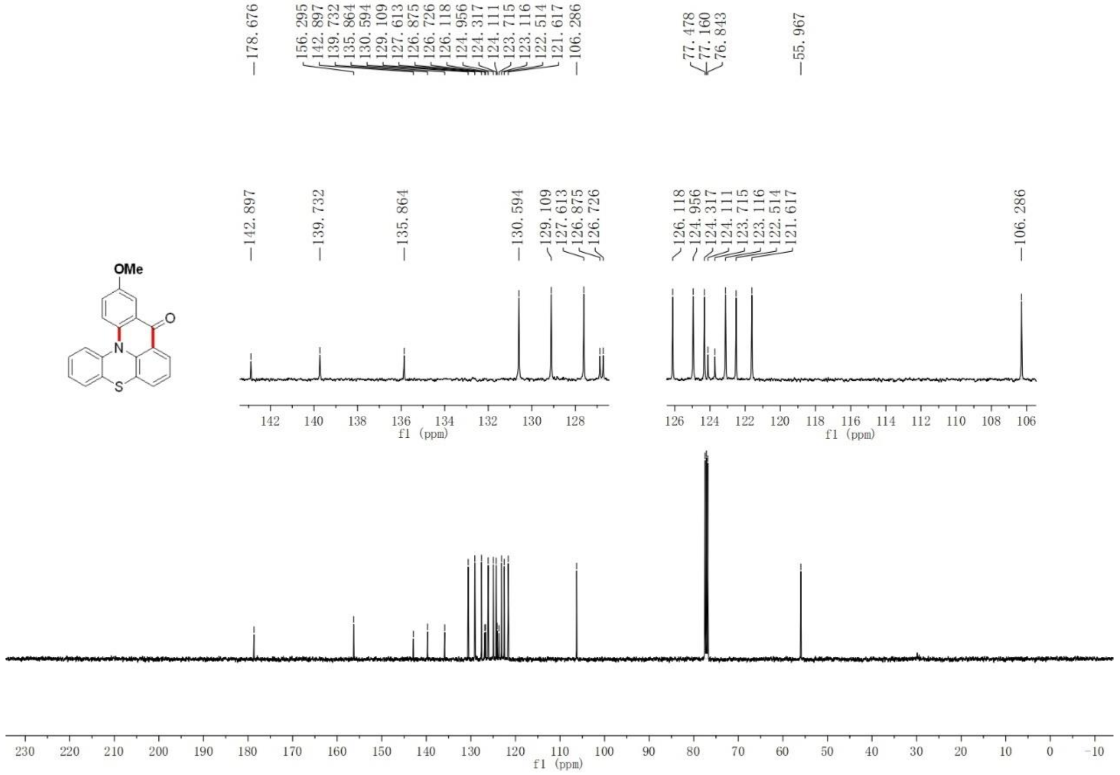
178.676
156.295
142.897
139.732
135.864
130.594
129.109
127.613
126.875
126.726
126.118
124.956
124.317
124.111
123.715
123.116
122.514
121.617
106.286
77.478
77.160
76.843
55.967
OMe
N
O
S
f1 (ppm)

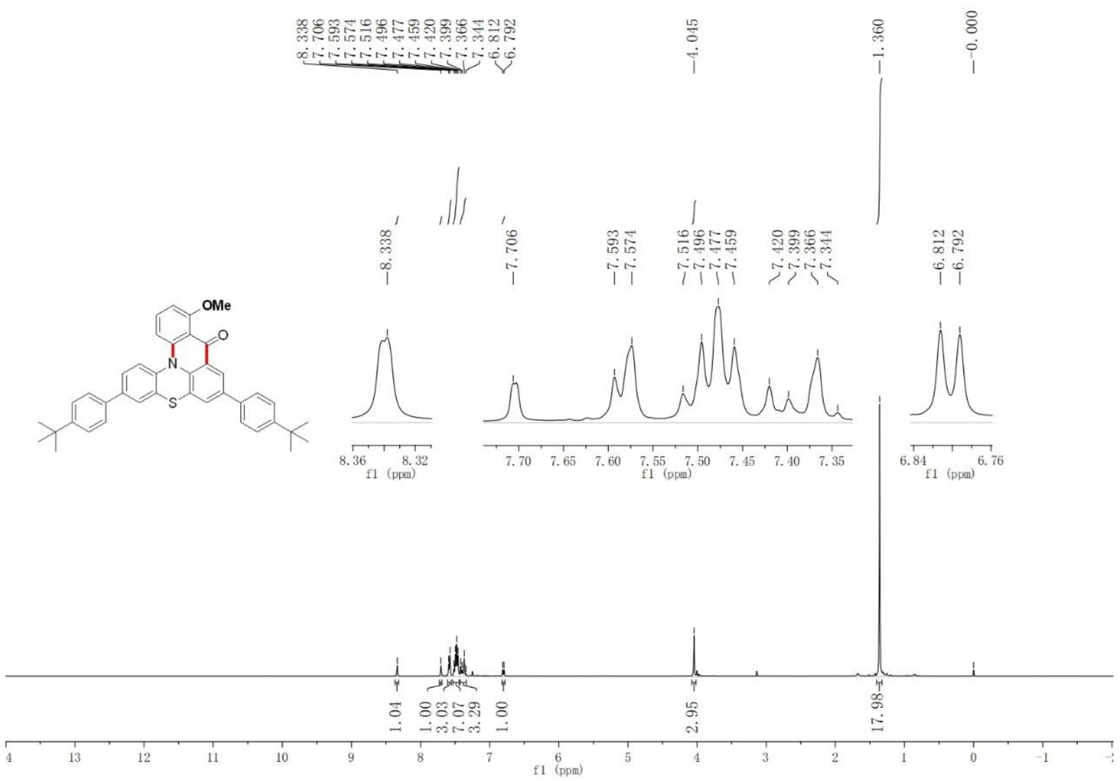

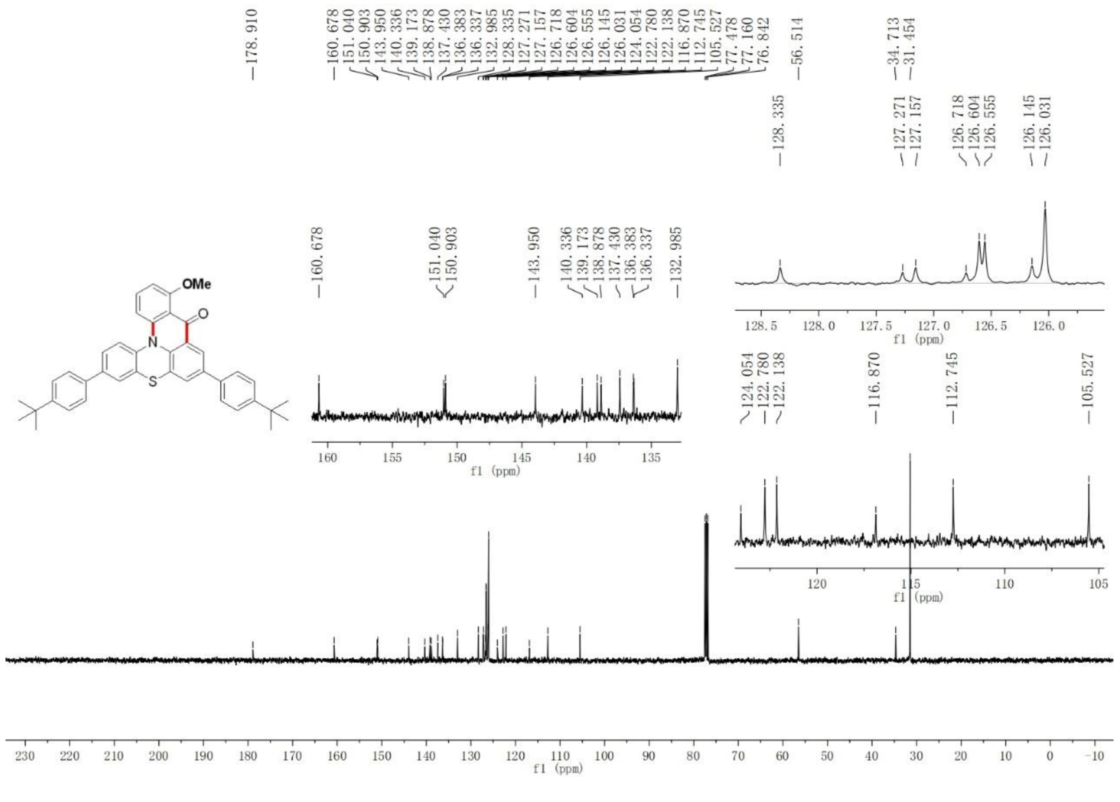

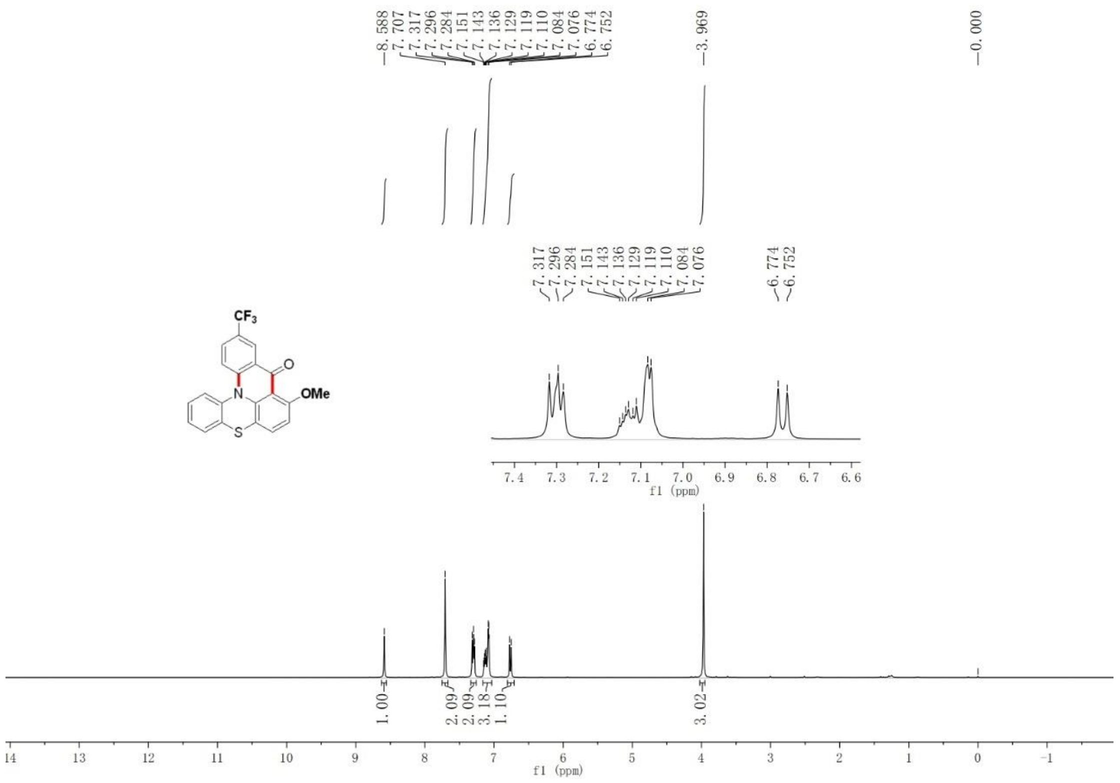
8.588
7.707
7.317
7.296
7.284
7.151
7.143
7.136
7.129
7.119
7.110
7.084
7.076
6.774
6.752
3.969
0.000
CF3
O
N
OMe
S
1.00
2.09
2.09
3.18
1.10
3.02
f1 (ppm)

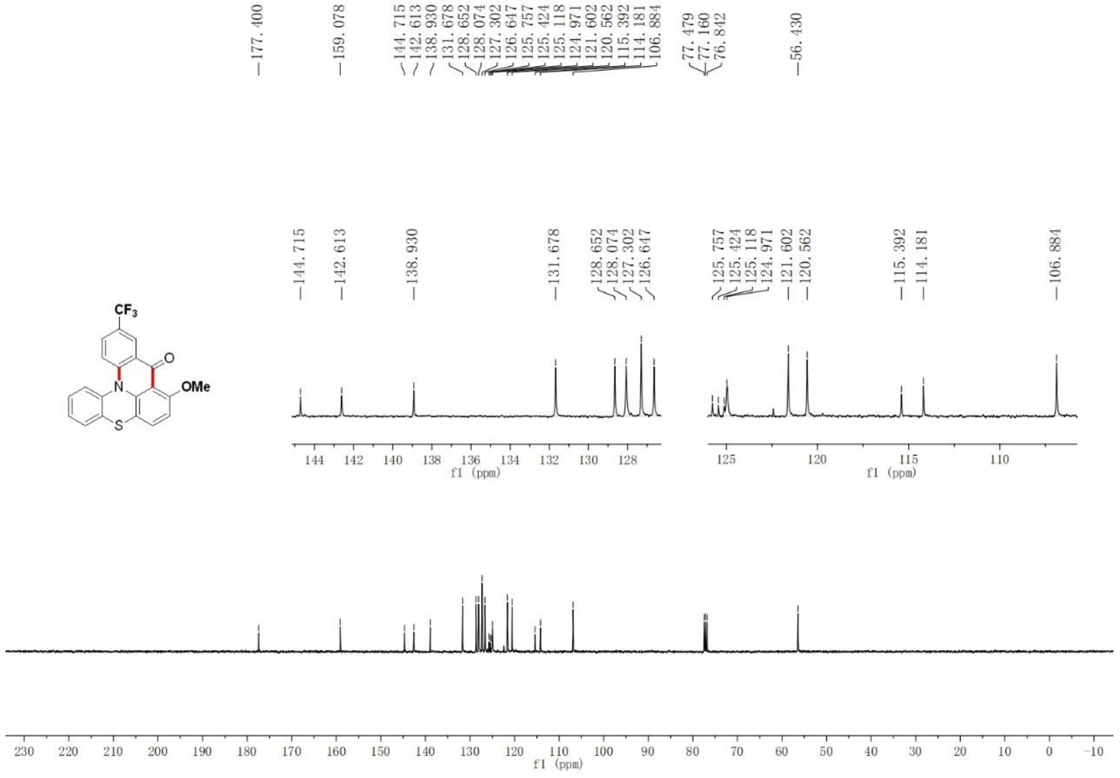
177.400
159.078
144.715
142.613
138.930
131.678
128.652
128.074
127.302
126.647
125.757
125.424
125.118
124.971
121.602
120.562
115.392
114.181
106.884
77.479
77.160
76.842
56.430
CF3
O
N
OMe
S
f1 (ppm)

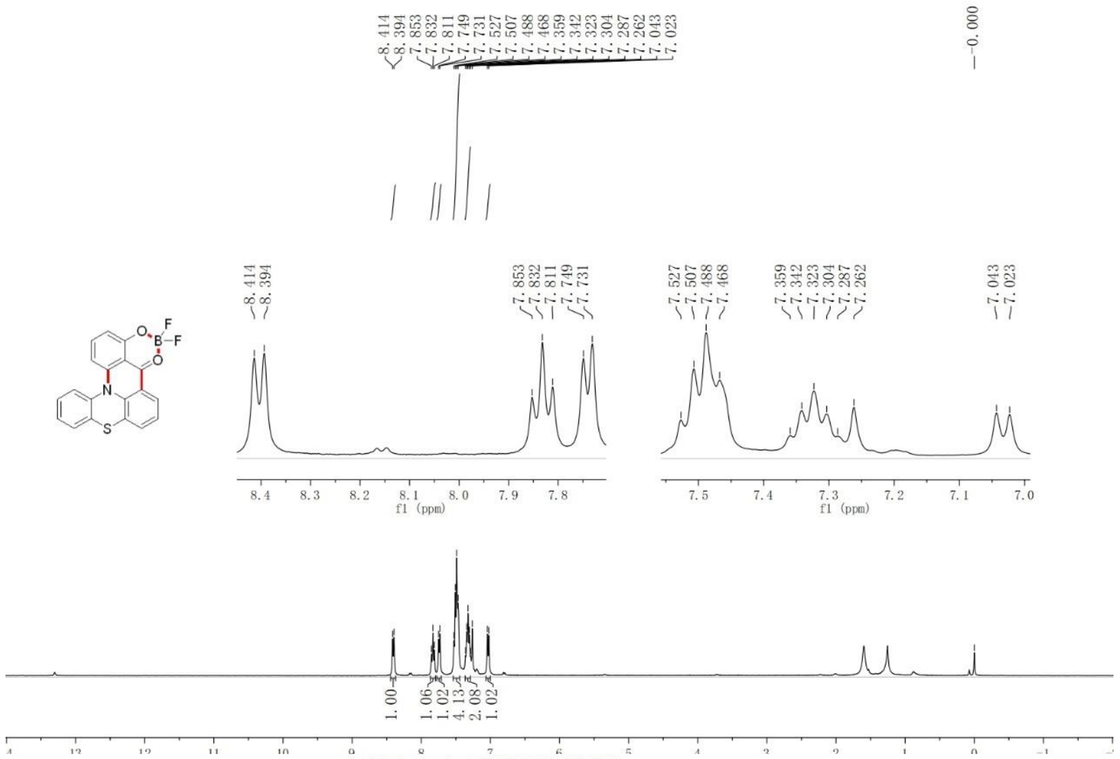
8.414
8.394
7.853
7.832
7.811
7.749
7.731
7.527
7.507
7.488
7.468
7.359
7.342
7.323
7.304
7.287
7.262
7.043
7.023
0.000
O
B
F
F
O
N
S
1.00
1.06
1.02
4.13
2.08
1.02
f1 (ppm)

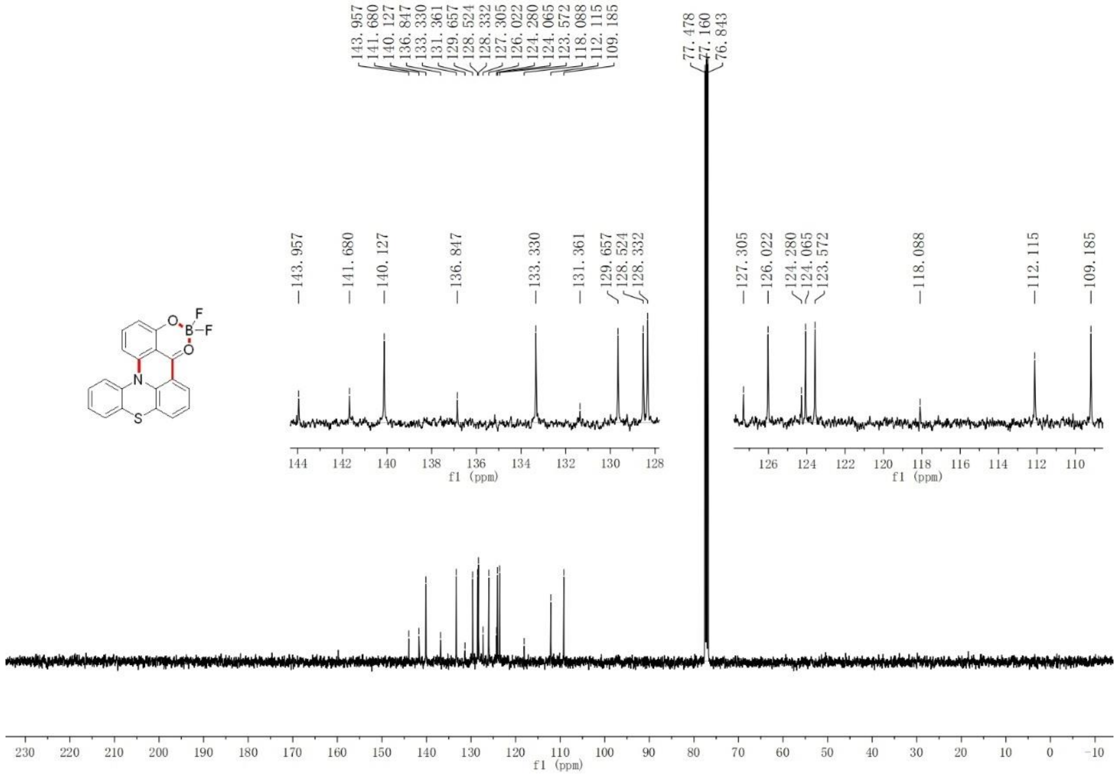
143.957
141.680
140.127
136.847
133.330
131.361
129.657
128.524
128.332
127.305
126.022
124.280
124.065
123.572
118.088
112.115
109.185
77.478
77.160
76.843
O
B
F
F
O
N
S
f1 (ppm)

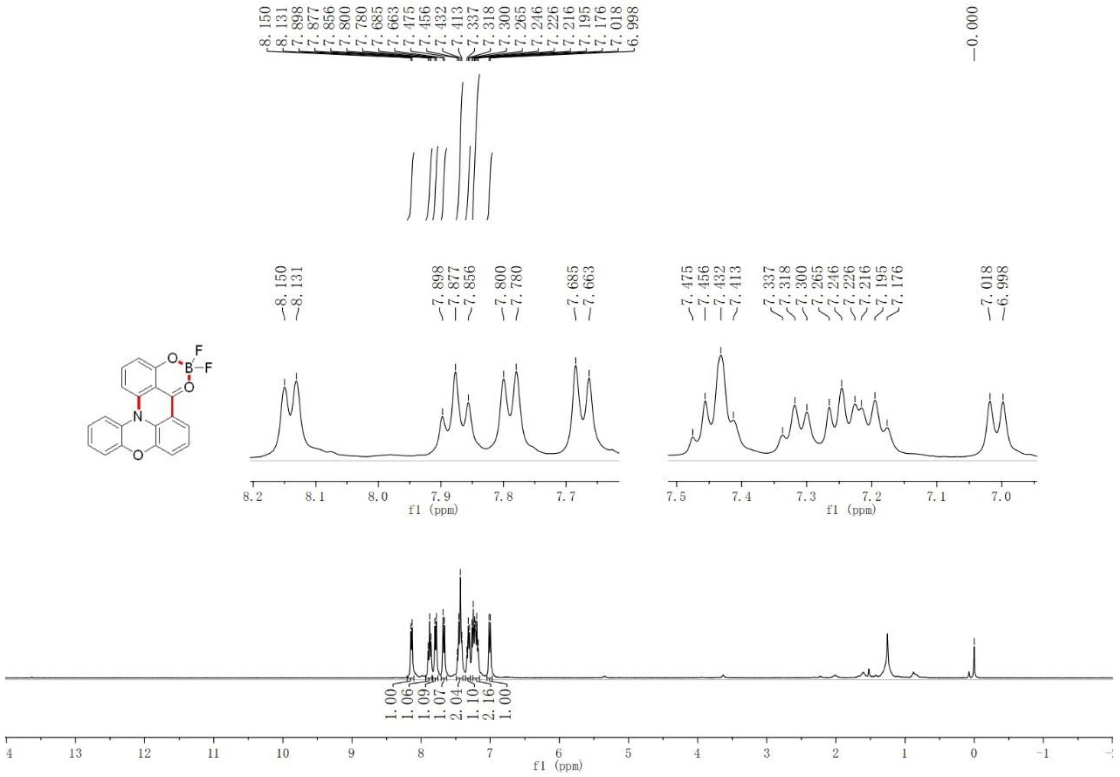
8.150
8.131
7.898
7.877
7.856
7.800
7.780
7.685
7.663
7.475
7.456
7.432
7.413
7.337
7.318
7.300
7.265
7.246
7.226
7.216
7.195
7.176
7.018
6.998
0.000
1.00
1.06
1.09
1.07
2.04
1.10
2.16
1.00
f1 (ppm)

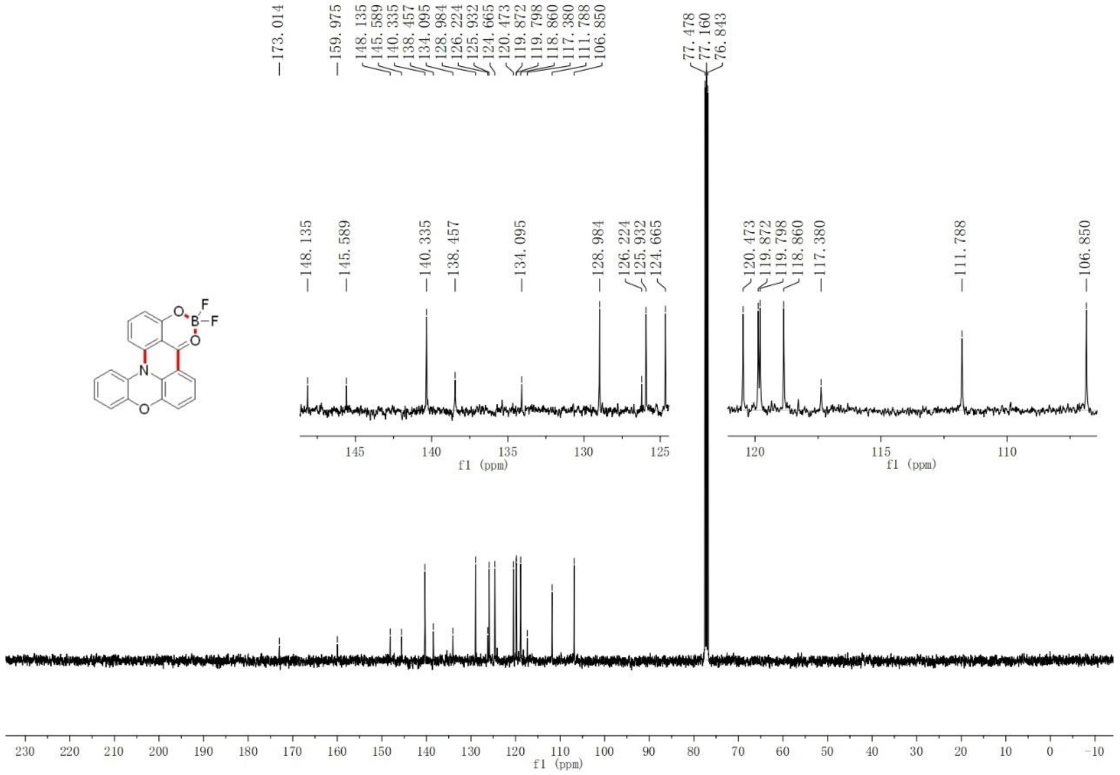
173.014
159.975
148.135
145.589
140.335
138.457
134.095
128.984
126.224
125.932
124.665
120.473
119.872
119.798
118.860
117.380
111.788
106.850
77.478
77.160
76.843
f1 (ppm)

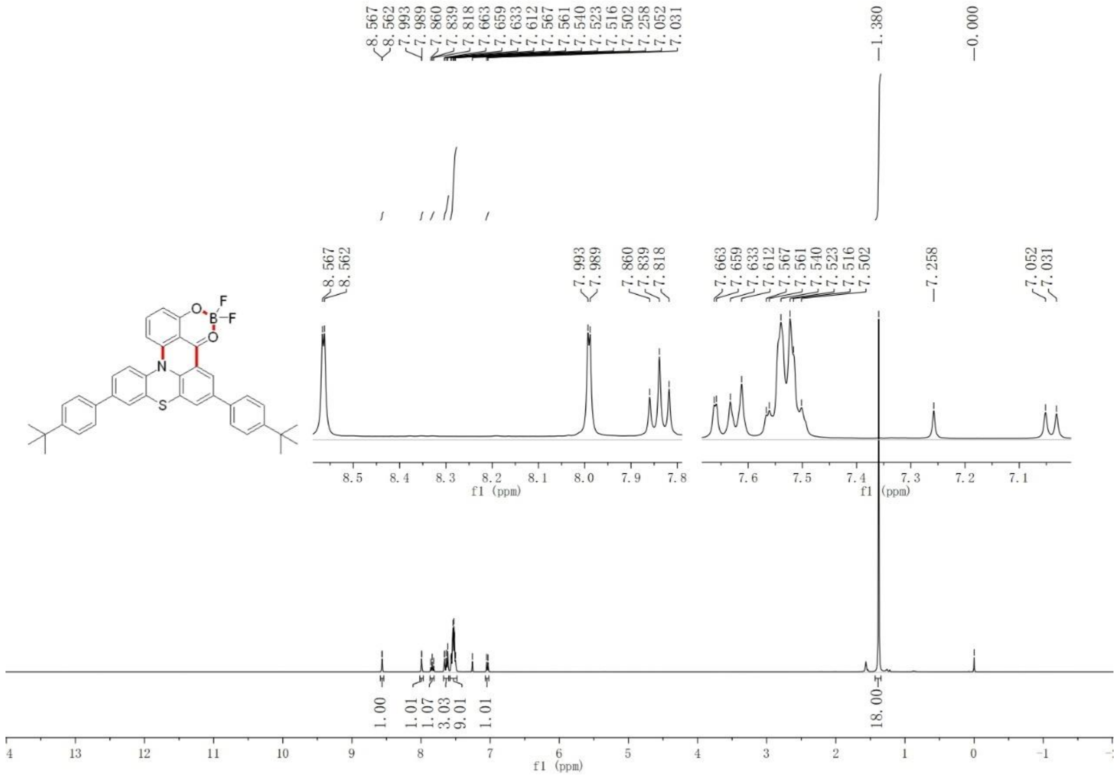
8.567 8.562 7.993 7.989 7.860 7.839 7.818 7.663 7.659 7.633 7.612 7.567 7.561 7.540 7.523 7.516 7.502 7.258 7.052 7.031
1.380
0.000
1.00 1.01 1.07 3.03 9.01 1.01
18.00
f1 (ppm)

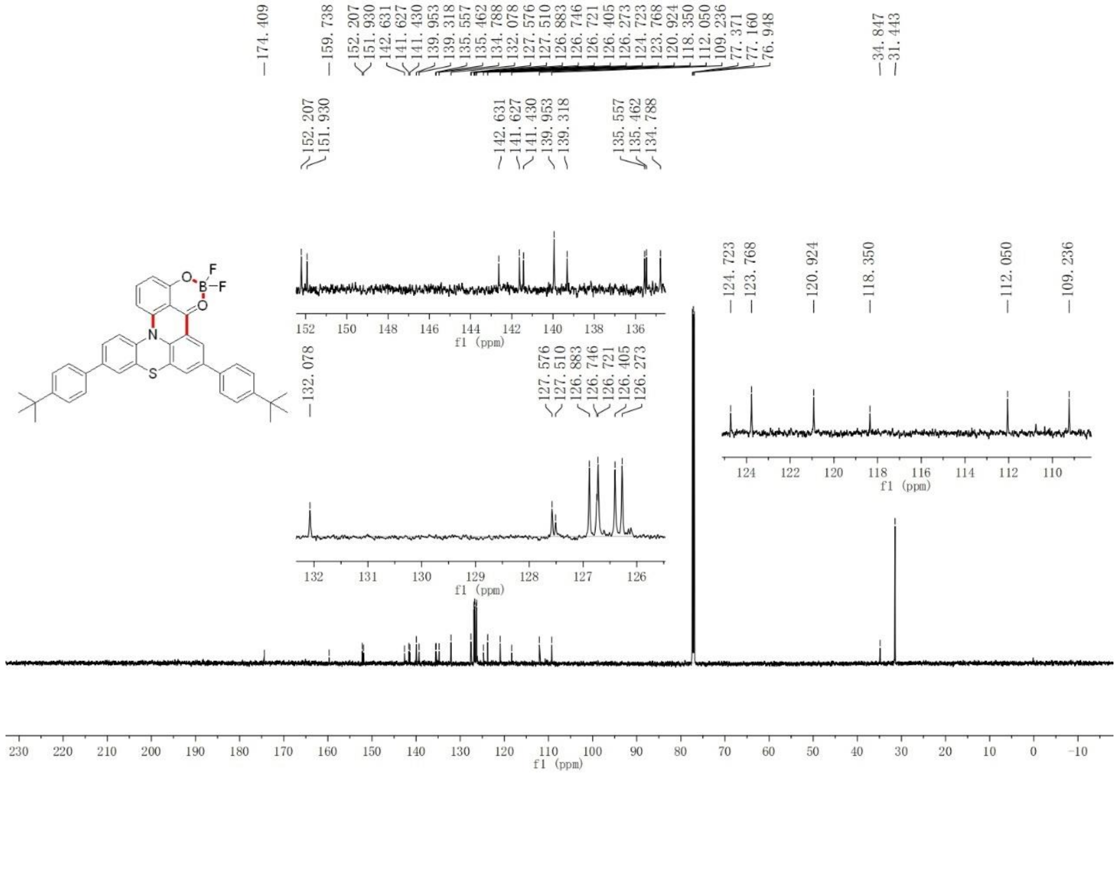
174.409
159.738
152.207 151.930 142.631 141.627 141.430 139.953 139.318 135.557 135.462 134.788 132.078 127.576 127.510 126.883 126.746 126.721 126.405 126.273 124.723 123.768 120.924 118.350 112.050 109.236 77.371 77.160 76.948
34.847
31.443
f1 (ppm)

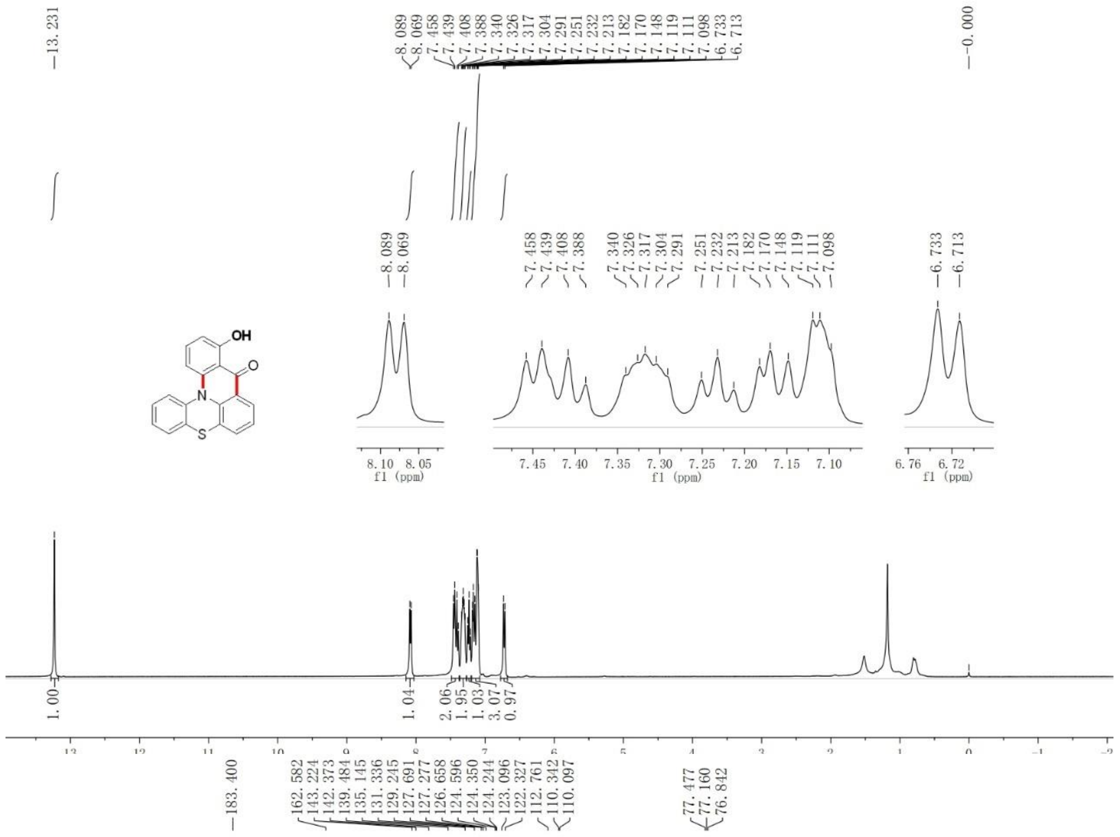
OH
O
N
S
f1 (ppm)

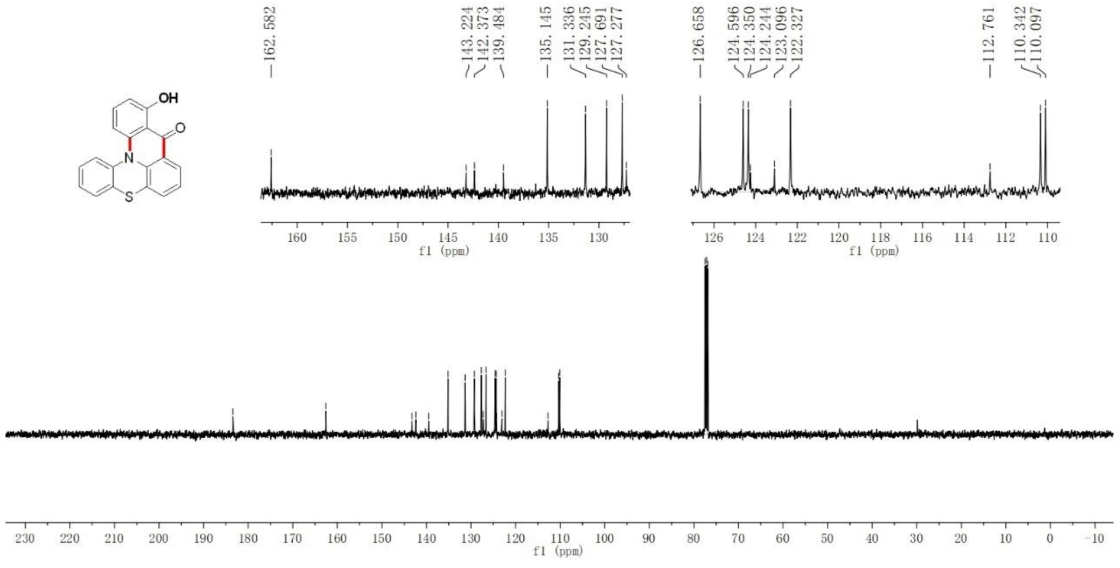
OH
O
N
S
f1 (ppm)

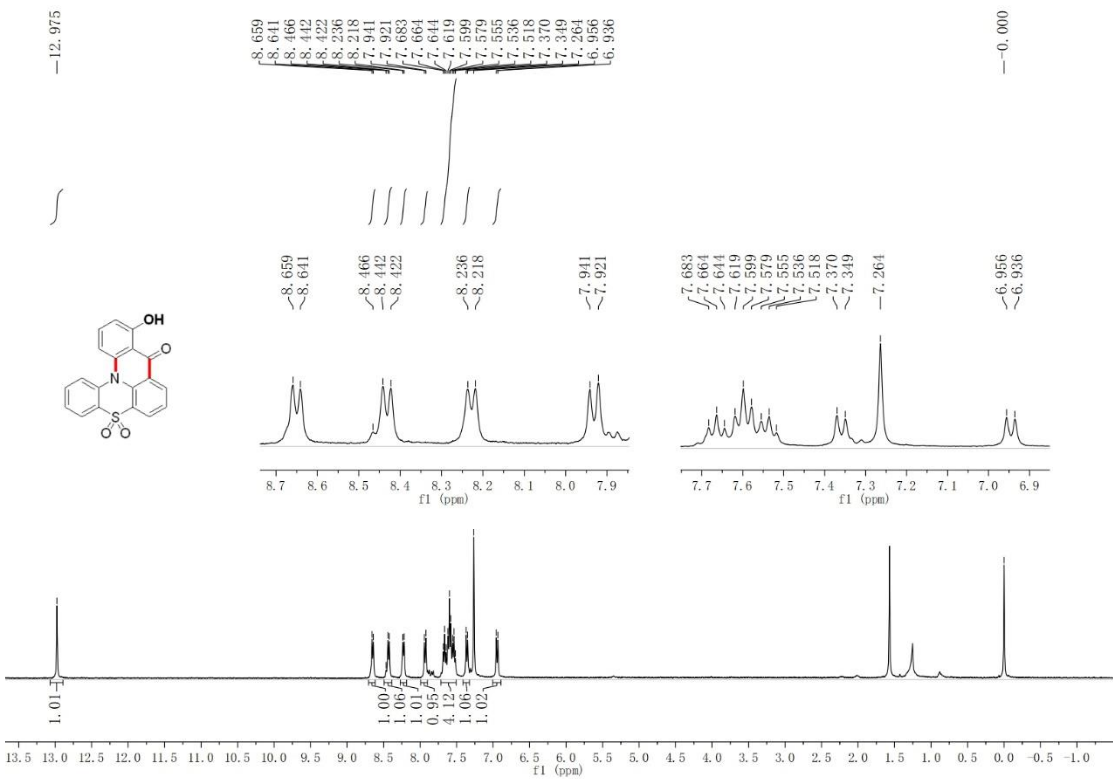
12.975
8.659 8.641 8.466 8.442 8.422 8.236 8.218 7.941 7.921 7.683 7.664 7.644 7.619 7.599 7.579 7.555 7.536 7.518 7.370 7.349 7.264 6.956 6.936
0.000
OH
O
N
S
O O
1.01
1.00 1.06 1.01 0.95 4.12 1.06 1.02
f1 (ppm)

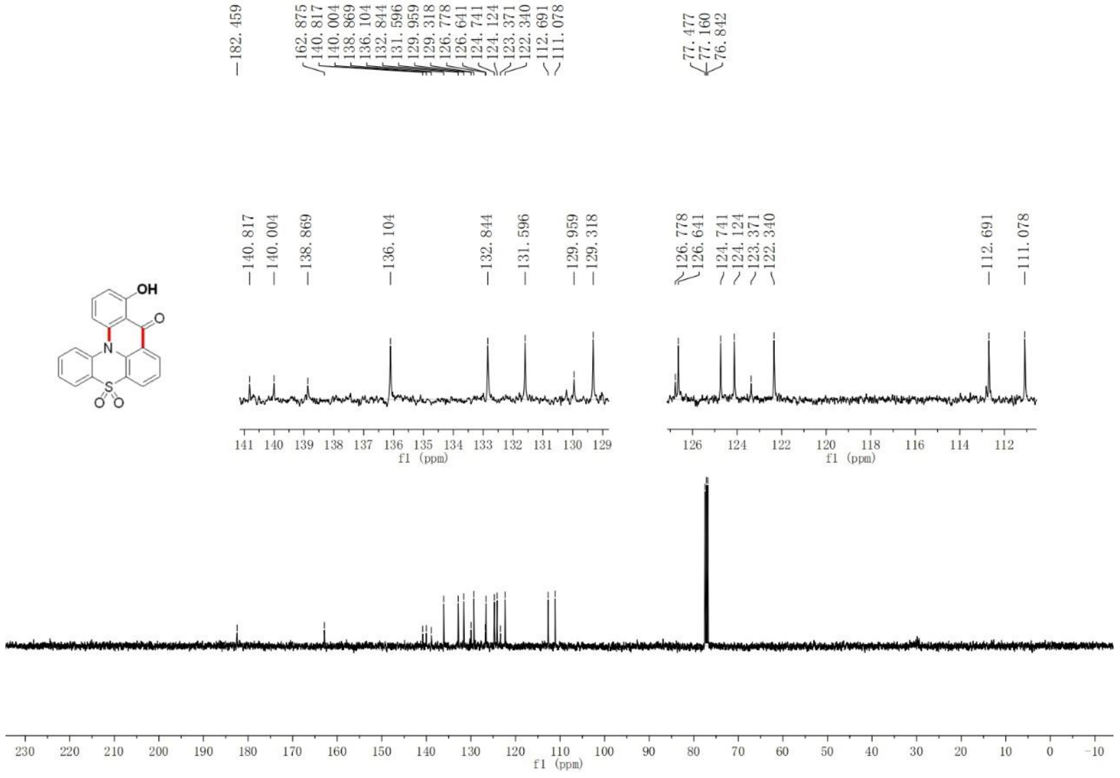
182.459
162.875 140.817 140.004 138.869 136.104 132.844 131.596 129.959 129.318 126.778 126.641 124.741 124.124 123.371 122.340 112.691 111.078
77.477 77.160 76.842
OH
O
N
S
O O
f1 (ppm)

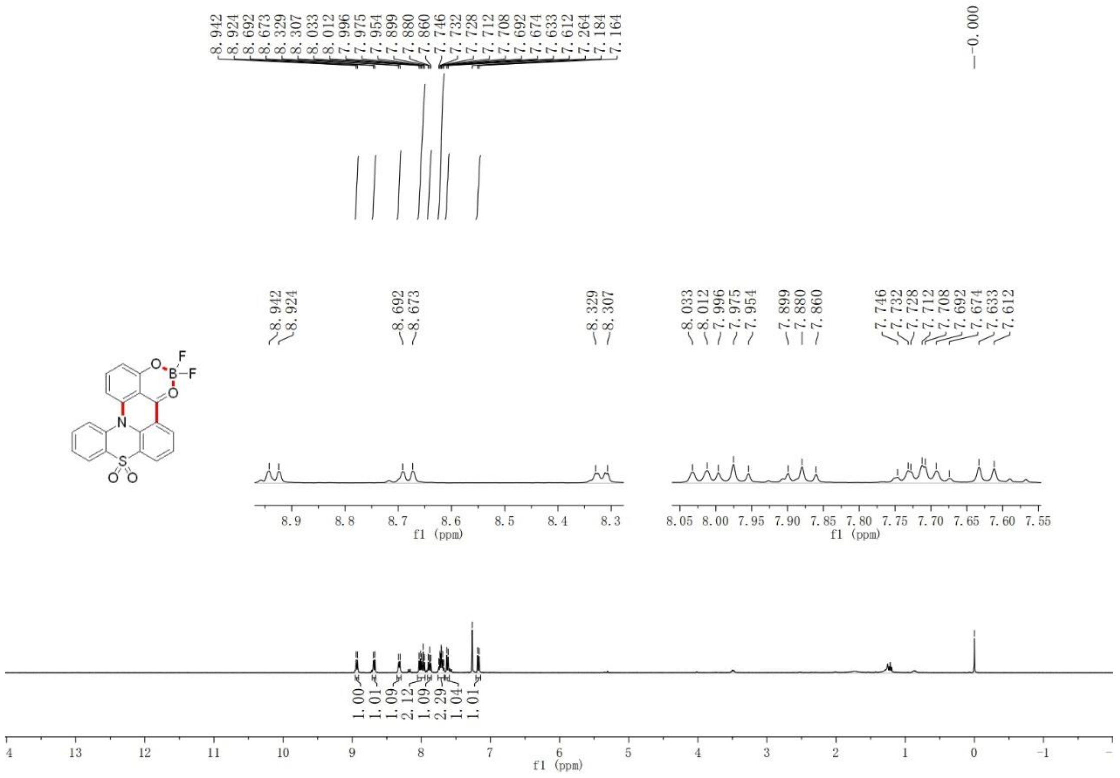
8.942 8.924 8.692 8.673 8.329 8.307 8.033 8.012 7.996 7.975 7.954 7.899 7.880 7.860 7.746 7.732 7.728 7.712 7.708 7.692 7.674 7.633 7.612 7.264 7.184 7.164
—0.000
O B F F O N S O O
1.00 1.01 1.09 2.12 1.09 2.29 1.04 1.01
f1 (ppm)

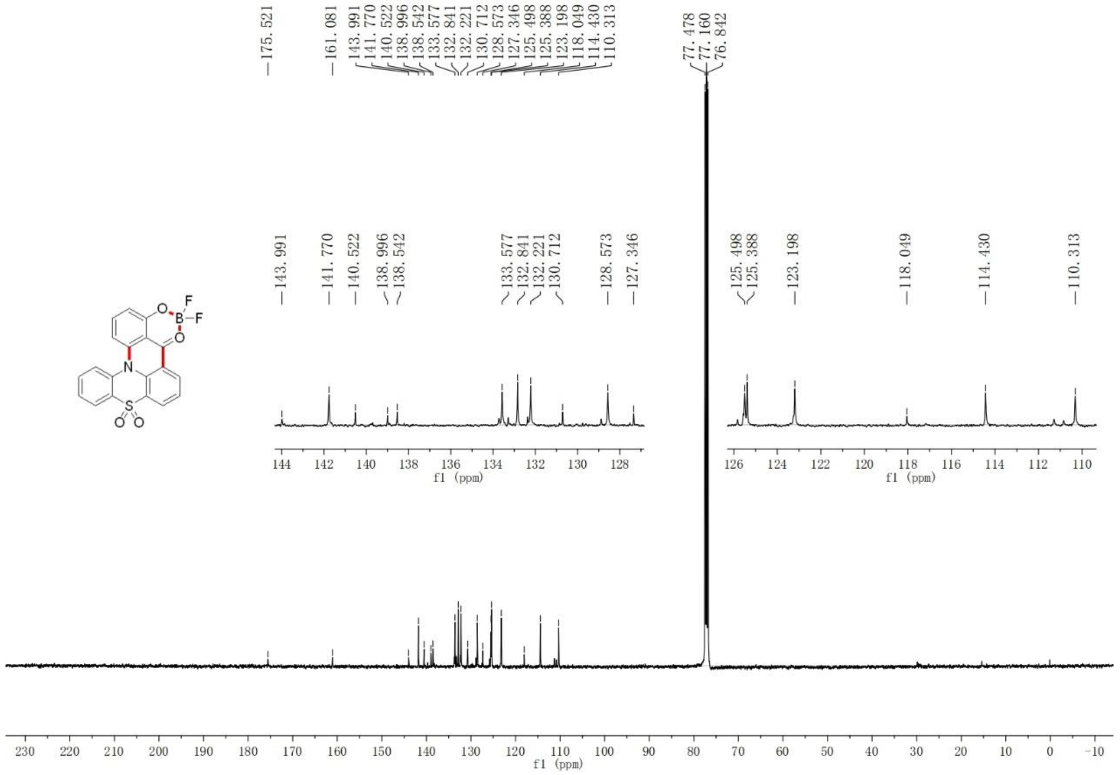
175.521 161.081 143.991 141.770 140.522 138.996 138.542 133.577 132.841 132.221 130.712 128.573 127.346 125.498 125.388 123.198 118.049 114.430 110.313
77.478 77.160 76.842
O B F F O N S O O
f1 (ppm)

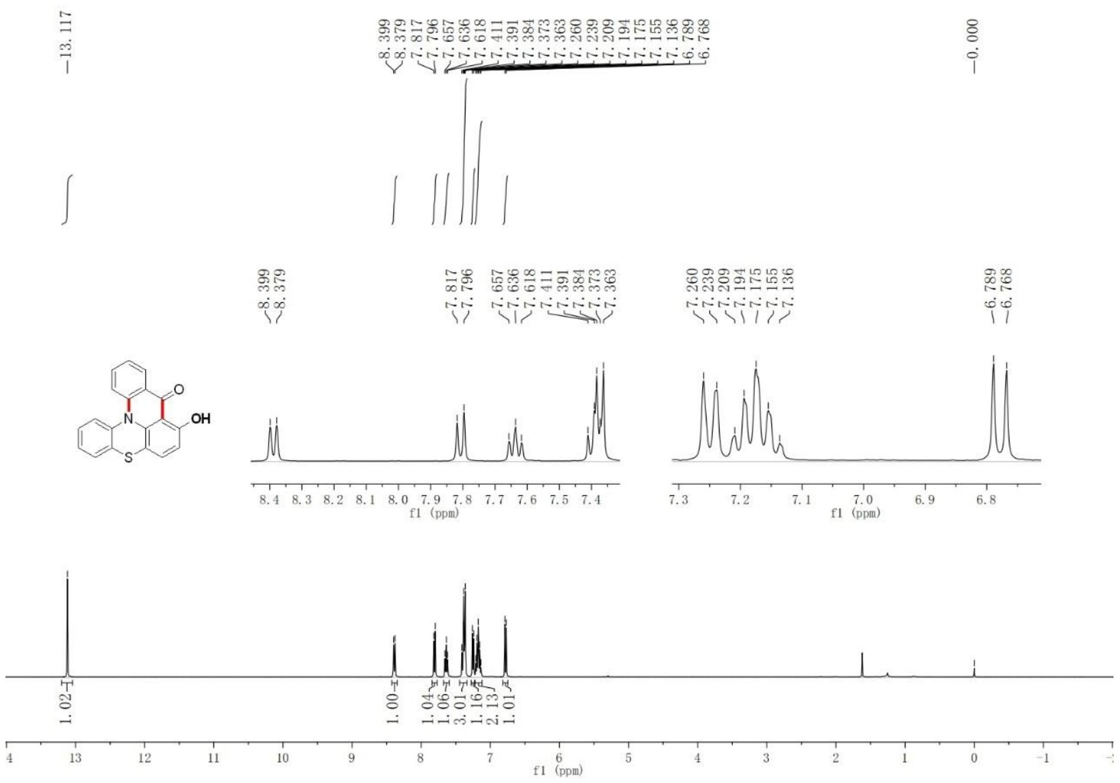
13.117
8.399 8.379 7.817 7.796 7.657 7.636 7.618 7.411 7.391 7.384 7.373 7.363 7.260 7.239 7.209 7.194 7.175 7.155 7.136 6.789 6.768
0.000
OH
N
S
1.02 1.00 1.04 1.06 3.01 1.16 2.13 1.01
f1 (ppm)

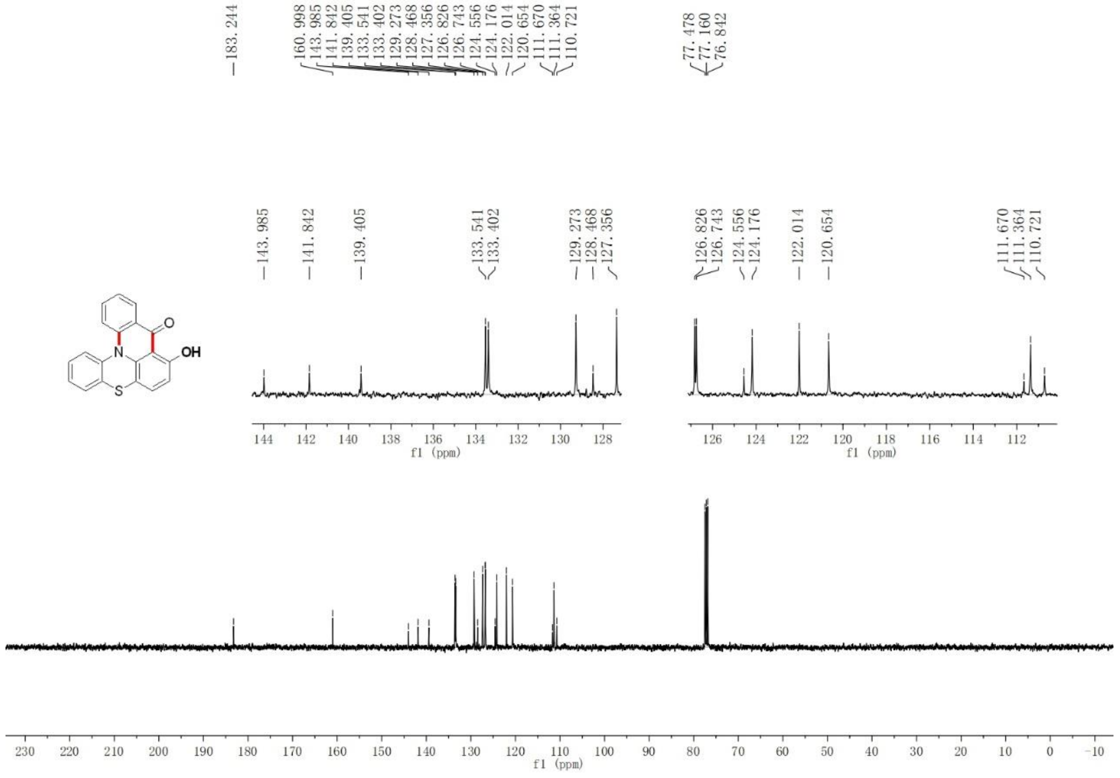
183.244
160.998 143.985 141.842 139.405 133.541 133.402 129.273 128.468 127.356 126.826 126.743 124.556 124.176 122.014 120.654 111.670 111.364 110.721
77.478 77.160 76.842
OH
N
S
f1 (ppm)

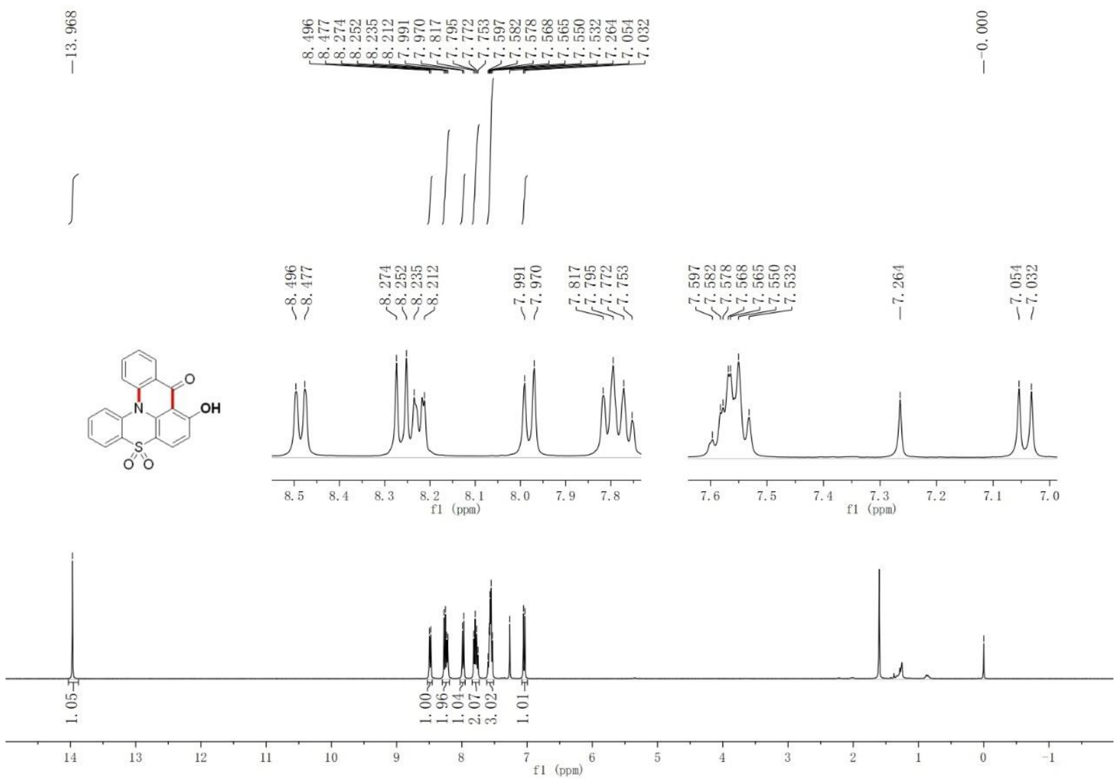
13.968
8.496 8.477 8.274 8.252 8.235 8.212 7.991 7.970 7.817 7.795 7.772 7.753 7.597 7.582 7.578 7.568 7.565 7.550 7.532 7.264 7.054 7.032
0.000
OH
N
S
O O
1.05
1.00 1.96 1.04 2.07 3.02 1.01
f1 (ppm)

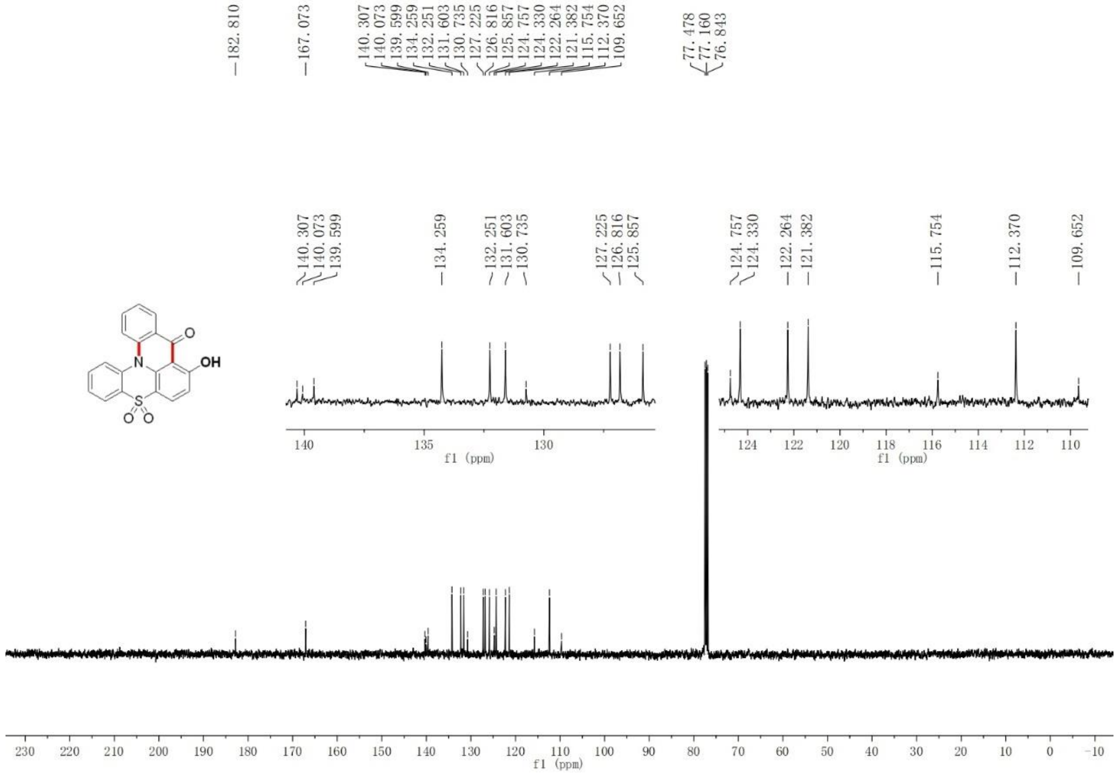
182.810
167.073
140.307 140.073 139.599 134.259 132.251 131.603 130.735 127.225 126.816 125.857 124.757 124.330 122.264 121.382 115.754 112.370 109.652
77.478 77.160 76.843
OH
N
S
O O
f1 (ppm)

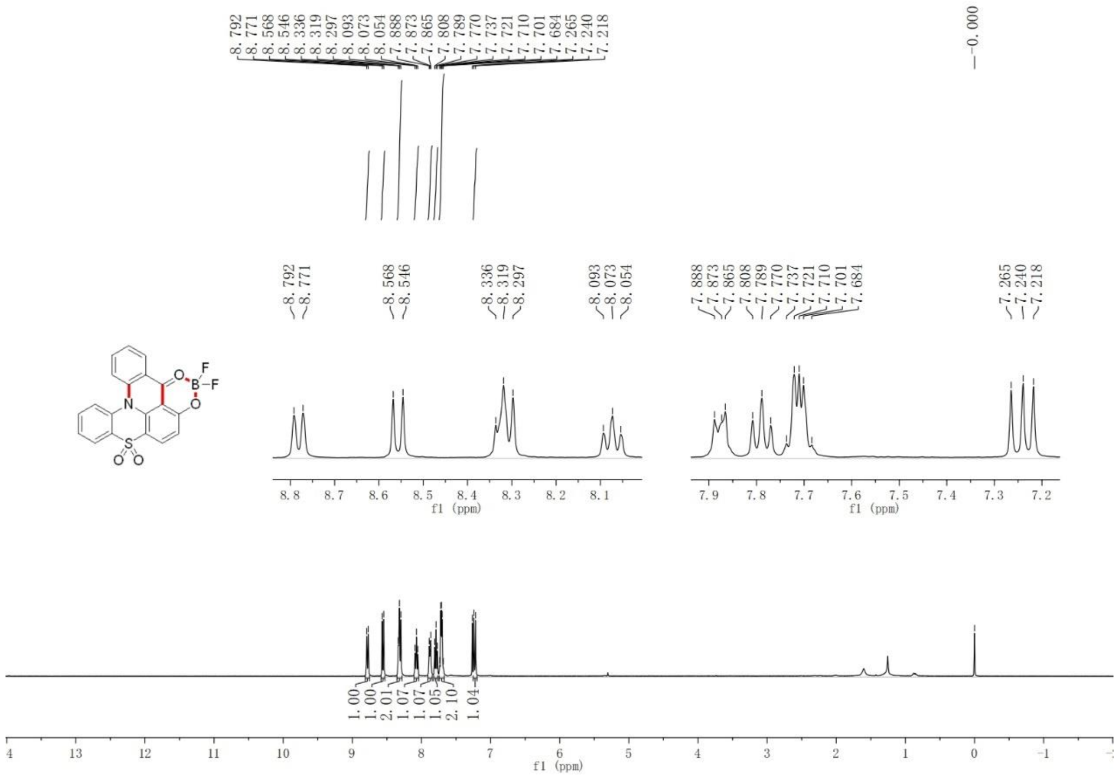
8.792
8.771
8.568
8.546
8.336
8.319
8.297
8.093
8.073
8.054
7.888
7.873
7.865
7.808
7.789
7.770
7.737
7.721
7.710
7.701
7.684
7.265
7.240
7.218
0.000
1.00
1.00
2.01
1.07
1.07
1.05
2.10
1.04
f1 (ppm)

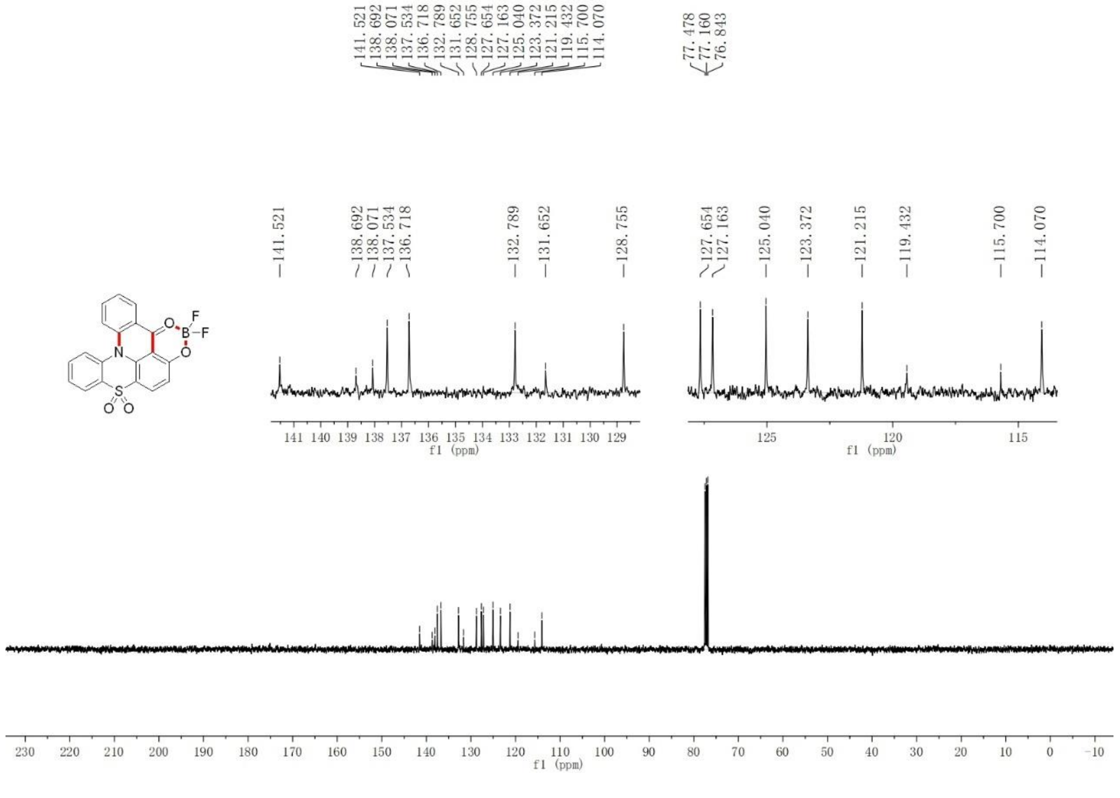
141.521
138.692
138.071
137.534
136.718
132.789
131.652
128.755
127.654
127.163
125.040
123.372
121.215
119.432
115.700
114.070
77.478
77.160
76.843
f1 (ppm)

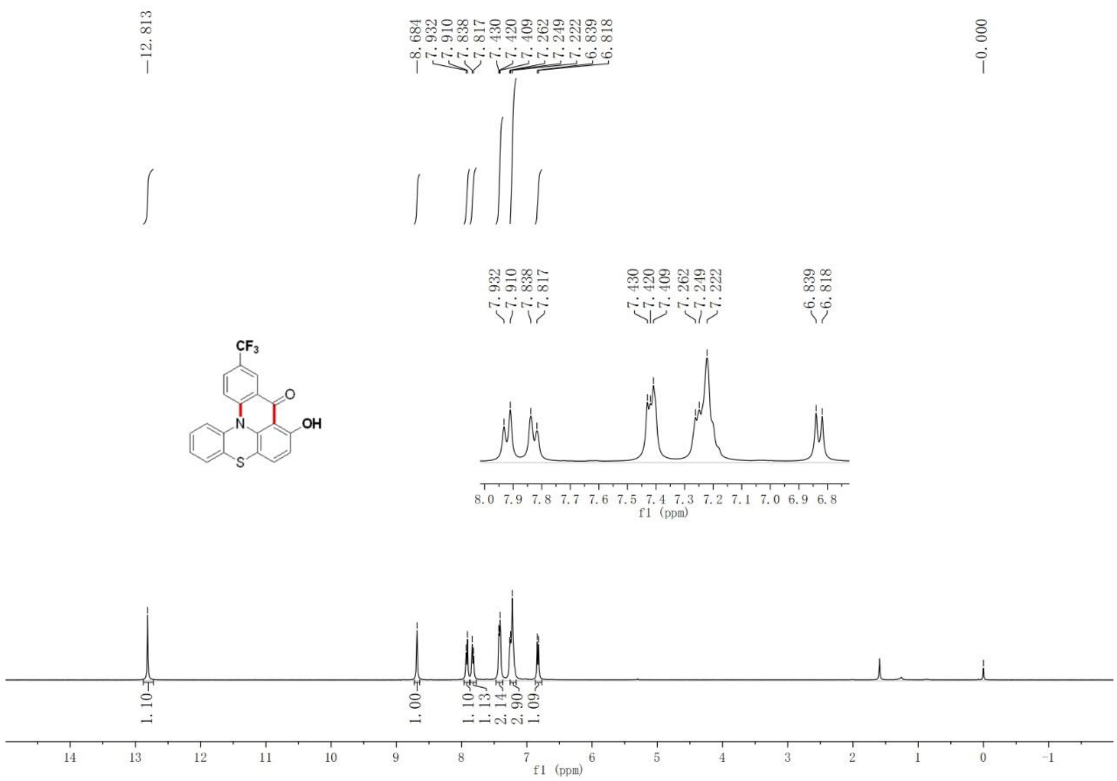
12.813
8.684
7.932
7.910
7.838
7.817
7.430
7.420
7.409
7.262
7.249
7.222
6.839
6.818
0.000
CF3
OH
N
S
1.10
1.00
1.10
1.13
2.14
2.90
1.09
f1 (ppm)

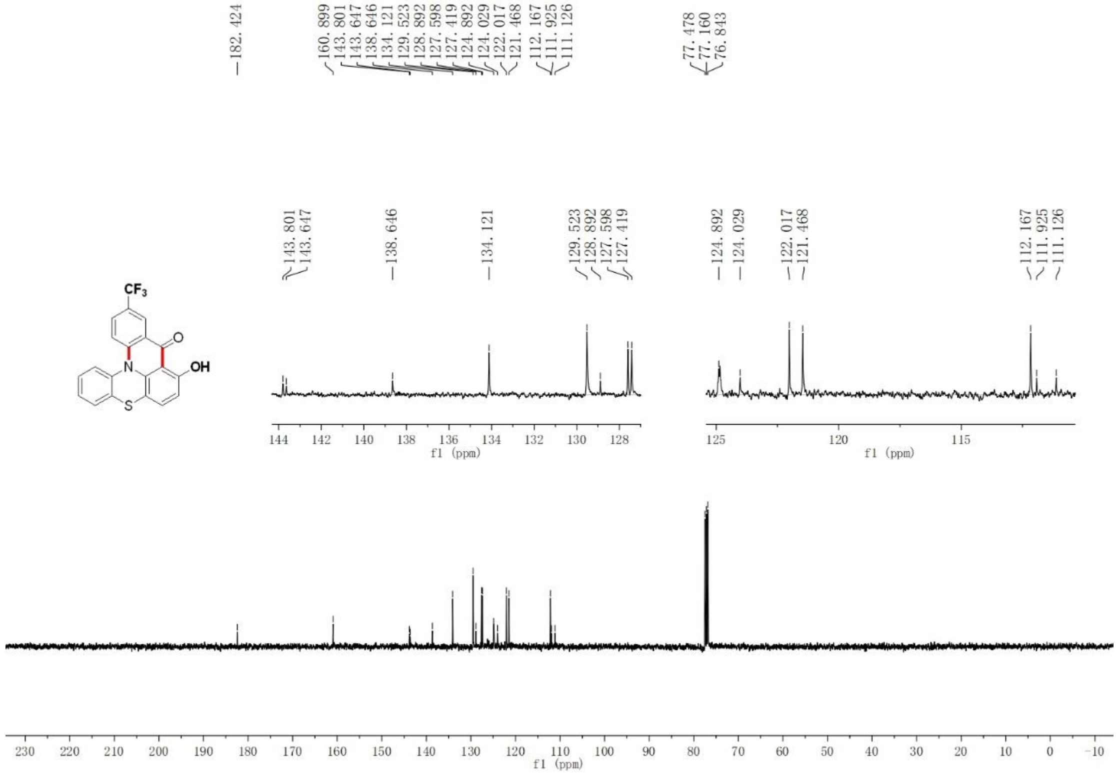
182.424
160.899
143.801
143.647
138.646
134.121
129.523
128.892
127.598
127.419
124.892
124.029
122.017
121.468
112.167
111.925
111.126
77.478
77.160
76.843
CF3
OH
N
S
f1 (ppm)

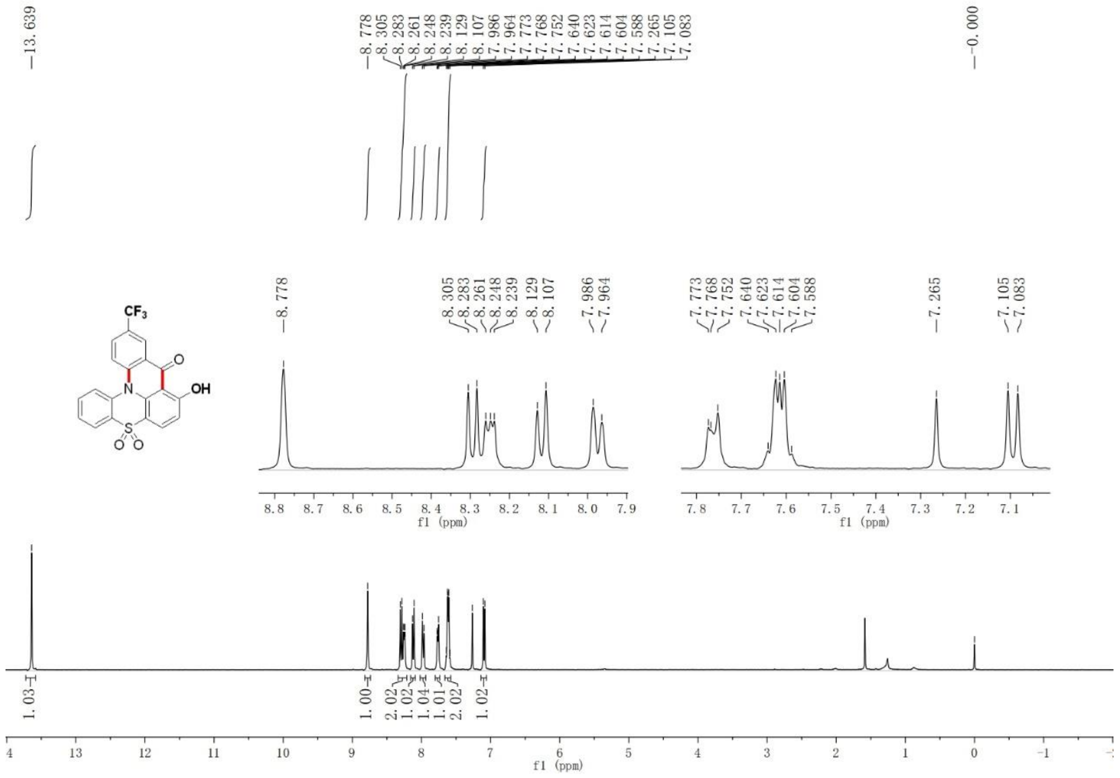

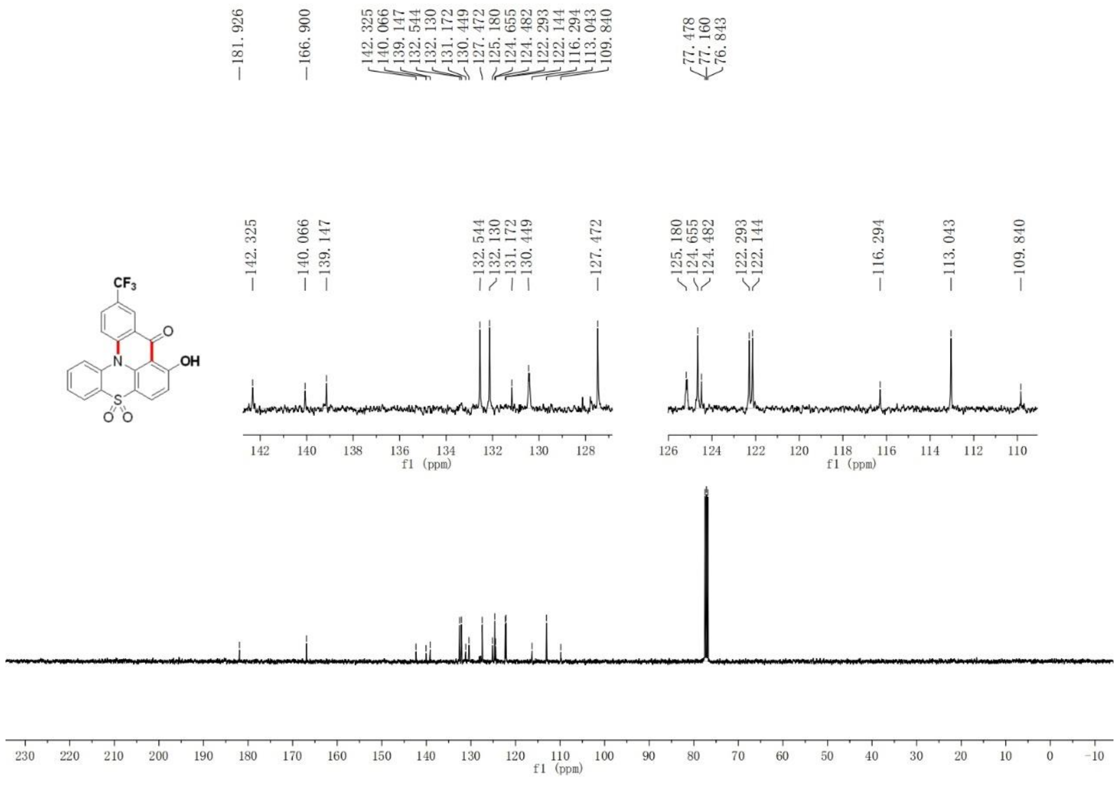

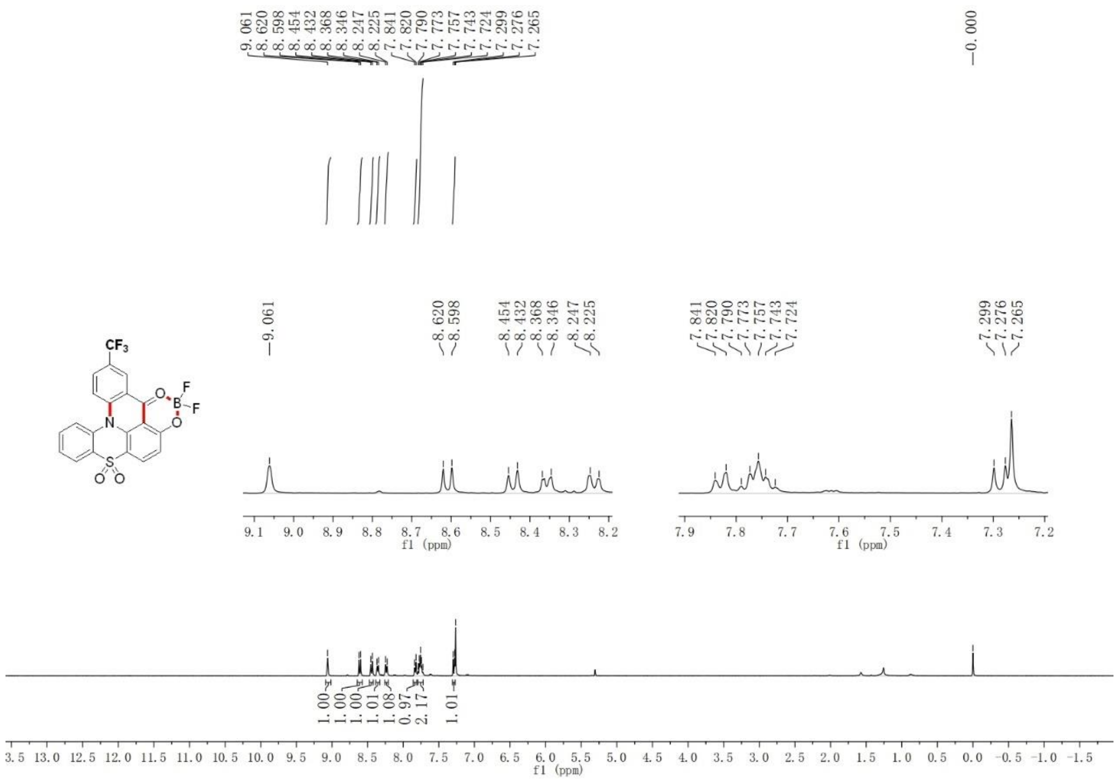
9.061
8.620
8.598
8.454
8.432
8.368
8.346
8.247
8.225
7.841
7.820
7.790
7.773
7.757
7.743
7.724
7.299
7.276
7.265
0.000
CF3
O
B
F
F
O
N
S
O O
1.00
1.00
1.00
1.01
1.08
0.97
2.17
1.01
f1 (ppm)

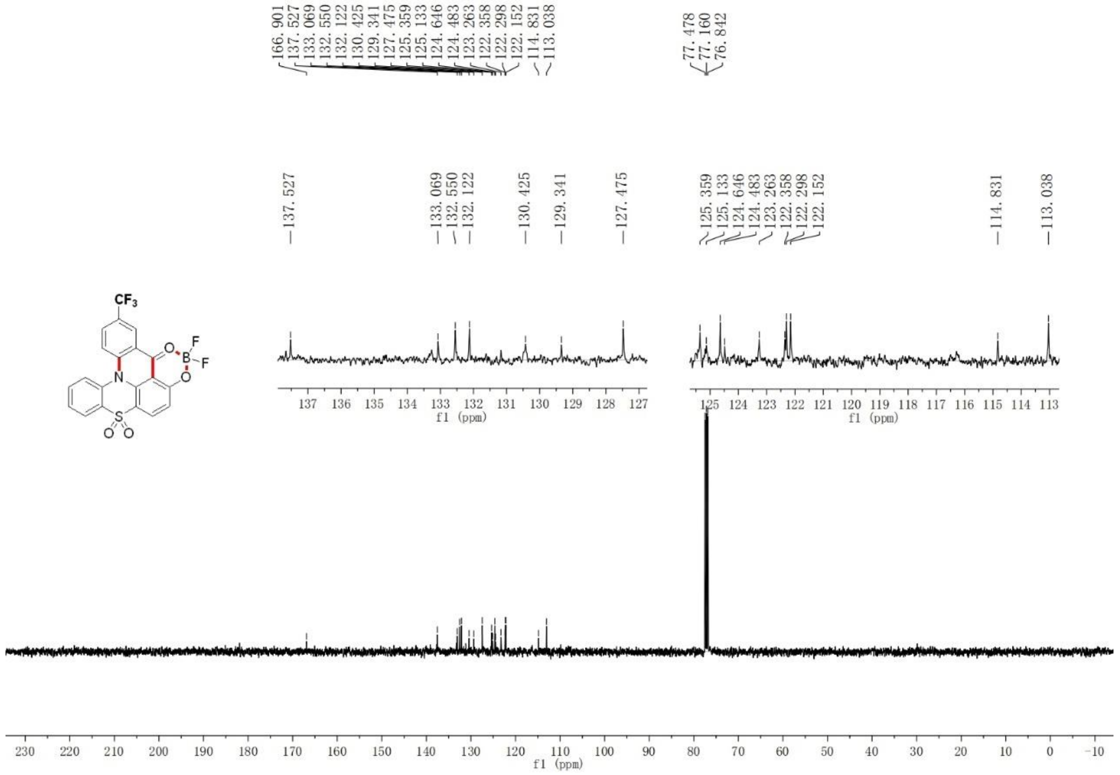
166.901
137.527
133.069
132.550
132.122
130.425
129.341
127.475
125.359
125.133
124.646
124.483
123.263
122.358
122.298
122.152
114.831
113.038
77.478
77.160
76.842
CF3
O
B
F
F
O
N
S
O O
f1 (ppm)
